# Spatio—Temporal distribution of a vector of cutaneous leishmaniasis: *Pintomyia longiflocosa*, in a population from the Colombian Andean Mountains

**DOI:** 10.1371/journal.pntd.0012237

**Published:** 2024-06-17

**Authors:** Astrid Muñoz-Ortiz, Miguel Beltrán, Jennifer Vargas Durango, Gelys Mestre, Erika Santamaria Herreño, Jesús E. Escovar

**Affiliations:** 1 Escuela de Ciencias Básicas y Aplicadas, Universidad de La Salle, Bogotá D.C., Colombia; 2 Grupo de Entomología, Instituto Nacional de Salud, Bogotá, Colombia; University of Antwerp Drie Eiken Campus: Universiteit Antwerpen Campus Drie Eiken, BELGIUM

## Abstract

**Background:**

Leishmaniasis, a neglected disease and public health concern, is associated with various factors such as biological, social, economical conditions and climate, increasing the risk of human infection. Understanding the population dynamics of the vectors, like *Pintomyia longiflocosa*, and its relationship with ecological variables is crucial for developing effective strategies to control sand fly populations and combat cutaneous leishmaniasis in a tropical country like Colombia.

**Methodology:**

Adult sand flies were collected in three different sample locations: outdoor, indoor, and peri-domestic areas in three houses located in the rural settlement of Campoalegre (Huila) between February 2020 and February 2021, using the CDC light traps. The sand fly density was quantified and associated with the sample locations and the sampling months using Analysis of Variance and Pearson correlations.

**Principal findings:**

In the period of the sample, 98.86% of sand fly collected was identified as *Pi*. *longiflocosa*. The density of this species was significantly different between males and females, the latter contributing more to density in all sample locations (*P<*0.0001). The outdoor was the sample location with the highest and most significative density in this study (70%, *P =* 0.04). The density of these sand flies is related to the seasonality of Campoalegre, revealing a density peak from February and June to October (*P <* 0.05). Finally, precipitation is the environmental variable prominently linked to the density pattern, showing a negative correlation with it. Months with the highest precipitations show the lowest values of *Pi*. *longiflocosa* abundance.

**Conclusions/Signicance:**

Our investigation reveals a inverse correlation between precipitation levels and the abundance of *Pi*. *longiflocosa* in Campoalegre (Huila), particularly in outdoor areas. This suggests that vector control strategies to periods of reduced precipitation in outdoor settings could offer an effective approach to minimizing cases of cutaneous leishmaniasis in the region.

## Author summary

Understanding the population dynamics of *Pintomyia longiflocosa* (named before like *Lutzomyia longiflocosa*), and its interaction with ecological variables is crucial for devising effective strategies to control sand fly populations and combat cutaneous leishmaniasis, a neglected tropical disease, and a significant public health challenge in tropical countries like Colombia. To investigate this, we collected adult sand flies from outdoor, indoor, and peri-domestic areas in Campoalegre, Huila, Colombia, The density of *Pi*. *longiflocosa*, comprising 98.86% of the collected sand flies, with females contributing more to the overall density. Outdoor areas exhibited the highest density, highlighting their importance in the transmission of the disease. Additionally, the density of this sand flies showed a seasonal pattern, peaking from February to June and October, aligning with the local climate conditions. Notably, precipitation emerged as a significant environmental variable, displaying a negative correlation with sand fly density. Months with higher rainfall had lower abundance of *Pi*. *longiflocosa*. Overall, our study provides valuable insights into the population dynamics of the sand fly vector and its correlation with ecological factors, paving the way for effective control strategies to combat cutaneous leishmaniasis in Colombia.

## Introducction

Leishmaniasis is a disease product of the invasion of several species of *Leishmania*, an obligate intracellular pathogen transmitted by the bites of sand fly species’ vectors (Diptera: Psychodidae: Phlebotominae) on either humans or animals [1,2]. Out of about 900 recognized sandfly species, 98 are responsible for transmitting at least 20 species of *Leishmania* [3]. The clinical manifestations of this disease are diverse, including cutaneous (ZCL), diffuse cutaneous, mucocutaneous (MCL), visceral (VL, also known as the kala-azar), and post kala-azar dermal leishmaniasis (PKDL) [2,4,5].

Leishmaniasis endemicity was described in 2021, showing that of the more 200 countries and territories reported to the World Health Organization (WHO), 99 (49%) were considered endemic [1,5,6]. The risk for human leishmaniasis is associated with the genetic background of the population including socioeconomic status, environmental and climatic conditions, demographic, and human behaviors [1,2,7,8]. This risk is high in some of these endemic regions converting this neglected disease into a public health problem in areas around Mediterranean Basin, Eastern Africa, Southeastern Asia, and the Americas [1].

In the region of the Americas, three different clinical manifestations of leishmaniasis have been reported from 2001 to 2021, with an average of 52,645 cases per year of cutaneous leishmaniasis (ZCL) and mucosal leishmaniasis (ML) and an annual average of 2,488 cases of visceral leishmaniasis (VL) were recorded [9]. The data describe that the two first clinical manifestations are predominant in the Americas Region. They also indicate that there was an increasing trend in cases of cutaneous (ZCL) and mucosal leishmaniasis (ML), during the first years of the report (2001–2005). However, in the last five years, these cases tended to decrease. In the same period, for cases of visceral leishmaniasis (VL) were reported, like the other types of leishmaniasis between 2001 to 2010, there was a growing trend except in Colombia [9]. Yet, the trend during the last ten years is downward, with 1,799 cases in 2021, the lowest number of VL cases recorded in 21 years [9]. In the Americas, roughly 15 pathogenic types of *Leishmania* have been identified in humans, and nearly 54 non-vector species have been regarded as potentially involved in transmission (https://www.paho.org/en/topics/leishmaniasis). The parasite is transmitted through the bite of female sand flies from the Phlebotominae subfamily, colloquially known as *“*chiclero”, “asa branca”, “palomilla”, “sand fly palha”, or “torito”, among others. These insects are active at night and they inoculate the parasite through their bites on human (https://www.paho.org/en/topics/leishmaniasis). The control of leishmaniasis in the countries of the region relies on case detection and ambulatory treatment without comprehensive follow-up, converting this disease in neglected and thus being considered a public health problem, especially in Lain America [4,10]. Accordingly, the Pan American Health Organization (PAHO) approved the *Plan of Action on Entomology and Vector Control 2018–2023* in 2018, highlighting that diseases where the human-animal-environment interface is present, e.g., leishmaniasis, it is fundamental to the control and implement integrated approaches across different aspects and spheres of disease [9,11].

Currently, Colombia is one of the three countries in the America´s region with the highest number of *Leishmania* species affecting human populations: *Leishmania amazonensis*, *L*. *braziliensis*, *L*. *colombiensis*, *L*. *guyanensis*, *L*. *lainsoni*, *L*. *mexicana*, *L*. *naiffi*, *L*. *panamensis*, and *L*. *venezuelensis* [12]. 6,175 new cases were reported for cutaneous and mucosal leishmaniasis in 2021, which evidences that the dominant form of leishmaniasis in this country is cutaneous (ZCL) representing 99% of the cases [9,12]. The distribution of this disease in Colombia shows cases between 1,100 and 2,400 m.a.s.l., which are localities with the ideal conditions for vectors of *Leishmania*. Some phlebotomine species as *Bichromomyia*. *flaviscutellata*, *Psychodopygus panamensis*, *Lutzomyia gomezi*, *Lu*. *hartmanni*, *Nyssomyia trapidoi*, *Ny*. *umbratillis*, *Pintomyia spinicrassa*,and *Pi*. *longiflocosa* are found there [9,10,13]. Multiple mechanisms have been used to reduce the incidence of leishmaniasis in the country since the early 2000s [14], however the control on vector insects is an important element, and in some cases, it is the most efficient way to reduce the transmission of this type of tropical diseases [2,4,5]

*Pintomyia longiflocosa* is considered an important vector in the transmission of cutaneous leishmaniasis (ZCL) in Colombia [10,13,15]. This species is endemic to the sub-Andean region and even though it has been a species studied in different rural locations in the country, studies on more data about the distribution and density of the species are necessary to improve the control strategies for this disease [8,13]. This study focused on analyzing how the weather or climatological conditions are associated with the dynamic population of *Pi*. *longiflocosa* in a rural location in Colombia. Hence, the data examined can be employed in more efficient prediction, prevention, and control models to manage cutaneous leishmaniasis in Colombia. Therefore, this might contribute to PAHO’s Plan of Action on Entomology and Vector Control 2018–2023.

## Methods

### Study area

**Fig 1:**
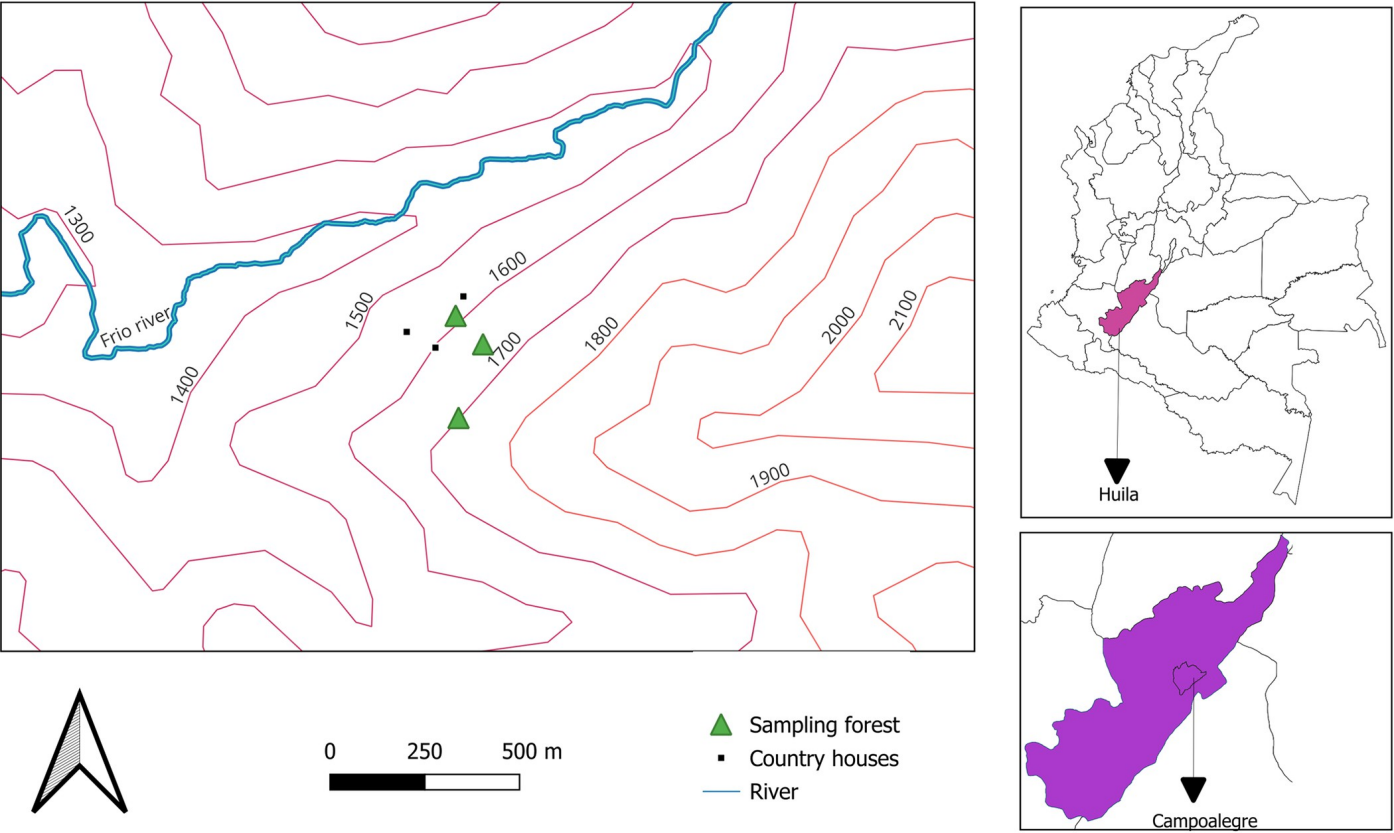
Location of the study and sample sites description. The map was obtained from the database of INSTITUTO GEOGRÁFICO AGUSTÍN CODAZZI–IGAC a free and open-source database (license: https://creativecommons.org/licenses/by/4.0/deed.es, CC BY 4.0) through the QGIS 3.32.

This study was conducted in the rural area of Campoalegre (Huila) in the rural settlement of Venecia (2°39´47´´N, 75°14´31´´W), located in the central sub-Andean region of Colombia at the elevation of 1,600 m.a.s.l. approximately (Fig 1). The average annual temperature and precipitation annual in this area are 20°C and 1,000 mm respectively, with bimodal rainfall periods [13]. The main economic activity of the rural human population is coffee production, which generates a landscape with high anthropogenic intervention and little remanence of the original forest. This area has been a point of observation regarding the dynamics of a population of the phebotomine sand fly *Pi*. *longiflocosa* in preview studies.

### Sand fly collection and taxonomic identification

The sand flies collected in this study were systematically sampled from three different locations, spanning the period from February 2020 to February 2021. Two of these locations were named as “indoor” and “peri-domestic areas” respectively. This assignment was according the presence of current or historical cases of leishmaniasis in households in the rural settlement of Venecia. On the other hand, the “outdoor” locations were selected according to these inclusion criteria: presence of trees with a diameter at breast height superior to 30 cm, trees with rough bark, a litter of 5 cm, and previous presence of phlebotomine sand flies and close to the forest [16,17]. The sampling in each location was done using a CDC miniature light traps that were placed for three consecutive nights for 12 hours, from 18:00 to 06:00 every two weeks by month. The traps distribution was: two inside of one house (bedroom; named “indoor”), two placed outside of the house (maximum 10 m away from the house; named “peri-domestic areas”), and six traps were placed 5 m away from the limit of the forest with a distance between traps higher to 15 m (named “outdoor”). After collect, the phlebotomine sand flies were removed from the traps, separated using external morphology, and preserved in 70% ethanol. The phlebotomine sand flies were cleared in the laboratory in 10% hot potassium hydroxide and transferred to saturated phenol [18]. Later, the sand flies were morphologically identified in Universidad de La Salle based on the classification adopted by Galaty in 2019 [19]. Finally, the sand flies were stored at *Insitituto Nacional de Salud* (INS).

### Data density analysis

The density of *Pi*. *longiflocosa* was calculated considering the number of females per trap per night (f/t/n) and the monthly average was calculated over each type of capture location (indoor, peri-domestic areas, and outdoor). For the analysis, it was not possible to normalize the data abundance of males (Shapiro-Wilk normality test, *P <* 0.0001). For this reason, the test used to compare between sexes was the Wilcoxon signed rank. On the other hand, the average density of females was normalized with the log transformation of the data (Shapiro-Will test, *W =* 0.979, *P =* 0.701). Depending on the normality of the dataset, the comparison between localities and months was made using the non-parametric Kruskal-Wallis rank sum test or the parametric ANOVA test. Pairwise differences in abundance were compared using the post-hoc Tukey-Kramer HSD test with a significance level of 5%. Statistical analysis was carried out using R [20] and RStudio [21].

### Climatic variables measures and analysis

The data of temperature, relative humidity, and precipitation *ex situ* were obtained from the annual registers of Algeciras climatic station COD 21105030 of the *Instituto de Hidrología*, *Meteorología y Estudios Ambientales* (IDEAM), this station is placed 7.2 km away from the sample locations. To evaluate the relationship between these climatic variables, Pearson correlation was done. Comparisons and analyses with the environmental variables were done with the female density from the outdoor location.

## Results

### Density analysis

Over the study period, a total of 750 trap nights were used and 84,848 sand flies of Phlebotomini tribu were collected in all sample locations. Among the sand flies captured, the majority were *Pi*. *longiflocosa* (*n =* 83,885), followed by *Lutzomyia* (Helcocyrtomyia) *sp*. (*n =* 812), *Pi*. *nuneztovari* (*n =* 127) and *Micropygomyia trinidadensis* (*n =* 24).

**Fig 2:**
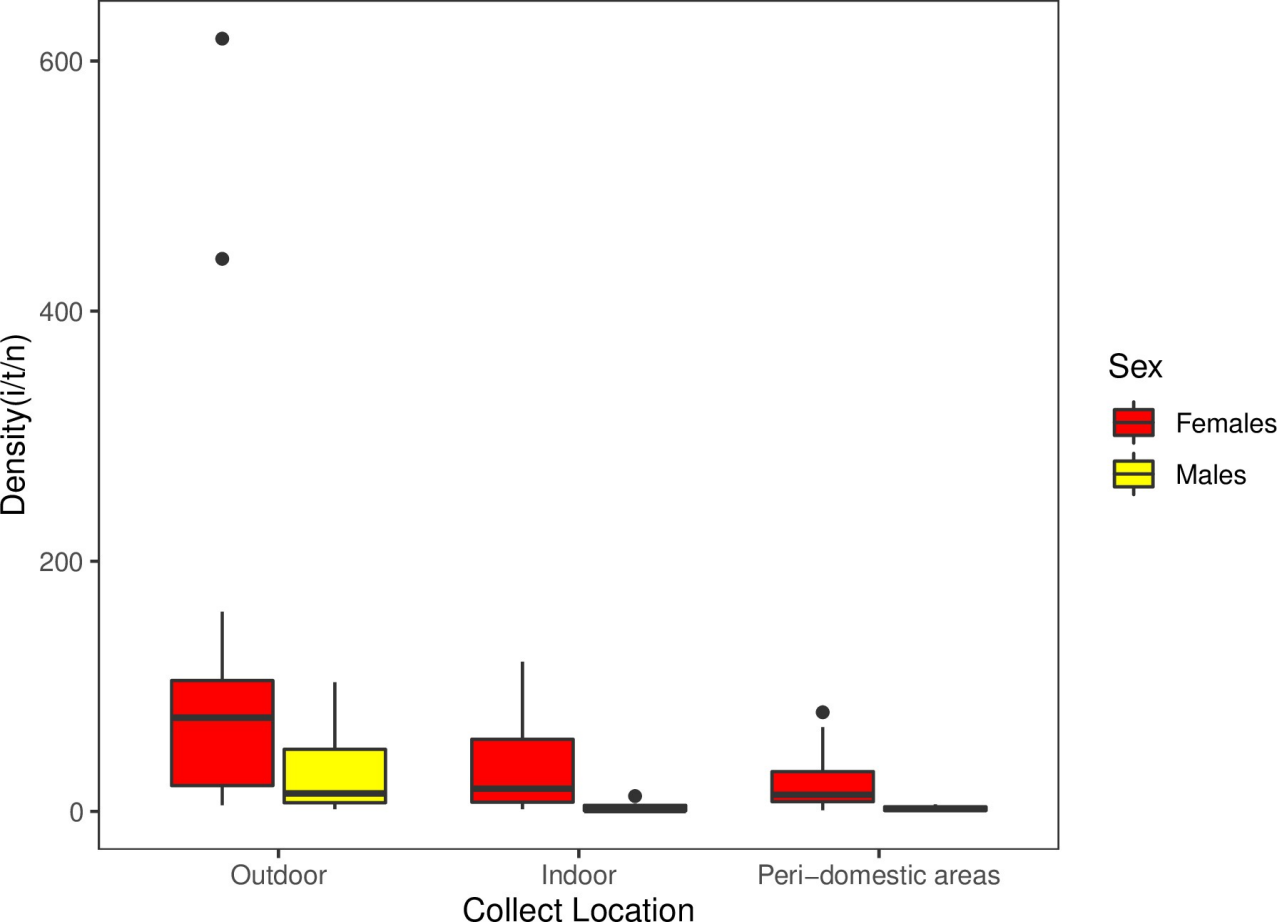
Density per sex of *Pi*. *longiflocosa* in the collection locations of this study. Points outside of boxplots represent outliers’ data.

*Pintomyia longiflocosa* varied considerably among sex (Wilcoxon signed-rank, *V =* 780, *P<*0.0001; Table 1), The females were the sex with high abundance in all traps and all study locations, contributing significantly to the abundance of *Pi*. *longiflocosa* (Fig 2). The density of this species varied too when the study locations were analyzed (Kruskal-Wallis test, *X*^*2*^ *=* 6.398, *P =* 0.04, *df =* 2; Table 1), showing that high density is found in the outdoor location with a significative difference when compared with the indoor collect location (TukeyHSD, *P =* 0.048; Fig 2). In turn, the two localities associated with houses (indoor and peri-domestic areas) indicated similar density values (TukeyHSD, *P =* 0.944; Fig 2).

**Table 1:** Density of *Pi*. *longiflocosa* (average of density by trap by month).

		Collect Location								
		Indoor			Peri-domestic areas			Outdoor		
		♀	♂	Total	♀	♂	Total	♀	♂	Total
2020	February	82.83	5.83	88.67	31.83	0.66	32.5	89.94	14.39	104.33
	March	6.91	0.5	7.41	7.66	0.66	8.33	21.47	6.91	28.39
	April	1.75	0.16	1.91	1	0.33	1.33	4.88	1.77	6.66
	May	10	1.08	11.08	10.17	1.33	11.5	16.31	4.33	20.64
	June	42	4.83	46.83	17.67	5.66	23.33	78.53	46.03	124.6
	July	99.58	6.25	105.8	67.33	4.25	71.58	441.78	103.2	544.9
	August	57.67	1.08	58.75	79.25	3.25	82.5	617.8	52.4	670.3
	September	18.17	0.16	18.33	13.25	0.33	13.58	104.6	8.41	113
	October	8.08	0.16	8.25	5.16	0.5	5.66	59.86	18.81	78.67
	November	6.75	0.83	7.58	9.75	2.08	11.83	20.64	6.61	27.25
	December	7.41	2	9.41	7.75	4.75	12.5	18.69	13.69	32.39
2021	January	45	2.66	47.67	25.25	3.41	28.67	74.92	67.44	142.4
	February	119.6	12.25	131.8	48.75	3.66	52.42	159.6	49.64	209.2

**Fig 3:**
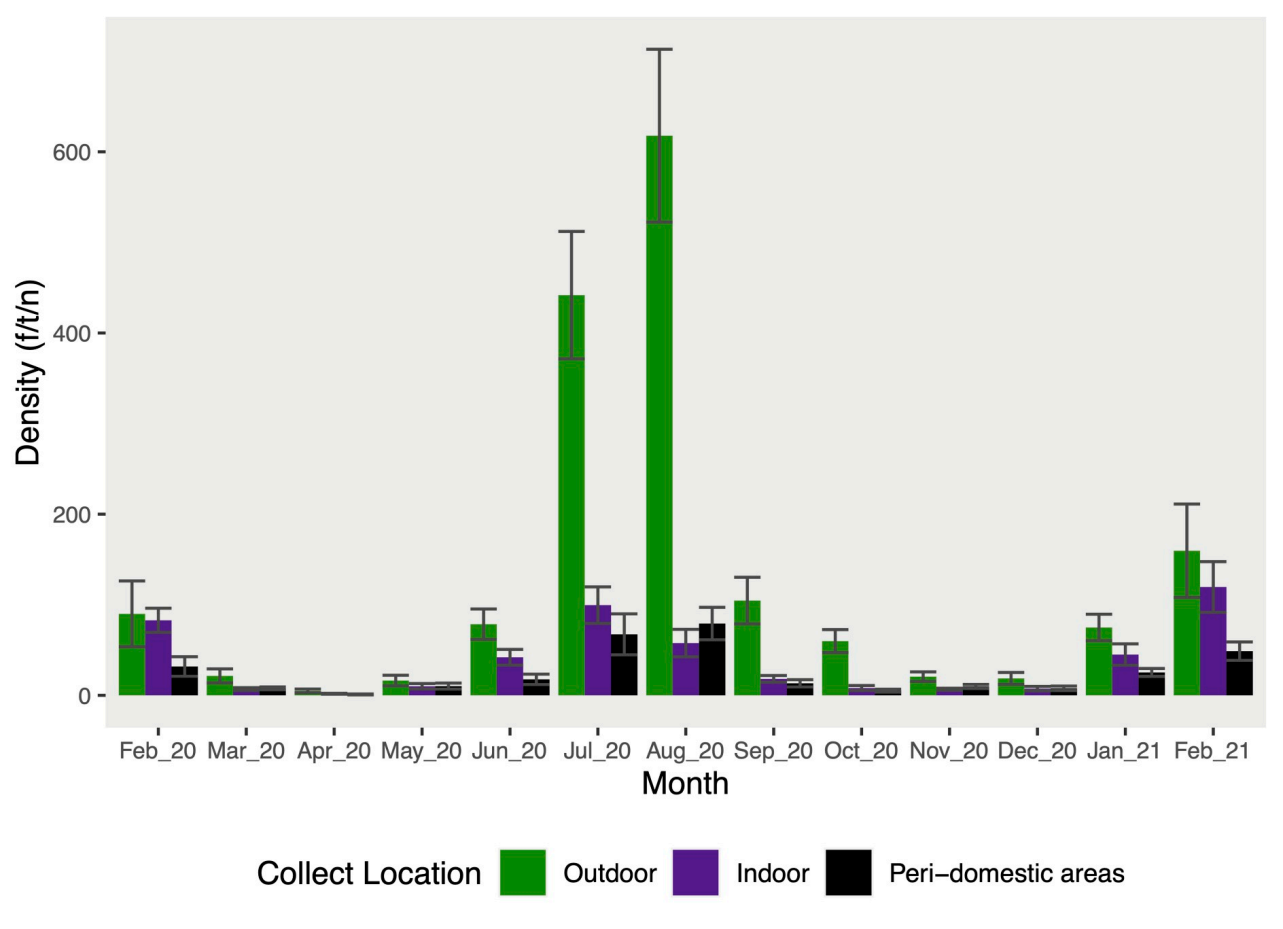
Average and standard error of *Pi*. *longiflocosa* female density in the months and different collection locations of this study. Density is described as the number of females/traps per night.

When the density was observed during the time of collect, significative differences were detected between months (Kruskal-Wallis test, *X*^*2*^ *=* 28,297, *P =* 0.005, *df =* 12; Table 1). The analysis revealed a high and significant density of *Pi*. *longiflocosa* during two periods in the year of study (ANOVA, *F =* 6,845, *P<*0.0001, *df =* 12). The first period took place in January and February while the second one was from June to October, with the months of July and August accounted for the highest density (TukeyHSD, *P<*0.05; Fig 3). In contrast, April showed the lowest significative density values during the study period in the outdoor (TukeyHSD, *P<*0.05; Fig 3). The tendency of density data is similar in all collection locations.

### Climatic Variables Analysis

The climatic variables data analyzed in this study showed normality (Shapiro-Will, *P>*0.05). The correlation analysis described a significative relation between humidity and both temperature and precipitation (Pearson´s correlation, *df =* 11, *P<*0.01; Table 2). Still, the analysis revealed independence between temperature and precipitation (Pearson´s correlation, *df =* 11, *P =* 0.665; Table 2).

**Table 2:** Pearson Correlation Results between Climatic Variables and Density of *Pi*. *longiflocosa*.

	Humidity	Temperature	Precipitation	Density *Pi*. *longiflocosa* (f/t/m)
Humidity	-	-0.715**	0.625**	-0.332
Temperature		-	-0.132	-0.131
Precipitation			-	-0.251

The asterisks represent significative Pearson correlation values (** *P <* 0.01)

**Fig 4:**
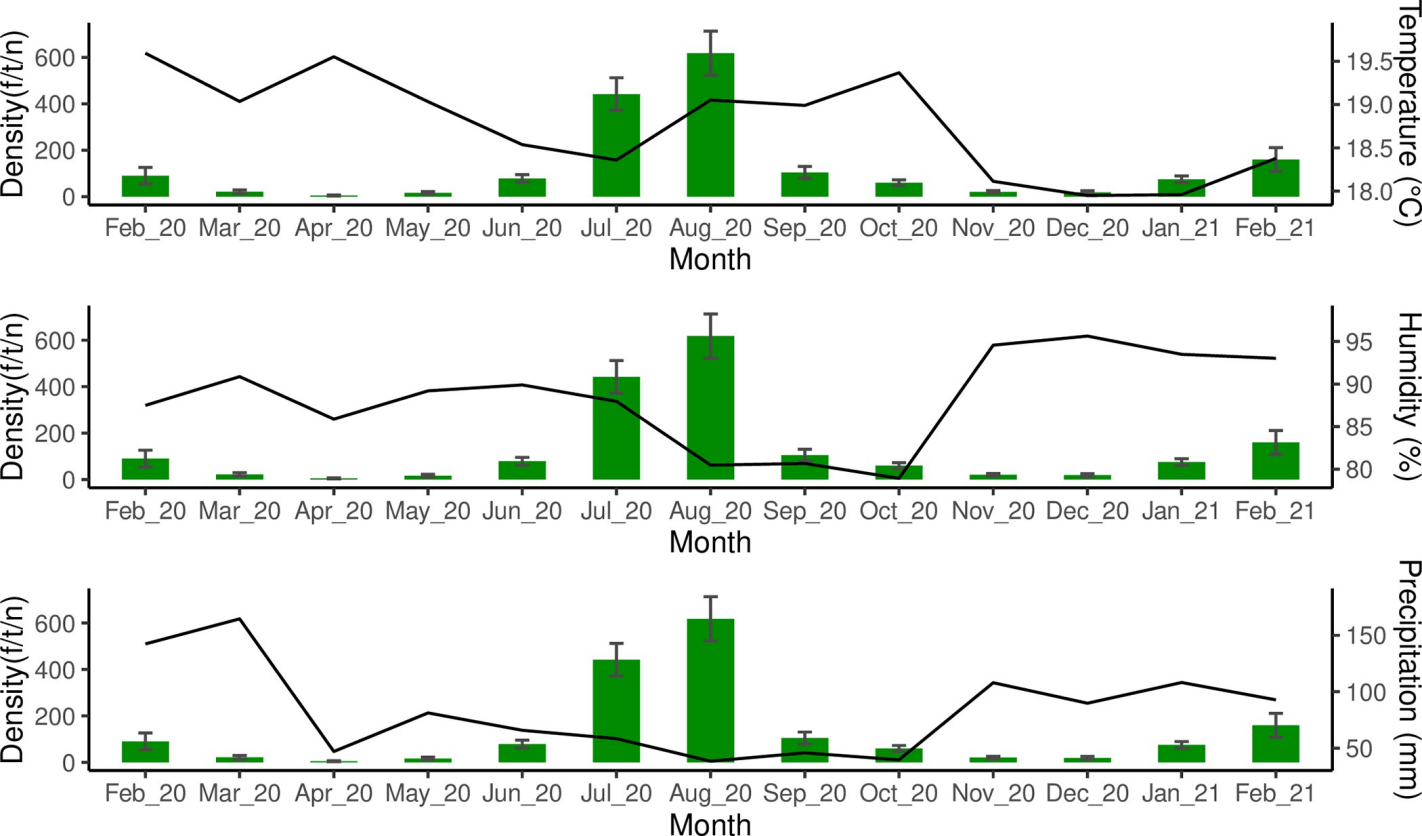
Relations between density of *Pi*. *longiflocosa* and the climatic variables analyzed in this study.

Additionally, although the density of *Pi*. *longiflocosa* was not correlated significantly with any climatic variable (Pearson´s correlation, *df =* 11, *P>*0.05; Table 2), the correlation results highlighted some relations. The range of humidity during the period of this study was between 78.91% and 95.62%, with the lowest values from August to October. This partially overlaps with one of the months with the highest density of sand flies, confirming the negative correlation obtained between these variables (Table 2; Fig 4). The temperature did not show obvious relations with density and it was constant during the year of study with a close variation of 1.6°C (Range _(max–min)_ *=* 19.59–17.94; Fig 4). Finally, precipitation had a range between 34.8mm and 164.6mm and described a negative association with density although not significative (Table 2), where the period with the highest sand flies’ density values (June and September) had the lowest precipitation data and in the same way, months with high precipitations showed low-density values (Fig 4).

## Discussion

Studies of spatial and temporal distributions of vectors associated with diseases like cutaneous leishmaniasis offer powerful tools for identifying risk zones and periods where interactions between hosts, vectors, and parasites are more frequent [7,8]. Our results not only contribute to understanding the population dynamics of *Pi*. *longiflocosa* but also to provide a baseline to improve management and control strategies on insect vectors of this tropical disease in Colombia.

As for the spatial level, the results confirm that *Pi*. *Longiflocosa* females contribute significantly to the density of the species in all locations in this study (Fig 2). This result is coherent with other studies’ findings, where the collect traps favored female capture and support that behavior is different between sexes [13,15]. This hypothesis is also supported when the life cycle has been studied in the laboratory or in semi-wild conditions where the offspring of wild females have a sex ratio of 50:50 [13,17]. The outdoor was the only location where the density of males was high, which makes this location the main place to capture females and males in Campoalegre (Huila) (Fig 2). The density values of the indoor showed that this place is the second in order of abundance of sand flies (Fig 2). These results are coherent with the observations of this behavior in other species of *Pintomyia* and *Lutzomyia* that transmit leishmaniasis in the forest or human habitations near the forest (locations named “outdoor”) [2,22].

Concerning the temporal level, after monitoring the density of *Pi*. *longiflocosa* for 13 months, the precipitation is the most associated variable with the density of these sand flies, revealing that those months with the lowest precipitations have the highest density of *Pi*. *longiflocosa*. This negative association was reported in another study where ecological variables linked to forest microhabitats (“outdoors”)were described and confirmed that regional levels of temperature and precipitation are determinants in the abundance of this species [23,24]. In this study, although the values of temperature were in the rank proposed for the same study (18°C–19.9°C), the association was not obvious.

The dynamics of insect populations and their behaviors are affected by climate, and in the same way, they affect the transmission cycles of pathogens vectors of these arthropods [25–29]. For this reason, elucidating the environmental conditions and habitat attributes fostering elevated sand fly population density is crucial. This initiative not only advances our comprehension of the Dynamics governing sand fly abundance but also facilitates the anticipation of months characterized by heightended susceptibility to cutaneous leishmaniasis, there by elucidating its potential repercussions on human health [23]. For the locality of this study, Campoalegre (Huila), our results describe that months of February, June, July, August, September, and October as well as the human population close to the forest, “outdoor”, are the factors highest risk to acquire the disease. This is because of the relation between the high densities of sand flies and the presence and abundance of leishmaniasis cases [2,4,7]. Moreover, this study corroborates that these months exhibit the lowest precipitation levels in this region. Thereby these observations could prompt further investigations in other localities to validate that variations in this environmental variable could potentially enable the prediction of *Pintomyia longiflocosa* density, thus helping to organize mitigation and priority actions. Given the absence of vaccines for leishmaniasis, studies like this serves as a valuable tool or strategy to enhance surveillance and control programs. Thereby, it boosts the idea that a good understanding of vector habitats is essential to formulate operational strategies [30–34].

## Conclusion

In summary, our study on the spatial and temporal dynamics of *Pi*. *longiflocosa*, a sand fly species linked to cutaneous leishmaniasis in Colombia, reveals significant insights. The research emphasizes the importance of understanding environmental conditions influencing sand fly density. We found that *Pi*. *longiflocosa* females play a crucial role in overall population density across various locations, with outdoor environments being the primary capture site. Temporally, lower precipitation levels correlate with higher sand fly density, with February, June, July, August, September, and October identified as high-risk months in Campoalegre (Huila). These findings underscore the impact of climate on sand fly behaviors and population dynamics. The observed associations provide a basis to explore more in detail the relationship between low precipitation and high sandfly density and for predictive modeling and targeted interventions in regions at risk for leishmaniasis, contributing valuable information for surveillance and control programs in the absence of vaccines for this disease.

## Supporting information

S1 File Data collection. (PDF)

S2 File Environmental variables. (PDF)
