# Supplementary material for: Spatio—Temporal distribution of a vector of cutaneous leishmaniasis: Pintomyia longiflocosa, in a population from the Colombian Andean Mountains
Source: PLoS Negl Trop Dis. 2024 Jun 17;18(6):e0012237. doi: 10.1371/journal.pntd.0012237 (PMC11213335; doi:10.1371/journal.pntd.0012237)
Supplement: S1 File — (PDF) [file pntd.0012237.s001.pdf]

Month	Capture_Location	Males	Females	Total
Feb_20	forest	4	120	124
Feb_20	forest	4	123	127
Feb_20	forest	8	359	367
Feb_20	forest	0	78	78
Feb_20	forest	0	0	0
Feb_20	forest	3	78	81
Feb_20	intradomicilio	3	75	78
Feb_20	intradomicilio	0	70	70
Feb_20	peridomicilio	1	43	44
Feb_20	peridomicilio	0	0	0
Feb_20	forest	23	96	119
Feb_20	forest	200	598	798
Feb_20	forest	3	105	108
Feb_20	forest	8	28	36
Feb_20	forest	5	16	21
Feb_20	forest	0	0	0
Feb_20	intradomicilio	2	61	63
Feb_20	intradomicilio	5	110	115
Feb_20	peridomicilio	0	33	33
Feb_20	peridomicilio	0	74	74
Feb_20	forest	0	1	1
Feb_20	forest	1	14	15
Feb_20	forest	0	0	0
Feb_20	forest	0	3	3
Feb_20	forest	0	0	0
Feb_20	forest	0	0	0
Feb_20	intradomicilio	24	134	158
Feb_20	intradomicilio	1	47	48
Feb_20	peridomicilio	1	8	9
Feb_20	peridomicilio	2	33	35
Mar_20	forest	2	36	38
Mar_20	forest	112	254	366
Mar_20	forest	0	16	16
Mar_20	forest	0	9	9
Mar_20	forest	4	19	23
Mar_20	forest	2	2	4
Mar_20	intradomicilio	0	6	6
Mar_20	intradomicilio	0	19	19
Mar_20	peridomicilio	2	10	12
Mar_20	peridomicilio	0	4	4
Mar_20	forest	2	16	18
Mar_20	forest	30	66	96

Mar_20	forest	0	2	2
Mar_20	forest	1	21	22
Mar_20	forest	0	4	4
Mar_20	forest	0	0	0
Mar_20	intradomicilio	1	6	7
Mar_20	intradomicilio	1	0	1
Mar_20	peridomicilio	0	4	4
Mar_20	peridomicilio	0	6	6
Mar_20	forest	0	17	17
Mar_20	forest	0	0	0
Mar_20	forest	0	2	2
Mar_20	forest	2	9	11
Mar_20	forest	0	3	3
Mar_20	forest	0	1	1
Mar_20	intradomicilio	0	13	13
Mar_20	intradomicilio	2	11	13
Mar_20	peridomicilio	1	9	10
Mar_20	peridomicilio	0	14	14
Mar_20	forest	20	40	60
Mar_20	forest	59	126	185
Mar_20	forest	1	5	6
Mar_20	forest	0	22	22
Mar_20	forest	3	20	23
Mar_20	forest	0	3	3
Mar_20	intradomicilio	1	8	9
Mar_20	intradomicilio	0	9	9
Mar_20	peridomicilio	2	20	22
Mar_20	peridomicilio	3	10	13
Mar_20	forest	0	21	21
Mar_20	forest	10	31	41
Mar_20	forest	0	3	3
Mar_20	forest	1	12	13
Mar_20	forest	0	1	1
Mar_20	forest	0	2	2
Mar_20	intradomicilio	0	1	1
Mar_20	intradomicilio	0	5	5
Mar_20	peridomicilio	0	8	8
Mar_20	peridomicilio	0	5	5
Mar_20	forest	0	0	0
Mar_20	forest	0	3	3
Mar_20	forest	0	0	0
Mar_20	forest	0	0	0
Mar_20	forest	0	5	5

Mar_20	forest	0	2	2
Mar_20	intradomicilio	0	2	2
Mar_20	intradomicilio	1	3	4
Mar_20	peridomicilio	0	2	2
Mar_20	peridomicilio	0	0	0
Apr_20	forest	0	3	3
Apr_20	forest	31	61	92
Apr_20	forest	0	11	11
Apr_20	forest	0	4	4
Apr_20	forest	0	1	1
Apr_20	forest	1	1	2
Apr_20	intradomicilio	0	5	5
Apr_20	intradomicilio	1	4	5
Apr_20	peridomicilio	1	4	5
Apr_20	peridomicilio	1	5	6
Apr_20	forest	0	1	1
Apr_20	forest	9	8	17
Apr_20	forest	1	0	1
Apr_20	forest	1	0	1
Apr_20	forest	0	0	0
Apr_20	forest	0	1	1
Apr_20	intradomicilio	0	3	3
Apr_20	intradomicilio	0	1	1
Apr_20	peridomicilio	0	2	2
Apr_20	peridomicilio	0	0	0
Apr_20	forest	0	0	0
Apr_20	forest	0	2	2
Apr_20	forest	0	1	1
Apr_20	forest	0	0	0
Apr_20	forest	0	0	0
Apr_20	forest	0	0	0
Apr_20	intradomicilio	0	0	0
Apr_20	intradomicilio	1	2	3
Apr_20	peridomicilio	1	0	1
Apr_20	peridomicilio	0	0	0
Apr_20	forest	0	0	0
Apr_20	forest	15	43	58
Apr_20	forest	1	2	3
Apr_20	forest	0	2	2
Apr_20	forest	0	0	0
Apr_20	forest	0	0	0
Apr_20	intradomicilio	0	2	2
Apr_20	intradomicilio	0	2	2

Apr_20	peridomicilio	1	1	2
Apr_20	peridomicilio	0	0	0
Apr_20	forest	0	5	5
Apr_20	forest	2	9	11
Apr_20	forest	0	0	0
Apr_20	forest	0	14	14
Apr_20	forest	0	0	0
Apr_20	forest	0	0	0
Apr_20	intradomicilio	0	0	0
Apr_20	intradomicilio	0	0	0
Apr_20	peridomicilio	0	0	0
Apr_20	peridomicilio	0	0	0
Apr_20	forest	0	0	0
Apr_20	forest	3	4	7
Apr_20	forest	0	2	2
Apr_20	forest	0	0	0
Apr_20	forest	0	0	0
Apr_20	forest	0	1	1
Apr_20	intradomicilio	0	0	0
Apr_20	intradomicilio	0	2	2
Apr_20	peridomicilio	0	0	0
Apr_20	peridomicilio	0	0	0
May_20	forest	0	0	0
May_20	forest	4	4	8
May_20	forest	0	0	0
May_20	forest	0	0	0
May_20	forest	0	0	0
May_20	forest	0	1	1
May_20	intradomicilio	1	4	5
May_20	intradomicilio	2	3	5
May_20	peridomicilio	0	2	2
May_20	peridomicilio	1	3	4
May_20	forest	0	0	0
May_20	forest	0	1	1
May_20	forest	0	0	0
May_20	forest	0	0	0
May_20	forest	0	0	0
May_20	forest	0	0	0
May_20	intradomicilio	0	0	0
May_20	intradomicilio	1	0	1
May_20	peridomicilio	0	0	0
May_20	peridomicilio	0	0	0
May_20	forest	1	0	1

May_20	forest	6	93	99
May_20	forest	0	0	0
May_20	forest	0	2	2
May_20	forest	0	0	0
May_20	forest	0	0	0
May_20	intradomicilio	3	37	40
May_20	intradomicilio	2	12	14
May_20	peridomicilio	2	28	30
May_20	peridomicilio	0	0	0
May_20	forest	3	23	26
May_20	forest	29	150	179
May_20	forest	2	13	15
May_20	forest	1	11	12
May_20	forest	0	1	1
May_20	forest	1	1	2
May_20	intradomicilio	1	12	13
May_20	intradomicilio	1	11	12
May_20	peridomicilio	2	15	17
May_20	peridomicilio	1	19	20
May_20	forest	0	0	0
May_20	forest	12	96	108
May_20	forest	0	4	4
May_20	forest	0	10	10
May_20	forest	0	0	0
May_20	forest	0	0	0
May_20	intradomicilio	0	20	20
May_20	intradomicilio	0	12	12
May_20	peridomicilio	0	0	0
May_20	peridomicilio	3	30	33
May_20	forest	22	66	88
May_20	forest	64	84	148
May_20	forest	7	12	19
May_20	forest	4	15	19
May_20	forest	0	0	0
May_20	forest	0	0	0
May_20	intradomicilio	1	8	9
May_20	intradomicilio	1	1	2
May_20	peridomicilio	1	4	5
May_20	peridomicilio	6	21	27
Jun_20	forest	9	17	26
Jun_20	forest	44	43	87
Jun_20	forest	0	2	2
Jun_20	forest	0	8	8

Jun_20	forest	58	56	114
Jun_20	forest	53	45	98
Jun_20	intradomicilio	3	11	14
Jun_20	intradomicilio	1	5	6
Jun_20	peridomicilio	0	5	5
Jun_20	peridomicilio	3	7	10
Jun_20	forest	38	53	91
Jun_20	forest	153	196	349
Jun_20	forest	22	16	38
Jun_20	forest	7	18	25
Jun_20	forest	133	129	262
Jun_20	forest	18	21	39
Jun_20	intradomicilio	6	31	37
Jun_20	intradomicilio	1	61	62
Jun_20	peridomicilio	17	39	56
Jun_20	peridomicilio	13	26	39
Jun_20	forest	37	110	147
Jun_20	forest	0	3	3
Jun_20	forest	2	4	6
Jun_20	forest	199	183	382
Jun_20	forest	0	0	0
Jun_20	forest	144	160	304
Jun_20	intradomicilio	0	48	48
Jun_20	intradomicilio	3	43	46
Jun_20	peridomicilio	10	36	46
Jun_20	peridomicilio	1	10	11
Jun_20	forest	55	49	104
Jun_20	forest	27	101	128
Jun_20	forest	6	30	36
Jun_20	forest	3	7	10
Jun_20	forest	2	71	73
Jun_20	forest	94	139	233
Jun_20	intradomicilio	2	24	26
Jun_20	intradomicilio	2	28	30
Jun_20	peridomicilio	1	2	3
Jun_20	peridomicilio	17	11	28
Jun_20	forest	16	26	42
Jun_20	forest	27	338	365
Jun_20	forest	0	7	7
Jun_20	forest	0	1	1
Jun_20	forest	112	257	369
Jun_20	forest	286	438	724
Jun_20	intradomicilio	9	98	107

Jun_20	intradomicilio	31	49	80
Jun_20	peridomicilio	1	65	66
Jun_20	peridomicilio	0	5	5
Jun_20	forest	8	17	25
Jun_20	forest	74	66	140
Jun_20	forest	1	3	4
Jun_20	forest	3	19	22
Jun_20	forest	3	149	152
Jun_20	forest	23	45	68
Jun_20	intradomicilio	0	13	13
Jun_20	intradomicilio	0	93	93
Jun_20	peridomicilio	4	2	6
Jun_20	peridomicilio	1	4	5
Jul_20	forest	4	365	369
Jul_20	forest	405	791	1196
Jul_20	forest	1	90	91
Jul_20	forest	2	105	107
Jul_20	forest	115	418	533
Jul_20	forest	526	738	1264
Jul_20	intradomicilio	4	223	227
Jul_20	intradomicilio	0	97	97
Jul_20	peridomicilio	2	102	104
Jul_20	peridomicilio	3	274	277
Jul_20	forest	583	1432	2015
Jul_20	forest	58	1221	1279
Jul_20	forest	0	101	101
Jul_20	forest	2	16	18
Jul_20	forest	165	391	556
Jul_20	forest	42	505	547
Jul_20	intradomicilio	1	116	117
Jul_20	intradomicilio	0	86	86
Jul_20	peridomicilio	0	43	43
Jul_20	peridomicilio	4	104	108
Jul_20	forest	2	29	31
Jul_20	forest	28	167	195
Jul_20	forest	0	7	7
Jul_20	forest	0	18	18
Jul_20	forest	72	197	269
Jul_20	forest	105	641	746
Jul_20	intradomicilio	1	18	19
Jul_20	intradomicilio	32	70	102
Jul_20	peridomicilio	1	13	14
Jul_20	peridomicilio	0	8	8

Jul_20	forest	13	874	887
Jul_20	forest	136	1367	1503
Jul_20	forest	8	188	196
Jul_20	forest	16	196	212
Jul_20	forest	119	924	1043
Jul_20	forest	189	1136	1325
Jul_20	intradomicilio	3	172	175
Jul_20	intradomicilio	7	134	141
Jul_20	peridomicilio	7	33	40
Jul_20	peridomicilio	23	134	157
Jul_20	forest	14	280	294
Jul_20	forest	25	127	152
Jul_20	forest	12	25	37
Jul_20	forest	0	24	24
Jul_20	forest	66	569	635
Jul_20	forest	102	271	373
Jul_20	intradomicilio	1	44	45
Jul_20	intradomicilio	1	32	33
Jul_20	peridomicilio	1	9	10
Jul_20	peridomicilio	1	11	12
Jul_20	forest	117	332	449
Jul_20	forest	179	838	1017
Jul_20	forest	1	46	47
Jul_20	forest	8	53	61
Jul_20	forest	442	984	1426
Jul_20	forest	157	438	595
Jul_20	intradomicilio	1	11	12
Jul_20	intradomicilio	24	192	216
Jul_20	peridomicilio	8	64	72
Jul_20	peridomicilio	1	13	14
Aug_20	forest	11	1050	1061
Aug_20	forest	174	2109	2283
Aug_20	forest	1	253	254
Aug_20	forest	0	186	186
Aug_20	forest	146	1907	2053
Aug_20	forest	223	1887	2110
Aug_20	intradomicilio	1	74	75
Aug_20	intradomicilio	1	1	2
Aug_20	peridomicilio	6	168	174
Aug_20	peridomicilio	1	82	83
Aug_20	forest	3	204	207
Aug_20	forest	97	976	1073
Aug_20	forest	119	523	642

Aug_20	forest	51	413	464
Aug_20	forest	102	581	683
Aug_20	forest	169	977	1146
Aug_20	intradomicilio	0	35	35
Aug_20	intradomicilio	1	142	143
Aug_20	peridomicilio	24	195	219
Aug_20	peridomicilio	2	44	46
Aug_20	forest	0	1	1
Aug_20	forest	4	149	153
Aug_20	forest	0	1	1
Aug_20	forest	0	4	4
Aug_20	forest	0	101	101
Aug_20	forest	13	190	203
Aug_20	intradomicilio	1	5	6
Aug_20	intradomicilio	1	20	21
Aug_20	peridomicilio	2	15	17
Aug_20	peridomicilio	0	8	8
Aug_20	forest	7	606	613
Aug_20	forest	62	1739	1801
Aug_20	forest	7	174	181
Aug_20	forest	6	195	201
Aug_20	forest	125	468	593
Aug_20	forest	11	247	258
Aug_20	intradomicilio	0	32	32
Aug_20	intradomicilio	4	80	84
Aug_20	peridomicilio	0	28	28
Aug_20	peridomicilio	2	112	114
Aug_20	forest	9	787	796
Aug_20	forest	49	827	876
Aug_20	forest	9	183	192
Aug_20	forest	48	639	687
Aug_20	forest	10	399	409
Aug_20	forest	105	1515	1620
Aug_20	intradomicilio	0	59	59
Aug_20	intradomicilio	0	26	26
Aug_20	peridomicilio	0	48	48
Aug_20	peridomicilio	1	57	58
Aug_20	forest	14	679	693
Aug_20	forest	138	681	819
Aug_20	forest	56	266	322
Aug_20	forest	14	301	315
Aug_20	forest	81	593	674
Aug_20	forest	25	430	455

Aug_20	intradomicilio	3	171	174
Aug_20	intradomicilio	1	47	48
Aug_20	peridomicilio	1	146	147
Aug_20	peridomicilio	0	48	48
Sep_20	forest	4	202	206
Sep_20	forest	88	599	687
Sep_20	forest	1	19	20
Sep_20	forest	1	36	37
Sep_20	forest	5	391	396
Sep_20	forest	39	392	431
Sep_20	intradomicilio	0	28	28
Sep_20	intradomicilio	0	44	44
Sep_20	peridomicilio	1	24	25
Sep_20	peridomicilio	0	5	5
Sep_20	forest	0	68	68
Sep_20	forest	16	370	386
Sep_20	forest	2	94	96
Sep_20	forest	6	57	63
Sep_20	forest	17	288	305
Sep_20	forest	29	399	428
Sep_20	intradomicilio	0	20	20
Sep_20	intradomicilio	0	24	24
Sep_20	peridomicilio	2	50	52
Sep_20	peridomicilio	0	24	24
Sep_20	forest	0	2	2
Sep_20	forest	0	23	23
Sep_20	forest	0	0	0
Sep_20	forest	0	0	0
Sep_20	forest	0	25	25
Sep_20	forest	1	67	68
Sep_20	intradomicilio	0	3	3
Sep_20	intradomicilio	0	25	25
Sep_20	peridomicilio	0	16	16
Sep_20	peridomicilio	0	12	12
Sep_20	forest	0	1	1
Sep_20	forest	6	81	87
Sep_20	forest	0	0	0
Sep_20	forest	0	0	0
Sep_20	forest	0	24	24
Sep_20	forest	9	52	61
Sep_20	intradomicilio	0	16	16
Sep_20	intradomicilio	2	9	11
Sep_20	peridomicilio	0	3	3

Sep_20	peridomicilio	0	5	5
Sep_20	forest	2	16	18
Sep_20	forest	62	90	152
Sep_20	forest	0	0	0
Sep_20	forest	1	5	6
Sep_20	forest	8	211	219
Sep_20	forest	6	237	243
Sep_20	intradomicilio	0	28	28
Sep_20	intradomicilio	0	18	18
Sep_20	peridomicilio	1	2	3
Sep_20	peridomicilio	0	8	8
Sep_20	forest	0	1	1
Sep_20	forest	0	3	3
Sep_20	forest	0	0	0
Sep_20	forest	0	0	0
Sep_20	forest	0	11	11
Sep_20	forest	0	1	1
Sep_20	intradomicilio	0	3	3
Sep_20	intradomicilio	0	0	0
Sep_20	peridomicilio	0	2	2
Sep_20	peridomicilio	0	8	8
Oct_20	forest	1	15	16
Oct_20	forest	10	78	88
Oct_20	forest	0	2	2
Oct_20	forest	0	1	1
Oct_20	forest	43	114	157
Oct_20	forest	8	110	118
Oct_20	intradomicilio	0	6	6
Oct_20	intradomicilio	0	7	7
Oct_20	peridomicilio	0	12	12
Oct_20	peridomicilio	0	0	0
Oct_20	forest	2	65	67
Oct_20	forest	49	157	206
Oct_20	forest	0	0	0
Oct_20	forest	0	1	1
Oct_20	forest	51	138	189
Oct_20	forest	69	204	273
Oct_20	intradomicilio	0	2	2
Oct_20	intradomicilio	0	8	8
Oct_20	peridomicilio	0	2	2
Oct_20	peridomicilio	0	7	7
Oct_20	forest	26	117	143
Oct_20	forest	206	261	467

Oct_20	forest	8	24	32
Oct_20	forest	7	17	24
Oct_20	forest	29	141	170
Oct_20	forest	91	233	324
Oct_20	intradomicilio	1	4	5
Oct_20	intradomicilio	0	12	12
Oct_20	peridomicilio	2	18	20
Oct_20	peridomicilio	2	5	7
Oct_20	forest	0	0	0
Oct_20	forest	3	22	25
Oct_20	forest	0	0	0
Oct_20	forest	0	0	0
Oct_20	forest	9	15	24
Oct_20	forest	30	31	61
Oct_20	intradomicilio	0	2	2
Oct_20	intradomicilio	0	0	0
Oct_20	peridomicilio	0	2	2
Oct_20	peridomicilio	0	3	3
Oct_20	forest	6	120	126
Oct_20	forest	9	185	194
Oct_20	forest	1	1	2
Oct_20	forest	0	6	6
Oct_20	forest	9	34	43
Oct_20	forest	9	55	64
Oct_20	intradomicilio	0	14	14
Oct_20	intradomicilio	1	35	36
Oct_20	peridomicilio	2	9	11
Oct_20	peridomicilio	0	4	4
Oct_20	forest	1	3	4
Oct_20	forest	0	3	3
Oct_20	forest	0	0	0
Oct_20	forest	0	0	0
Oct_20	forest	0	1	1
Oct_20	forest	0	1	1
Oct_20	intradomicilio	0	0	0
Oct_20	intradomicilio	0	7	7
Oct_20	peridomicilio	0	0	0
Oct_20	peridomicilio	0	0	0
Nov_20	forest	8	52	60
Nov_20	forest	6	42	48
Nov_20	forest	0	8	8
Nov_20	forest	0	9	9
Nov_20	forest	6	25	31

Nov_20	forest	15	145	160
Nov_20	intradomicilio	2	11	13
Nov_20	intradomicilio	1	11	12
Nov_20	peridomicilio	1	11	12
Nov_20	peridomicilio	1	25	26
Nov_20	forest	1	2	3
Nov_20	forest	20	5	25
Nov_20	forest	0	0	0
Nov_20	forest	0	0	0
Nov_20	forest	15	60	75
Nov_20	forest	5	27	32
Nov_20	intradomicilio	0	6	6
Nov_20	intradomicilio	2	3	5
Nov_20	peridomicilio	0	3	3
Nov_20	peridomicilio	2	8	10
Nov_20	forest	0	0	0
Nov_20	forest	1	4	5
Nov_20	forest	0	2	2
Nov_20	forest	1	2	3
Nov_20	forest	8	21	29
Nov_20	forest	9	13	22
Nov_20	intradomicilio	0	0	0
Nov_20	intradomicilio	1	7	8
Nov_20	peridomicilio	8	12	20
Nov_20	peridomicilio	0	3	3
Nov_20	forest	0	0	0
Nov_20	forest	0	0	0
Nov_20	forest	0	1	1
Nov_20	forest	0	0	0
Nov_20	forest	8	9	17
Nov_20	forest	9	20	29
Nov_20	intradomicilio	0	2	2
Nov_20	intradomicilio	1	8	9
Nov_20	peridomicilio	2	9	11
Nov_20	peridomicilio	2	1	3
Nov_20	forest	1	4	5
Nov_20	forest	2	0	2
Nov_20	forest	9	79	88
Nov_20	forest	3	2	5
Nov_20	forest	0	0	0
Nov_20	forest	30	73	103
Nov_20	intradomicilio	1	12	13
Nov_20	intradomicilio	0	9	9

Nov_20	peridomicilio	5	25	30
Nov_20	peridomicilio	1	8	9
Nov_20	forest	0	0	0
Nov_20	forest	16	30	46
Nov_20	forest	1	2	3
Nov_20	forest	1	2	3
Nov_20	forest	57	64	121
Nov_20	forest	6	40	46
Nov_20	intradomicilio	2	8	10
Nov_20	intradomicilio	0	4	4
Nov_20	peridomicilio	2	9	11
Nov_20	peridomicilio	1	3	4
Dec_20	forest	0	0	0
Dec_20	forest	1	20	21
Dec_20	forest	0	1	1
Dec_20	forest	0	0	0
Dec_20	forest	7	32	39
Dec_20	forest	61	75	136
Dec_20	intradomicilio	0	8	8
Dec_20	intradomicilio	0	0	0
Dec_20	peridomicilio	0	8	8
Dec_20	peridomicilio	0	0	0
Dec_20	forest	8	8	16
Dec_20	forest	45	71	116
Dec_20	forest	2	2	4
Dec_20	forest	0	0	0
Dec_20	forest	22	122	144
Dec_20	forest	173	195	368
Dec_20	intradomicilio	0	5	5
Dec_20	intradomicilio	1	14	15
Dec_20	peridomicilio	6	29	35
Dec_20	peridomicilio	4	15	19
Dec_20	forest	0	0	0
Dec_20	forest	1	7	8
Dec_20	forest	0	0	0
Dec_20	forest	0	0	0
Dec_20	forest	5	10	15
Dec_20	forest	12	19	31
Dec_20	intradomicilio	0	0	0
Dec_20	intradomicilio	1	3	4
Dec_20	peridomicilio	0	8	8
Dec_20	peridomicilio	2	1	3
Dec_20	forest	0	4	4

Dec_20	forest	36	12	48
Dec_20	forest	0	0	0
Dec_20	forest	0	0	0
Dec_20	forest	25	15	40
Dec_20	forest	15	7	22
Dec_20	intradomicilio	0	0	0
Dec_20	intradomicilio	1	0	1
Dec_20	peridomicilio	0	1	1
Dec_20	peridomicilio	18	12	30
Dec_20	forest	0	0	0
Dec_20	forest	37	22	59
Dec_20	forest	0	0	0
Dec_20	forest	0	0	0
Dec_20	forest	1	5	6
Dec_20	forest	15	16	31
Dec_20	intradomicilio	8	7	15
Dec_20	intradomicilio	9	6	15
Dec_20	peridomicilio	5	3	8
Dec_20	peridomicilio	0	0	0
Dec_20	forest	0	3	3
Dec_20	forest	24	14	38
Dec_20	forest	0	0	0
Dec_20	forest	0	0	0
Dec_20	forest	0	3	3
Dec_20	forest	3	10	13
Dec_20	intradomicilio	3	26	29
Dec_20	intradomicilio	1	20	21
Dec_20	peridomicilio	22	14	36
Dec_20	peridomicilio	0	2	2
Jan_21	forest	15	35	50
Jan_21	forest	143	175	318
Jan_21	forest	2	19	21
Jan_21	forest	7	12	19
Jan_21	forest	143	171	314
Jan_21	forest	121	179	300
Jan_21	intradomicilio	2	49	51
Jan_21	intradomicilio	3	66	69
Jan_21	peridomicilio	6	20	26
Jan_21	peridomicilio	8	42	50
Jan_21	forest	27	9	36
Jan_21	forest	12	143	155
Jan_21	forest	0	10	10
Jan_21	forest	1	5	6

Jan_21	forest	110	111	221
Jan_21	forest	416	217	633
Jan_21	intradomicilio	1	24	25
Jan_21	intradomicilio	1	55	56
Jan_21	peridomicilio	8	56	64
Jan_21	peridomicilio	0	0	0
Jan_21	forest	0	0	0
Jan_21	forest	12	60	72
Jan_21	forest	0	4	4
Jan_21	forest	0	2	2
Jan_21	forest	65	54	119
Jan_21	forest	546	219	765
Jan_21	intradomicilio	3	12	15
Jan_21	intradomicilio	5	24	29
Jan_21	peridomicilio	0	15	15
Jan_21	peridomicilio	3	23	26
Jan_21	forest	0	1	1
Jan_21	forest	10	133	143
Jan_21	forest	1	2	3
Jan_21	forest	0	3	3
Jan_21	forest	54	86	140
Jan_21	forest	288	328	616
Jan_21	intradomicilio	0	28	28
Jan_21	intradomicilio	6	142	148
Jan_21	peridomicilio	3	26	29
Jan_21	peridomicilio	2	23	25
Jan_21	forest	0	2	2
Jan_21	forest	2	111	113
Jan_21	forest	0	0	0
Jan_21	forest	0	2	2
Jan_21	forest	38	64	102
Jan_21	forest	164	249	413
Jan_21	intradomicilio	3	16	19
Jan_21	intradomicilio	3	99	102
Jan_21	peridomicilio	4	40	44
Jan_21	peridomicilio	0	31	31
Jan_21	forest	1	2	3
Jan_21	forest	10	112	122
Jan_21	forest	0	1	1
Jan_21	forest	0	2	2
Jan_21	forest	41	41	82
Jan_21	forest	199	133	332
Jan_21	intradomicilio	0	0	0

Jan_21	intradomicilio	5	25	30
Jan_21	peridomicilio	5	16	21
Jan_21	peridomicilio	2	11	13
Feb_21	forest	12	332	344
Feb_21	forest	245	1193	1438
Feb_21	forest	6	208	214
Feb_21	forest	7	205	212
Feb_21	forest	257	1091	1348
Feb_21	forest	817	1009	1826
Feb_21	intradomicilio	8	54	62
Feb_21	intradomicilio	12	142	154
Feb_21	peridomicilio	11	67	78
Feb_21	peridomicilio	0	61	61
Feb_21	forest	1	5	6
Feb_21	forest	3	29	32
Feb_21	forest	0	0	0
Feb_21	forest	0	0	0
Feb_21	forest	6	44	50
Feb_21	forest	4	25	29
Feb_21	intradomicilio	6	56	62
Feb_21	intradomicilio	3	69	72
Feb_21	peridomicilio	0	35	35
Feb_21	peridomicilio	6	105	111
Feb_21	forest	2	6	8
Feb_21	forest	0	7	7
Feb_21	forest	2	4	6
Feb_21	forest	1	19	20
Feb_21	forest	15	60	75
Feb_21	forest	36	36	72
Feb_21	intradomicilio	3	43	46
Feb_21	intradomicilio	7	35	42
Feb_21	peridomicilio	5	42	47
Feb_21	peridomicilio	3	18	21
Feb_21	forest	0	0	0
Feb_21	forest	0	2	2
Feb_21	forest	0	1	1
Feb_21	forest	0	0	0
Feb_21	forest	8	45	53
Feb_21	forest	7	35	42
Feb_21	intradomicilio	48	130	178
Feb_21	intradomicilio	47	245	292
Feb_21	peridomicilio	4	33	37
Feb_21	peridomicilio	1	6	7

Feb_21	forest	0	1	1
Feb_21	forest	0	0	0
Feb_21	forest	0	0	0
Feb_21	forest	35	298	333
Feb_21	forest	174	425	599
Feb_21	forest	0	0	0
Feb_21	intradomicilio	2	81	83
Feb_21	intradomicilio	7	363	370
Feb_21	peridomicilio	11	121	132
Feb_21	peridomicilio	1	55	56
Feb_21	forest	0	1	1
Feb_21	forest	5	198	203
Feb_21	forest	2	6	8
Feb_21	forest	0	10	10
Feb_21	forest	90	315	405
Feb_21	forest	52	136	188
Feb_21	intradomicilio	2	75	77
Feb_21	intradomicilio	2	142	144
Feb_21	peridomicilio	1	24	25
Feb_21	peridomicilio	1	18	19
