# Supplementary material for: Spatio—Temporal distribution of a vector of cutaneous leishmaniasis: Pintomyia longiflocosa, in a population from the Colombian Andean Mountains
Source: PLoS Negl Trop Dis. 2024 Jun 17;18(6):e0012237. doi: 10.1371/journal.pntd.0012237 (PMC11213335; doi:10.1371/journal.pntd.0012237)
Supplement: S2 File — (PDF) [file pntd.0012237.s002.pdf]

Date	Time	Temp Out	Out Hum	Rain mm3
2/18/2020	12:00:00 a, m	28.6	53	0
2/18/2020	1:00:00 a, m,	28.3	53	0
2/18/2020	2:00:00 a, m,	28.2	54	0
2/18/2020	3:00:00 a, m,	28.1	54	0
2/18/2020	4:00:00 a, m,	27.9	54	0
2/18/2020	5:00:00 a, m,	27.8	55	0
2/18/2020	6:00:00 a, m,	27.7	55	0
2/18/2020	7:00:00 a, m,	27.6	56	0
2/18/2020	8:00:00 a, m,	27.6	57	0
2/18/2020	9:00:00 a, m,	27.6	57	0
2/18/2020	10:00:00 a, m	27.6	61	0
2/18/2020	11:00:00 a, m	29.1	60	0
2/18/2020	12:00:00 p, m	30.6	43	0.2
2/18/2020	1:00:00 p, m,	28.2	45	0
2/18/2020	2:00:00 p, m,	26.7	47	0
2/18/2020	3:00:00 p, m,	25.9	48	0.4
2/18/2020	4:00:00 p, m,	23.3	59	2.4
2/18/2020	5:00:00 p, m,	22.8	62	0
2/18/2020	6:00:00 p, m,	22.3	56	0
2/18/2020	7:00:00 p, m,	21.9	55	0
2/18/2020	8:00:00 p, m,	21.4	59	0
2/18/2020	9:00:00 p, m,	21.3	59	0
2/18/2020	10:00:00 p, m	21.7	61	0
2/18/2020	11:00:00 p, m	21.7	60	0
2/19/2020	12:00:00 a, m	21.3	61	0
2/19/2020	1:00:00 a, m,	21.2	61	0
2/19/2020	2:00:00 a, m,	21.1	60	0
2/19/2020	3:00:00 a, m,	21.3	60	0
2/19/2020	4:00:00 a, m,	20.9	62	0
2/19/2020	5:00:00 a, m,	20.5	65	0
2/19/2020	6:00:00 a, m,	20.3	65	0
2/19/2020	7:00:00 a, m,	20.4	66	0
2/19/2020	8:00:00 a, m,	20.6	69	0
2/19/2020	9:00:00 a, m,	21.2	68	0
2/19/2020	10:00:00 a, m	21.8	68	0
2/19/2020	11:00:00 a, m	19.8	81	0
2/19/2020	12:00:00 p, m	19	86	0
2/19/2020	1:00:00 p, m,	19.9	77	0
2/19/2020	2:00:00 p, m,	20.9	77	0
2/19/2020	3:00:00 p, m,	22.8	69	0
2/19/2020	4:00:00 p, m,	22.8	67	0
2/19/2020	5:00:00 p, m,	22.7	72	0
2/19/2020	6:00:00 p, m,	21.5	73	0
2/19/2020	7:00:00 p, m,	20.6	74	0
2/19/2020	8:00:00 p, m,	20.2	73	0

2/19/2020	9:00:00 p, m,	20.2	70	0
2/19/2020	10:00:00 p, m	20.3	67	0
2/19/2020	11:00:00 p, m	19.9	68	0
2/20/2020	12:00:00 a, m	20	69	0
2/20/2020	1:00:00 a, m,	19.8	69	0
2/20/2020	2:00:00 a, m,	19.9	67	0
2/20/2020	3:00:00 a, m,	19.4	68	0
2/20/2020	4:00:00 a, m,	19.3	68	0
2/20/2020	5:00:00 a, m,	19.8	67	0
2/20/2020	6:00:00 a, m,	19.8	67	0
2/20/2020	7:00:00 a, m,	19.8	69	0
2/20/2020	8:00:00 a, m,	19.7	72	0
2/20/2020	9:00:00 a, m,	19.9	75	0
2/20/2020	10:00:00 a, m	20.4	76	0
2/20/2020	11:00:00 a, m	21.8	72	0
2/20/2020	12:00:00 p, m	22.6	71	0
2/20/2020	1:00:00 p, m,	23.7	68	0
2/20/2020	2:00:00 p, m,	22.7	71	0
2/20/2020	3:00:00 p, m,	22.2	73	0
2/20/2020	4:00:00 p, m,	22.1	73	0
2/20/2020	5:00:00 p, m,	23.1	68	0
2/20/2020	6:00:00 p, m,	21.9	68	0
2/20/2020	7:00:00 p, m,	21	70	0
2/20/2020	8:00:00 p, m,	21.2	68	0
2/20/2020	9:00:00 p, m,	20.9	68	0
2/20/2020	10:00:00 p, m	20.8	70	0
2/20/2020	11:00:00 p, m	20.4	71	0
2/21/2020	12:00:00 a, m	20.1	73	0
2/21/2020	1:00:00 a, m,	19.8	73	0
2/21/2020	2:00:00 a, m,	19.8	75	0
2/21/2020	3:00:00 a, m,	19.4	76	0
2/21/2020	4:00:00 a, m,	19.3	78	0
2/21/2020	5:00:00 a, m,	19.2	78	0
2/21/2020	6:00:00 a, m,	19.1	79	0
2/21/2020	7:00:00 a, m,	18.9	80	0
2/21/2020	8:00:00 a, m,	19.2	81	0
2/21/2020	9:00:00 a, m,	19.7	81	0
2/21/2020	10:00:00 a, m	20.3	81	0
2/21/2020	11:00:00 a, m	20.7	80	0
2/21/2020	12:00:00 p, m	21.5	78	0
2/21/2020	1:00:00 p, m,	23.2	74	0
2/21/2020	2:00:00 p, m,	24	71	0
2/21/2020	3:00:00 p, m,	24.4	70	0
2/21/2020	4:00:00 p, m,	20.2	84	0.6
2/21/2020	5:00:00 p, m,	18.8	91	1
2/21/2020	6:00:00 p, m,	18.8	90	0
2/21/2020	7:00:00 p, m,	19.2	89	0

2/21/2020	8:00:00 p, m,	19.1	91	0
2/21/2020	9:00:00 p, m,	19.3	88	0
2/21/2020	10:00:00 p, m	19.2	90	0
2/21/2020	11:00:00 p, m	18.6	91	0
2/22/2020	12:00:00 a, m	18.4	91	0
2/22/2020	1:00:00 a, m,	18.4	92	0
2/22/2020	2:00:00 a, m,	18.3	93	0
2/22/2020	3:00:00 a, m,	17.8	94	1.6
2/22/2020	4:00:00 a, m,	17.6	95	1
2/22/2020	5:00:00 a, m,	17.6	96	0.2
2/22/2020	6:00:00 a, m,	17.6	96	1.2
2/22/2020	7:00:00 a, m,	17.3	96	4.4
2/22/2020	8:00:00 a, m,	17.3	97	1
2/22/2020	9:00:00 a, m,	17.7	97	0.2
2/22/2020	10:00:00 a, m	18.4	97	0
2/22/2020	11:00:00 a, m	18.6	97	0
2/22/2020	12:00:00 p, m	18.4	97	0
2/22/2020	1:00:00 p, m,	18.7	97	8.2
2/22/2020	2:00:00 p, m,	19.7	98	0.6
2/22/2020	3:00:00 p, m,	18.1	97	16.4
2/22/2020	4:00:00 p, m,	18.6	98	11.4
2/22/2020	5:00:00 p, m,	18.4	97	1.2
2/22/2020	6:00:00 p, m,	18.3	97	0.2
2/22/2020	7:00:00 p, m,	17.8	97	0
2/22/2020	8:00:00 p, m,	17.7	97	0
2/22/2020	9:00:00 p, m,	17.4	97	0
2/22/2020	10:00:00 p, m	17.5	97	0
2/22/2020	11:00:00 p, m	17.3	97	0
2/23/2020	12:00:00 a, m	17.3	97	0
2/23/2020	1:00:00 a, m,	17.2	97	0
2/23/2020	2:00:00 a, m,	17.3	97	0
2/23/2020	3:00:00 a, m,	17.3	97	0
2/23/2020	4:00:00 a, m,	17.3	97	0
2/23/2020	5:00:00 a, m,	17.3	98	0
2/23/2020	6:00:00 a, m,	17.3	97	0
2/23/2020	7:00:00 a, m,	17.3	97	0
2/23/2020	8:00:00 a, m,	17.7	98	0
2/23/2020	9:00:00 a, m,	18	98	0
2/23/2020	10:00:00 a, m	18.3	98	0
2/23/2020	11:00:00 a, m	18.3	98	0.2
2/23/2020	12:00:00 p, m	18.3	98	2.2
2/23/2020	1:00:00 p, m,	18	98	4.8
2/23/2020	2:00:00 p, m,	18.7	98	2.2
2/23/2020	3:00:00 p, m,	18.8	98	0.2
2/23/2020	4:00:00 p, m,	18.3	98	0
2/23/2020	5:00:00 p, m,	17.8	98	0.4
2/23/2020	6:00:00 p, m,	17.8	98	0.2

2/23/2020	7:00:00 p, m,	17.3	98	0.2
2/23/2020	8:00:00 p, m,	17	97	0
2/23/2020	9:00:00 p, m,	16.7	97	0
2/23/2020	10:00:00 p, m	16.9	95	0
2/23/2020	11:00:00 p, m	16.8	97	0
2/24/2020	12:00:00 a, m	16.5	96	0
2/24/2020	1:00:00 a, m,	16.4	97	0
2/24/2020	2:00:00 a, m,	16.2	96	0
2/24/2020	3:00:00 a, m,	16.3	96	0
2/24/2020	4:00:00 a, m,	16.3	96	0
2/24/2020	5:00:00 a, m,	16.2	96	0
2/24/2020	6:00:00 a, m,	16.2	96	0
2/24/2020	7:00:00 a, m,	16.3	96	0
2/24/2020	8:00:00 a, m,	16.7	97	0
2/24/2020	9:00:00 a, m,	17.2	98	0
2/24/2020	10:00:00 a, m	17.8	98	0
2/24/2020	11:00:00 a, m	18.3	98	0
2/24/2020	12:00:00 p, m	18.9	97	0
2/24/2020	1:00:00 p, m,	19.6	94	0
2/24/2020	2:00:00 p, m,	20.3	93	0
2/24/2020	3:00:00 p, m,	20.3	93	4
2/24/2020	4:00:00 p, m,	19.3	97	11.6
2/24/2020	5:00:00 p, m,	19.3	98	0.2
2/24/2020	6:00:00 p, m,	19	97	0
2/24/2020	7:00:00 p, m,	18.4	97	0
2/24/2020	8:00:00 p, m,	18.2	98	0
2/24/2020	9:00:00 p, m,	18	97	0.2
2/24/2020	10:00:00 p, m	17.5	97	0.2
2/24/2020	11:00:00 p, m	17.6	97	0
2/25/2020	12:00:00 a, m	17.5	97	0
2/25/2020	1:00:00 a, m,	17.6	96	0
2/25/2020	2:00:00 a, m,	17.4	96	0
2/25/2020	3:00:00 a, m,	17.6	97	0
2/25/2020	4:00:00 a, m,	17.5	97	0
2/25/2020	5:00:00 a, m,	17.3	96	0
2/25/2020	6:00:00 a, m,	17.4	97	0
2/25/2020	7:00:00 a, m,	17.7	98	0
2/25/2020	8:00:00 a, m,	17.8	98	0
2/25/2020	9:00:00 a, m,	18.2	98	0
2/25/2020	10:00:00 a, m	18.4	98	0
2/25/2020	11:00:00 a, m	18.6	99	0
2/25/2020	12:00:00 p, m	19.2	98	0
2/25/2020	1:00:00 p, m,	20	98	0
2/25/2020	2:00:00 p, m,	19.8	97	0
2/25/2020	3:00:00 p, m,	20	99	0
2/25/2020	4:00:00 p, m,	20.1	99	0
2/25/2020	5:00:00 p, m,	20.1	98	0

2/25/2020	6:00:00 p, m,	20.2	98	0
2/25/2020	7:00:00 p, m,	19.4	98	0
2/25/2020	8:00:00 p, m,	19.1	98	0
2/25/2020	9:00:00 p, m,	18.6	97	0
2/25/2020	10:00:00 p, m	18.6	95	0
2/25/2020	11:00:00 p, m	18.4	94	0
2/26/2020	12:00:00 a, m	18.3	95	0
2/26/2020	1:00:00 a, m,	18.2	95	0
2/26/2020	2:00:00 a, m,	18.3	97	0
2/26/2020	3:00:00 a, m,	17.9	96	0
2/26/2020	4:00:00 a, m,	17.9	98	0
2/26/2020	5:00:00 a, m,	17.6	98	0
2/26/2020	6:00:00 a, m,	17.2	99	2.2
2/26/2020	7:00:00 a, m,	16.9	99	3.2
2/26/2020	8:00:00 a, m,	16.3	98	4.4
2/26/2020	9:00:00 a, m,	16.5	99	1.2
2/26/2020	10:00:00 a, m	16.9	99	0.2
2/26/2020	11:00:00 a, m	17.3	99	0
2/26/2020	12:00:00 p, m	17.8	99	0
2/26/2020	1:00:00 p, m,	18.4	99	0
2/26/2020	2:00:00 p, m,	17.8	99	3.2
2/26/2020	3:00:00 p, m,	18.1	99	2.2
2/26/2020	4:00:00 p, m,	18.3	99	0.6
2/26/2020	5:00:00 p, m,	18.1	99	0.2
2/26/2020	6:00:00 p, m,	17.8	99	0
2/26/2020	7:00:00 p, m,	17.2	98	0
2/26/2020	8:00:00 p, m,	16.6	98	0.2
2/26/2020	9:00:00 p, m,	16.5	98	0
2/26/2020	10:00:00 p, m	16.1	98	0
2/26/2020	11:00:00 p, m	16.1	98	0
2/27/2020	12:00:00 a, m	16.2	96	0
2/27/2020	1:00:00 a, m,	16.3	95	0
2/27/2020	2:00:00 a, m,	16.7	96	0
2/27/2020	3:00:00 a, m,	16.8	97	0
2/27/2020	4:00:00 a, m,	16.7	98	1.8
2/27/2020	5:00:00 a, m,	16.7	98	19
2/27/2020	6:00:00 a, m,	16.7	98	2.2
2/27/2020	7:00:00 a, m,	16.2	98	0
2/27/2020	8:00:00 a, m,	16.2	99	0
2/27/2020	9:00:00 a, m,	17.1	99	0
2/27/2020	10:00:00 a, m	17.7	99	0
2/27/2020	11:00:00 a, m	18.5	99	0
2/27/2020	12:00:00 p, m	18.4	99	0
2/27/2020	1:00:00 p, m,	19.1	99	0
2/27/2020	2:00:00 p, m,	19.3	99	0
2/27/2020	3:00:00 p, m,	20.1	96	0
2/27/2020	4:00:00 p, m,	20.6	92	0

2/27/2020	5:00:00 p, m,	20.7	94	0
2/27/2020	6:00:00 p, m,	19.4	93	0
2/27/2020	7:00:00 p, m,	18.4	94	0
2/27/2020	8:00:00 p, m,	18.4	94	0
2/27/2020	9:00:00 p, m,	18.4	94	0
2/27/2020	10:00:00 p, m	17.8	97	1.8
2/27/2020	11:00:00 p, m	17.4	98	4.2
2/28/2020	12:00:00 a, m	17.4	98	0.2
2/28/2020	1:00:00 a, m,	17.3	98	1.2
2/28/2020	2:00:00 a, m,	17.2	98	1
2/28/2020	3:00:00 a, m,	17.1	98	0.4
2/28/2020	4:00:00 a, m,	16.8	98	3.2
2/28/2020	5:00:00 a, m,	16.9	99	6.2
2/28/2020	6:00:00 a, m,	17	99	1
2/28/2020	7:00:00 a, m,	17	99	3.4
2/28/2020	8:00:00 a, m,	17.3	99	0
2/28/2020	9:00:00 a, m,	17.7	99	0.2
2/28/2020	10:00:00 a, m	18.4	99	0
2/28/2020	11:00:00 a, m	18	99	0
2/28/2020	12:00:00 p, m	18.6	100	0
2/28/2020	1:00:00 p, m,	18.8	99	0
2/28/2020	2:00:00 p, m,	18.9	99	0
2/28/2020	3:00:00 p, m,	19.3	100	0
2/28/2020	4:00:00 p, m,	19.2	99	0
2/28/2020	5:00:00 p, m,	18.8	99	0
2/28/2020	6:00:00 p, m,	18.4	98	0
2/28/2020	7:00:00 p, m,	17.9	97	0
2/28/2020	8:00:00 p, m,	17.9	97	0
2/28/2020	9:00:00 p, m,	18.1	96	0
2/28/2020	10:00:00 p, m	18.1	95	0
2/28/2020	11:00:00 p, m	18.1	95	0
2/29/2020	12:00:00 a, m	18.1	95	0
2/29/2020	1:00:00 a, m,	18.2	95	0
2/29/2020	2:00:00 a, m,	18.1	94	0
2/29/2020	3:00:00 a, m,	18.3	95	0
2/29/2020	4:00:00 a, m,	18.2	94	0
2/29/2020	5:00:00 a, m,	17.7	94	0
2/29/2020	6:00:00 a, m,	17.2	95	0
2/29/2020	7:00:00 a, m,	17.3	97	0
2/29/2020	8:00:00 a, m,	17.8	97	0
2/29/2020	9:00:00 a, m,	18.2	98	0
2/29/2020	10:00:00 a, m	18.7	98	0.2
2/29/2020	11:00:00 a, m	19.2	98	0
2/29/2020	12:00:00 p, m	19.3	96	0
2/29/2020	1:00:00 p, m,	20.3	94	0
2/29/2020	2:00:00 p, m,	21	92	0
2/29/2020	3:00:00 p, m,	20.2	97	0

2/29/2020	4:00:00 p, m,	21.2	95	0
2/29/2020	5:00:00 p, m,	20.9	95	0
2/29/2020	6:00:00 p, m,	20.1	94	0
2/29/2020	7:00:00 p, m,	19.2	92	0
2/29/2020	8:00:00 p, m,	18.7	92	0
2/29/2020	9:00:00 p, m,	18.7	88	0
2/29/2020	10:00:00 p, m	18.5	91	0
2/29/2020	11:00:00 p, m	18.4	87	0
Feb_20		19.3399306	87.4826389	142.4
3/1/2020	12:00:00 a, m	18.3	90	0
3/1/2020	1:00:00 a, m,	18.1	93	0
3/1/2020	2:00:00 a, m,	18.3	92	0
3/1/2020	3:00:00 a, m,	18.3	94	0
3/1/2020	4:00:00 a, m,	18.2	93	0
3/1/2020	5:00:00 a, m,	17.7	94	0
3/1/2020	6:00:00 a, m,	17.6	93	0
3/1/2020	7:00:00 a, m,	17.8	94	0
3/1/2020	8:00:00 a, m,	17.9	95	0
3/1/2020	9:00:00 a, m,	18.4	96	0
3/1/2020	10:00:00 a, m	19.1	96	0
3/1/2020	11:00:00 a, m	19.8	94	0
3/1/2020	12:00:00 p, m	20.7	91	0
3/1/2020	1:00:00 p, m,	21.1	84	0
3/1/2020	2:00:00 p, m,	21.4	89	0
3/1/2020	3:00:00 p, m,	20.7	93	0
3/1/2020	4:00:00 p, m,	21.9	83	0
3/1/2020	5:00:00 p, m,	21.2	88	0
3/1/2020	6:00:00 p, m,	20.2	90	0
3/1/2020	7:00:00 p, m,	19.3	91	0
3/1/2020	8:00:00 p, m,	19.3	91	0
3/1/2020	9:00:00 p, m,	19.2	84	0
3/1/2020	10:00:00 p, m	19.2	82	0
3/1/2020	11:00:00 p, m	19.3	84	0
3/2/2020	12:00:00 a, m	19.1	84	0
3/2/2020	1:00:00 a, m,	19.3	82	0
3/2/2020	2:00:00 a, m,	19.1	84	0
3/2/2020	3:00:00 a, m,	18.9	86	0
3/2/2020	4:00:00 a, m,	18.7	87	0
3/2/2020	5:00:00 a, m,	18.7	87	0
3/2/2020	6:00:00 a, m,	18.7	88	0
3/2/2020	7:00:00 a, m,	18.7	87	0
3/2/2020	8:00:00 a, m,	18.6	90	0
3/2/2020	9:00:00 a, m,	18.8	90	0
3/2/2020	10:00:00 a, m	18.8	93	0
3/2/2020	11:00:00 a, m	19.2	93	0
3/2/2020	12:00:00 p, m	20.1	91	0
3/2/2020	1:00:00 p, m,	21.7	86	0

3/2/2020	2:00:00 p, m,	22.8	82	0
3/2/2020	3:00:00 p, m,	21.9	87	0
3/2/2020	4:00:00 p, m,	20.9	90	0
3/2/2020	5:00:00 p, m,	20.6	88	0
3/2/2020	6:00:00 p, m,	19.8	84	0
3/2/2020	7:00:00 p, m,	19.6	83	0
3/2/2020	8:00:00 p, m,	19.7	82	0
3/2/2020	9:00:00 p, m,	19.5	81	0
3/2/2020	10:00:00 p, m	19.4	79	0
3/2/2020	11:00:00 p, m	19.2	81	0
3/3/2020	12:00:00 a, m	18.6	82	0
3/3/2020	1:00:00 a, m,	18.2	83	0
3/3/2020	2:00:00 a, m,	18.2	83	0
3/3/2020	3:00:00 a, m,	18.1	83	0
3/3/2020	4:00:00 a, m,	17.8	84	0
3/3/2020	5:00:00 a, m,	17.7	85	0
3/3/2020	6:00:00 a, m,	17.7	85	0
3/3/2020	7:00:00 a, m,	17.9	84	0
3/3/2020	8:00:00 a, m,	18.3	86	0
3/3/2020	9:00:00 a, m,	18.8	85	0
3/3/2020	10:00:00 a, m	19	86	0
3/3/2020	11:00:00 a, m	19.2	88	0
3/3/2020	12:00:00 p, m	19.6	88	0
3/3/2020	1:00:00 p, m,	19.6	88	0
3/3/2020	2:00:00 p, m,	20	85	0
3/3/2020	3:00:00 p, m,	20.5	86	0
3/3/2020	4:00:00 p, m,	20.6	86	0
3/3/2020	5:00:00 p, m,	20.6	89	0
3/3/2020	6:00:00 p, m,	20.3	87	0
3/3/2020	7:00:00 p, m,	19.2	86	0
3/3/2020	8:00:00 p, m,	19.2	85	0
3/3/2020	9:00:00 p, m,	19.1	78	0
3/3/2020	10:00:00 p, m	18.9	82	0
3/3/2020	11:00:00 p, m	18.8	82	0
3/4/2020	12:00:00 a, m	18.6	81	0
3/4/2020	1:00:00 a, m,	18.2	83	0
3/4/2020	2:00:00 a, m,	18.5	82	0
3/4/2020	3:00:00 a, m,	18.9	81	0
3/4/2020	4:00:00 a, m,	18.8	82	0
3/4/2020	5:00:00 a, m,	18.7	82	0
3/4/2020	6:00:00 a, m,	18.9	81	0
3/4/2020	7:00:00 a, m,	19	81	0
3/4/2020	8:00:00 a, m,	18.3	91	0
3/4/2020	9:00:00 a, m,	17.6	93	0
3/4/2020	10:00:00 a, m	18.6	94	0
3/4/2020	11:00:00 a, m	19.4	92	0
3/4/2020	12:00:00 p, m	20.7	86	0

3/4/2020	1:00:00 p, m,	21.3	85	0
3/4/2020	2:00:00 p, m,	21.9	84	0
3/4/2020	3:00:00 p, m,	21.5	85	0
3/4/2020	4:00:00 p, m,	22.3	83	0
3/4/2020	5:00:00 p, m,	22.3	84	0
3/4/2020	6:00:00 p, m,	21.4	78	0
3/4/2020	7:00:00 p, m,	20.9	76	0
3/4/2020	8:00:00 p, m,	20.5	75	0
3/4/2020	9:00:00 p, m,	20.4	78	0
3/4/2020	10:00:00 p, m	20.2	74	0
3/4/2020	11:00:00 p, m	20.1	76	0
3/5/2020	12:00:00 a, m	19.9	77	0
3/5/2020	1:00:00 a, m,	19.3	76	0
3/5/2020	2:00:00 a, m,	19.1	80	0
3/5/2020	3:00:00 a, m,	18.7	80	0
3/5/2020	4:00:00 a, m,	18.7	78	0
3/5/2020	5:00:00 a, m,	18.6	77	0
3/5/2020	6:00:00 a, m,	18.6	75	0
3/5/2020	7:00:00 a, m,	18.1	77	0
3/5/2020	8:00:00 a, m,	18.7	76	0
3/5/2020	9:00:00 a, m,	19.1	81	0
3/5/2020	10:00:00 a, m	19.8	81	0
3/5/2020	11:00:00 a, m	20.8	83	0
3/5/2020	12:00:00 p, m	21.3	79	0
3/5/2020	1:00:00 p, m,	21.4	77	0
3/5/2020	2:00:00 p, m,	21	83	0
3/5/2020	3:00:00 p, m,	20.8	83	0
3/5/2020	4:00:00 p, m,	21.7	80	0
3/5/2020	5:00:00 p, m,	21.8	82	0
3/5/2020	6:00:00 p, m,	21.3	83	0
3/5/2020	7:00:00 p, m,	20.2	83	0
3/5/2020	8:00:00 p, m,	19.8	79	0
3/5/2020	9:00:00 p, m,	20.3	74	0
3/5/2020	10:00:00 p, m	20	72	0
3/5/2020	11:00:00 p, m	19.4	74	0
3/6/2020	12:00:00 a, m	19.2	76	0
3/6/2020	1:00:00 a, m,	18.8	77	0
3/6/2020	2:00:00 a, m,	18.4	78	0
3/6/2020	3:00:00 a, m,	18.4	78	0
3/6/2020	4:00:00 a, m,	18.4	78	0
3/6/2020	5:00:00 a, m,	18.2	79	0
3/6/2020	6:00:00 a, m,	18.1	80	0
3/6/2020	7:00:00 a, m,	18.2	80	0
3/6/2020	8:00:00 a, m,	18.6	81	0
3/6/2020	9:00:00 a, m,	18.9	84	0
3/6/2020	10:00:00 a, m	19.7	83	0
3/6/2020	11:00:00 a, m	21	75	0

3/6/2020	12:00:00 p, m	21.7	77	0
3/6/2020	1:00:00 p, m,	22.8	72	0
3/6/2020	2:00:00 p, m,	23.5	68	0
3/6/2020	3:00:00 p, m,	23.8	72	0
3/6/2020	4:00:00 p, m,	23.1	75	0
3/6/2020	5:00:00 p, m,	22.4	79	0
3/6/2020	6:00:00 p, m,	21.7	80	0
3/6/2020	7:00:00 p, m,	21	78	0
3/6/2020	8:00:00 p, m,	20.7	81	0
3/6/2020	9:00:00 p, m,	20.8	79	0
3/6/2020	10:00:00 p, m	20.7	77	0
3/6/2020	11:00:00 p, m	20.3	80	0
3/7/2020	12:00:00 a, m	20.1	79	0
3/7/2020	1:00:00 a, m,	19.7	80	0
3/7/2020	2:00:00 a, m,	19.8	85	0
3/7/2020	3:00:00 a, m,	19.5	85	0
3/7/2020	4:00:00 a, m,	18.8	87	0
3/7/2020	5:00:00 a, m,	18.7	87	0
3/7/2020	6:00:00 a, m,	18.3	88	0
3/7/2020	7:00:00 a, m,	18.7	89	0
3/7/2020	8:00:00 a, m,	18.8	88	0
3/7/2020	9:00:00 a, m,	19.2	91	0
3/7/2020	10:00:00 a, m	20.3	92	0
3/7/2020	11:00:00 a, m	20.3	92	0
3/7/2020	12:00:00 p, m	21.6	85	0
3/7/2020	1:00:00 p, m,	20.5	85	0
3/7/2020	2:00:00 p, m,	21.1	88	0
3/7/2020	3:00:00 p, m,	21.8	80	0
3/7/2020	4:00:00 p, m,	20.5	85	0
3/7/2020	5:00:00 p, m,	19.8	89	0
3/7/2020	6:00:00 p, m,	19.8	85	0
3/7/2020	7:00:00 p, m,	19.3	86	0
3/7/2020	8:00:00 p, m,	18.9	86	0
3/7/2020	9:00:00 p, m,	18.9	87	0
3/7/2020	10:00:00 p, m	18.9	87	0
3/7/2020	11:00:00 p, m	18.8	87	0
3/8/2020	12:00:00 a, m	18.6	90	0
3/8/2020	1:00:00 a, m,	17.9	90	0
3/8/2020	2:00:00 a, m,	17.8	87	0
3/8/2020	3:00:00 a, m,	17.7	88	0
3/8/2020	4:00:00 a, m,	17.4	87	0
3/8/2020	5:00:00 a, m,	17.3	87	0
3/8/2020	6:00:00 a, m,	17.1	88	0
3/8/2020	7:00:00 a, m,	17.3	89	0
3/8/2020	8:00:00 a, m,	17.4	88	0
3/8/2020	9:00:00 a, m,	17.9	91	0
3/8/2020	10:00:00 a, m	18.8	94	0

3/8/2020	11:00:00 a, m	19.5	92	0
3/8/2020	12:00:00 p, m	20.1	87	0
3/8/2020	1:00:00 p, m,	20.6	89	0
3/8/2020	2:00:00 p, m,	21.3	84	0
3/8/2020	3:00:00 p, m,	20.4	86	0
3/8/2020	4:00:00 p, m,	19.2	93	0.2
3/8/2020	5:00:00 p, m,	19.7	86	0
3/8/2020	6:00:00 p, m,	19.6	86	0
3/8/2020	7:00:00 p, m,	19.1	84	0
3/8/2020	8:00:00 p, m,	19.3	81	0
3/8/2020	9:00:00 p, m,	19.3	82	0
3/8/2020	10:00:00 p, m	19.1	84	0
3/8/2020	11:00:00 p, m	19.3	82	0
3/9/2020	12:00:00 a, m	18.6	84	0
3/9/2020	1:00:00 a, m,	18.7	85	0
3/9/2020	2:00:00 a, m,	18.4	86	0
3/9/2020	3:00:00 a, m,	17.9	86	0
3/9/2020	4:00:00 a, m,	17.7	84	0
3/9/2020	5:00:00 a, m,	17.8	85	0
3/9/2020	6:00:00 a, m,	17.8	88	0
3/9/2020	7:00:00 a, m,	17.8	88	0
3/9/2020	8:00:00 a, m,	18.2	88	0
3/9/2020	9:00:00 a, m,	18.8	92	0
3/9/2020	10:00:00 a, m	19.1	88	0
3/9/2020	11:00:00 a, m	19.4	93	0
3/9/2020	12:00:00 p, m	19.5	90	0
3/9/2020	1:00:00 p, m,	19.2	93	0
3/9/2020	2:00:00 p, m,	19.6	94	0
3/9/2020	3:00:00 p, m,	20.3	94	0
3/9/2020	4:00:00 p, m,	19.9	94	0
3/9/2020	5:00:00 p, m,	19.8	93	0
3/9/2020	6:00:00 p, m,	19.6	89	0
3/9/2020	7:00:00 p, m,	19.2	88	0
3/9/2020	8:00:00 p, m,	19.1	88	0
3/9/2020	9:00:00 p, m,	18.9	85	0
3/9/2020	10:00:00 p, m	18.9	86	0
3/9/2020	11:00:00 p, m	18.8	85	0
3/10/2020	12:00:00 a, m	18.8	85	0
3/10/2020	1:00:00 a, m,	18.7	85	0
3/10/2020	2:00:00 a, m,	18.6	85	0
3/10/2020	3:00:00 a, m,	18.3	84	0
3/10/2020	4:00:00 a, m,	18.1	87	0
3/10/2020	5:00:00 a, m,	17.6	87	0
3/10/2020	6:00:00 a, m,	17.3	87	0
3/10/2020	7:00:00 a, m,	17.6	89	0
3/10/2020	8:00:00 a, m,	18.1	90	0
3/10/2020	9:00:00 a, m,	18.7	91	0

3/10/2020	10:00:00 a, m	19.5	92	0
3/10/2020	11:00:00 a, m	20.4	90	0
3/10/2020	12:00:00 p, m	21.9	87	0
3/10/2020	1:00:00 p, m,	23	80	0
3/10/2020	2:00:00 p, m,	22.8	76	0
3/10/2020	3:00:00 p, m,	21.8	72	0
3/10/2020	4:00:00 p, m,	22.3	76	0
3/10/2020	5:00:00 p, m,	22.2	75	0
3/10/2020	6:00:00 p, m,	21.4	78	0
3/10/2020	7:00:00 p, m,	20.8	78	0
3/10/2020	8:00:00 p, m,	20.5	75	0
3/10/2020	9:00:00 p, m,	20.7	75	0
3/10/2020	10:00:00 p, m	20.3	73	0
3/10/2020	11:00:00 p, m	19.9	74	0
3/11/2020	12:00:00 a, m	19.7	78	0
3/11/2020	1:00:00 a, m,	19	82	0
3/11/2020	2:00:00 a, m,	18.8	82	0
3/11/2020	3:00:00 a, m,	18.4	84	0
3/11/2020	4:00:00 a, m,	18.3	86	0
3/11/2020	5:00:00 a, m,	17.9	88	0
3/11/2020	6:00:00 a, m,	17.5	88	0
3/11/2020	7:00:00 a, m,	17.4	90	0
3/11/2020	8:00:00 a, m,	18	91	0
3/11/2020	9:00:00 a, m,	18.3	92	0
3/11/2020	10:00:00 a, m	18.8	93	0
3/11/2020	11:00:00 a, m	20.8	92	0
3/11/2020	12:00:00 p, m	22.1	88	0.8
3/11/2020	1:00:00 p, m,	23.2	83	0
3/11/2020	2:00:00 p, m,	21.4	83	16.6
3/11/2020	3:00:00 p, m,	19.9	96	6
3/11/2020	4:00:00 p, m,	20.6	96	0
3/11/2020	5:00:00 p, m,	19.4	93	0
3/11/2020	6:00:00 p, m,	18.9	95	0
3/11/2020	7:00:00 p, m,	18.8	94	0
3/11/2020	8:00:00 p, m,	18.7	93	0
3/11/2020	9:00:00 p, m,	18.4	95	0
3/11/2020	10:00:00 p, m	18.7	94	0
3/11/2020	11:00:00 p, m	18.8	96	0
3/12/2020	12:00:00 a, m	18.4	95	0
3/12/2020	1:00:00 a, m,	18.3	97	0
3/12/2020	2:00:00 a, m,	18.2	98	0
3/12/2020	3:00:00 a, m,	17.9	98	0
3/12/2020	4:00:00 a, m,	17.3	98	0
3/12/2020	5:00:00 a, m,	17	98	0
3/12/2020	6:00:00 a, m,	17.1	98	0
3/12/2020	7:00:00 a, m,	17.4	98	0.2
3/12/2020	8:00:00 a, m,	17.7	98	0

3/12/2020	9:00:00 a, m,	17.7	97	0
3/12/2020	10:00:00 a, m	18	98	0
3/12/2020	11:00:00 a, m	18.2	97	0
3/12/2020	12:00:00 p, m	18.7	97	0
3/12/2020	1:00:00 p, m,	19.4	95	0
3/12/2020	2:00:00 p, m,	20.4	93	0
3/12/2020	3:00:00 p, m,	20.8	90	0
3/12/2020	4:00:00 p, m,	20.7	86	0
3/12/2020	5:00:00 p, m,	19.7	93	0
3/12/2020	6:00:00 p, m,	18.8	89	0
3/12/2020	7:00:00 p, m,	18.4	89	0
3/12/2020	8:00:00 p, m,	18.6	89	0
3/12/2020	9:00:00 p, m,	18.3	89	0
3/12/2020	10:00:00 p, m	18.5	86	0
3/12/2020	11:00:00 p, m	18.6	86	0
3/13/2020	12:00:00 a, m	18.8	85	0
3/13/2020	1:00:00 a, m,	18.7	83	0
3/13/2020	2:00:00 a, m,	18.6	85	0
3/13/2020	3:00:00 a, m,	18.4	85	0
3/13/2020	4:00:00 a, m,	18.2	87	0
3/13/2020	5:00:00 a, m,	18.2	89	0
3/13/2020	6:00:00 a, m,	17.7	90	0
3/13/2020	7:00:00 a, m,	17.7	91	0
3/13/2020	8:00:00 a, m,	17.9	93	0
3/13/2020	9:00:00 a, m,	18.8	96	0
3/13/2020	10:00:00 a, m	19.9	94	0
3/13/2020	11:00:00 a, m	20.6	93	0
3/13/2020	12:00:00 p, m	21.9	88	0
3/13/2020	1:00:00 p, m,	19.9	90	3.2
3/13/2020	2:00:00 p, m,	20.3	98	1
3/13/2020	3:00:00 p, m,	21.2	97	1.2
3/13/2020	4:00:00 p, m,	21.4	97	0.2
3/13/2020	5:00:00 p, m,	19.1	97	6
3/13/2020	6:00:00 p, m,	18.4	97	0.2
3/13/2020	7:00:00 p, m,	18.1	97	0
3/13/2020	8:00:00 p, m,	18.4	96	0
3/13/2020	9:00:00 p, m,	18	97	0.8
3/13/2020	10:00:00 p, m	18	98	0
3/13/2020	11:00:00 p, m	18	97	0
3/14/2020	12:00:00 a, m	17.4	97	0
3/14/2020	1:00:00 a, m,	17	97	0
3/14/2020	2:00:00 a, m,	17	98	0
3/14/2020	3:00:00 a, m,	16.7	97	0
3/14/2020	4:00:00 a, m,	17	97	0
3/14/2020	5:00:00 a, m,	16.9	98	0
3/14/2020	6:00:00 a, m,	16.9	98	0
3/14/2020	7:00:00 a, m,	17.1	98	0.2

3/14/2020	8:00:00 a, m,	17.3	99	0
3/14/2020	9:00:00 a, m,	17.7	99	0
3/14/2020	10:00:00 a, m	17.7	98	0
3/14/2020	11:00:00 a, m	18.4	99	0
3/14/2020	12:00:00 p, m	18.9	98	0
3/14/2020	1:00:00 p, m,	20.2	94	0
3/14/2020	2:00:00 p, m,	19.2	96	0
3/14/2020	3:00:00 p, m,	19.6	95	0
3/14/2020	4:00:00 p, m,	19.2	96	0.2
3/14/2020	5:00:00 p, m,	17.9	96	0
3/14/2020	6:00:00 p, m,	17.8	97	0
3/14/2020	7:00:00 p, m,	17.6	97	0
3/14/2020	8:00:00 p, m,	17.4	96	0
3/14/2020	9:00:00 p, m,	17.4	96	0
3/14/2020	10:00:00 p, m	17.4	96	0
3/14/2020	11:00:00 p, m	17.7	97	0
3/15/2020	12:00:00 a, m	17.5	97	0
3/15/2020	1:00:00 a, m,	17.1	97	0.2
3/15/2020	2:00:00 a, m,	16.5	97	0.4
3/15/2020	3:00:00 a, m,	16.4	97	0
3/15/2020	4:00:00 a, m,	16.2	98	3.8
3/15/2020	5:00:00 a, m,	16.2	98	1.4
3/15/2020	6:00:00 a, m,	16.2	98	0
3/15/2020	7:00:00 a, m,	16.5	98	0
3/15/2020	8:00:00 a, m,	17.2	99	0
3/15/2020	9:00:00 a, m,	17.4	98	0
3/15/2020	10:00:00 a, m	17.9	99	0
3/15/2020	11:00:00 a, m	18.1	98	0
3/15/2020	12:00:00 p, m	18.2	98	0
3/15/2020	1:00:00 p, m,	19	97	0
3/15/2020	2:00:00 p, m,	19.5	97	0
3/15/2020	3:00:00 p, m,	19.2	98	0
3/15/2020	4:00:00 p, m,	18.9	98	0
3/15/2020	5:00:00 p, m,	18.8	98	0
3/15/2020	6:00:00 p, m,	18.6	98	0
3/15/2020	7:00:00 p, m,	18.4	98	0
3/15/2020	8:00:00 p, m,	18.3	98	0
3/15/2020	9:00:00 p, m,	17.8	97	0
3/15/2020	10:00:00 p, m	17.8	98	0
3/15/2020	11:00:00 p, m	17.9	98	0
3/16/2020	12:00:00 a, m	17.6	98	0
3/16/2020	1:00:00 a, m,	17.5	98	0
3/16/2020	2:00:00 a, m,	17.5	98	0
3/16/2020	3:00:00 a, m,	17.3	98	0.4
3/16/2020	4:00:00 a, m,	17.3	98	0.2
3/16/2020	5:00:00 a, m,	17.3	98	0
3/16/2020	6:00:00 a, m,	17.2	98	0

3/16/2020	7:00:00 a, m,	17.6	99	0
3/16/2020	8:00:00 a, m,	17.9	99	0
3/16/2020	9:00:00 a, m,	18.4	99	0
3/16/2020	10:00:00 a, m	18.9	98	0
3/16/2020	11:00:00 a, m	18.5	99	0
3/16/2020	12:00:00 p, m	18.8	98	0
3/16/2020	1:00:00 p, m,	19.8	97	0
3/16/2020	2:00:00 p, m,	21	86	0
3/16/2020	3:00:00 p, m,	21.2	90	0
3/16/2020	4:00:00 p, m,	20.1	97	0
3/16/2020	5:00:00 p, m,	20.1	97	0
3/16/2020	6:00:00 p, m,	19.1	97	0
3/16/2020	7:00:00 p, m,	18.7	96	0
3/16/2020	8:00:00 p, m,	18.2	94	0
3/16/2020	9:00:00 p, m,	17.9	93	0
3/16/2020	10:00:00 p, m	18.2	95	0
3/16/2020	11:00:00 p, m	18.2	96	0
3/17/2020	12:00:00 a, m	18.1	97	1.8
3/17/2020	1:00:00 a, m,	16.3	97	30.2
3/17/2020	2:00:00 a, m,	15.9	97	4.2
3/17/2020	3:00:00 a, m,	15.8	98	2
3/17/2020	4:00:00 a, m,	15.8	98	6
3/17/2020	5:00:00 a, m,	15.5	98	0.4
3/17/2020	6:00:00 a, m,	15.8	99	0
3/17/2020	7:00:00 a, m,	16.9	99	0
3/17/2020	8:00:00 a, m,	17.3	99	0
3/17/2020	9:00:00 a, m,	18.2	99	0
3/17/2020	10:00:00 a, m	18.6	99	0
3/17/2020	11:00:00 a, m	18.8	99	0
3/17/2020	12:00:00 p, m	19.7	99	0
3/17/2020	1:00:00 p, m,	19.9	98	0
3/17/2020	2:00:00 p, m,	20.7	95	0
3/17/2020	3:00:00 p, m,	21.1	92	0
3/17/2020	4:00:00 p, m,	20.6	92	0
3/17/2020	5:00:00 p, m,	19.9	94	0
3/17/2020	6:00:00 p, m,	18.9	95	0
3/17/2020	7:00:00 p, m,	18.4	95	0
3/17/2020	8:00:00 p, m,	18.4	93	0
3/17/2020	9:00:00 p, m,	18.7	95	0
3/17/2020	10:00:00 p, m	18.6	93	0
3/17/2020	11:00:00 p, m	18.2	95	0
3/18/2020	12:00:00 a, m	18.4	94	0
3/18/2020	1:00:00 a, m,	18.6	93	0
3/18/2020	2:00:00 a, m,	17.3	93	0
3/18/2020	3:00:00 a, m,	17.2	94	0
3/18/2020	4:00:00 a, m,	17.2	94	0
3/18/2020	5:00:00 a, m,	17.1	95	0

3/18/2020	6:00:00 a, m,	16.9	96	0
3/18/2020	7:00:00 a, m,	16.9	95	0
3/18/2020	8:00:00 a, m,	17.6	97	0.2
3/18/2020	9:00:00 a, m,	18.2	96	0
3/18/2020	10:00:00 a, m	19.3	94	0
3/18/2020	11:00:00 a, m	19.3	94	0
3/18/2020	12:00:00 p, m	21.6	87	0
3/18/2020	1:00:00 p, m,	21.8	85	0
3/18/2020	2:00:00 p, m,	22.6	83	0
3/18/2020	3:00:00 p, m,	22.8	82	0
3/18/2020	4:00:00 p, m,	21.6	84	0
3/18/2020	5:00:00 p, m,	20.6	90	0
3/18/2020	6:00:00 p, m,	20.1	91	0
3/18/2020	7:00:00 p, m,	19.8	91	0
3/18/2020	8:00:00 p, m,	19.6	91	0
3/18/2020	9:00:00 p, m,	19.3	91	0
3/18/2020	10:00:00 p, m	19.2	90	0
3/18/2020	11:00:00 p, m	19	91	0
3/19/2020	12:00:00 a, m	18.8	93	0
3/19/2020	1:00:00 a, m,	18.7	94	0
3/19/2020	2:00:00 a, m,	18.4	94	0
3/19/2020	3:00:00 a, m,	18.2	94	0
3/19/2020	4:00:00 a, m,	18.1	93	0
3/19/2020	5:00:00 a, m,	17.9	93	0
3/19/2020	6:00:00 a, m,	17.8	95	0
3/19/2020	7:00:00 a, m,	17.9	95	0
3/19/2020	8:00:00 a, m,	18.3	94	0
3/19/2020	9:00:00 a, m,	19.1	95	0
3/19/2020	10:00:00 a, m	19.6	94	0
3/19/2020	11:00:00 a, m	19.8	95	0
3/19/2020	12:00:00 p, m	21.9	88	0
3/19/2020	1:00:00 p, m,	22.3	87	0
3/19/2020	2:00:00 p, m,	22.8	84	0
3/19/2020	3:00:00 p, m,	22.7	81	0
3/19/2020	4:00:00 p, m,	21.8	86	0
3/19/2020	5:00:00 p, m,	20.7	90	0
3/19/2020	6:00:00 p, m,	20.1	91	0
3/19/2020	7:00:00 p, m,	19.9	91	0
3/19/2020	8:00:00 p, m,	19.8	92	0
3/19/2020	9:00:00 p, m,	19.7	91	0
3/19/2020	10:00:00 p, m	19.3	94	0
3/19/2020	11:00:00 p, m	18.3	95	0.2
3/20/2020	12:00:00 a, m	17	97	16.8
3/20/2020	1:00:00 a, m,	17.1	97	1.8
3/20/2020	2:00:00 a, m,	17.1	98	0.4
3/20/2020	3:00:00 a, m,	16.9	98	4
3/20/2020	4:00:00 a, m,	16.6	98	3.2

3/20/2020	5:00:00 a, m,	16.3	98	0.4
3/20/2020	6:00:00 a, m,	16.5	98	0
3/20/2020	7:00:00 a, m,	16.6	98	2.6
3/20/2020	8:00:00 a, m,	16.8	99	1.6
3/20/2020	9:00:00 a, m,	16.7	98	5
3/20/2020	10:00:00 a, m	17.1	99	2
3/20/2020	11:00:00 a, m	17.6	99	0.2
3/20/2020	12:00:00 p, m	18.2	99	0
3/20/2020	1:00:00 p, m,	18.8	97	0
3/20/2020	2:00:00 p, m,	19.5	97	0
3/20/2020	3:00:00 p, m,	18.9	98	0
3/20/2020	4:00:00 p, m,	18.4	98	0
3/20/2020	5:00:00 p, m,	18.4	99	0
3/20/2020	6:00:00 p, m,	17.8	99	0
3/20/2020	7:00:00 p, m,	17.4	98	0
3/20/2020	8:00:00 p, m,	17.4	98	0
3/20/2020	9:00:00 p, m,	17.6	99	0
3/20/2020	10:00:00 p, m	17.4	98	0
3/20/2020	11:00:00 p, m	16.9	97	0
3/21/2020	12:00:00 a, m	16.7	97	0
3/21/2020	1:00:00 a, m,	16.7	97	0
3/21/2020	2:00:00 a, m,	16.3	97	0
3/21/2020	3:00:00 a, m,	15.8	97	0
3/21/2020	4:00:00 a, m,	15.8	97	0
3/21/2020	5:00:00 a, m,	15.7	97	0
3/21/2020	6:00:00 a, m,	15.7	97	0
3/21/2020	7:00:00 a, m,	16.2	98	0
3/21/2020	8:00:00 a, m,	16.7	98	0
3/21/2020	9:00:00 a, m,	17.6	98	0
3/21/2020	10:00:00 a, m	17.6	98	0
3/21/2020	11:00:00 a, m	18.1	97	0
3/21/2020	12:00:00 p, m	19.3	93	0
3/21/2020	1:00:00 p, m,	19.1	94	0
3/21/2020	2:00:00 p, m,	19.4	92	0
3/21/2020	3:00:00 p, m,	19.6	93	0
3/21/2020	4:00:00 p, m,	19.7	90	0
3/21/2020	5:00:00 p, m,	19.1	94	0
3/21/2020	6:00:00 p, m,	18.3	95	0
3/21/2020	7:00:00 p, m,	17.7	94	0
3/21/2020	8:00:00 p, m,	17.3	95	0
3/21/2020	9:00:00 p, m,	16.9	94	0
3/21/2020	10:00:00 p, m	16.8	94	0
3/21/2020	11:00:00 p, m	16.6	93	0
3/22/2020	12:00:00 a, m	16.7	94	0
3/22/2020	1:00:00 a, m,	16.7	93	0
3/22/2020	2:00:00 a, m,	16.5	92	0
3/22/2020	3:00:00 a, m,	16.3	94	0

3/22/2020	4:00:00 a, m,	16	93	0
3/22/2020	5:00:00 a, m,	15.8	94	0
3/22/2020	6:00:00 a, m,	15.8	94	0
3/22/2020	7:00:00 a, m,	16.2	96	0
3/22/2020	8:00:00 a, m,	16.8	96	0
3/22/2020	9:00:00 a, m,	17.6	95	0
3/22/2020	10:00:00 a, m	18.8	92	0
3/22/2020	11:00:00 a, m	20.4	88	0
3/22/2020	12:00:00 p, m	21.3	86	0
3/22/2020	1:00:00 p, m,	21.1	86	0
3/22/2020	3:00:00 p, m,	21.7	84	0
3/22/2020	4:00:00 p, m,	21.8	81	0
3/22/2020	5:00:00 p, m,	21.2	88	0
3/22/2020	6:00:00 p, m,	20.1	89	0
3/22/2020	7:00:00 p, m,	19.4	90	0
3/22/2020	8:00:00 p, m,	18.8	91	0
3/22/2020	9:00:00 p, m,	18.9	88	0
3/22/2020	10:00:00 p, m	18.9	90	0
3/22/2020	11:00:00 p, m	18.9	89	0
3/23/2020	12:00:00 a, m	18.6	89	0
3/23/2020	1:00:00 a, m,	18.6	90	0
3/23/2020	2:00:00 a, m,	18.1	90	0
3/23/2020	3:00:00 a, m,	18.1	91	0
3/23/2020	4:00:00 a, m,	18.1	91	0
3/23/2020	5:00:00 a, m,	17.8	92	0
3/23/2020	6:00:00 a, m,	17.6	95	1
3/23/2020	7:00:00 a, m,	16.9	97	7.4
3/23/2020	8:00:00 a, m,	16.7	98	4
3/23/2020	9:00:00 a, m,	17.1	98	1.4
3/23/2020	10:00:00 a, m	17.6	99	0
3/23/2020	11:00:00 a, m	18.1	99	0
3/23/2020	12:00:00 p, m	18.4	98	0
3/23/2020	1:00:00 p, m,	18.8	98	0
3/23/2020	2:00:00 p, m,	18.9	99	0
3/23/2020	3:00:00 p, m,	18.7	99	0.4
3/23/2020	4:00:00 p, m,	18.8	98	0
3/23/2020	5:00:00 p, m,	18.9	98	0
3/23/2020	6:00:00 p, m,	17.9	97	0
3/23/2020	7:00:00 p, m,	17.1	97	0
3/23/2020	8:00:00 p, m,	16.7	96	0
3/23/2020	9:00:00 p, m,	16.8	95	0
3/23/2020	10:00:00 p, m	16.8	93	0
3/23/2020	11:00:00 p, m	16.7	93	0
3/24/2020	12:00:00 a, m	16.7	93	0
3/24/2020	1:00:00 a, m,	17.1	92	0
3/24/2020	2:00:00 a, m,	17.1	92	0
3/24/2020	3:00:00 a, m,	17.1	94	0

3/24/2020	4:00:00 a, m,	16.8	94	0
3/24/2020	5:00:00 a, m,	16.8	92	0
3/24/2020	6:00:00 a, m,	16.7	94	0
3/24/2020	7:00:00 a, m,	16.8	94	0
3/24/2020	8:00:00 a, m,	17.2	95	0
3/24/2020	9:00:00 a, m,	17.6	96	0
3/24/2020	10:00:00 a, m	18.2	98	0
3/24/2020	11:00:00 a, m	18.6	96	0
3/24/2020	12:00:00 p, m	18.8	95	0
3/24/2020	1:00:00 p, m,	18.8	98	0
3/24/2020	2:00:00 p, m,	19.3	94	0
3/24/2020	3:00:00 p, m,	18.6	94	0
3/24/2020	4:00:00 p, m,	18.6	97	0
3/24/2020	5:00:00 p, m,	18.6	96	0
3/24/2020	6:00:00 p, m,	18.4	96	0
3/24/2020	7:00:00 p, m,	17.4	95	0
3/24/2020	8:00:00 p, m,	17.6	94	0
3/24/2020	9:00:00 p, m,	17.3	93	0
3/24/2020	10:00:00 p, m	17.4	93	0
3/24/2020	11:00:00 p, m	17.1	93	0
3/25/2020	12:00:00 a, m	16.8	91	0
3/25/2020	1:00:00 a, m,	16.9	92	0
3/25/2020	2:00:00 a, m,	17	91	0
3/25/2020	3:00:00 a, m,	16.9	92	0
3/25/2020	4:00:00 a, m,	16.9	92	0
3/25/2020	5:00:00 a, m,	16.9	93	0
3/25/2020	6:00:00 a, m,	16.9	93	0
3/25/2020	7:00:00 a, m,	16.9	94	0
3/25/2020	8:00:00 a, m,	17.2	94	0.2
3/25/2020	9:00:00 a, m,	17.7	94	0
3/25/2020	10:00:00 a, m	18.4	94	0
3/25/2020	11:00:00 a, m	19.5	94	0
3/25/2020	12:00:00 p, m	19.3	95	0
3/25/2020	1:00:00 p, m,	19.3	94	0
3/25/2020	2:00:00 p, m,	19.4	93	0
3/25/2020	3:00:00 p, m,	19.5	95	0
3/25/2020	4:00:00 p, m,	19.8	92	0
3/25/2020	5:00:00 p, m,	20.3	93	0
3/25/2020	6:00:00 p, m,	19.8	91	0
3/25/2020	7:00:00 p, m,	18.9	92	0
3/25/2020	8:00:00 p, m,	18.7	91	0
3/25/2020	9:00:00 p, m,	18.4	89	0
3/25/2020	10:00:00 p, m	18.7	90	0
3/25/2020	11:00:00 p, m	18.3	91	0
3/26/2020	12:00:00 a, m	18.6	91	0
3/26/2020	1:00:00 a, m,	18.5	90	0
3/26/2020	2:00:00 a, m,	18.6	90	0

3/26/2020	3:00:00 a, m,	18.3	92	0
3/26/2020	4:00:00 a, m,	17.8	94	0
3/26/2020	5:00:00 a, m,	17.6	95	0
3/26/2020	6:00:00 a, m,	17.4	94	0
3/26/2020	7:00:00 a, m,	17.6	95	0
3/26/2020	8:00:00 a, m,	17.8	97	0
3/26/2020	9:00:00 a, m,	17.8	98	0
3/26/2020	10:00:00 a, m	18.3	97	0
3/26/2020	11:00:00 a, m	19	94	0
3/26/2020	12:00:00 p, m	19.5	96	0
3/26/2020	1:00:00 p, m,	20.8	92	0
3/26/2020	2:00:00 p, m,	21.9	90	0
3/26/2020	3:00:00 p, m,	20.7	93	0
3/26/2020	4:00:00 p, m,	22.2	89	0
3/26/2020	5:00:00 p, m,	21.2	91	0
3/26/2020	6:00:00 p, m,	20.4	92	0
3/26/2020	7:00:00 p, m,	19.7	93	0
3/26/2020	8:00:00 p, m,	19.3	95	0
3/26/2020	9:00:00 p, m,	18.8	93	0
3/26/2020	10:00:00 p, m	18.7	93	0
3/26/2020	11:00:00 p, m	18.7	92	0
3/27/2020	12:00:00 a, m	18.9	94	0
3/27/2020	1:00:00 a, m,	18.8	94	0
3/27/2020	2:00:00 a, m,	18.4	93	0
3/27/2020	3:00:00 a, m,	17.6	95	4.8
3/27/2020	4:00:00 a, m,	16.9	98	3.2
3/27/2020	5:00:00 a, m,	16.6	98	0
3/27/2020	6:00:00 a, m,	16.3	98	0
3/27/2020	7:00:00 a, m,	16.5	98	0
3/27/2020	8:00:00 a, m,	17.2	98	0
3/27/2020	9:00:00 a, m,	17.8	99	0
3/27/2020	10:00:00 a, m	18.2	99	0
3/27/2020	11:00:00 a, m	18.6	99	0
3/27/2020	12:00:00 p, m	18.7	98	0
3/27/2020	1:00:00 p, m,	19.7	99	0
3/27/2020	2:00:00 p, m,	20.4	97	0
3/27/2020	3:00:00 p, m,	20.4	98	0
3/27/2020	4:00:00 p, m,	20.6	95	0
3/27/2020	5:00:00 p, m,	20.6	95	0
3/27/2020	6:00:00 p, m,	20.3	95	0
3/27/2020	7:00:00 p, m,	18.4	92	0
3/27/2020	8:00:00 p, m,	18.4	94	0
3/27/2020	9:00:00 p, m,	18.2	92	0
3/27/2020	10:00:00 p, m	18.2	92	0
3/27/2020	11:00:00 p, m	18.4	92	0
3/28/2020	12:00:00 a, m	18.3	93	0
3/28/2020	1:00:00 a, m,	17.7	97	3.6

3/28/2020	2:00:00 a, m,	16.9	98	4.8
3/28/2020	3:00:00 a, m,	16.8	98	1
3/28/2020	4:00:00 a, m,	16.3	98	1.8
3/28/2020	5:00:00 a, m,	16.1	98	1.8
3/28/2020	6:00:00 a, m,	16.2	98	0.4
3/28/2020	7:00:00 a, m,	16.8	99	0
3/28/2020	8:00:00 a, m,	17.4	99	0
3/28/2020	9:00:00 a, m,	17.9	99	0
3/28/2020	10:00:00 a, m	18.8	99	0
3/28/2020	11:00:00 a, m	19.3	99	0
3/28/2020	12:00:00 p, m	19.3	99	0
3/28/2020	1:00:00 p, m,	20.3	94	0
3/28/2020	2:00:00 p, m,	20.9	93	0
3/28/2020	3:00:00 p, m,	21.5	92	0
3/28/2020	4:00:00 p, m,	21.9	92	0
3/28/2020	5:00:00 p, m,	21.9	91	0
3/28/2020	6:00:00 p, m,	20.7	93	0
3/28/2020	7:00:00 p, m,	20	96	0
3/28/2020	8:00:00 p, m,	19.6	94	0
3/28/2020	9:00:00 p, m,	19	92	0
3/28/2020	10:00:00 p, m	18.9	90	0
3/28/2020	11:00:00 p, m	18.4	89	0
3/29/2020	12:00:00 a, m	18.1	89	0
3/29/2020	1:00:00 a, m,	17.9	84	0
3/29/2020	2:00:00 a, m,	17.3	86	0
3/29/2020	3:00:00 a, m,	17	91	0
3/29/2020	4:00:00 a, m,	16.8	90	0
3/29/2020	5:00:00 a, m,	16.9	93	0
3/29/2020	6:00:00 a, m,	16.9	94	0
3/29/2020	7:00:00 a, m,	17.3	95	0
3/29/2020	8:00:00 a, m,	17.9	94	0
3/29/2020	9:00:00 a, m,	18.4	95	0
3/29/2020	10:00:00 a, m	18.9	95	0
3/29/2020	11:00:00 a, m	19.7	93	0
3/29/2020	12:00:00 p, m	20.2	93	0
3/29/2020	1:00:00 p, m,	21.1	87	0
3/29/2020	2:00:00 p, m,	21	88	0
3/29/2020	3:00:00 p, m,	21.5	90	0
3/29/2020	4:00:00 p, m,	22.7	84	0
3/29/2020	5:00:00 p, m,	21.5	89	0.4
3/29/2020	6:00:00 p, m,	20.8	91	0.4
3/29/2020	7:00:00 p, m,	19.7	85	0
3/29/2020	8:00:00 p, m,	19.5	81	0
3/29/2020	9:00:00 p, m,	19.4	78	0
3/29/2020	10:00:00 p, m	19.3	78	0
3/29/2020	11:00:00 p, m	18.9	79	0
3/30/2020	12:00:00 a, m	18.9	75	0

3/30/2020	1:00:00 a, m,	18.7	83	0
3/30/2020	2:00:00 a, m,	18.7	86	0
3/30/2020	3:00:00 a, m,	18.3	88	0
3/30/2020	4:00:00 a, m,	18.4	91	0
3/30/2020	5:00:00 a, m,	18.2	86	0
3/30/2020	6:00:00 a, m,	18.2	89	0
3/30/2020	7:00:00 a, m,	18.3	89	0
3/30/2020	8:00:00 a, m,	18.6	92	0
3/30/2020	9:00:00 a, m,	19	92	0
3/30/2020	10:00:00 a, m	19.7	92	0
3/30/2020	11:00:00 a, m	19.6	91	0
3/30/2020	12:00:00 p, m	19.3	94	0
3/30/2020	1:00:00 p, m,	18.7	95	0.4
3/30/2020	2:00:00 p, m,	17.8	95	1
3/30/2020	3:00:00 p, m,	18.1	98	0.2
3/30/2020	4:00:00 p, m,	17.8	98	0
3/30/2020	5:00:00 p, m,	17.6	97	0
3/30/2020	6:00:00 p, m,	17	97	0
3/30/2020	7:00:00 p, m,	16.7	97	0
3/30/2020	8:00:00 p, m,	16.9	97	0
3/30/2020	9:00:00 p, m,	17	95	0
3/30/2020	10:00:00 p, m	17	95	0
3/30/2020	11:00:00 p, m	17.1	94	0
3/31/2020	12:00:00 a, m	16.9	93	0
3/31/2020	1:00:00 a, m,	17	92	0
3/31/2020	2:00:00 a, m,	17.4	91	0
3/31/2020	3:00:00 a, m,	17.5	91	0
3/31/2020	4:00:00 a, m,	17.3	91	0
3/31/2020	5:00:00 a, m,	17.2	90	0
3/31/2020	6:00:00 a, m,	17.2	91	0
3/31/2020	7:00:00 a, m,	17.3	91	0
3/31/2020	8:00:00 a, m,	17.8	92	0
3/31/2020	9:00:00 a, m,	18.1	92	0
3/31/2020	10:00:00 a, m	18.4	94	0
3/31/2020	11:00:00 a, m	18.8	92	0
3/31/2020	12:00:00 p, m	18.4	95	0
3/31/2020	1:00:00 p, m,	18.2	98	0.2
3/31/2020	2:00:00 p, m,	18.3	98	0
3/31/2020	3:00:00 p, m,	18.4	98	0
3/31/2020	4:00:00 p, m,	19.3	96	0
3/31/2020	5:00:00 p, m,	19.5	96	0
3/31/2020	6:00:00 p, m,	19.2	97	0
3/31/2020	7:00:00 p, m,	18.4	96	0
3/31/2020	8:00:00 p, m,	17.9	94	0
3/31/2020	9:00:00 p, m,	17.8	92	0
3/31/2020	10:00:00 p, m	17.9	92	0
3/31/2020	11:00:00 p, m	18.2	92	0

Mar_20	18.7820996	90.8600269	164.6
4/1/2020 12:00:00 a, m	18.2	89	0
4/1/2020 1:00:00 a, m,	18	89	0
4/1/2020 2:00:00 a, m,	17.9	88	0
4/1/2020 3:00:00 a, m,	18.3	85	0
4/1/2020 4:00:00 a, m,	18.1	87	0
4/1/2020 5:00:00 a, m,	17.9	87	0
4/1/2020 6:00:00 a, m,	17.7	88	0
4/1/2020 7:00:00 a, m,	17.7	91	0
4/1/2020 8:00:00 a, m,	17.7	90	0
4/1/2020 9:00:00 a, m,	18.1	94	0
4/1/2020 10:00:00 a, m	18.8	95	0
4/1/2020 11:00:00 a, m	18.8	94	0
4/1/2020 12:00:00 p, m	19.3	96	0
4/1/2020 1:00:00 p, m,	19.2	96	0
4/1/2020 2:00:00 p, m,	19.4	96	0
4/1/2020 3:00:00 p, m,	20.9	84	0
4/1/2020 4:00:00 p, m,	20.6	89	0
4/1/2020 5:00:00 p, m,	19.2	94	0.6
4/1/2020 6:00:00 p, m,	18.8	97	0.2
4/1/2020 7:00:00 p, m,	18.2	97	0
4/1/2020 8:00:00 p, m,	17.9	97	0
4/1/2020 9:00:00 p, m,	17.8	97	0
4/1/2020 10:00:00 p, m	17.8	97	0
4/1/2020 11:00:00 p, m	17.8	96	0
4/2/2020 12:00:00 a, m	17.9	96	0
4/2/2020 1:00:00 a, m,	18	96	0
4/2/2020 2:00:00 a, m,	17.9	96	0
4/2/2020 3:00:00 a, m,	17.8	96	0
4/2/2020 4:00:00 a, m,	17.5	96	0
4/2/2020 5:00:00 a, m,	17.6	98	0
4/2/2020 6:00:00 a, m,	17.6	98	0
4/2/2020 7:00:00 a, m,	17.5	98	0
4/2/2020 8:00:00 a, m,	17.7	99	0
4/2/2020 9:00:00 a, m,	17.8	99	0
4/2/2020 10:00:00 a, m	17.6	99	0
4/2/2020 11:00:00 a, m	17.8	99	0
4/2/2020 12:03:00 p, m	18.5	99	0
4/2/2020 1:00:00 p, m,	18.7	99	0
4/2/2020 2:00:00 p, m,	19.6	98	0
4/2/2020 3:00:00 p, m,	19.3	98	0
4/2/2020 4:00:00 p, m,	19.9	98	0
4/2/2020 5:00:00 p, m,	20.1	96	0
4/2/2020 6:00:00 p, m,	19.4	96	0
4/2/2020 7:00:00 p, m,	18.8	94	0
4/2/2020 8:00:00 p, m,	18.2	94	0
4/2/2020 9:00:00 p, m,	17.8	91	0

4/2/2020	10:00:00 p, m	17.6	89	0
4/2/2020	11:00:00 p, m	17.6	88	0
4/3/2020	12:00:00 a, m	17.6	91	0
4/3/2020	1:00:00 a, m,	18.2	85	0
4/3/2020	2:00:00 a, m,	18.3	87	0
4/3/2020	3:00:00 a, m,	18.1	89	0
4/3/2020	4:00:00 a, m,	17.6	89	0
4/3/2020	5:00:00 a, m,	17.6	90	0
4/3/2020	6:00:00 a, m,	17.7	89	0
4/3/2020	7:00:00 a, m,	17.7	90	0
4/3/2020	8:00:00 a, m,	18.1	91	0
4/3/2020	9:00:00 a, m,	18.8	92	0
4/3/2020	10:00:00 a, m	19.3	90	0
4/3/2020	11:00:00 a, m	19.2	92	0
4/3/2020	12:00:00 p, m	19.8	92	0
4/3/2020	1:00:00 p, m,	20.3	86	0
4/3/2020	2:00:00 p, m,	22.7	82	0
4/3/2020	3:00:00 p, m,	22.5	77	0
4/3/2020	4:00:00 p, m,	23.7	74	0
4/3/2020	5:00:00 p, m,	23	72	0
4/3/2020	6:00:00 p, m,	21.8	81	0
4/3/2020	7:00:00 p, m,	20.2	82	0
4/3/2020	8:00:00 p, m,	19.8	77	0
4/3/2020	9:00:00 p, m,	20	76	0
4/3/2020	10:00:00 p, m	20.1	80	0
4/3/2020	11:00:00 p, m	20.2	75	0
4/4/2020	12:00:00 a, m	19.9	77	0
4/4/2020	1:00:00 a, m,	19.6	81	0
4/4/2020	2:00:00 a, m,	19.3	80	0
4/4/2020	3:00:00 a, m,	19.2	82	0
4/4/2020	4:00:00 a, m,	18.9	82	0
4/4/2020	5:00:00 a, m,	18.8	82	0
4/4/2020	6:00:00 a, m,	18.8	83	0
4/4/2020	7:00:00 a, m,	18.9	82	0
4/4/2020	8:00:00 a, m,	19.4	85	0
4/4/2020	9:00:00 a, m,	19.7	85	0
4/4/2020	10:00:00 a, m	20	84	0
4/4/2020	11:00:00 a, m	20.5	83	0
4/4/2020	12:00:00 p, m	21.6	83	0
4/4/2020	1:00:00 p, m,	20.8	87	0
4/4/2020	2:00:00 p, m,	21.9	82	0
4/4/2020	3:00:00 p, m,	22.7	82	0
4/4/2020	4:00:00 p, m,	23.4	75	0
4/4/2020	5:00:00 p, m,	23.3	73	0
4/4/2020	6:00:00 p, m,	21.9	80	0
4/4/2020	7:00:00 p, m,	20.6	81	0
4/4/2020	8:00:00 p, m,	20.2	81	0

4/4/2020	9:00:00 p, m,	20	81	0
4/4/2020	10:00:00 p, m	20.3	81	0
4/4/2020	11:00:00 p, m	20.1	80	0
4/5/2020	12:00:00 a, m	19.9	81	0
4/5/2020	1:00:00 a, m,	20.1	80	0
4/5/2020	2:00:00 a, m,	19.7	80	0
4/5/2020	3:00:00 a, m,	19.4	81	0
4/5/2020	4:00:00 a, m,	19.3	81	0
4/5/2020	5:00:00 a, m,	19.2	83	0
4/5/2020	6:00:00 a, m,	19.1	83	0
4/5/2020	7:00:00 a, m,	18.8	85	0
4/5/2020	8:00:00 a, m,	19.5	84	0
4/5/2020	9:00:00 a, m,	19.4	87	0
4/5/2020	10:00:00 a, m	19.7	88	0
4/5/2020	11:00:00 a, m	19.7	90	0
4/5/2020	12:00:00 p, m	18.9	94	0
4/5/2020	1:00:00 p, m,	18.8	96	0.4
4/5/2020	2:00:00 p, m,	17.4	95	0.4
4/5/2020	3:00:00 p, m,	17.2	97	1.8
4/5/2020	4:00:00 p, m,	17.7	97	0
4/5/2020	5:00:00 p, m,	17.7	96	0
4/5/2020	6:00:00 p, m,	17.2	91	0
4/5/2020	7:00:00 p, m,	17.4	87	0
4/5/2020	8:00:00 p, m,	17.7	85	0
4/5/2020	9:00:00 p, m,	17.7	86	0
4/5/2020	10:00:00 p, m	17.8	85	0
4/5/2020	11:00:00 p, m	17.8	84	0
4/6/2020	12:00:00 a, m	17.7	84	0
4/6/2020	1:00:00 a, m,	17.8	83	0
4/6/2020	2:00:00 a, m,	18.2	82	0
4/6/2020	3:00:00 a, m,	17.9	83	0
4/6/2020	4:00:00 a, m,	18.3	83	0
4/6/2020	5:00:00 a, m,	17.7	84	0
4/6/2020	6:00:00 a, m,	17.7	84	0
4/6/2020	7:00:00 a, m,	18.7	82	0
4/6/2020	8:00:00 a, m,	19.2	84	0
4/6/2020	9:00:00 a, m,	19.5	85	0
4/6/2020	10:00:00 a, m	19.6	81	0.2
4/6/2020	11:00:00 a, m	19.8	84	0
4/6/2020	12:00:00 p, m	20.4	87	0
4/6/2020	1:00:00 p, m,	20.7	86	0
4/6/2020	2:00:00 p, m,	20.8	82	0
4/6/2020	3:00:00 p, m,	21	83	0
4/6/2020	4:00:00 p, m,	22.3	79	0
4/6/2020	5:00:00 p, m,	23.1	81	0
4/6/2020	6:00:00 p, m,	21.8	84	0
4/6/2020	7:00:00 p, m,	20.7	81	0

4/6/2020	8:00:00 p, m,	20.5	77	0
4/6/2020	9:00:00 p, m,	20.6	76	0
4/6/2020	10:00:00 p, m	20.9	72	0
4/6/2020	11:00:00 p, m	20.6	76	0
4/7/2020	12:00:00 a, m	20.3	81	0
4/7/2020	1:00:00 a, m,	19	87	0
4/7/2020	2:00:00 a, m,	18.8	83	0
4/7/2020	3:00:00 a, m,	18.3	85	0
4/7/2020	4:00:00 a, m,	18	83	0
4/7/2020	5:00:00 a, m,	17.6	86	0
4/7/2020	6:00:00 a, m,	17.2	89	0
4/7/2020	7:00:00 a, m,	17.7	93	0
4/7/2020	8:00:00 a, m,	18.3	90	0
4/7/2020	9:00:00 a, m,	19.1	91	0
4/7/2020	10:00:00 a, m	19.3	90	0
4/7/2020	11:00:00 a, m	20.1	90	0
4/7/2020	12:00:00 p, m	20.4	90	0
4/7/2020	1:00:00 p, m,	20.7	92	0
4/7/2020	2:00:00 p, m,	20.6	77	0
4/7/2020	3:00:00 p, m,	20.7	78	0
4/7/2020	4:00:00 p, m,	21	88	0
4/7/2020	5:00:00 p, m,	20.6	82	0
4/7/2020	6:00:00 p, m,	20.2	78	0
4/7/2020	7:00:00 p, m,	19.8	80	0
4/7/2020	8:00:00 p, m,	19.8	78	0
4/7/2020	9:00:00 p, m,	19.6	78	0
4/7/2020	10:00:00 p, m	19.6	79	0
4/7/2020	11:00:00 p, m	20	76	0
4/8/2020	12:00:00 a, m	19.9	75	0
4/8/2020	1:00:00 a, m,	19.4	77	0
4/8/2020	2:00:00 a, m,	18.9	80	0
4/8/2020	3:00:00 a, m,	18.8	81	0
4/8/2020	4:00:00 a, m,	18.7	81	0
4/8/2020	5:00:00 a, m,	18.4	82	0
4/8/2020	6:00:00 a, m,	18.8	82	0
4/8/2020	7:00:00 a, m,	18.9	82	0
4/8/2020	8:00:00 a, m,	19.2	82	0
4/8/2020	9:00:00 a, m,	19.5	85	0
4/8/2020	10:00:00 a, m	19.6	88	0
4/8/2020	11:00:00 a, m	19.6	88	0
4/8/2020	12:00:00 p, m	19.9	87	0
4/8/2020	1:00:00 p, m,	20.1	88	0
4/8/2020	2:00:00 p, m,	19.4	90	0
4/8/2020	3:00:00 p, m,	18.9	94	0
4/8/2020	4:00:00 p, m,	19.1	94	0
4/8/2020	5:00:00 p, m,	18.7	91	0
4/8/2020	6:00:00 p, m,	17.9	92	0.2

4/8/2020	7:00:00 p, m,	17.9	90	0
4/8/2020	8:00:00 p, m,	18.3	87	0
4/8/2020	9:00:00 p, m,	18.9	85	0
4/8/2020	10:00:00 p, m	19.8	80	0
4/8/2020	11:00:00 p, m	19.7	83	0
4/9/2020	12:00:00 a, m	19.6	82	0
4/9/2020	1:00:00 a, m,	19.5	81	0
4/9/2020	2:00:00 a, m,	19.4	82	0
4/9/2020	3:00:00 a, m,	19.4	82	0
4/9/2020	4:00:00 a, m,	19.3	82	0
4/9/2020	5:00:00 a, m,	19.1	84	0
4/9/2020	6:00:00 a, m,	18.8	85	0
4/9/2020	7:00:00 a, m,	18.5	88	0
4/9/2020	8:00:00 a, m,	18.6	88	0
4/9/2020	9:00:00 a, m,	18.9	90	0
4/9/2020	10:00:00 a, m	19.2	90	0
4/9/2020	11:00:00 a, m	20	92	0
4/9/2020	12:00:00 p, m	20.2	89	0
4/9/2020	1:00:00 p, m,	20.4	90	0
4/9/2020	2:00:00 p, m,	21.2	87	0
4/9/2020	3:00:00 p, m,	22	83	0
4/9/2020	4:00:00 p, m,	22.3	83	0
4/9/2020	5:00:00 p, m,	21.6	83	0
4/9/2020	6:00:00 p, m,	20.8	86	0
4/9/2020	7:00:00 p, m,	19.7	85	0
4/9/2020	8:00:00 p, m,	19.6	78	0
4/9/2020	9:00:00 p, m,	19.4	77	0
4/9/2020	10:00:00 p, m	19.3	79	0
4/9/2020	11:00:00 p, m	19.3	79	0
4/10/2020	12:00:00 a, m	19.3	79	0
4/10/2020	1:00:00 a, m,	18.9	80	0
4/10/2020	2:00:00 a, m,	18.6	81	0
4/10/2020	3:00:00 a, m,	19.3	80	0
4/10/2020	4:00:00 a, m,	18.6	81	0
4/10/2020	5:00:00 a, m,	18.9	81	0
4/10/2020	6:00:00 a, m,	19.1	81	0
4/10/2020	7:00:00 a, m,	19	83	0
4/10/2020	8:00:00 a, m,	19.5	82	0
4/10/2020	9:00:00 a, m,	19.6	85	0
4/10/2020	10:00:00 a, m	20.1	85	0
4/10/2020	11:00:00 a, m	20.2	85	0
4/10/2020	12:00:00 p, m	20.3	85	0
4/10/2020	1:00:00 p, m,	20.4	87	0
4/10/2020	2:00:00 p, m,	22	78	0
4/10/2020	3:00:00 p, m,	22.9	79	0
4/10/2020	4:00:00 p, m,	23.2	81	0
4/10/2020	5:00:00 p, m,	22.6	78	0

4/10/2020	6:00:00 p, m,	20.9	83	0
4/10/2020	7:00:00 p, m,	20.6	82	0
4/10/2020	8:00:00 p, m,	20.1	83	0
4/10/2020	9:00:00 p, m,	19.8	83	0
4/10/2020	10:00:00 p, m	19.5	83	0
4/10/2020	11:00:00 p, m	19.6	83	0
4/11/2020	12:00:00 a, m	19.7	81	0
4/11/2020	1:00:00 a, m,	19.6	81	0
4/11/2020	2:00:00 a, m,	19.7	80	0
4/11/2020	3:00:00 a, m,	19.8	81	0
4/11/2020	4:00:00 a, m,	19.7	80	0
4/11/2020	5:00:00 a, m,	19.6	81	0
4/11/2020	6:00:00 a, m,	19.3	81	0
4/11/2020	7:00:00 a, m,	19.4	82	0
4/11/2020	8:00:00 a, m,	19.7	79	0
4/11/2020	9:00:00 a, m,	20.3	85	0
4/11/2020	10:00:00 a, m	20.6	85	0
4/11/2020	11:00:00 a, m	21	84	0
4/11/2020	12:00:00 p, m	21.3	84	0
4/11/2020	1:00:00 p, m,	21.1	82	0
4/11/2020	2:00:00 p, m,	21.2	84	0
4/11/2020	3:00:00 p, m,	22	83	0
4/11/2020	4:00:00 p, m,	22.1	83	0
4/11/2020	5:00:00 p, m,	21.9	79	0
4/11/2020	6:00:00 p, m,	21.3	79	0
4/11/2020	7:00:00 p, m,	20.7	77	0
4/11/2020	8:00:00 p, m,	20.7	77	0
4/11/2020	9:00:00 p, m,	20.4	79	0
4/11/2020	10:00:00 p, m	20.6	77	0
4/11/2020	11:00:00 p, m	20.2	81	0
4/12/2020	12:00:00 a, m	19.8	81	0
4/12/2020	1:00:00 a, m,	19.6	80	0
4/12/2020	2:00:00 a, m,	19.7	81	0
4/12/2020	3:00:00 a, m,	19.4	81	0
4/12/2020	4:00:00 a, m,	19.3	83	0
4/12/2020	5:00:00 a, m,	19.1	85	0
4/12/2020	6:00:00 a, m,	19.1	84	0
4/12/2020	7:00:00 a, m,	19	88	0
4/12/2020	8:00:00 a, m,	19.3	89	0
4/12/2020	9:00:00 a, m,	19.5	90	0
4/12/2020	10:00:00 a, m	19.8	92	0
4/12/2020	11:00:00 a, m	19.5	93	0
4/12/2020	12:00:00 p, m	19.6	89	0
4/12/2020	1:00:00 p, m,	19.8	85	0
4/12/2020	2:00:00 p, m,	19.7	94	0
4/12/2020	3:00:00 p, m,	20.3	88	0
4/12/2020	4:00:00 p, m,	21.6	89	0

4/12/2020	5:00:00 p, m,	20.8	89	0
4/12/2020	6:00:00 p, m,	20.4	89	0
4/12/2020	7:00:00 p, m,	18.9	89	0
4/12/2020	8:00:00 p, m,	18.1	78	0
4/12/2020	9:00:00 p, m,	18.2	73	0
4/12/2020	10:00:00 p, m	18.8	75	0
4/12/2020	11:00:00 p, m	18.7	73	0
4/13/2020	12:00:00 a, m	18.7	76	0
4/13/2020	1:00:00 a, m,	18.4	76	0
4/13/2020	2:00:00 a, m,	18.2	77	0
4/13/2020	3:00:00 a, m,	18.4	75	0
4/13/2020	4:00:00 a, m,	18.2	76	0
4/13/2020	5:00:00 a, m,	17.8	76	0
4/13/2020	6:00:00 a, m,	17.6	78	0
4/13/2020	7:00:00 a, m,	17.7	81	0
4/13/2020	8:00:00 a, m,	18.2	81	0
4/13/2020	9:00:00 a, m,	18.7	83	0
4/13/2020	10:00:00 a, m	19.5	84	0
4/13/2020	11:00:00 a, m	21.6	83	0
4/13/2020	12:00:00 p, m	21.7	81	0
4/13/2020	1:00:00 p, m,	21.6	81	0
4/13/2020	2:00:00 p, m,	24	71	0
4/13/2020	3:00:00 p, m,	24.3	67	0
4/13/2020	4:00:00 p, m,	24.3	67	0
4/13/2020	5:00:00 p, m,	23.8	70	0
4/13/2020	6:00:00 p, m,	22.4	76	0
4/13/2020	7:00:00 p, m,	21.3	78	0
4/13/2020	8:00:00 p, m,	21.1	78	0
4/13/2020	9:00:00 p, m,	20.5	80	0
4/13/2020	10:00:00 p, m	20.2	82	0
4/13/2020	11:00:00 p, m	20.1	81	0
4/14/2020	12:00:00 a, m	19.8	85	0
4/14/2020	1:00:00 a, m,	19.4	87	0
4/14/2020	2:00:00 a, m,	19.1	90	0
4/14/2020	3:00:00 a, m,	18.7	88	0
4/14/2020	4:00:00 a, m,	18.6	93	0
4/14/2020	5:00:00 a, m,	17.1	94	9.4
4/14/2020	6:00:00 a, m,	16.7	97	8
4/14/2020	7:00:00 a, m,	16.7	97	1.4
4/14/2020	8:00:00 a, m,	17.1	98	1.2
4/14/2020	9:00:00 a, m,	17.6	98	0
4/14/2020	10:00:00 a, m	18.2	98	0
4/14/2020	11:00:00 a, m	18.5	98	0.2
4/14/2020	12:00:00 p, m	18.8	98	0
4/14/2020	1:00:00 p, m,	18.7	98	0
4/14/2020	2:00:00 p, m,	19.2	99	0
4/14/2020	3:00:00 p, m,	19.5	99	0

4/14/2020	4:00:00 p, m,	19.4	98	0
4/14/2020	5:00:00 p, m,	19.6	98	0
4/14/2020	6:00:00 p, m,	19.3	97	0
4/14/2020	7:00:00 p, m,	18.6	97	0
4/14/2020	8:00:00 p, m,	18.1	97	0
4/14/2020	9:00:00 p, m,	17.8	96	0
4/14/2020	10:00:00 p, m	17.8	95	0
4/14/2020	11:00:00 p, m	17.7	93	0
4/15/2020	12:00:00 a, m	17.7	96	1.6
4/15/2020	1:00:00 a, m,	17.3	98	0.4
4/15/2020	2:00:00 a, m,	17.3	97	0.2
4/15/2020	3:00:00 a, m,	17.2	98	0
4/15/2020	4:00:00 a, m,	17.3	97	0
4/15/2020	5:00:00 a, m,	17.3	97	0
4/15/2020	6:00:00 a, m,	17.1	97	0
4/15/2020	7:00:00 a, m,	17.2	97	0
4/15/2020	8:00:00 a, m,	17.7	98	0
4/15/2020	9:00:00 a, m,	18.1	98	0
4/15/2020	10:00:00 a, m	18.1	97	0
4/15/2020	11:00:00 a, m	18.6	99	0
4/15/2020	12:00:00 p, m	19.3	99	0
4/15/2020	1:00:00 p, m,	19.7	97	0
4/15/2020	2:00:00 p, m,	19.8	96	0
4/15/2020	3:00:00 p, m,	19.9	95	0
4/15/2020	4:00:00 p, m,	19.8	96	0
4/15/2020	5:00:00 p, m,	19.6	97	0
4/15/2020	6:00:00 p, m,	19	96	0
4/15/2020	7:00:00 p, m,	18.8	97	0
4/15/2020	8:00:00 p, m,	18.6	96	0
4/15/2020	9:00:00 p, m,	18.3	96	0
4/15/2020	10:00:00 p, m	18.3	95	0
4/15/2020	11:00:00 p, m	18.2	94	0
4/16/2020	12:00:00 a, m	17.3	97	9.4
4/16/2020	1:00:00 a, m,	16.2	97	1.6
4/16/2020	2:00:00 a, m,	16.2	98	0.2
4/16/2020	3:00:00 a, m,	15.9	97	0.2
4/16/2020	4:00:00 a, m,	16.1	98	0
4/16/2020	5:00:00 a, m,	16.1	97	0
4/16/2020	6:00:00 a, m,	16.3	97	0
4/16/2020	7:00:00 a, m,	16.4	98	0
4/16/2020	8:00:00 a, m,	16.4	98	0.4
4/16/2020	9:00:00 a, m,	16.8	99	0.6
4/16/2020	10:00:00 a, m	17.5	99	0
4/16/2020	11:00:00 a, m	18.5	99	0
4/16/2020	12:00:00 p, m	18.6	99	0
4/16/2020	1:00:00 p, m,	17.9	97	0
4/16/2020	2:00:00 p, m,	18.6	99	0.2

4/16/2020	3:00:00 p, m,	18.8	98	0
4/16/2020	4:00:00 p, m,	19.3	95	0
4/16/2020	5:00:00 p, m,	19.2	96	0
4/16/2020	6:00:00 p, m,	18.9	97	0
4/16/2020	7:00:00 p, m,	18.6	97	0
4/16/2020	8:00:00 p, m,	17.8	96	0
4/16/2020	9:00:00 p, m,	17.3	92	0
4/16/2020	10:00:00 p, m	17.5	89	0
4/16/2020	11:00:00 p, m	17.2	89	0
4/17/2020	12:00:00 a, m	17.1	87	0
4/17/2020	1:00:00 a, m,	17.4	85	0
4/17/2020	2:00:00 a, m,	17.3	88	0
4/17/2020	3:00:00 a, m,	17.3	91	0
4/17/2020	4:00:00 a, m,	17	90	0
4/17/2020	5:00:00 a, m,	16.9	93	0
4/17/2020	6:00:00 a, m,	16.9	92	0
4/17/2020	7:00:00 a, m,	17.3	91	0
4/17/2020	8:00:00 a, m,	17.9	91	0
4/17/2020	9:00:00 a, m,	18.4	91	0
4/17/2020	10:00:00 a, m	18.5	93	0
4/17/2020	11:00:00 a, m	18.9	91	0
4/17/2020	12:00:00 p, m	18.9	92	0
4/17/2020	1:00:00 p, m,	20.7	86	0
4/17/2020	2:00:00 p, m,	20.7	86	0
4/17/2020	3:00:00 p, m,	20.1	88	0
4/17/2020	4:00:00 p, m,	19.9	88	0
4/17/2020	5:00:00 p, m,	20.4	83	0
4/17/2020	6:00:00 p, m,	19.7	87	0
4/17/2020	7:00:00 p, m,	18.7	91	0
4/17/2020	8:00:00 p, m,	18.4	89	0
4/17/2020	9:00:00 p, m,	18.6	86	0
4/17/2020	10:00:00 p, m	18.9	82	0
4/17/2020	11:00:00 p, m	18.8	84	0
4/18/2020	12:00:00 a, m	18.5	87	0
4/18/2020	1:00:00 a, m,	18.3	87	0
4/18/2020	2:00:00 a, m,	18.2	86	0
4/18/2020	3:00:00 a, m,	17.9	86	0
4/18/2020	4:00:00 a, m,	17.1	91	0
4/18/2020	5:00:00 a, m,	17.3	90	0
4/18/2020	6:00:00 a, m,	17.2	89	0
4/18/2020	7:00:00 a, m,	17.2	90	0
4/18/2020	8:00:00 a, m,	17.4	92	0
4/18/2020	9:00:00 a, m,	17.7	92	0
4/18/2020	10:00:00 a, m	17.9	91	0
4/18/2020	11:00:00 a, m	18.1	92	0
4/18/2020	12:00:00 p, m	18.1	90	0
4/18/2020	1:00:00 p, m,	18.5	92	0

4/18/2020	2:00:00 p, m,	19.3	91	0
4/18/2020	3:00:00 p, m,	19.8	91	0
4/18/2020	4:00:00 p, m,	21.1	85	0
4/18/2020	5:00:00 p, m,	19.4	89	0
4/18/2020	6:00:00 p, m,	18.8	88	0
4/18/2020	7:00:00 p, m,	18.7	84	0
4/18/2020	8:00:00 p, m,	18.5	81	0
4/18/2020	9:00:00 p, m,	18.3	86	0
4/18/2020	10:00:00 p, m	18.2	85	0
4/18/2020	11:00:00 p, m	18.3	82	0
4/19/2020	12:00:00 a, m	18.2	82	0
4/19/2020	1:00:00 a, m,	17.9	82	0
4/19/2020	2:00:00 a, m,	17.7	83	0
4/19/2020	3:00:00 a, m,	17.7	83	0
4/19/2020	4:00:00 a, m,	17.7	84	0
4/19/2020	5:00:00 a, m,	17.3	84	0
4/19/2020	6:00:00 a, m,	16.9	86	0
4/19/2020	7:00:00 a, m,	17.5	85	0
4/19/2020	8:00:00 a, m,	18	87	0
4/19/2020	9:00:00 a, m,	18.3	86	0
4/19/2020	10:00:00 a, m	18.9	87	0
4/19/2020	11:00:00 a, m	19.3	89	0
4/19/2020	12:00:00 p, m	19.3	88	0
4/19/2020	1:00:00 p, m,	20.1	84	0
4/19/2020	2:00:00 p, m,	21.3	84	0
4/19/2020	3:00:00 p, m,	20.3	87	0
4/19/2020	4:00:00 p, m,	21.2	82	0.4
4/19/2020	5:00:00 p, m,	22.9	71	0
4/19/2020	6:00:00 p, m,	21.1	79	0
4/19/2020	7:00:00 p, m,	19.4	82	0
4/19/2020	8:00:00 p, m,	19.2	78	0
4/19/2020	9:00:00 p, m,	18.9	78	0
4/19/2020	10:00:00 p, m	18.4	78	0
4/19/2020	11:00:00 p, m	18.2	79	0
4/20/2020	12:00:00 a, m	18.1	80	0
4/20/2020	1:00:00 a, m,	18.1	78	0
4/20/2020	2:00:00 a, m,	17.4	80	0
4/20/2020	3:00:00 a, m,	17.6	80	0
4/20/2020	4:00:00 a, m,	17.1	82	0
4/20/2020	5:00:00 a, m,	16.8	84	0
4/20/2020	6:00:00 a, m,	17.1	82	0
4/20/2020	7:00:00 a, m,	17	80	0
4/20/2020	8:00:00 a, m,	17.2	83	0
4/20/2020	9:00:00 a, m,	18.1	84	0
4/20/2020	10:00:00 a, m	19.6	83	0
4/20/2020	11:00:00 a, m	19.7	86	0
4/20/2020	12:00:00 p, m	20.8	81	0

4/20/2020	1:00:00 p, m,	20.4	82	0
4/20/2020	2:00:00 p, m,	21.4	81	0
4/20/2020	3:00:00 p, m,	22.5	71	0
4/20/2020	4:00:00 p, m,	23.7	68	0
4/20/2020	5:00:00 p, m,	20.7	81	0
4/20/2020	6:00:00 p, m,	20.9	71	0
4/20/2020	7:00:00 p, m,	20.2	72	0
4/20/2020	8:00:00 p, m,	20.4	71	0
4/20/2020	9:00:00 p, m,	20.4	71	0
4/20/2020	10:00:00 p, m	20.4	71	0
4/20/2020	11:00:00 p, m	19.9	75	0
4/21/2020	12:00:00 a, m	19.2	77	0
4/21/2020	1:00:00 a, m,	18.8	78	0
4/21/2020	2:00:00 a, m,	18.5	77	0
4/21/2020	3:00:00 a, m,	18.2	77	0
4/21/2020	4:00:00 a, m,	18.4	75	0
4/21/2020	5:00:00 a, m,	18.4	76	0
4/21/2020	6:00:00 a, m,	17.7	77	0
4/21/2020	7:00:00 a, m,	17.8	79	0
4/21/2020	8:00:00 a, m,	18.5	81	0
4/21/2020	9:00:00 a, m,	18.8	83	0
4/21/2020	10:00:00 a, m	19.4	81	0
4/21/2020	11:00:00 a, m	20.6	76	0
4/21/2020	12:00:00 p, m	20.6	82	0
4/21/2020	1:00:00 p, m,	21.1	81	0
4/21/2020	2:00:00 p, m,	23.2	67	0
4/21/2020	3:00:00 p, m,	23.9	67	0
4/21/2020	4:00:00 p, m,	23.2	75	0
4/21/2020	5:00:00 p, m,	23.9	63	0
4/21/2020	6:00:00 p, m,	22.2	75	0
4/21/2020	7:00:00 p, m,	21	75	0
4/21/2020	8:00:00 p, m,	20.3	76	0
4/21/2020	9:00:00 p, m,	19.9	78	0
4/21/2020	10:00:00 p, m	19.3	81	0
4/21/2020	11:00:00 p, m	19.1	81	0
4/22/2020	12:00:00 a, m	18.8	83	0
4/22/2020	1:00:00 a, m,	18.4	84	0
4/22/2020	2:00:00 a, m,	18.1	86	0
4/22/2020	3:00:00 a, m,	17.8	86	0
4/22/2020	4:00:00 a, m,	17.4	88	0
4/22/2020	5:00:00 a, m,	17.3	89	0
4/22/2020	6:00:00 a, m,	17	88	0
4/22/2020	7:00:00 a, m,	17.1	89	0
4/22/2020	8:00:00 a, m,	17.6	90	0
4/22/2020	9:00:00 a, m,	18.2	91	0
4/22/2020	10:00:00 a, m	19.4	89	0
4/22/2020	11:00:00 a, m	20.7	87	0

4/22/2020	12:00:00 p, m	20	90	0
4/22/2020	1:00:00 p, m,	19.3	93	0
4/22/2020	2:00:00 p, m,	18.6	95	2.6
4/22/2020	3:00:00 p, m,	18.6	96	0
4/22/2020	4:00:00 p, m,	19.6	97	0
4/22/2020	5:00:00 p, m,	18.6	95	0
4/22/2020	6:00:00 p, m,	18.4	92	0
4/22/2020	7:00:00 p, m,	17.9	91	0
4/22/2020	8:00:00 p, m,	17.8	89	0
4/22/2020	9:00:00 p, m,	17.7	90	0
4/22/2020	10:00:00 p, m	17.6	88	0
4/22/2020	11:00:00 p, m	17.6	90	0
4/23/2020	12:00:00 a, m	17.6	89	0
4/23/2020	1:00:00 a, m,	17.7	88	0
4/23/2020	2:00:00 a, m,	17.6	90	0
4/23/2020	3:00:00 a, m,	17.5	90	0
4/23/2020	4:00:00 a, m,	17.4	90	0
4/23/2020	5:00:00 a, m,	17.4	91	0
4/23/2020	6:00:00 a, m,	17.2	90	0
4/23/2020	7:00:00 a, m,	17.2	92	0
4/23/2020	8:00:00 a, m,	17.7	91	0
4/23/2020	9:00:00 a, m,	17.9	92	0
4/23/2020	10:00:00 a, m	18.7	92	0
4/23/2020	11:00:00 a, m	19.8	93	0
4/23/2020	12:00:00 p, m	20.4	87	0
4/23/2020	1:00:00 p, m,	21	91	0
4/23/2020	2:00:00 p, m,	21.4	82	0
4/23/2020	3:00:00 p, m,	21	87	0
4/23/2020	4:00:00 p, m,	20.8	86	0
4/23/2020	5:00:00 p, m,	20.8	86	0
4/23/2020	6:00:00 p, m,	20.4	83	0
4/23/2020	7:00:00 p, m,	20.2	83	0
4/23/2020	8:00:00 p, m,	20.3	81	0
4/23/2020	9:00:00 p, m,	19.9	81	0
4/23/2020	10:00:00 p, m	19.8	84	0
4/23/2020	11:00:00 p, m	19.2	83	0
4/24/2020	12:00:00 a, m	19.3	84	0
4/24/2020	1:00:00 a, m,	18.7	87	0
4/24/2020	2:00:00 a, m,	18.7	88	0
4/24/2020	3:00:00 a, m,	18.5	87	0
4/24/2020	4:00:00 a, m,	18.1	88	0
4/24/2020	5:00:00 a, m,	17.8	88	0
4/24/2020	6:00:00 a, m,	17.6	89	0
4/24/2020	7:00:00 a, m,	18	88	0
4/24/2020	8:00:00 a, m,	18.4	91	0
4/24/2020	9:00:00 a, m,	18.7	90	0
4/24/2020	10:00:00 a, m	19.2	92	0

4/24/2020	11:00:00 a, m	20.2	89	0
4/24/2020	12:00:00 p, m	19.3	90	0
4/24/2020	1:00:00 p, m,	18.4	96	0.2
4/24/2020	2:00:00 p, m,	18.4	96	1
4/24/2020	3:00:00 p, m,	18.8	95	0
4/24/2020	4:00:00 p, m,	19.3	93	0
4/24/2020	5:00:00 p, m,	19.9	89	0
4/24/2020	6:00:00 p, m,	19.3	90	0
4/24/2020	7:00:00 p, m,	18.7	90	0
4/24/2020	8:00:00 p, m,	18.7	90	0
4/24/2020	9:00:00 p, m,	18.4	90	0
4/24/2020	10:00:00 p, m	18.3	90	0
4/24/2020	11:00:00 p, m	18.3	91	0
4/25/2020	12:00:00 a, m	18.4	93	0
4/25/2020	1:00:00 a, m,	18.2	91	0
4/25/2020	2:00:00 a, m,	17.6	91	0
4/25/2020	3:00:00 a, m,	17.4	92	0
4/25/2020	4:00:00 a, m,	17.5	93	0
4/25/2020	5:00:00 a, m,	17.4	91	0
4/25/2020	6:00:00 a, m,	17.3	91	0
4/25/2020	7:00:00 a, m,	17.7	90	0
4/25/2020	8:00:00 a, m,	17.8	92	0
4/25/2020	9:00:00 a, m,	18.4	94	0
4/25/2020	10:00:00 a, m	19.3	93	0
4/25/2020	11:00:00 a, m	19.8	93	0
4/25/2020	12:00:00 p, m	21.4	89	0
4/25/2020	1:00:00 p, m,	22.9	84	0
4/25/2020	2:00:00 p, m,	23.5	81	0
4/25/2020	3:00:00 p, m,	23.7	79	0
4/25/2020	4:00:00 p, m,	23.2	81	0
4/25/2020	5:00:00 p, m,	23.1	75	0
4/25/2020	6:00:00 p, m,	21.3	82	0
4/25/2020	7:00:00 p, m,	20.2	85	0
4/25/2020	8:00:00 p, m,	19.7	84	0
4/25/2020	9:00:00 p, m,	19.3	81	0
4/25/2020	10:00:00 p, m	19.2	83	0
4/25/2020	11:00:00 p, m	19.2	84	0
4/26/2020	12:00:00 a, m	18.9	82	0
4/26/2020	1:00:00 a, m,	18.4	83	0
4/26/2020	2:00:00 a, m,	17.8	84	0
4/26/2020	3:00:00 a, m,	17.7	88	0
4/26/2020	4:00:00 a, m,	17.8	87	0
4/26/2020	5:00:00 a, m,	16.9	82	0
4/26/2020	6:00:00 a, m,	17.5	89	0
4/26/2020	7:00:00 a, m,	17.4	86	0
4/26/2020	8:00:00 a, m,	17.9	89	0
4/26/2020	9:00:00 a, m,	18.6	89	0

4/26/2020	10:00:00 a, m	20	85	0
4/26/2020	11:00:00 a, m	21.2	83	0
4/26/2020	12:00:00 p, m	21.5	75	0
4/26/2020	1:00:00 p, m,	21.3	79	0
4/26/2020	2:00:00 p, m,	21.6	82	0
4/26/2020	3:00:00 p, m,	21.9	73	0
4/26/2020	4:00:00 p, m,	23.3	72	0
4/26/2020	5:00:00 p, m,	22.8	70	0
4/26/2020	6:00:00 p, m,	22.1	70	0
4/26/2020	7:00:00 p, m,	21.4	70	0
4/26/2020	8:00:00 p, m,	21.2	71	0
4/26/2020	9:00:00 p, m,	21.6	68	0
4/26/2020	10:00:00 p, m	18.8	84	0
4/26/2020	11:00:00 p, m	18.6	84	0
4/27/2020	12:00:00 a, m	18.2	84	0
4/27/2020	1:00:00 a, m,	19	87	0
4/27/2020	2:00:00 a, m,	18.2	85	0
4/27/2020	3:00:00 a, m,	17.9	87	0
4/27/2020	4:00:00 a, m,	17.9	88	0
4/27/2020	5:00:00 a, m,	17.9	87	0
4/27/2020	6:00:00 a, m,	18	85	0
4/27/2020	7:00:00 a, m,	18.4	86	0
4/27/2020	8:00:00 a, m,	19	87	0
4/27/2020	9:00:00 a, m,	19.3	86	0
4/27/2020	10:00:00 a, m	19.8	85	0
4/27/2020	11:00:00 a, m	20.3	84	0
4/27/2020	12:00:00 p, m	21.4	74	0
4/27/2020	1:00:00 p, m,	21	78	0
4/27/2020	2:00:00 p, m,	21.4	81	0
4/27/2020	3:00:00 p, m,	23.1	76	0
4/27/2020	4:00:00 p, m,	22.1	80	0
4/27/2020	5:00:00 p, m,	22.6	76	0
4/27/2020	6:00:00 p, m,	21.4	80	0
4/27/2020	7:00:00 p, m,	20.2	79	0
4/27/2020	8:00:00 p, m,	20.4	76	0
4/27/2020	9:00:00 p, m,	20.4	74	0
4/27/2020	10:00:00 p, m	20.3	72	0
4/27/2020	11:00:00 p, m	20.2	75	0
4/28/2020	12:00:00 a, m	18.8	82	0
4/28/2020	1:00:00 a, m,	19.2	79	0
4/28/2020	2:00:00 a, m,	19.6	76	0
4/28/2020	3:00:00 a, m,	19.1	80	0
4/28/2020	4:00:00 a, m,	18.5	81	0
4/28/2020	5:00:00 a, m,	17.6	87	0
4/28/2020	6:00:00 a, m,	16.8	90	0
4/28/2020	7:00:00 a, m,	17.3	89	0
4/28/2020	8:00:00 a, m,	17.7	89	0

4/28/2020	9:00:00 a, m,	18.3	89	0
4/28/2020	10:00:00 a, m	19.2	88	0
4/28/2020	11:00:00 a, m	19.9	89	0
4/28/2020	12:00:00 p, m	21.3	89	0
4/28/2020	1:00:00 p, m,	22.8	81	0
4/28/2020	2:00:00 p, m,	21.3	82	0
4/28/2020	3:00:00 p, m,	21.3	83	0
4/28/2020	4:00:00 p, m,	22.1	78	0
4/28/2020	5:00:00 p, m,	21.3	83	0
4/28/2020	6:00:00 p, m,	20.7	84	0
4/28/2020	7:00:00 p, m,	20.4	84	0
4/28/2020	8:00:00 p, m,	20.3	80	0
4/28/2020	9:00:00 p, m,	19.3	86	0
4/28/2020	10:00:00 p, m	19.4	81	0
4/28/2020	11:00:00 p, m	19.4	83	0
4/29/2020	12:00:00 a, m	19.1	81	0
4/29/2020	1:00:00 a, m,	18.4	83	0
4/29/2020	2:00:00 a, m,	18.2	82	0
4/29/2020	3:00:00 a, m,	18.1	81	0
4/29/2020	4:00:00 a, m,	18.1	82	0
4/29/2020	5:00:00 a, m,	17.9	83	0
4/29/2020	6:00:00 a, m,	18	83	0
4/29/2020	7:00:00 a, m,	17.9	84	0
4/29/2020	8:00:00 a, m,	18.7	85	0
4/29/2020	9:00:00 a, m,	18.9	85	0
4/29/2020	10:00:00 a, m	19.7	88	0
4/29/2020	11:00:00 a, m	20.2	90	0
4/29/2020	12:00:00 p, m	22.2	84	0
4/29/2020	1:00:00 p, m,	21.2	85	0
4/29/2020	2:00:00 p, m,	22.4	81	0
4/29/2020	3:00:00 p, m,	21.9	81	0
4/29/2020	4:00:00 p, m,	19.7	90	1.6
4/29/2020	5:00:00 p, m,	18.7	95	0.2
4/29/2020	6:00:00 p, m,	18.7	89	0
4/29/2020	7:00:00 p, m,	18.8	88	0
4/29/2020	8:00:00 p, m,	18.7	87	0
4/29/2020	9:00:00 p, m,	19.2	83	0
4/29/2020	10:00:00 p, m	19.2	83	0
4/29/2020	11:00:00 p, m	19.2	85	0
4/30/2020	12:00:00 a, m	18.9	86	0
4/30/2020	1:00:00 a, m,	18.8	84	0
4/30/2020	2:00:00 a, m,	18.7	82	0
4/30/2020	3:00:00 a, m,	18.8	85	0
4/30/2020	4:00:00 a, m,	18.8	85	0
4/30/2020	5:00:00 a, m,	18.7	86	0
4/30/2020	6:00:00 a, m,	18	87	0
4/30/2020	7:00:00 a, m,	18.2	87	0

4/30/2020	8:00:00 a, m,	18.6	88	0
4/30/2020	9:00:00 a, m,	19.4	90	0
4/30/2020	10:00:00 a, m	19.8	89	0
4/30/2020	11:00:00 a, m	20.3	88	0
4/30/2020	12:00:00 p, m	20.9	88	0
4/30/2020	1:00:00 p, m,	21.5	83	0
4/30/2020	2:00:00 p, m,	21.7	85	0
4/30/2020	3:00:00 p, m,	22.4	80	0
4/30/2020	4:00:00 p, m,	25	68	0
4/30/2020	5:00:00 p, m,	24.6	69	0
4/30/2020	6:00:00 p, m,	21.2	89	0.2
4/30/2020	7:00:00 p, m,	19.6	86	0.2
4/30/2020	8:00:00 p, m,	19.8	87	0
4/30/2020	9:00:00 p, m,	19.2	87	0
4/30/2020	10:00:00 p, m	18.7	91	0
4/30/2020	11:00:00 p, m	18.1	96	1.8
Apr_20		19.2756944	85.8666667	47
5/1/2020	12:00:00 a, m	17.6	96	0.4
5/1/2020	1:00:00 a, m,	17.2	97	2.2
5/1/2020	2:00:00 a, m,	17	97	1.2
5/1/2020	3:00:00 a, m,	16.8	97	1.2
5/1/2020	4:00:00 a, m,	16.8	97	0.6
5/1/2020	5:00:00 a, m,	16.8	97	0.6
5/1/2020	6:00:00 a, m,	16.8	97	0.2
5/1/2020	7:00:00 a, m,	16.8	97	0
5/1/2020	8:00:00 a, m,	16.9	98	0
5/1/2020	9:00:00 a, m,	17.4	97	0
5/1/2020	10:00:00 a, m	18.2	98	0
5/1/2020	11:00:00 a, m	18.7	97	0
5/1/2020	12:00:00 p, m	18.9	98	0
5/1/2020	1:00:00 p, m,	20.2	96	0
5/1/2020	2:00:00 p, m,	21.2	92	0
5/1/2020	3:00:00 p, m,	20.1	94	0
5/1/2020	4:00:00 p, m,	20.7	88	0
5/1/2020	5:00:00 p, m,	20.1	95	0
5/1/2020	6:00:00 p, m,	19.9	94	0
5/1/2020	7:00:00 p, m,	19.2	95	0
5/1/2020	8:00:00 p, m,	18.7	94	0
5/1/2020	9:00:00 p, m,	18.2	90	0
5/1/2020	10:00:00 p, m	18.1	90	0
5/1/2020	11:00:00 p, m	18.5	91	0
5/2/2020	12:00:00 a, m	18.6	90	0
5/2/2020	1:00:00 a, m,	18.4	89	0
5/2/2020	2:00:00 a, m,	18.4	91	0
5/2/2020	3:00:00 a, m,	18.5	91	0
5/2/2020	4:00:00 a, m,	18.6	90	0
5/2/2020	5:00:00 a, m,	18.4	89	0

5/2/2020	6:00:00 a, m,	18.3	88	0
5/2/2020	7:00:00 a, m,	18.4	89	0.2
5/2/2020	8:00:00 a, m,	18.5	91	0
5/2/2020	9:00:00 a, m,	18.7	94	0
5/2/2020	10:00:00 a, m	19.4	94	0
5/2/2020	11:00:00 a, m	20.3	94	0
5/2/2020	12:00:00 p, m	20.3	95	0
5/2/2020	1:00:00 p, m,	20.3	85	0
5/2/2020	2:00:00 p, m,	20.3	90	0
5/2/2020	3:00:00 p, m,	19.9	93	0
5/2/2020	4:00:00 p, m,	19.2	93	0
5/2/2020	5:00:00 p, m,	19.2	95	0
5/2/2020	6:00:00 p, m,	18.9	92	0
5/2/2020	7:00:00 p, m,	18.3	91	0
5/2/2020	8:00:00 p, m,	18.1	88	0
5/2/2020	9:00:00 p, m,	18.2	86	0
5/2/2020	10:00:00 p, m	18.6	86	0
5/2/2020	11:00:00 p, m	18.3	85	0
5/3/2020	12:00:00 a, m	18.3	87	0
5/3/2020	1:00:00 a, m,	18.3	86	0
5/3/2020	2:00:00 a, m,	18.6	86	0
5/3/2020	3:00:00 a, m,	18.4	86	0
5/3/2020	4:00:00 a, m,	18.3	86	0
5/3/2020	5:00:00 a, m,	18.3	87	0
5/3/2020	6:00:00 a, m,	18.1	89	0
5/3/2020	7:00:00 a, m,	17.8	92	0
5/3/2020	8:00:00 a, m,	18.4	92	0
5/3/2020	9:00:00 a, m,	18.6	91	0
5/3/2020	10:00:00 a, m	18.9	94	0
5/3/2020	11:00:00 a, m	19.2	92	0
5/3/2020	12:00:00 p, m	20.1	87	0
5/3/2020	1:00:00 p, m,	20	90	0
5/3/2020	2:00:00 p, m,	20.2	93	0
5/3/2020	3:00:00 p, m,	19.6	89	0
5/3/2020	4:00:00 p, m,	19.7	90	0
5/3/2020	5:00:00 p, m,	19.8	89	0
5/3/2020	6:00:00 p, m,	20.2	87	0
5/3/2020	7:00:00 p, m,	19.4	83	0
5/3/2020	8:00:00 p, m,	19	84	0
5/3/2020	9:00:00 p, m,	19.1	86	0
5/3/2020	10:00:00 p, m	19.1	80	0
5/3/2020	11:00:00 p, m	19.3	79	0
5/4/2020	12:00:00 a, m	19.2	79	0
5/4/2020	1:00:00 a, m,	19.2	77	0
5/4/2020	2:00:00 a, m,	18.7	80	0
5/4/2020	3:00:00 a, m,	18.6	79	0
5/4/2020	4:00:00 a, m,	18.7	79	0

5/4/2020	5:00:00 a, m,	18.4	80	0
5/4/2020	6:00:00 a, m,	18.4	80	0
5/4/2020	7:00:00 a, m,	18.5	83	0
5/4/2020	8:00:00 a, m,	19	83	0
5/4/2020	9:00:00 a, m,	19.3	84	0
5/4/2020	10:00:00 a, m	19.3	88	0
5/4/2020	11:00:00 a, m	19.8	87	0
5/4/2020	12:00:00 p, m	20.7	85	0
5/4/2020	1:00:00 p, m,	20.3	84	0
5/4/2020	2:00:00 p, m,	21.2	79	0
5/4/2020	3:00:00 p, m,	21.1	78	0
5/4/2020	4:00:00 p, m,	22.8	74	0
5/4/2020	5:00:00 p, m,	22.9	77	0
5/4/2020	6:00:00 p, m,	21.8	82	0
5/4/2020	7:00:00 p, m,	20.3	80	0
5/4/2020	8:00:00 p, m,	19.9	81	0
5/4/2020	9:00:00 p, m,	19.8	78	0
5/4/2020	10:00:00 p, m	19.6	80	0
5/4/2020	11:00:00 p, m	19.8	79	0
5/5/2020	12:00:00 a, m	20	80	0
5/5/2020	1:00:00 a, m,	20	82	0
5/5/2020	2:00:00 a, m,	17.1	95	4.2
5/5/2020	3:00:00 a, m,	16.8	96	3
5/5/2020	4:00:00 a, m,	16.3	97	1.8
5/5/2020	5:00:00 a, m,	16.3	97	1
5/5/2020	6:00:00 a, m,	15.4	95	0
5/5/2020	7:00:00 a, m,	15.4	96	0
5/5/2020	8:00:00 a, m,	15.9	96	0
5/5/2020	9:00:00 a, m,	16.8	95	0
5/5/2020	10:00:00 a, m	18.1	96	0
5/5/2020	11:00:00 a, m	18.9	95	0
5/5/2020	12:00:00 p, m	20.1	95	0
5/5/2020	1:00:00 p, m,	21.1	86	0
5/5/2020	2:00:00 p, m,	21.8	86	0
5/5/2020	3:00:00 p, m,	22.3	87	0
5/5/2020	4:00:00 p, m,	22.8	84	0
5/5/2020	5:00:00 p, m,	21.5	86	0
5/5/2020	6:00:00 p, m,	20.4	81	0.2
5/5/2020	7:00:00 p, m,	19.9	81	0
5/5/2020	8:00:00 p, m,	20.6	75	0
5/5/2020	9:00:00 p, m,	20.6	78	0
5/5/2020	10:00:00 p, m	20.3	80	0
5/5/2020	11:00:00 p, m	18.9	87	0
5/6/2020	12:00:00 a, m	18.9	88	0
5/6/2020	1:00:00 a, m,	18.7	88	0
5/6/2020	2:00:00 a, m,	18.7	89	0
5/6/2020	3:00:00 a, m,	18.3	88	0

5/6/2020	4:00:00 a, m,	18.4	89	0
5/6/2020	5:00:00 a, m,	18.2	89	0
5/6/2020	6:00:00 a, m,	18.3	88	0
5/6/2020	7:00:00 a, m,	18.4	87	0
5/6/2020	8:00:00 a, m,	18.7	87	0
5/6/2020	9:00:00 a, m,	19.5	90	0
5/6/2020	10:00:00 a, m	19.7	90	0
5/6/2020	11:00:00 a, m	20.2	87	0.4
5/6/2020	12:00:00 p, m	21.1	81	0
5/6/2020	1:00:00 p, m,	20.8	84	0
5/6/2020	2:00:00 p, m,	20.3	85	0
5/6/2020	3:00:00 p, m,	20.2	82	0
5/6/2020	4:00:00 p, m,	20	81	0
5/6/2020	5:00:00 p, m,	20.1	78	0
5/6/2020	6:00:00 p, m,	20.1	77	0
5/6/2020	7:00:00 p, m,	19.6	79	0
5/6/2020	8:00:00 p, m,	19.2	79	0
5/6/2020	9:00:00 p, m,	19.2	79	0
5/6/2020	10:00:00 p, m	19.3	80	0
5/6/2020	11:00:00 p, m	19	82	0
5/7/2020	12:00:00 a, m	18.7	85	0
5/7/2020	1:00:00 a, m,	18.3	84	0
5/7/2020	2:00:00 a, m,	17	94	0.2
5/7/2020	3:00:00 a, m,	16.4	96	3.6
5/7/2020	4:00:00 a, m,	16.4	97	1.4
5/7/2020	5:00:00 a, m,	16.3	97	0
5/7/2020	6:00:00 a, m,	16.3	97	0.2
5/7/2020	7:00:00 a, m,	16.4	97	1.8
5/7/2020	8:00:00 a, m,	16.6	98	1.2
5/7/2020	9:00:00 a, m,	17	98	0.4
5/7/2020	10:00:00 a, m	17.3	98	0.2
5/7/2020	11:00:00 a, m	17.4	98	0
5/7/2020	12:00:00 p, m	17.9	98	0
5/7/2020	1:00:00 p, m,	18.6	98	0
5/7/2020	2:00:00 p, m,	19.1	99	0
5/7/2020	3:00:00 p, m,	19	98	0
5/7/2020	4:00:00 p, m,	18.8	97	0
5/7/2020	5:00:00 p, m,	18.4	96	0
5/7/2020	6:00:00 p, m,	17.8	94	0
5/7/2020	7:00:00 p, m,	17.5	88	0
5/7/2020	8:00:00 p, m,	18.4	82	0
5/7/2020	9:00:00 p, m,	18.9	79	0
5/7/2020	10:00:00 p, m	19.2	78	0
5/7/2020	11:00:00 p, m	19.2	79	0
5/8/2020	12:00:00 a, m	19.1	79	0
5/8/2020	1:00:00 a, m,	19.2	78	0
5/8/2020	2:00:00 a, m,	18.8	81	0

5/8/2020	3:00:00 a, m,	18.4	80	0
5/8/2020	4:00:00 a, m,	18.4	81	0
5/8/2020	5:00:00 a, m,	18.3	83	0
5/8/2020	6:00:00 a, m,	18.2	83	0
5/8/2020	7:00:00 a, m,	18.4	84	0
5/8/2020	8:00:00 a, m,	18.4	88	0
5/8/2020	9:00:00 a, m,	18.7	88	0
5/8/2020	10:00:00 a, m	17.9	90	0
5/8/2020	11:00:00 a, m	17.2	96	2.6
5/8/2020	12:00:00 p, m	17.7	98	1.8
5/8/2020	1:00:00 p, m,	17.7	98	0.2
5/8/2020	2:00:00 p, m,	18.1	98	0
5/8/2020	3:00:00 p, m,	18.2	98	0
5/8/2020	4:00:00 p, m,	18.1	98	0
5/8/2020	5:00:00 p, m,	17.8	96	0
5/8/2020	6:00:00 p, m,	17.6	97	0
5/8/2020	7:00:00 p, m,	17.3	96	0
5/8/2020	8:00:00 p, m,	17.3	96	0
5/8/2020	9:00:00 p, m,	17.3	93	0
5/8/2020	10:00:00 p, m	16.9	94	0
5/8/2020	11:00:00 p, m	16.9	93	0
5/9/2020	12:00:00 a, m	16.7	94	0
5/9/2020	1:00:00 a, m,	16.3	96	0
5/9/2020	2:00:00 a, m,	16.3	95	0
5/9/2020	3:00:00 a, m,	16.2	95	0
5/9/2020	4:00:00 a, m,	16.4	95	0
5/9/2020	5:00:00 a, m,	16.7	93	0
5/9/2020	6:00:00 a, m,	16.8	92	0
5/9/2020	7:00:00 a, m,	17.1	92	0
5/9/2020	8:00:00 a, m,	17.5	93	0
5/9/2020	9:00:00 a, m,	17.9	95	0
5/9/2020	10:00:00 a, m	18.4	93	0
5/9/2020	11:00:00 a, m	18.8	93	0
5/9/2020	12:00:00 p, m	20.2	88	0
5/9/2020	1:00:00 p, m,	20.9	83	0.2
5/9/2020	2:00:00 p, m,	21.4	84	0
5/9/2020	3:00:00 p, m,	21.7	85	0
5/9/2020	4:00:00 p, m,	22.2	84	0
5/9/2020	5:00:00 p, m,	21.5	89	0
5/9/2020	6:00:00 p, m,	21	86	0
5/9/2020	7:00:00 p, m,	19.8	82	0
5/9/2020	8:00:00 p, m,	19.4	81	0
5/9/2020	9:00:00 p, m,	20.1	77	0
5/9/2020	10:00:00 p, m	19.9	77	0
5/9/2020	11:00:00 p, m	19.1	80	0
5/10/2020	12:00:00 a, m	19.3	80	0
5/10/2020	1:00:00 a, m,	19.6	78	0

5/10/2020	2:00:00 a, m,	19.2	81	0
5/10/2020	3:00:00 a, m,	18.9	82	0
5/10/2020	4:00:00 a, m,	18.6	83	0
5/10/2020	5:00:00 a, m,	18.4	84	0
5/10/2020	6:00:00 a, m,	18.6	85	0
5/10/2020	7:00:00 a, m,	18.6	86	0
5/10/2020	8:00:00 a, m,	18.8	89	0
5/10/2020	9:00:00 a, m,	19.3	90	0
5/10/2020	10:00:00 a, m	19.7	91	0
5/10/2020	11:00:00 a, m	20.3	86	0
5/10/2020	12:00:00 p, m	20.8	86	0
5/10/2020	1:00:00 p, m,	21.3	86	0.8
5/10/2020	2:00:00 p, m,	21.3	87	0
5/10/2020	3:00:00 p, m,	21.4	84	0
5/10/2020	4:00:00 p, m,	21.4	87	0
5/10/2020	5:00:00 p, m,	22.3	84	0
5/10/2020	6:00:00 p, m,	21.1	88	0
5/10/2020	7:00:00 p, m,	19.6	91	0
5/10/2020	8:00:00 p, m,	19.3	91	0
5/10/2020	9:00:00 p, m,	19.1	90	0
5/10/2020	10:00:00 p, m	18.9	89	0
5/10/2020	11:00:00 p, m	19	87	0
5/11/2020	12:00:00 a, m	18.8	86	0
5/11/2020	1:00:00 a, m,	18.6	86	0
5/11/2020	2:00:00 a, m,	18.3	85	0
5/11/2020	3:00:00 a, m,	18.7	84	0
5/11/2020	4:00:00 a, m,	18.7	84	0
5/11/2020	5:00:00 a, m,	18.2	84	0
5/11/2020	6:00:00 a, m,	18.1	85	0
5/11/2020	7:00:00 a, m,	18.3	85	0
5/11/2020	8:00:00 a, m,	18.7	86	0
5/11/2020	9:00:00 a, m,	19.4	86	0
5/11/2020	10:00:00 a, m	20.7	87	0
5/11/2020	11:00:00 a, m	21.6	86	0
5/11/2020	12:00:00 p, m	21.7	87	0
5/11/2020	1:00:00 p, m,	22.2	82	0
5/11/2020	2:00:00 p, m,	22.2	84	0
5/11/2020	3:00:00 p, m,	22	86	0
5/11/2020	4:00:00 p, m,	22.2	86	0
5/11/2020	5:00:00 p, m,	21.6	85	0
5/11/2020	6:00:00 p, m,	21	83	0
5/11/2020	7:00:00 p, m,	20.6	81	0
5/11/2020	8:00:00 p, m,	20.3	78	0
5/11/2020	9:00:00 p, m,	20.2	82	0
5/11/2020	10:00:00 p, m	19.6	84	0
5/11/2020	11:00:00 p, m	19.2	86	0
5/12/2020	12:00:00 a, m	19.6	88	0

5/12/2020	1:00:00 a, m,	18.4	96	8.2
5/12/2020	2:00:00 a, m,	17.9	96	0
5/12/2020	3:00:00 a, m,	17.7	97	0
5/12/2020	4:00:00 a, m,	17.6	97	0
5/12/2020	5:00:00 a, m,	17.3	97	0
5/12/2020	6:00:00 a, m,	17.4	97	0
5/12/2020	7:00:00 a, m,	17.9	97	0
5/12/2020	8:00:00 a, m,	18.2	98	0
5/12/2020	9:00:00 a, m,	18.7	98	0
5/12/2020	10:00:00 a, m	18.7	97	0
5/12/2020	11:00:00 a, m	18.4	97	1.2
5/12/2020	12:00:00 p, m	18.7	98	0.8
5/12/2020	1:00:00 p, m,	19.4	97	0
5/12/2020	2:00:00 p, m,	19.9	97	0
5/12/2020	3:00:00 p, m,	20.2	97	0
5/12/2020	4:00:00 p, m,	20.4	98	1.2
5/12/2020	5:00:00 p, m,	19.7	98	0
5/12/2020	6:00:00 p, m,	19.1	97	0
5/12/2020	7:00:00 p, m,	18.4	97	0
5/12/2020	8:00:00 p, m,	18.2	97	0
5/12/2020	9:00:00 p, m,	17.8	97	0
5/12/2020	10:00:00 p, m	17.5	97	0
5/12/2020	11:00:00 p, m	17.4	96	0
5/13/2020	12:00:00 a, m	17.4	95	0
5/13/2020	1:00:00 a, m,	17.3	95	0
5/13/2020	2:00:00 a, m,	17.1	96	0
5/13/2020	3:00:00 a, m,	17.2	96	0
5/13/2020	4:00:00 a, m,	16.8	96	0
5/13/2020	5:00:00 a, m,	16.7	96	0
5/13/2020	6:00:00 a, m,	16.7	96	0
5/13/2020	7:00:00 a, m,	16.9	96	0
5/13/2020	8:00:00 a, m,	16.9	98	0.4
5/13/2020	9:00:00 a, m,	17.1	98	0
5/13/2020	10:00:00 a, m	17.4	98	0.2
5/13/2020	11:00:00 a, m	17.9	98	0.2
5/13/2020	12:00:00 p, m	18.1	98	0.4
5/13/2020	1:00:00 p, m,	17.8	98	0.8
5/13/2020	2:00:00 p, m,	17.7	99	0.4
5/13/2020	3:00:00 p, m,	17.3	98	1.4
5/13/2020	4:00:00 p, m,	17.7	99	0.4
5/13/2020	5:00:00 p, m,	17.8	99	0.2
5/13/2020	6:00:00 p, m,	17.4	98	0
5/13/2020	7:00:00 p, m,	16.5	97	0
5/13/2020	8:00:00 p, m,	15.9	97	0
5/13/2020	9:00:00 p, m,	15.7	96	0
5/13/2020	10:00:00 p, m	15.7	96	0
5/13/2020	11:00:00 p, m	15.7	95	0

5/14/2020	12:00:00 a, m	15.4	94	0
5/14/2020	1:00:00 a, m,	15.3	94	0
5/14/2020	2:00:00 a, m,	15.2	94	0
5/14/2020	3:00:00 a, m,	15	95	0
5/14/2020	4:00:00 a, m,	15.2	95	0
5/14/2020	5:00:00 a, m,	15	94	0
5/14/2020	6:00:00 a, m,	14.9	94	0
5/14/2020	7:00:00 a, m,	15.2	95	0
5/14/2020	8:00:00 a, m,	15.8	94	0
5/14/2020	9:00:00 a, m,	16.7	95	0
5/14/2020	10:00:00 a, m	17.5	95	0
5/14/2020	11:00:00 a, m	18.3	96	0.2
5/14/2020	12:00:00 p, m	20	92	0
5/14/2020	1:00:00 p, m,	20.5	91	0
5/14/2020	2:00:00 p, m,	20.4	92	0
5/14/2020	3:00:00 p, m,	20.6	91	0
5/14/2020	4:00:00 p, m,	20.2	92	0
5/14/2020	5:00:00 p, m,	19.7	92	0
5/14/2020	6:00:00 p, m,	19	86	0
5/14/2020	7:00:00 p, m,	18.7	87	0
5/14/2020	8:00:00 p, m,	18.5	88	0
5/14/2020	9:00:00 p, m,	18.6	86	0
5/14/2020	10:00:00 p, m	18	86	0
5/14/2020	11:00:00 p, m	17.9	87	0
5/15/2020	12:00:00 a, m	17.8	87	0
5/15/2020	1:00:00 a, m,	18.1	85	0
5/15/2020	2:00:00 a, m,	18.1	86	0
5/15/2020	3:00:00 a, m,	17.9	87	0
5/15/2020	4:00:00 a, m,	17.8	87	0
5/15/2020	5:00:00 a, m,	18.1	84	0
5/15/2020	6:00:00 a, m,	18.2	85	0
5/15/2020	7:00:00 a, m,	17.7	90	0
5/15/2020	8:00:00 a, m,	17.5	93	0
5/15/2020	9:00:00 a, m,	18.4	93	0
5/15/2020	10:00:00 a, m	19.1	92	0
5/15/2020	11:00:00 a, m	19.6	92	0
5/15/2020	12:00:00 p, m	19.9	91	0
5/15/2020	1:00:00 p, m,	20.3	91	0
5/15/2020	2:00:00 p, m,	20.9	88	0
5/15/2020	3:00:00 p, m,	22.1	82	0
5/15/2020	4:00:00 p, m,	22.3	84	0
5/15/2020	5:00:00 p, m,	22.1	83	0
5/15/2020	6:00:00 p, m,	21.3	87	0
5/15/2020	7:00:00 p, m,	19.6	87	0
5/15/2020	8:00:00 p, m,	19.2	81	0
5/15/2020	9:00:00 p, m,	18.9	85	0
5/15/2020	10:00:00 p, m	18.8	82	0

5/15/2020	11:00:00 p, m	19.6	78	0
5/16/2020	12:00:00 a, m	19.2	82	0
5/16/2020	1:00:00 a, m,	18.8	85	0
5/16/2020	2:00:00 a, m,	18.7	85	0
5/16/2020	3:00:00 a, m,	18.6	85	0
5/16/2020	4:00:00 a, m,	18.6	85	0
5/16/2020	5:00:00 a, m,	18.3	87	0
5/16/2020	6:00:00 a, m,	18.2	86	0
5/16/2020	7:00:00 a, m,	18.4	88	0
5/16/2020	8:00:00 a, m,	18.7	88	0
5/16/2020	9:00:00 a, m,	18.8	91	0
5/16/2020	10:00:00 a, m	19	92	0
5/16/2020	11:00:00 a, m	19.8	90	0
5/16/2020	12:00:00 p, m	20.4	91	0
5/16/2020	1:00:00 p, m,	20.7	89	0
5/16/2020	2:00:00 p, m,	21.4	88	0
5/16/2020	3:00:00 p, m,	21.4	86	0
5/16/2020	4:00:00 p, m,	21.1	89	0
5/16/2020	5:00:00 p, m,	21.1	86	0
5/16/2020	6:00:00 p, m,	20.4	81	0
5/16/2020	7:00:00 p, m,	20	81	0
5/16/2020	8:00:00 p, m,	19.9	81	0
5/16/2020	9:00:00 p, m,	19.4	84	0
5/16/2020	10:00:00 p, m	18.7	89	0
5/16/2020	11:00:00 p, m	17.3	96	2.8
5/17/2020	12:00:00 a, m	17	96	0
5/17/2020	1:00:00 a, m,	17	96	0
5/17/2020	2:00:00 a, m,	17.1	95	0
5/17/2020	3:00:00 a, m,	17.1	95	0
5/17/2020	4:00:00 a, m,	17.1	96	0
5/17/2020	5:00:00 a, m,	17	94	0
5/17/2020	6:00:00 a, m,	16.8	94	0
5/17/2020	7:00:00 a, m,	17.1	96	0
5/17/2020	8:00:00 a, m,	17.8	96	0
5/17/2020	9:00:00 a, m,	18.3	95	0
5/17/2020	10:00:00 a, m	18.6	95	0.2
5/17/2020	11:00:00 a, m	18.9	95	0
5/17/2020	12:00:00 p, m	19.6	93	0
5/17/2020	1:00:00 p, m,	19.8	97	0
5/17/2020	2:00:00 p, m,	19.4	96	0
5/17/2020	3:00:00 p, m,	19.3	98	0
5/17/2020	4:00:00 p, m,	19.3	97	0
5/17/2020	5:00:00 p, m,	18.6	96	0
5/17/2020	6:00:00 p, m,	17.8	97	0
5/17/2020	7:00:00 p, m,	17.7	97	0
5/17/2020	8:00:00 p, m,	17.7	96	0
5/17/2020	9:00:00 p, m,	17.8	96	0

5/17/2020	10:00:00 p, m	17.6	95	0
5/17/2020	11:00:00 p, m	17.5	96	0
5/18/2020	12:00:00 a, m	17.6	96	0
5/18/2020	1:00:00 a, m,	17.6	96	0
5/18/2020	2:00:00 a, m,	17.6	96	0
5/18/2020	3:00:00 a, m,	17.4	95	0
5/18/2020	4:00:00 a, m,	17.3	95	0
5/18/2020	5:00:00 a, m,	17.3	95	0
5/18/2020	6:00:00 a, m,	16.8	95	0
5/18/2020	7:00:00 a, m,	17.1	96	0
5/18/2020	8:00:00 a, m,	17.8	96	0
5/18/2020	9:00:00 a, m,	18.4	95	0
5/18/2020	10:00:00 a, m	19.2	94	0.4
5/18/2020	11:00:00 a, m	19.8	94	0
5/18/2020	12:00:00 p, m	19.9	92	0
5/18/2020	1:00:00 p, m,	20.6	93	0
5/18/2020	2:00:00 p, m,	21.1	94	0
5/18/2020	3:00:00 p, m,	21.3	88	0
5/18/2020	4:00:00 p, m,	21.5	88	0
5/18/2020	5:00:00 p, m,	21.6	90	0
5/18/2020	6:00:00 p, m,	20.7	87	0
5/18/2020	7:00:00 p, m,	19.5	85	0
5/18/2020	8:00:00 p, m,	19.2	83	0
5/18/2020	9:00:00 p, m,	19.2	84	0
5/18/2020	10:00:00 p, m	19	83	0
5/18/2020	11:00:00 p, m	18.4	85	0
5/19/2020	12:00:00 a, m	18.2	86	0
5/19/2020	1:00:00 a, m,	18.3	86	0
5/19/2020	2:00:00 a, m,	18.2	86	0
5/19/2020	3:00:00 a, m,	17.8	87	0
5/19/2020	4:00:00 a, m,	18.2	84	0
5/19/2020	5:00:00 a, m,	18.2	85	0
5/19/2020	6:00:00 a, m,	18.1	86	0
5/19/2020	7:00:00 a, m,	18.2	86	0
5/19/2020	8:00:00 a, m,	17.2	94	0.2
5/19/2020	9:00:00 a, m,	17.3	96	0.4
5/19/2020	10:00:00 a, m	17.6	96	0.6
5/19/2020	11:00:00 a, m	17.7	97	0.4
5/19/2020	12:00:00 p, m	17.8	97	0
5/19/2020	1:00:00 p, m,	18.2	98	0.6
5/19/2020	2:00:00 p, m,	18.2	96	0.4
5/19/2020	3:00:00 p, m,	18.7	97	0
5/19/2020	4:00:00 p, m,	19.5	98	0
5/19/2020	5:00:00 p, m,	19.8	90	0
5/19/2020	6:00:00 p, m,	19.3	92	0
5/19/2020	7:00:00 p, m,	18.5	95	0
5/19/2020	8:00:00 p, m,	18.2	95	0

5/19/2020	9:00:00 p, m,	17.8	94	0
5/19/2020	10:00:00 p, m	17.6	92	0
5/19/2020	11:00:00 p, m	17.7	94	0
5/20/2020	12:00:00 a, m	17.9	92	0
5/20/2020	1:00:00 a, m,	17.7	93	0
5/20/2020	2:00:00 a, m,	17.8	91	0
5/20/2020	3:00:00 a, m,	17.8	87	0
5/20/2020	4:00:00 a, m,	17.5	90	0
5/20/2020	5:00:00 a, m,	17.6	88	0
5/20/2020	6:00:00 a, m,	17.2	90	0
5/20/2020	7:00:00 a, m,	17.7	91	0
5/20/2020	8:00:00 a, m,	17.8	91	0
5/20/2020	9:00:00 a, m,	18.4	92	0
5/20/2020	10:00:00 a, m	19.3	92	0
5/20/2020	11:00:00 a, m	19.3	93	0
5/20/2020	12:00:00 p, m	20.1	91	0
5/20/2020	1:00:00 p, m,	20	88	0
5/20/2020	2:00:00 p, m,	20.4	92	0
5/20/2020	3:00:00 p, m,	20.3	88	0
5/20/2020	4:00:00 p, m,	20.2	89	0
5/20/2020	5:00:00 p, m,	21.4	87	0
5/20/2020	6:00:00 p, m,	20.7	85	0
5/20/2020	7:00:00 p, m,	19.7	81	0
5/20/2020	8:00:00 p, m,	19.6	81	0
5/20/2020	9:00:00 p, m,	19.4	83	0
5/20/2020	10:00:00 p, m	19.7	79	0
5/20/2020	11:00:00 p, m	19.1	83	0
5/21/2020	12:00:00 a, m	19.2	79	0
5/21/2020	1:00:00 a, m,	19.4	79	0
5/21/2020	2:00:00 a, m,	18.8	84	0
5/21/2020	3:00:00 a, m,	18.6	85	0
5/21/2020	4:00:00 a, m,	18.3	84	0
5/21/2020	5:00:00 a, m,	18.3	85	0
5/21/2020	6:00:00 a, m,	18.1	86	0
5/21/2020	7:00:00 a, m,	18.4	85	0
5/21/2020	8:00:00 a, m,	18.7	86	0
5/21/2020	9:00:00 a, m,	19.2	88	0
5/21/2020	10:00:00 a, m	19.8	87	0
5/21/2020	11:00:00 a, m	20.3	87	0
5/21/2020	12:00:00 p, m	20.4	87	0
5/21/2020	1:00:00 p, m,	20.8	87	0
5/21/2020	2:00:00 p, m,	20.6	85	0
5/21/2020	3:00:00 p, m,	20.9	89	0.2
5/21/2020	4:00:00 p, m,	22.1	81	0
5/21/2020	5:00:00 p, m,	22.3	81	0
5/21/2020	6:00:00 p, m,	21.7	83	0
5/21/2020	7:00:00 p, m,	20.2	79	0

5/21/2020	8:00:00 p, m,	19.9	82	0
5/21/2020	9:00:00 p, m,	18.3	90	0
5/21/2020	10:00:00 p, m	19.5	79	0
5/21/2020	11:00:00 p, m	19.5	80	0
5/22/2020	12:00:00 a, m	19.1	83	0
5/22/2020	1:00:00 a, m,	19.1	79	0
5/22/2020	2:00:00 a, m,	19.3	77	0
5/22/2020	3:00:00 a, m,	18.9	81	0
5/22/2020	4:00:00 a, m,	19.2	80	0
5/22/2020	5:00:00 a, m,	18.6	81	0
5/22/2020	6:00:00 a, m,	19.1	80	0
5/22/2020	7:00:00 a, m,	18.9	78	0
5/22/2020	8:00:00 a, m,	19.2	83	0
5/22/2020	9:00:00 a, m,	19.7	84	0
5/22/2020	10:00:00 a, m	20.2	87	0
5/22/2020	11:00:00 a, m	20.4	81	0
5/22/2020	12:00:00 p, m	20.7	83	0
5/22/2020	1:00:00 p, m,	21.3	86	0
5/22/2020	2:00:00 p, m,	21.1	81	0
5/22/2020	3:00:00 p, m,	20.8	79	0
5/22/2020	4:00:00 p, m,	21.1	79	0
5/22/2020	5:00:00 p, m,	20.8	79	0
5/22/2020	6:00:00 p, m,	20.3	80	0
5/22/2020	7:00:00 p, m,	19.7	79	0
5/22/2020	8:00:00 p, m,	19.8	75	0
5/22/2020	9:00:00 p, m,	20.1	76	0
5/22/2020	10:00:00 p, m	19.9	79	0
5/22/2020	11:00:00 p, m	19.8	79	0
5/23/2020	12:00:00 a, m	19.4	82	0
5/23/2020	1:00:00 a, m,	19.1	83	0
5/23/2020	2:00:00 a, m,	19.1	83	0
5/23/2020	3:00:00 a, m,	18.7	84	0
5/23/2020	4:00:00 a, m,	18.7	84	0
5/23/2020	5:00:00 a, m,	18.6	84	0
5/23/2020	6:00:00 a, m,	19.1	78	0
5/23/2020	7:00:00 a, m,	18.8	80	0
5/23/2020	8:00:00 a, m,	19.3	80	0
5/23/2020	9:00:00 a, m,	20.2	82	0
5/23/2020	10:00:00 a, m	19.9	84	0
5/23/2020	11:00:00 a, m	20.6	85	0
5/23/2020	12:00:00 p, m	20.9	87	0
5/23/2020	1:00:00 p, m,	22.3	82	0
5/23/2020	2:00:00 p, m,	22.3	83	0
5/23/2020	3:00:00 p, m,	22.3	81	0
5/23/2020	4:00:00 p, m,	22.3	80	0
5/23/2020	5:00:00 p, m,	22.7	65	0
5/23/2020	6:00:00 p, m,	21.8	70	0

5/23/2020	7:00:00 p, m,	20.6	71	0
5/23/2020	8:00:00 p, m,	19.8	72	0
5/23/2020	9:00:00 p, m,	20.1	70	0
5/23/2020	10:00:00 p, m	19.4	74	0
5/23/2020	11:00:00 p, m	19.1	76	0
5/24/2020	12:00:00 a, m	19.3	75	0
5/24/2020	1:00:00 a, m,	19.5	76	0
5/24/2020	2:00:00 a, m,	19.4	76	0
5/24/2020	3:00:00 a, m,	19.3	78	0
5/24/2020	4:00:00 a, m,	19.4	78	0
5/24/2020	5:00:00 a, m,	18.3	87	0.2
5/24/2020	6:00:00 a, m,	16.4	94	1.2
5/24/2020	7:00:00 a, m,	16.8	94	0
5/24/2020	8:00:00 a, m,	17.1	95	0
5/24/2020	9:00:00 a, m,	17.3	97	1.2
5/24/2020	10:00:00 a, m	18	94	0
5/24/2020	11:00:00 a, m	18.9	96	0
5/24/2020	12:00:00 p, m	19.6	95	0
5/24/2020	1:00:00 p, m,	19.6	93	0.2
5/24/2020	2:00:00 p, m,	18.7	96	1
5/24/2020	3:00:00 p, m,	18.9	94	0
5/24/2020	4:00:00 p, m,	20.1	94	0
5/24/2020	5:00:00 p, m,	20.9	90	0
5/24/2020	6:00:00 p, m,	20.2	90	0
5/24/2020	7:00:00 p, m,	18.8	92	0
5/24/2020	8:00:00 p, m,	18.7	84	0
5/24/2020	9:00:00 p, m,	18.2	88	0
5/24/2020	10:00:00 p, m	18.9	86	0
5/24/2020	11:00:00 p, m	18.7	86	0
5/25/2020	12:00:00 a, m	18.3	85	0
5/25/2020	1:00:00 a, m,	18.8	80	0
5/25/2020	2:00:00 a, m,	18.3	82	0
5/25/2020	3:00:00 a, m,	18.2	84	0
5/25/2020	4:00:00 a, m,	17.8	85	0
5/25/2020	5:00:00 a, m,	17.9	86	0
5/25/2020	6:00:00 a, m,	18.1	85	0
5/25/2020	7:00:00 a, m,	18.7	86	0
5/25/2020	8:00:00 a, m,	18.4	88	0
5/25/2020	9:00:00 a, m,	19.1	87	0
5/25/2020	10:00:00 a, m	19.4	89	0
5/25/2020	11:00:00 a, m	20.3	85	0
5/25/2020	12:00:00 p, m	21.3	85	0
5/25/2020	1:00:00 p, m,	22.7	81	0
5/25/2020	2:00:00 p, m,	23.7	74	0
5/25/2020	3:00:00 p, m,	23.1	75	0
5/25/2020	4:00:00 p, m,	23.2	80	0
5/25/2020	5:00:00 p, m,	22.2	79	0

5/25/2020	6:00:00 p, m,	21.3	77	0
5/25/2020	7:00:00 p, m,	20.4	79	0
5/25/2020	8:00:00 p, m,	18.2	92	0.4
5/25/2020	9:00:00 p, m,	18.4	89	0
5/25/2020	10:00:00 p, m	18.8	86	0
5/25/2020	11:00:00 p, m	18.6	89	0
5/26/2020	12:00:00 a, m	18.2	91	0
5/26/2020	1:00:00 a, m,	17.9	90	0
5/26/2020	2:00:00 a, m,	17.8	92	0
5/26/2020	3:00:00 a, m,	17.3	93	0.2
5/26/2020	4:00:00 a, m,	17.4	93	0
5/26/2020	5:00:00 a, m,	17.6	93	0
5/26/2020	6:00:00 a, m,	17.4	93	0.2
5/26/2020	7:00:00 a, m,	17.1	96	1
5/26/2020	8:00:00 a, m,	17.4	98	0.2
5/26/2020	9:00:00 a, m,	17.5	97	0
5/26/2020	10:00:00 a, m	18.1	98	0.2
5/26/2020	11:00:00 a, m	19.2	98	0
5/26/2020	12:00:00 p, m	19	98	0.2
5/26/2020	1:00:00 p, m,	19	97	0
5/26/2020	2:00:00 p, m,	19	97	0
5/26/2020	3:00:00 p, m,	19.1	95	0
5/26/2020	4:00:00 p, m,	19.1	93	0
5/26/2020	5:00:00 p, m,	19.1	94	0
5/26/2020	6:00:00 p, m,	18.6	93	0
5/26/2020	7:00:00 p, m,	17.8	90	0
5/26/2020	8:00:00 p, m,	17.7	89	0
5/26/2020	9:00:00 p, m,	17.9	87	0
5/26/2020	10:00:00 p, m	18.1	87	0
5/26/2020	11:00:00 p, m	18.2	86	0
5/27/2020	12:00:00 a, m	18.1	86	0
5/27/2020	1:00:00 a, m,	17.8	88	0
5/27/2020	2:00:00 a, m,	17.8	86	0
5/27/2020	3:00:00 a, m,	17.8	86	0
5/27/2020	4:00:00 a, m,	17.8	84	0
5/27/2020	5:00:00 a, m,	17.5	87	0
5/27/2020	6:00:00 a, m,	17.3	88	0
5/27/2020	7:00:00 a, m,	17.3	89	0
5/27/2020	8:00:00 a, m,	17.8	87	0
5/27/2020	9:00:00 a, m,	18.3	87	0
5/27/2020	10:00:00 a, m	19.5	86	0
5/27/2020	11:00:00 a, m	19.4	86	0
5/27/2020	12:00:00 p, m	19.4	89	0
5/27/2020	1:00:00 p, m,	19.6	88	0
5/27/2020	2:00:00 p, m,	19.9	87	0
5/27/2020	3:00:00 p, m,	19.6	87	0
5/27/2020	4:00:00 p, m,	19.4	87	0

5/27/2020	5:00:00 p, m,	19.4	89	0
5/27/2020	6:00:00 p, m,	18.9	87	0
5/27/2020	7:00:00 p, m,	18.1	86	0
5/27/2020	8:00:00 p, m,	17.8	84	0
5/27/2020	9:00:00 p, m,	18.2	81	0
5/27/2020	10:00:00 p, m	18.3	80	0
5/27/2020	11:00:00 p, m	18.1	80	0
5/28/2020	12:00:00 a, m	18.3	81	0
5/28/2020	1:00:00 a, m,	18.3	80	0
5/28/2020	2:00:00 a, m,	17.9	82	0
5/28/2020	3:00:00 a, m,	18.1	81	0
5/28/2020	4:00:00 a, m,	17.9	82	0
5/28/2020	5:00:00 a, m,	17.8	82	0
5/28/2020	6:00:00 a, m,	17.7	83	0
5/28/2020	7:00:00 a, m,	18	84	0
5/28/2020	8:00:00 a, m,	18.2	85	0
5/28/2020	9:00:00 a, m,	18.7	85	0
5/28/2020	10:00:00 a, m	19.4	85	0
5/28/2020	11:00:00 a, m	20	86	0
5/28/2020	12:00:00 p, m	20.4	83	0
5/28/2020	1:00:00 p, m,	20.8	85	0
5/28/2020	2:00:00 p, m,	21.4	86	0
5/28/2020	3:00:00 p, m,	21.8	84	0
5/28/2020	4:00:00 p, m,	21.9	83	0
5/28/2020	5:00:00 p, m,	21.1	87	0.2
5/28/2020	6:00:00 p, m,	17.4	91	0.8
5/28/2020	7:00:00 p, m,	17.7	92	0
5/28/2020	8:00:00 p, m,	17.8	92	0
5/28/2020	9:00:00 p, m,	17.5	92	0
5/28/2020	10:00:00 p, m	17.7	91	0
5/28/2020	11:00:00 p, m	17.7	92	0
5/29/2020	12:00:00 a, m	17.6	92	0
5/29/2020	1:00:00 a, m,	17.5	92	0
5/29/2020	2:00:00 a, m,	17.4	93	0
5/29/2020	3:00:00 a, m,	17.3	95	0.6
5/29/2020	4:00:00 a, m,	16.9	96	0
5/29/2020	5:00:00 a, m,	16.9	96	0
5/29/2020	6:00:00 a, m,	16.7	96	0
5/29/2020	7:00:00 a, m,	16.7	96	0.4
5/29/2020	8:00:00 a, m,	16.8	98	0
5/29/2020	9:00:00 a, m,	17.3	98	0.2
5/29/2020	10:00:00 a, m	18.1	98	0
5/29/2020	11:00:00 a, m	17.8	97	3
5/29/2020	12:00:00 p, m	17.9	98	1.8
5/29/2020	1:00:00 p, m,	19.2	99	0
5/29/2020	2:00:00 p, m,	19.7	93	0.2
5/29/2020	3:00:00 p, m,	19.5	96	1.2

5/29/2020	4:00:00 p, m,	19.4	98	0.2
5/29/2020	5:00:00 p, m,	20.1	96	0
5/29/2020	6:00:00 p, m,	19.8	95	0
5/29/2020	7:00:00 p, m,	18.9	96	0
5/29/2020	8:00:00 p, m,	18.7	96	0
5/29/2020	9:00:00 p, m,	18.1	93	0
5/29/2020	10:00:00 p, m	17.4	93	0
5/29/2020	11:00:00 p, m	17.5	94	0
5/30/2020	12:00:00 a, m	17.4	93	0
5/30/2020	1:00:00 a, m,	17.3	93	0
5/30/2020	2:00:00 a, m,	17.4	94	0
5/30/2020	3:00:00 a, m,	17.4	95	0
5/30/2020	4:00:00 a, m,	17.3	93	0
5/30/2020	5:00:00 a, m,	17.3	94	0
5/30/2020	6:00:00 a, m,	17.3	95	0
5/30/2020	7:00:00 a, m,	17.1	94	0
5/30/2020	8:00:00 a, m,	17.1	98	0.4
5/30/2020	9:00:00 a, m,	17.4	98	0
5/30/2020	10:00:00 a, m	17.8	98	0
5/30/2020	11:00:00 a, m	17.6	98	1.2
5/30/2020	12:00:00 p, m	17.8	99	1.2
5/30/2020	1:00:00 p, m,	17.9	99	3.8
5/30/2020	2:00:00 p, m,	18.3	99	0.4
5/30/2020	3:00:00 p, m,	17.8	98	4
5/30/2020	4:00:00 p, m,	18.2	98	0.2
5/30/2020	5:00:00 p, m,	18.3	99	0
5/30/2020	6:00:00 p, m,	18.1	98	0
5/30/2020	7:00:00 p, m,	18	98	0
5/30/2020	8:00:00 p, m,	17.8	98	0
5/30/2020	9:00:00 p, m,	17.7	98	0.2
5/30/2020	10:00:00 p, m	17.4	98	0
5/30/2020	11:00:00 p, m	17.6	98	0
5/31/2020	12:00:00 a, m	17.1	98	0
5/31/2020	1:00:00 a, m,	16.8	98	0
5/31/2020	2:00:00 a, m,	16.5	98	0
5/31/2020	3:00:00 a, m,	16.6	98	0
5/31/2020	4:00:00 a, m,	16.4	98	0
5/31/2020	5:00:00 a, m,	16.2	97	0
5/31/2020	6:00:00 a, m,	16.4	98	0
5/31/2020	7:00:00 a, m,	16.6	98	0
5/31/2020	8:00:00 a, m,	17.1	98	0
5/31/2020	9:00:00 a, m,	17.5	98	0
5/31/2020	10:00:00 a, m	17.9	98	0
5/31/2020	11:00:00 a, m	18.5	97	0
5/31/2020	12:00:00 p, m	19	97	0
5/31/2020	1:00:00 p, m,	19.3	97	0
5/31/2020	2:00:00 p, m,	19.2	94	0.4

5/31/2020	3:00:00 p, m,	20	95	0.2
5/31/2020	4:00:00 p, m,	20.8	90	0
5/31/2020	5:00:00 p, m,	19.1	94	0.4
5/31/2020	6:00:00 p, m,	18.4	96	0
5/31/2020	7:00:00 p, m,	17.9	96	0
5/31/2020	8:00:00 p, m,	17.6	95	0
5/31/2020	9:00:00 p, m,	17.6	95	0
5/31/2020	10:00:00 p, m	17.7	94	0
5/31/2020	11:00:00 p, m	17.5	95	0
May_20		18.7924731	89.186828	81.2
6/1/2020	12:00:00 a, m	17.4	95	0
6/1/2020	1:00:00 a, m,	17.1	96	0.2
6/1/2020	2:00:00 a, m,	16.8	97	0
6/1/2020	3:00:00 a, m,	16.9	96	0
6/1/2020	4:00:00 a, m,	16.7	96	0
6/1/2020	5:00:00 a, m,	16.6	97	0
6/1/2020	6:00:00 a, m,	16.7	96	0
6/1/2020	7:00:00 a, m,	16.8	96	0
6/1/2020	8:00:00 a, m,	17.1	98	0
6/1/2020	9:00:00 a, m,	17.6	99	0
6/1/2020	10:00:00 a, m	17.7	99	1.2
6/1/2020	11:00:00 a, m	18.3	99	0
6/1/2020	12:00:00 p, m	18.6	98	1.6
6/1/2020	1:00:00 p, m,	19	99	0.4
6/1/2020	2:00:00 p, m,	19.6	99	0
6/1/2020	3:00:00 p, m,	19.9	96	1.4
6/1/2020	4:00:00 p, m,	18.3	97	0.2
6/1/2020	5:00:00 p, m,	17.9	96	0
6/1/2020	6:00:00 p, m,	17.6	96	0
6/1/2020	7:00:00 p, m,	17.4	96	0
6/1/2020	8:00:00 p, m,	17.5	97	0
6/1/2020	9:00:00 p, m,	17.7	96	0
6/1/2020	10:00:00 p, m	17.7	96	0
6/1/2020	11:00:00 p, m	17.7	95	0
6/2/2020	12:00:00 a, m	17.3	96	0
6/2/2020	1:00:00 a, m,	17.4	96	0
6/2/2020	2:00:00 a, m,	16.9	95	0
6/2/2020	3:00:00 a, m,	16.7	95	0
6/2/2020	4:00:00 a, m,	16.5	93	0
6/2/2020	5:00:00 a, m,	16.7	93	0
6/2/2020	6:00:00 a, m,	16.6	93	0
6/2/2020	7:00:00 a, m,	17.1	94	0
6/2/2020	8:00:00 a, m,	17.4	94	0
6/2/2020	9:00:00 a, m,	17.9	94	0
6/2/2020	10:00:00 a, m	18.9	94	0
6/2/2020	11:00:00 a, m	19.7	95	0
6/2/2020	12:00:00 p, m	19.9	92	0

6/2/2020	1:00:00 p, m,	20.4	88	0
6/2/2020	2:00:00 p, m,	19.6	91	0
6/2/2020	3:00:00 p, m,	20.1	89	0
6/2/2020	4:00:00 p, m,	19.6	91	0
6/2/2020	5:00:00 p, m,	19.4	91	0.2
6/2/2020	6:00:00 p, m,	18.9	89	0
6/2/2020	7:00:00 p, m,	18.2	89	0
6/2/2020	8:00:00 p, m,	18.1	89	0
6/2/2020	9:00:00 p, m,	17.9	88	0
6/2/2020	10:00:00 p, m	18.4	87	0
6/2/2020	11:00:00 p, m	18.1	88	0
6/3/2020	12:00:00 a, m	17.7	87	0
6/3/2020	1:00:00 a, m,	17.2	90	0
6/3/2020	2:00:00 a, m,	17	90	0
6/3/2020	3:00:00 a, m,	17	92	0
6/3/2020	4:00:00 a, m,	16.9	91	0
6/3/2020	5:00:00 a, m,	17.2	92	0
6/3/2020	6:00:00 a, m,	17.2	92	0
6/3/2020	7:00:00 a, m,	17.5	91	0
6/3/2020	8:00:00 a, m,	17.8	93	0
6/3/2020	9:00:00 a, m,	17.9	92	0
6/3/2020	10:00:00 a, m	17.9	96	0
6/3/2020	11:00:00 a, m	18.6	95	0
6/3/2020	12:00:00 p, m	18.7	97	0
6/3/2020	1:00:00 p, m,	18.6	97	0.2
6/3/2020	2:00:00 p, m,	18.2	97	0
6/3/2020	3:00:00 p, m,	17.4	97	0
6/3/2020	4:00:00 p, m,	17.7	96	0
6/3/2020	5:00:00 p, m,	17.7	96	0
6/3/2020	6:00:00 p, m,	17.6	95	0
6/3/2020	7:00:00 p, m,	17.2	95	0
6/3/2020	8:00:00 p, m,	16.9	94	0
6/3/2020	9:00:00 p, m,	17.1	94	0
6/3/2020	10:00:00 p, m	17.2	92	0
6/3/2020	11:00:00 p, m	17.1	92	0
6/4/2020	12:00:00 a, m	16.9	92	0
6/4/2020	1:00:00 a, m,	16.8	94	0
6/4/2020	2:00:00 a, m,	16.9	94	0
6/4/2020	3:00:00 a, m,	16.9	94	0
6/4/2020	4:00:00 a, m,	16.9	94	0
6/4/2020	5:00:00 a, m,	16.6	91	0
6/4/2020	6:00:00 a, m,	16.3	92	0
6/4/2020	7:00:00 a, m,	16.4	94	0
6/4/2020	8:00:00 a, m,	17.2	92	0
6/4/2020	9:00:00 a, m,	17.8	94	0
6/4/2020	10:00:00 a, m	18.7	95	0
6/4/2020	11:00:00 a, m	20.2	92	0

6/4/2020	12:00:00 p, m	21.7	89	0
6/4/2020	1:00:00 p, m,	22.6	86	0.2
6/4/2020	2:00:00 p, m,	22.3	87	0
6/4/2020	3:00:00 p, m,	21.9	88	0
6/4/2020	4:00:00 p, m,	21.9	88	0
6/4/2020	5:00:00 p, m,	20.9	79	0
6/4/2020	6:00:00 p, m,	20.6	76	0
6/4/2020	7:00:00 p, m,	20.1	75	0
6/4/2020	8:00:00 p, m,	19.3	85	0
6/4/2020	9:00:00 p, m,	19.1	81	0
6/4/2020	10:00:00 p, m	19.9	78	0
6/4/2020	11:00:00 p, m	19.8	79	0
6/5/2020	12:00:00 a, m	19.2	81	0
6/5/2020	1:00:00 a, m,	19.1	81	0
6/5/2020	2:00:00 a, m,	19.2	82	0
6/5/2020	3:00:00 a, m,	18.8	82	0
6/5/2020	4:00:00 a, m,	18.8	79	0
6/5/2020	5:00:00 a, m,	19.4	73	0
6/5/2020	6:00:00 a, m,	18.5	80	0
6/5/2020	7:00:00 a, m,	18.7	84	0
6/5/2020	8:00:00 a, m,	19.1	83	0
6/5/2020	9:00:00 a, m,	19.5	83	0
6/5/2020	10:00:00 a, m	19.6	84	0
6/5/2020	11:00:00 a, m	19.8	86	0
6/5/2020	12:00:00 p, m	20.5	84	0
6/5/2020	1:00:00 p, m,	21.5	80	0
6/5/2020	2:00:00 p, m,	21.8	80	0
6/5/2020	3:00:00 p, m,	22.4	76	0
6/5/2020	4:00:00 p, m,	22.6	80	0
6/5/2020	5:00:00 p, m,	22.3	83	0
6/5/2020	6:00:00 p, m,	20.9	86	0
6/5/2020	7:00:00 p, m,	20.5	77	0
6/5/2020	8:00:00 p, m,	19.9	77	0
6/5/2020	9:00:00 p, m,	19.4	81	0
6/5/2020	10:00:00 p, m	19.4	80	0
6/5/2020	11:00:00 p, m	19.1	84	0
6/6/2020	12:00:00 a, m	17.7	90	0
6/6/2020	1:00:00 a, m,	16.3	96	4.2
6/6/2020	2:00:00 a, m,	16.3	97	0.6
6/6/2020	3:00:00 a, m,	16.2	97	0
6/6/2020	4:00:00 a, m,	16.1	97	0
6/6/2020	5:00:00 a, m,	16.2	97	0
6/6/2020	6:00:00 a, m,	16.2	96	0
6/6/2020	7:00:00 a, m,	16.6	96	0
6/6/2020	8:00:00 a, m,	17.1	95	0
6/6/2020	9:00:00 a, m,	17.4	95	0
6/6/2020	10:00:00 a, m	17.6	96	0

6/6/2020	11:00:00 a, m	17.6	93	0
6/6/2020	12:00:00 p, m	17.4	96	0.4
6/6/2020	1:00:00 p, m,	17.1	98	3.6
6/6/2020	2:00:00 p, m,	17.8	98	0
6/6/2020	3:00:00 p, m,	18.5	97	0
6/6/2020	4:00:00 p, m,	18.8	95	0
6/6/2020	5:00:00 p, m,	18.2	94	0
6/6/2020	6:00:00 p, m,	18	95	0
6/6/2020	7:00:00 p, m,	17.6	94	0
6/6/2020	8:00:00 p, m,	17.2	92	0
6/6/2020	9:00:00 p, m,	16.9	90	0
6/6/2020	10:00:00 p, m	16.9	90	0
6/6/2020	11:00:00 p, m	16.9	91	0
6/7/2020	12:00:00 a, m	17.2	91	0
6/7/2020	1:00:00 a, m,	16.9	91	0
6/7/2020	2:00:00 a, m,	16.6	90	0
6/7/2020	3:00:00 a, m,	16.6	89	0
6/7/2020	4:00:00 a, m,	16.7	89	0
6/7/2020	5:00:00 a, m,	16.9	88	0
6/7/2020	6:00:00 a, m,	16.9	88	0
6/7/2020	7:00:00 a, m,	17.1	90	0
6/7/2020	8:00:00 a, m,	17.8	89	0
6/7/2020	9:00:00 a, m,	18.2	89	0
6/7/2020	10:00:00 a, m	20	85	0
6/7/2020	11:00:00 a, m	21.2	86	0
6/7/2020	12:00:00 p, m	21.9	83	0.4
6/7/2020	1:00:00 p, m,	21.8	76	0
6/7/2020	2:00:00 p, m,	21.5	85	0
6/7/2020	3:00:00 p, m,	22.8	75	0
6/7/2020	4:00:00 p, m,	21.7	80	0
6/7/2020	5:00:00 p, m,	18.8	91	0.2
6/7/2020	6:00:00 p, m,	18.6	85	0
6/7/2020	7:00:00 p, m,	18.7	83	0
6/7/2020	8:00:00 p, m,	19.5	78	0
6/7/2020	9:00:00 p, m,	18.8	81	0
6/7/2020	10:00:00 p, m	17.9	84	0
6/7/2020	11:00:00 p, m	17.9	83	0
6/8/2020	12:00:00 a, m	18.2	78	0
6/8/2020	1:00:00 a, m,	18.1	78	0
6/8/2020	2:00:00 a, m,	17.6	81	0
6/8/2020	3:00:00 a, m,	17.6	83	0
6/8/2020	4:00:00 a, m,	17.3	84	0
6/8/2020	5:00:00 a, m,	17.6	83	0
6/8/2020	6:00:00 a, m,	17.3	84	0
6/8/2020	7:00:00 a, m,	17	87	0
6/8/2020	8:00:00 a, m,	17.9	86	0
6/8/2020	9:00:00 a, m,	18.6	86	0

6/8/2020	10:00:00 a, m	18.9	85	0
6/8/2020	11:00:00 a, m	19.4	87	0
6/8/2020	12:00:00 p, m	20	87	0
6/8/2020	1:00:00 p, m,	20.2	88	0
6/8/2020	2:00:00 p, m,	20	86	0
6/8/2020	3:00:00 p, m,	20.4	87	0
6/8/2020	4:00:00 p, m,	20.5	86	0
6/8/2020	5:00:00 p, m,	21.3	78	0
6/8/2020	6:00:00 p, m,	20.4	74	0
6/8/2020	7:00:00 p, m,	18.8	76	0
6/8/2020	8:00:00 p, m,	18.6	76	0
6/8/2020	9:00:00 p, m,	18.5	75	0
6/8/2020	10:00:00 p, m	18.5	75	0
6/8/2020	11:00:00 p, m	18.9	71	0
6/9/2020	12:00:00 a, m	18.3	74	0
6/9/2020	1:00:00 a, m,	17.4	75	0
6/9/2020	2:00:00 a, m,	18.3	72	0
6/9/2020	3:00:00 a, m,	18.9	70	0
6/9/2020	4:00:00 a, m,	18.9	71	0
6/9/2020	5:00:00 a, m,	18.6	75	0
6/9/2020	6:00:00 a, m,	17.9	77	0
6/9/2020	7:00:00 a, m,	18.1	76	0
6/9/2020	8:00:00 a, m,	18.3	78	0
6/9/2020	9:00:00 a, m,	18.5	81	0
6/9/2020	10:00:00 a, m	19.6	80	0
6/9/2020	11:00:00 a, m	20.3	81	0
6/9/2020	12:00:00 p, m	20.8	83	0
6/9/2020	1:00:00 p, m,	21.3	83	0
6/9/2020	2:00:00 p, m,	21.9	78	0
6/9/2020	3:00:00 p, m,	21.4	81	0
6/9/2020	4:00:00 p, m,	22.5	76	0
6/9/2020	5:00:00 p, m,	22.1	70	0
6/9/2020	6:00:00 p, m,	21.4	73	0
6/9/2020	7:00:00 p, m,	20.1	72	0
6/9/2020	8:00:00 p, m,	20	71	0
6/9/2020	9:00:00 p, m,	20.2	71	0
6/9/2020	10:00:00 p, m	19.7	73	0
6/9/2020	11:00:00 p, m	19.4	71	0
6/10/2020	12:00:00 a, m	18.4	74	0
6/10/2020	1:00:00 a, m,	17.9	76	0
6/10/2020	2:00:00 a, m,	17.7	77	0
6/10/2020	3:00:00 a, m,	17.7	76	0
6/10/2020	4:00:00 a, m,	17.3	77	0
6/10/2020	5:00:00 a, m,	17.1	77	0
6/10/2020	6:00:00 a, m,	16.8	81	0
6/10/2020	7:00:00 a, m,	17.1	83	0
6/10/2020	8:00:00 a, m,	17.4	82	0

6/10/2020	9:00:00 a, m,	18.2	83	0
6/10/2020	10:00:00 a, m	19.2	85	0
6/10/2020	11:00:00 a, m	20.2	84	0
6/10/2020	12:00:00 p, m	21.7	80	0
6/10/2020	1:00:00 p, m,	22.7	74	0
6/10/2020	2:00:00 p, m,	23.1	70	0
6/10/2020	3:00:00 p, m,	22.7	76	0
6/10/2020	4:00:00 p, m,	23.8	72	0
6/10/2020	5:00:00 p, m,	22.6	79	0
6/10/2020	6:00:00 p, m,	21.1	75	0
6/10/2020	7:00:00 p, m,	19.3	82	0
6/10/2020	8:00:00 p, m,	16.3	92	0.6
6/10/2020	9:00:00 p, m,	16.3	93	0
6/10/2020	10:00:00 p, m	16.4	92	0
6/10/2020	11:00:00 p, m	16.4	92	0
6/11/2020	12:00:00 a, m	16.7	92	0
6/11/2020	1:00:00 a, m,	16.3	95	0
6/11/2020	2:00:00 a, m,	16.1	97	0.4
6/11/2020	3:00:00 a, m,	15.6	97	0.8
6/11/2020	4:00:00 a, m,	15.6	97	1.2
6/11/2020	5:00:00 a, m,	15.5	97	0.2
6/11/2020	6:00:00 a, m,	15.1	97	0
6/11/2020	7:00:00 a, m,	15.2	98	0.2
6/11/2020	8:00:00 a, m,	15.7	98	0
6/11/2020	9:00:00 a, m,	16.1	98	0
6/11/2020	10:00:00 a, m	16.9	98	0
6/11/2020	11:00:00 a, m	17.7	97	0
6/11/2020	12:00:00 p, m	18.8	95	0
6/11/2020	1:00:00 p, m,	19.1	96	0
6/11/2020	2:00:00 p, m,	19.1	94	0
6/11/2020	3:00:00 p, m,	19.3	94	0
6/11/2020	4:00:00 p, m,	20.7	92	0
6/11/2020	5:00:00 p, m,	20.3	92	0
6/11/2020	6:00:00 p, m,	20.2	93	0
6/11/2020	7:00:00 p, m,	19.2	94	0
6/11/2020	8:00:00 p, m,	18.7	94	0
6/11/2020	9:00:00 p, m,	18.4	94	0
6/11/2020	10:00:00 p, m	18.2	93	0
6/11/2020	11:00:00 p, m	17.9	93	0
6/12/2020	12:00:00 a, m	17.9	94	0
6/12/2020	1:00:00 a, m,	17.7	95	0
6/12/2020	2:00:00 a, m,	17.6	95	0
6/12/2020	3:00:00 a, m,	17.2	96	0
6/12/2020	4:00:00 a, m,	17.2	95	0
6/12/2020	5:00:00 a, m,	17.1	95	0
6/12/2020	6:00:00 a, m,	16.9	96	0
6/12/2020	7:00:00 a, m,	16.9	97	0

6/12/2020	8:00:00 a, m,	17.1	98	0
6/12/2020	9:00:00 a, m,	17.6	99	0
6/12/2020	10:00:00 a, m	17.8	97	0
6/12/2020	11:00:00 a, m	17.3	98	0
6/12/2020	12:00:00 p, m	17.6	98	0
6/12/2020	1:00:00 p, m,	17.2	98	0
6/12/2020	2:00:00 p, m,	17.4	99	0
6/12/2020	3:00:00 p, m,	17.3	99	0
6/12/2020	4:00:00 p, m,	18.1	99	0
6/12/2020	5:00:00 p, m,	18.4	98	2.4
6/12/2020	6:00:00 p, m,	17.9	98	0
6/12/2020	7:00:00 p, m,	17.3	98	0
6/12/2020	8:00:00 p, m,	16.7	97	0
6/12/2020	9:00:00 p, m,	16.7	97	0
6/12/2020	10:00:00 p, m	16.4	97	0
6/12/2020	11:00:00 p, m	16.4	96	0
6/13/2020	12:00:00 a, m	16.2	96	0
6/13/2020	1:00:00 a, m,	16.1	95	0
6/13/2020	2:00:00 a, m,	16	94	0
6/13/2020	3:00:00 a, m,	16.2	95	0
6/13/2020	4:00:00 a, m,	16.1	94	0
6/13/2020	5:00:00 a, m,	16.1	94	0
6/13/2020	6:00:00 a, m,	16.3	95	0
6/13/2020	7:00:00 a, m,	16.4	94	0
6/13/2020	8:00:00 a, m,	16.8	96	0
6/13/2020	9:00:00 a, m,	17.4	96	0
6/13/2020	10:00:00 a, m	18.2	98	0
6/13/2020	11:00:00 a, m	18.5	94	0
6/13/2020	12:00:00 p, m	18.9	95	0
6/13/2020	1:00:00 p, m,	20.3	93	0
6/13/2020	2:00:00 p, m,	20.3	86	0
6/13/2020	3:00:00 p, m,	19.9	91	0.4
6/13/2020	4:00:00 p, m,	20.4	94	0
6/13/2020	5:00:00 p, m,	20.8	92	0
6/13/2020	6:00:00 p, m,	20	92	0
6/13/2020	7:00:00 p, m,	19.1	93	0
6/13/2020	8:00:00 p, m,	18.7	90	0
6/13/2020	9:00:00 p, m,	18.3	89	0
6/13/2020	10:00:00 p, m	18.3	92	0
6/13/2020	11:00:00 p, m	18.1	89	0
6/14/2020	12:00:00 a, m	18.1	89	0
6/14/2020	1:00:00 a, m,	17.5	94	0.4
6/14/2020	2:00:00 a, m,	17.2	94	0
6/14/2020	3:00:00 a, m,	17.3	95	0
6/14/2020	4:00:00 a, m,	17.4	94	0
6/14/2020	5:00:00 a, m,	17	95	0
6/14/2020	6:00:00 a, m,	16.8	96	0

6/14/2020	7:00:00 a, m,	17.4	96	0
6/14/2020	8:00:00 a, m,	17.4	94	0
6/14/2020	9:00:00 a, m,	18.1	93	0
6/14/2020	10:00:00 a, m	18.6	93	0
6/14/2020	11:00:00 a, m	18.8	93	0
6/14/2020	12:00:00 p, m	18	96	3.8
6/14/2020	1:00:00 p, m,	16.3	96	0.6
6/14/2020	2:00:00 p, m,	16.4	98	0
6/14/2020	3:00:00 p, m,	16.7	98	0.2
6/14/2020	4:00:00 p, m,	17.6	98	0.2
6/14/2020	5:00:00 p, m,	17.3	95	0
6/14/2020	6:00:00 p, m,	17	96	0
6/14/2020	7:00:00 p, m,	16.7	95	0
6/14/2020	8:00:00 p, m,	16.4	91	0
6/14/2020	9:00:00 p, m,	16.6	89	0
6/14/2020	10:00:00 p, m	16.6	87	0
6/14/2020	11:00:00 p, m	16.8	88	0
6/15/2020	12:00:00 a, m	16.8	88	0
6/15/2020	1:00:00 a, m,	16.8	87	0
6/15/2020	2:00:00 a, m,	16.9	89	0
6/15/2020	3:00:00 a, m,	16.9	89	0
6/15/2020	4:00:00 a, m,	16.6	91	0
6/15/2020	5:00:00 a, m,	16.3	91	0
6/15/2020	6:00:00 a, m,	16.3	90	0
6/15/2020	7:00:00 a, m,	16.7	92	0
6/15/2020	8:00:00 a, m,	17.1	92	0
6/15/2020	9:00:00 a, m,	17.6	90	0
6/15/2020	10:00:00 a, m	18	92	0.4
6/15/2020	11:00:00 a, m	18.7	91	0
6/15/2020	12:00:00 p, m	18.8	92	0
6/15/2020	1:00:00 p, m,	18.6	95	1.2
6/15/2020	2:00:00 p, m,	18.6	97	2
6/15/2020	3:00:00 p, m,	18.8	97	0.2
6/15/2020	4:00:00 p, m,	19	98	0
6/15/2020	5:00:00 p, m,	19.3	96	0
6/15/2020	6:00:00 p, m,	18.9	95	0
6/15/2020	7:00:00 p, m,	17.1	92	0
6/15/2020	8:00:00 p, m,	16.7	92	0
6/15/2020	9:00:00 p, m,	16.8	90	0
6/15/2020	10:00:00 p, m	16.6	89	0
6/15/2020	11:00:00 p, m	17.1	87	0
6/16/2020	12:00:00 a, m	17.6	84	0
6/16/2020	1:00:00 a, m,	17.2	84	0
6/16/2020	2:00:00 a, m,	17.1	84	0
6/16/2020	3:00:00 a, m,	16.7	86	0
6/16/2020	4:00:00 a, m,	16.4	86	0
6/16/2020	5:00:00 a, m,	16.7	86	0

6/16/2020	6:00:00 a, m,	16.2	87	0
6/16/2020	7:00:00 a, m,	16.7	87	0
6/16/2020	8:00:00 a, m,	17.3	88	0
6/16/2020	9:00:00 a, m,	18.1	88	0
6/16/2020	10:00:00 a, m	18.7	89	0
6/16/2020	11:00:00 a, m	19.6	88	0
6/16/2020	12:00:00 p, m	20.2	87	0
6/16/2020	1:00:00 p, m,	20.8	85	0
6/16/2020	2:00:00 p, m,	21.6	83	0
6/16/2020	3:00:00 p, m,	21.1	85	0
6/16/2020	4:00:00 p, m,	21.7	83	0
6/16/2020	5:00:00 p, m,	20.9	83	0
6/16/2020	6:00:00 p, m,	20.4	83	0
6/16/2020	7:00:00 p, m,	19.2	83	0
6/16/2020	8:00:00 p, m,	19.4	79	0
6/16/2020	9:00:00 p, m,	18.8	80	0
6/16/2020	10:00:00 p, m	18.6	82	0
6/16/2020	11:00:00 p, m	17.3	91	1
6/17/2020	12:00:00 a, m	16	95	1.6
6/17/2020	1:00:00 a, m,	15.9	96	0.4
6/17/2020	2:00:00 a, m,	15.7	96	0.2
6/17/2020	3:00:00 a, m,	15.8	95	0
6/17/2020	4:00:00 a, m,	15.9	94	0
6/17/2020	5:00:00 a, m,	16	94	0
6/17/2020	6:00:00 a, m,	16.2	93	0
6/17/2020	7:00:00 a, m,	16.5	93	0
6/17/2020	8:00:00 a, m,	17	94	0
6/17/2020	9:00:00 a, m,	17.6	94	0
6/17/2020	10:00:00 a, m	17.8	95	0
6/17/2020	11:00:00 a, m	18.4	94	0
6/17/2020	12:00:00 p, m	18.9	95	0
6/17/2020	1:00:00 p, m,	19.4	94	0
6/17/2020	2:00:00 p, m,	19.7	94	0
6/17/2020	3:00:00 p, m,	18.9	96	1
6/17/2020	4:00:00 p, m,	17.6	95	3.2
6/17/2020	5:00:00 p, m,	16.7	97	1.2
6/17/2020	6:00:00 p, m,	16.5	98	0
6/17/2020	7:00:00 p, m,	16.4	98	0
6/17/2020	8:00:00 p, m,	16.3	98	0.2
6/17/2020	9:00:00 p, m,	16.4	98	0
6/17/2020	10:00:00 p, m	16.2	97	0.2
6/17/2020	11:00:00 p, m	16.1	97	0
6/18/2020	12:00:00 a, m	15.9	97	0
6/18/2020	1:00:00 a, m,	15.7	96	0
6/18/2020	2:00:00 a, m,	16	96	0
6/18/2020	3:00:00 a, m,	16.3	94	0
6/18/2020	4:00:00 a, m,	16.4	95	0

6/18/2020	5:00:00 a, m,	16.4	94	0
6/18/2020	6:00:00 a, m,	16.4	93	0
6/18/2020	7:00:00 a, m,	16.8	94	0
6/18/2020	8:00:00 a, m,	17.4	94	0
6/18/2020	9:00:00 a, m,	18.1	95	0
6/18/2020	10:00:00 a, m	18.6	90	0
6/18/2020	11:00:00 a, m	19.2	93	0
6/18/2020	12:00:00 p, m	19.4	92	0.2
6/18/2020	1:00:00 p, m,	20.5	93	0
6/18/2020	2:00:00 p, m,	21.7	91	0
6/18/2020	3:00:00 p, m,	22.4	88	0
6/18/2020	4:00:00 p, m,	22.1	89	0
6/18/2020	5:00:00 p, m,	22	85	0
6/18/2020	6:00:00 p, m,	20.7	90	0
6/18/2020	7:00:00 p, m,	19.5	89	0
6/18/2020	8:00:00 p, m,	19.2	87	0
6/18/2020	9:00:00 p, m,	18.9	88	0
6/18/2020	10:00:00 p, m	18.6	92	0
6/18/2020	11:00:00 p, m	17.8	93	0
6/19/2020	12:00:00 a, m	17.6	93	0
6/19/2020	1:00:00 a, m,	17.8	94	0
6/19/2020	2:00:00 a, m,	17.7	93	0
6/19/2020	3:00:00 a, m,	17.4	94	0
6/19/2020	4:00:00 a, m,	17.4	95	0
6/19/2020	5:00:00 a, m,	17.2	95	0
6/19/2020	6:00:00 a, m,	17.2	96	0
6/19/2020	7:00:00 a, m,	17.3	95	0
6/19/2020	8:00:00 a, m,	17.4	97	0.4
6/19/2020	9:00:00 a, m,	17.6	96	0
6/19/2020	10:00:00 a, m	18.3	96	0
6/19/2020	11:00:00 a, m	19.1	94	0
6/19/2020	12:00:00 p, m	20.2	91	0
6/19/2020	1:00:00 p, m,	20.3	91	0
6/19/2020	2:00:00 p, m,	20.3	94	0.4
6/19/2020	3:00:00 p, m,	20.3	94	0
6/19/2020	4:00:00 p, m,	20.3	91	0
6/19/2020	5:00:00 p, m,	19.7	93	0
6/19/2020	6:00:00 p, m,	19.2	94	0
6/19/2020	7:00:00 p, m,	18.6	94	0
6/19/2020	8:00:00 p, m,	18.5	94	0
6/19/2020	9:00:00 p, m,	18.4	92	0
6/19/2020	10:00:00 p, m	18.4	92	0
6/19/2020	11:00:00 p, m	18.3	90	0
6/20/2020	12:00:00 a, m	18.2	90	0
6/20/2020	1:00:00 a, m,	18.3	88	0
6/20/2020	2:00:00 a, m,	18	89	0
6/20/2020	3:00:00 a, m,	17.9	88	0

6/20/2020	4:00:00 a, m,	17.6	89	0
6/20/2020	5:00:00 a, m,	17.7	89	0
6/20/2020	6:00:00 a, m,	17.4	89	0
6/20/2020	7:00:00 a, m,	17.8	90	0
6/20/2020	8:00:00 a, m,	18.3	91	0
6/20/2020	9:00:00 a, m,	18.9	90	0
6/20/2020	10:00:00 a, m	19.2	89	0
6/20/2020	11:00:00 a, m	18.6	96	1.2
6/20/2020	12:00:00 p, m	18.6	96	2.2
6/20/2020	1:00:00 p, m,	18.2	97	0.2
6/20/2020	2:00:00 p, m,	17.3	97	3.8
6/20/2020	3:00:00 p, m,	18	98	0.6
6/20/2020	4:00:00 p, m,	18.4	98	0
6/20/2020	5:00:00 p, m,	18.6	96	0
6/20/2020	6:00:00 p, m,	18.7	96	0
6/20/2020	7:00:00 p, m,	16.9	97	0.2
6/20/2020	8:00:00 p, m,	17.3	96	0
6/20/2020	9:00:00 p, m,	16.9	97	0
6/20/2020	10:00:00 p, m	16.9	90	0
6/20/2020	11:00:00 p, m	16.9	88	0
6/21/2020	12:00:00 a, m	16.9	90	0
6/21/2020	1:00:00 a, m,	17	89	0
6/21/2020	2:00:00 a, m,	17.3	86	0
6/21/2020	3:00:00 a, m,	17.1	86	0
6/21/2020	4:00:00 a, m,	16.8	88	0
6/21/2020	5:00:00 a, m,	16.8	88	0
6/21/2020	6:00:00 a, m,	16.7	89	0
6/21/2020	7:00:00 a, m,	17.1	90	0
6/21/2020	8:00:00 a, m,	17.7	90	0
6/21/2020	9:00:00 a, m,	18.1	89	0
6/21/2020	10:00:00 a, m	18.9	90	0
6/21/2020	11:00:00 a, m	19.1	91	0
6/21/2020	12:00:00 p, m	19.3	88	0
6/21/2020	1:00:00 p, m,	19.3	89	0
6/21/2020	2:00:00 p, m,	19.1	91	0
6/21/2020	3:00:00 p, m,	18.8	94	0.4
6/21/2020	4:00:00 p, m,	19.7	89	0
6/21/2020	5:00:00 p, m,	20.1	89	0
6/21/2020	6:00:00 p, m,	20.2	90	0
6/21/2020	7:00:00 p, m,	18.2	87	0
6/21/2020	8:00:00 p, m,	18.2	88	0
6/21/2020	9:00:00 p, m,	18.3	85	0
6/21/2020	10:00:00 p, m	17.7	91	0.2
6/21/2020	11:00:00 p, m	16.4	95	1
6/22/2020	12:00:00 a, m	16.1	97	1
6/22/2020	1:00:00 a, m,	16.1	98	1.2
6/22/2020	2:00:00 a, m,	15.9	98	1

6/22/2020	3:00:00 a, m,	15.7	98	1.4
6/22/2020	4:00:00 a, m,	15.7	98	0
6/22/2020	5:00:00 a, m,	15.7	98	0
6/22/2020	6:00:00 a, m,	15.7	98	0
6/22/2020	7:00:00 a, m,	15.9	98	0
6/22/2020	8:00:00 a, m,	16.3	98	0
6/22/2020	9:00:00 a, m,	16.7	98	0
6/22/2020	10:00:00 a, m	17.2	98	0
6/22/2020	11:00:00 a, m	17.9	98	0.2
6/22/2020	12:00:00 p, m	18.7	98	0
6/22/2020	1:00:00 p, m,	19	98	0
6/22/2020	2:00:00 p, m,	19.4	97	0
6/22/2020	3:00:00 p, m,	20.9	92	0
6/22/2020	4:00:00 p, m,	20.3	93	0
6/22/2020	5:00:00 p, m,	19.9	95	0
6/22/2020	6:00:00 p, m,	19.7	93	0
6/22/2020	7:00:00 p, m,	18.9	94	0
6/22/2020	8:00:00 p, m,	18.7	95	0
6/22/2020	9:00:00 p, m,	18.1	95	0
6/22/2020	10:00:00 p, m	18.2	95	0
6/22/2020	11:00:00 p, m	18	94	0
6/23/2020	12:00:00 a, m	17.9	95	0
6/23/2020	1:00:00 a, m,	17.8	95	0
6/23/2020	2:00:00 a, m,	17.8	95	0
6/23/2020	3:00:00 a, m,	17.6	95	0
6/23/2020	4:00:00 a, m,	17.2	93	0
6/23/2020	5:00:00 a, m,	17.1	91	0
6/23/2020	6:00:00 a, m,	16.9	93	0
6/23/2020	7:00:00 a, m,	17.2	92	0
6/23/2020	8:00:00 a, m,	17.9	90	0
6/23/2020	9:00:00 a, m,	18.7	89	0
6/23/2020	10:00:00 a, m	19	91	0
6/23/2020	11:00:00 a, m	19.9	88	0
6/23/2020	12:00:00 p, m	21.6	86	0
6/23/2020	1:00:00 p, m,	21.2	83	0
6/23/2020	2:00:00 p, m,	21.3	82	0
6/23/2020	3:00:00 p, m,	20	88	0
6/23/2020	4:00:00 p, m,	20.8	85	0
6/23/2020	5:00:00 p, m,	20.4	83	0
6/23/2020	6:00:00 p, m,	19.4	87	0
6/23/2020	7:00:00 p, m,	18.8	85	0
6/23/2020	8:00:00 p, m,	18.2	85	0
6/23/2020	9:00:00 p, m,	18.3	88	0
6/23/2020	10:00:00 p, m	18.1	86	0
6/23/2020	11:00:00 p, m	18.2	87	0
6/24/2020	12:00:00 a, m	18.3	86	0
6/24/2020	1:00:00 a, m,	17.9	85	0

6/24/2020	2:00:00 a, m,	17.6	88	0
6/24/2020	3:00:00 a, m,	17.6	88	0
6/24/2020	4:00:00 a, m,	17.7	88	0
6/24/2020	5:00:00 a, m,	17.4	88	0
6/24/2020	6:00:00 a, m,	17	89	0
6/24/2020	7:00:00 a, m,	17.2	90	0
6/24/2020	8:00:00 a, m,	17.7	89	0
6/24/2020	9:00:00 a, m,	18.6	91	0
6/24/2020	10:00:00 a, m	19.3	88	0
6/24/2020	11:00:00 a, m	20.1	85	0
6/24/2020	12:00:00 p, m	21.1	85	0
6/24/2020	1:00:00 p, m,	21.6	84	0
6/24/2020	2:00:00 p, m,	21.4	86	0
6/24/2020	3:00:00 p, m,	20.9	86	0
6/24/2020	4:00:00 p, m,	21.4	84	0
6/24/2020	5:00:00 p, m,	22.1	78	0
6/24/2020	6:00:00 p, m,	20.2	82	0
6/24/2020	7:00:00 p, m,	18.8	84	0
6/24/2020	8:00:00 p, m,	18.6	85	0
6/24/2020	9:00:00 p, m,	18.3	85	0
6/24/2020	10:00:00 p, m	18.1	86	0
6/24/2020	11:00:00 p, m	18.1	86	0
6/25/2020	12:00:00 a, m	17.8	89	0
6/25/2020	1:00:00 a, m,	17.7	89	0
6/25/2020	2:00:00 a, m,	18	89	0
6/25/2020	3:00:00 a, m,	18.1	88	0
6/25/2020	4:00:00 a, m,	18.1	89	0
6/25/2020	5:00:00 a, m,	18.2	89	0
6/25/2020	6:00:00 a, m,	17.9	89	0
6/25/2020	7:00:00 a, m,	18	91	0
6/25/2020	8:00:00 a, m,	18.3	90	0
6/25/2020	9:00:00 a, m,	18.6	92	0
6/25/2020	10:00:00 a, m	18.9	93	0
6/25/2020	11:00:00 a, m	19.6	92	0
6/25/2020	12:00:00 p, m	20.6	85	0
6/25/2020	1:00:00 p, m,	21.8	82	0
6/25/2020	2:00:00 p, m,	21.2	84	0
6/25/2020	3:00:00 p, m,	21.9	85	0
6/25/2020	4:00:00 p, m,	22.4	82	0
6/25/2020	5:00:00 p, m,	22.1	83	0
6/25/2020	6:00:00 p, m,	21.1	84	0.2
6/25/2020	7:00:00 p, m,	19.9	85	0
6/25/2020	8:00:00 p, m,	19.6	82	0
6/25/2020	9:00:00 p, m,	19.3	83	0
6/25/2020	10:00:00 p, m	19.1	84	0
6/25/2020	11:00:00 p, m	19.1	85	0
6/26/2020	12:00:00 a, m	18.9	85	0

6/26/2020	1:00:00 a, m,	18.8	85	0
6/26/2020	2:00:00 a, m,	18.5	85	0
6/26/2020	3:00:00 a, m,	18.2	85	0
6/26/2020	4:00:00 a, m,	18.3	82	0
6/26/2020	5:00:00 a, m,	18.4	86	0
6/26/2020	6:00:00 a, m,	18.2	87	0
6/26/2020	7:00:00 a, m,	17.9	89	0
6/26/2020	8:00:00 a, m,	18.4	88	0
6/26/2020	9:00:00 a, m,	18.8	88	0
6/26/2020	10:00:00 a, m	19.5	87	0
6/26/2020	11:00:00 a, m	19.9	87	0
6/26/2020	12:00:00 p, m	19.2	89	0.4
6/26/2020	1:00:00 p, m,	20.6	87	0
6/26/2020	2:00:00 p, m,	21.2	86	0
6/26/2020	3:00:00 p, m,	21.7	81	0
6/26/2020	4:00:00 p, m,	19.4	91	0.6
6/26/2020	5:00:00 p, m,	17.8	95	0.2
6/26/2020	6:00:00 p, m,	17.8	93	0
6/26/2020	7:00:00 p, m,	17.3	92	0
6/26/2020	8:00:00 p, m,	17.3	92	0
6/26/2020	9:00:00 p, m,	17.8	91	0
6/26/2020	10:00:00 p, m	17.6	91	0
6/26/2020	11:00:00 p, m	17.6	91	0
6/27/2020	12:00:00 a, m	17.8	89	0
6/27/2020	1:00:00 a, m,	17.7	90	0
6/27/2020	2:00:00 a, m,	17.6	90	0
6/27/2020	3:00:00 a, m,	16.8	92	0
6/27/2020	4:00:00 a, m,	16.9	93	0
6/27/2020	5:00:00 a, m,	16.4	94	0.2
6/27/2020	6:00:00 a, m,	16.3	94	0
6/27/2020	7:00:00 a, m,	16.2	94	0
6/27/2020	8:00:00 a, m,	17.1	93	0
6/27/2020	9:00:00 a, m,	17.6	93	0
6/27/2020	10:00:00 a, m	18.3	89	0
6/27/2020	11:00:00 a, m	19.2	88	0
6/27/2020	12:00:00 p, m	20.2	85	0
6/27/2020	1:00:00 p, m,	20.4	85	0
6/27/2020	2:00:00 p, m,	18.3	90	4.6
6/27/2020	3:00:00 p, m,	18.8	99	0.4
6/27/2020	4:00:00 p, m,	19.4	97	0
6/27/2020	5:00:00 p, m,	19.9	96	0
6/27/2020	6:00:00 p, m,	19.7	96	0
6/27/2020	7:00:00 p, m,	17.9	95	0
6/27/2020	8:00:00 p, m,	17.5	94	0
6/27/2020	9:00:00 p, m,	17	92	0
6/27/2020	10:00:00 p, m	16.7	91	0
6/27/2020	11:00:00 p, m	17.1	92	0

6/28/2020	12:00:00 a, m	17.2	91	0
6/28/2020	1:00:00 a, m,	17.3	90	0
6/28/2020	2:00:00 a, m,	17.2	89	0
6/28/2020	3:00:00 a, m,	17.2	89	0
6/28/2020	4:00:00 a, m,	17.1	90	0
6/28/2020	5:00:00 a, m,	16.5	93	0
6/28/2020	6:00:00 a, m,	15.8	96	0.2
6/28/2020	7:00:00 a, m,	16.3	95	0
6/28/2020	8:00:00 a, m,	16.8	96	0
6/28/2020	9:00:00 a, m,	17.5	95	0
6/28/2020	10:00:00 a, m	17.9	95	0
6/28/2020	11:00:00 a, m	18.7	92	0
6/28/2020	12:00:00 p, m	17.9	96	0.4
6/28/2020	1:00:00 p, m,	18	95	1.4
6/28/2020	2:00:00 p, m,	17.5	96	0
6/28/2020	3:00:00 p, m,	17.1	97	1.4
6/28/2020	4:00:00 p, m,	17.3	98	0
6/28/2020	5:00:00 p, m,	17.4	98	0
6/28/2020	6:00:00 p, m,	16.8	96	0
6/28/2020	7:00:00 p, m,	16.4	96	0
6/28/2020	8:00:00 p, m,	16.2	96	0
6/28/2020	9:00:00 p, m,	16.4	95	0
6/28/2020	10:00:00 p, m	16.4	95	0
6/28/2020	11:00:00 p, m	16.4	95	0
6/29/2020	12:00:00 a, m	16.6	96	0
6/29/2020	1:00:00 a, m,	16.5	96	0
6/29/2020	2:00:00 a, m,	16	97	0.8
6/29/2020	3:00:00 a, m,	15.8	98	0.2
6/29/2020	4:00:00 a, m,	15.7	98	0
6/29/2020	5:00:00 a, m,	15.6	97	0
6/29/2020	6:00:00 a, m,	15.6	97	0
6/29/2020	7:00:00 a, m,	15.9	97	0
6/29/2020	8:00:00 a, m,	16.5	96	0
6/29/2020	9:00:00 a, m,	17	96	0
6/29/2020	10:00:00 a, m	17.5	95	0
6/29/2020	11:00:00 a, m	18.3	97	0
6/29/2020	12:00:00 p, m	18.9	94	0
6/29/2020	1:00:00 p, m,	19.3	93	0
6/29/2020	2:00:00 p, m,	20.2	92	0
6/29/2020	3:00:00 p, m,	21.5	85	0
6/29/2020	4:00:00 p, m,	20.9	90	0
6/29/2020	5:00:00 p, m,	21.2	85	0
6/29/2020	6:00:00 p, m,	20.1	83	0
6/29/2020	7:00:00 p, m,	18.8	83	0
6/29/2020	8:00:00 p, m,	17.6	84	0
6/29/2020	9:00:00 p, m,	17.3	85	0
6/29/2020	10:00:00 p, m	18	81	0

6/29/2020	11:00:00 p, m	17.8	80	0
6/30/2020	12:00:00 a, m	17.1	84	0
6/30/2020	1:00:00 a, m,	17.3	82	0
6/30/2020	2:00:00 a, m,	16.8	85	0
6/30/2020	3:00:00 a, m,	16.3	85	0
6/30/2020	4:00:00 a, m,	16.3	86	0
6/30/2020	5:00:00 a, m,	16.1	86	0
6/30/2020	6:00:00 a, m,	16	85	0
6/30/2020	7:00:00 a, m,	16.1	87	0
6/30/2020	8:00:00 a, m,	16.4	88	0
6/30/2020	9:00:00 a, m,	17.3	87	0
6/30/2020	10:00:00 a, m	18.6	87	0
6/30/2020	11:00:00 a, m	19.3	88	0
6/30/2020	12:00:00 p, m	20.2	84	0
6/30/2020	1:00:00 p, m,	20.8	85	0
6/30/2020	2:00:00 p, m,	21.8	80	0
6/30/2020	3:00:00 p, m,	20.8	84	0
6/30/2020	4:00:00 p, m,	21.7	82	0
6/30/2020	5:00:00 p, m,	22.3	80	0
6/30/2020	6:00:00 p, m,	21.4	82	0
6/30/2020	7:00:00 p, m,	19.4	85	0
6/30/2020	8:00:00 p, m,	19.3	82	0
6/30/2020	9:00:00 p, m,	19.2	83	0
6/30/2020	10:00:00 p, m	19.1	83	0
6/30/2020	11:00:00 p, m	18.7	83	0
Jun_20		18.2691667	89.8847222	65.8
7/1/2020	12:00:00 a, m	18.1	83	0
7/1/2020	1:00:00 a, m,	18.2	84	0
7/1/2020	2:00:00 a, m,	18.2	85	0
7/1/2020	3:00:00 a, m,	18.4	84	0
7/1/2020	4:00:00 a, m,	18.3	85	0
7/1/2020	5:00:00 a, m,	17.6	86	0
7/1/2020	6:00:00 a, m,	16.7	89	0
7/1/2020	7:00:00 a, m,	16.9	90	0
7/1/2020	8:00:00 a, m,	17.2	92	0
7/1/2020	9:00:00 a, m,	17.9	92	0
7/1/2020	10:00:00 a, m	19.6	88	0
7/1/2020	11:00:00 a, m	19.7	89	0
7/1/2020	12:00:00 p, m	20.3	87	0
7/1/2020	1:00:00 p, m,	21.2	82	0
7/1/2020	2:00:00 p, m,	20.8	87	0
7/1/2020	3:00:00 p, m,	21.2	85	0
7/1/2020	4:00:00 p, m,	18.6	94	0.8
7/1/2020	5:00:00 p, m,	19	93	0
7/1/2020	6:00:00 p, m,	19.3	93	0
7/1/2020	7:00:00 p, m,	18.6	94	0
7/1/2020	8:00:00 p, m,	18.2	92	0

7/1/2020	9:00:00 p, m,	18.2	93	0
7/1/2020	10:00:00 p, m	17.6	96	0.8
7/1/2020	11:00:00 p, m	17.2	98	0.8
7/2/2020	12:00:00 a, m	17.2	98	0.6
7/2/2020	1:00:00 a, m,	16.4	97	0.4
7/2/2020	2:00:00 a, m,	15.8	97	4.8
7/2/2020	3:00:00 a, m,	15.5	97	0
7/2/2020	4:00:00 a, m,	15.3	98	0
7/2/2020	5:00:00 a, m,	15.4	97	0
7/2/2020	6:00:00 a, m,	15.7	98	0
7/2/2020	7:00:00 a, m,	15.6	98	0
7/2/2020	8:00:00 a, m,	16.2	98	0
7/2/2020	9:00:00 a, m,	16.7	98	0
7/2/2020	10:00:00 a, m	17.2	98	0
7/2/2020	11:00:00 a, m	17.8	98	0.2
7/2/2020	12:00:00 p, m	18.1	99	0.4
7/2/2020	1:00:00 p, m,	18.2	94	0.4
7/2/2020	2:00:00 p, m,	19.2	95	0
7/2/2020	3:00:00 p, m,	20.2	96	0
7/2/2020	4:00:00 p, m,	19.3	94	0
7/2/2020	5:00:00 p, m,	19.1	96	0
7/2/2020	6:00:00 p, m,	19.2	93	0
7/2/2020	7:00:00 p, m,	17.8	94	0
7/2/2020	8:00:00 p, m,	16.9	94	0
7/2/2020	9:00:00 p, m,	16.9	95	0
7/2/2020	10:00:00 p, m	16.8	94	0
7/2/2020	11:00:00 p, m	16.7	91	0
7/3/2020	12:00:00 a, m	16.5	90	0
7/3/2020	1:00:00 a, m,	16.3	90	0
7/3/2020	2:00:00 a, m,	16.3	91	0
7/3/2020	3:00:00 a, m,	16.6	92	0
7/3/2020	4:00:00 a, m,	16.6	91	0
7/3/2020	5:00:00 a, m,	16.5	91	0
7/3/2020	6:00:00 a, m,	16.4	93	0
7/3/2020	7:00:00 a, m,	16.7	93	0
7/3/2020	8:00:00 a, m,	17.3	94	0
7/3/2020	9:00:00 a, m,	17.7	94	0
7/3/2020	10:00:00 a, m	18.2	95	0
7/3/2020	11:00:00 a, m	18.7	93	0
7/3/2020	12:00:00 p, m	19.1	94	0
7/3/2020	1:00:00 p, m,	20.4	86	0
7/3/2020	2:00:00 p, m,	21.1	81	0
7/3/2020	3:00:00 p, m,	22.1	78	0
7/3/2020	4:00:00 p, m,	22	79	0
7/3/2020	5:00:00 p, m,	21.9	79	0
7/3/2020	6:00:00 p, m,	21.3	82	0
7/3/2020	7:00:00 p, m,	19.6	89	0

7/3/2020	8:00:00 p, m,	18.1	93	0.2
7/3/2020	9:00:00 p, m,	16.6	96	0.6
7/3/2020	10:00:00 p, m	16.8	94	0
7/3/2020	11:00:00 p, m	17.2	96	0
7/4/2020	12:00:00 a, m	17.2	95	0.2
7/4/2020	1:00:00 a, m,	16.7	96	0
7/4/2020	2:00:00 a, m,	16.7	97	0
7/4/2020	3:00:00 a, m,	16.7	96	0
7/4/2020	4:00:00 a, m,	16.4	97	0.2
7/4/2020	5:00:00 a, m,	16.2	97	1
7/4/2020	6:00:00 a, m,	15.9	97	1.4
7/4/2020	7:00:00 a, m,	15.8	98	1.8
7/4/2020	8:00:00 a, m,	15.9	98	1.4
7/4/2020	9:00:00 a, m,	16.1	98	1
7/4/2020	10:00:00 a, m	16.2	98	1
7/4/2020	11:00:00 a, m	16.4	99	0.6
7/4/2020	12:00:00 p, m	17.1	99	0.2
7/4/2020	1:00:00 p, m,	18.3	99	0.2
7/4/2020	2:00:00 p, m,	18.4	99	0
7/4/2020	3:00:00 p, m,	18.3	99	0
7/4/2020	4:00:00 p, m,	18.9	99	0.2
7/4/2020	5:00:00 p, m,	18.7	98	0
7/4/2020	6:00:00 p, m,	18	98	3.6
7/4/2020	7:00:00 p, m,	16.8	98	0.8
7/4/2020	8:00:00 p, m,	16.4	98	0.2
7/4/2020	9:00:00 p, m,	15.9	98	0
7/4/2020	10:00:00 p, m	15.7	98	0
7/4/2020	11:00:00 p, m	15.6	97	0
7/5/2020	12:00:00 a, m	15.9	97	0
7/5/2020	1:00:00 a, m,	15.8	97	0
7/5/2020	2:00:00 a, m,	15.5	96	0
7/5/2020	3:00:00 a, m,	15.3	95	0
7/5/2020	4:00:00 a, m,	15.3	95	0
7/5/2020	5:00:00 a, m,	15.6	97	0
7/5/2020	6:00:00 a, m,	15.7	95	0
7/5/2020	7:00:00 a, m,	15.9	95	0
7/5/2020	8:00:00 a, m,	16.3	96	0
7/5/2020	9:00:00 a, m,	16.9	97	0
7/5/2020	10:00:00 a, m	17.8	98	0
7/5/2020	11:00:00 a, m	17.8	98	0.2
7/5/2020	12:00:00 p, m	17.8	98	1
7/5/2020	1:00:00 p, m,	19	99	0.2
7/5/2020	2:00:00 p, m,	18.8	98	0.6
7/5/2020	3:00:00 p, m,	18.9	99	0.2
7/5/2020	4:00:00 p, m,	19.5	98	0
7/5/2020	5:00:00 p, m,	19.6	98	0
7/5/2020	6:00:00 p, m,	19.6	98	0

7/5/2020	7:00:00 p, m,	18.5	97	0
7/5/2020	8:00:00 p, m,	17.9	97	0
7/5/2020	9:00:00 p, m,	17.6	98	0
7/5/2020	10:00:00 p, m	17.2	96	0
7/5/2020	11:00:00 p, m	17	97	0
7/6/2020	12:00:00 a, m	16.8	97	0
7/6/2020	1:00:00 a, m,	16.7	96	0
7/6/2020	2:00:00 a, m,	16.6	97	0.2
7/6/2020	3:00:00 a, m,	16.2	97	0
7/6/2020	4:00:00 a, m,	16.3	96	0
7/6/2020	5:00:00 a, m,	16.3	97	0
7/6/2020	6:00:00 a, m,	16.2	97	0
7/6/2020	7:00:00 a, m,	16.1	97	0
7/6/2020	8:00:00 a, m,	16.5	97	0
7/6/2020	9:00:00 a, m,	17.4	98	0
7/6/2020	10:00:00 a, m	17.3	98	0
7/6/2020	11:00:00 a, m	17.4	98	0
7/6/2020	12:00:00 p, m	18.4	97	0
7/6/2020	1:00:00 p, m,	19.5	92	0
7/6/2020	2:00:00 p, m,	20	93	0
7/6/2020	3:00:00 p, m,	20.4	93	0
7/6/2020	4:00:00 p, m,	20.8	91	0
7/6/2020	5:00:00 p, m,	20.1	94	0
7/6/2020	6:00:00 p, m,	19.3	95	0
7/6/2020	7:00:00 p, m,	18.4	93	0
7/6/2020	8:00:00 p, m,	17.8	94	0
7/6/2020	9:00:00 p, m,	17.6	93	0
7/6/2020	10:00:00 p, m	17.4	93	0
7/6/2020	11:00:00 p, m	17.4	94	0
7/7/2020	12:00:00 a, m	17.2	94	0
7/7/2020	1:00:00 a, m,	17.1	94	0
7/7/2020	2:00:00 a, m,	17.1	92	0
7/7/2020	3:00:00 a, m,	17.1	93	0
7/7/2020	4:00:00 a, m,	17.2	93	0
7/7/2020	5:00:00 a, m,	17.1	94	0
7/7/2020	6:00:00 a, m,	16.8	93	0
7/7/2020	7:00:00 a, m,	16.9	94	0
7/7/2020	8:00:00 a, m,	17.4	95	0
7/7/2020	9:00:00 a, m,	17.9	95	0
7/7/2020	10:00:00 a, m	18.3	95	0
7/7/2020	11:00:00 a, m	18.9	94	0
7/7/2020	12:00:00 p, m	19.3	95	0
7/7/2020	1:00:00 p, m,	19.5	92	0
7/7/2020	2:00:00 p, m,	20.2	89	0
7/7/2020	3:00:00 p, m,	21.8	80	0
7/7/2020	4:00:00 p, m,	21.3	84	0
7/7/2020	5:00:00 p, m,	20.9	84	0

7/7/2020	6:00:00 p, m,	20.8	81	0
7/7/2020	7:00:00 p, m,	18.9	83	0
7/7/2020	8:00:00 p, m,	18.3	81	0
7/7/2020	9:00:00 p, m,	17.8	83	0
7/7/2020	10:00:00 p, m	17.1	86	0
7/7/2020	11:00:00 p, m	17.1	88	0
7/8/2020	12:00:00 a, m	16.9	86	0
7/8/2020	1:00:00 a, m,	16.9	84	0
7/8/2020	2:00:00 a, m,	16.9	87	0
7/8/2020	3:00:00 a, m,	17.1	86	0
7/8/2020	4:00:00 a, m,	16.9	86	0
7/8/2020	5:00:00 a, m,	16.8	88	0
7/8/2020	6:00:00 a, m,	16.4	88	0
7/8/2020	7:00:00 a, m,	16.1	90	0
7/8/2020	8:00:00 a, m,	16.1	92	0
7/8/2020	9:00:00 a, m,	17.1	93	0
7/8/2020	10:00:00 a, m	17.9	92	0
7/8/2020	11:00:00 a, m	18.9	91	0
7/8/2020	12:00:00 p, m	19.2	88	0
7/8/2020	1:00:00 p, m,	20	88	0
7/8/2020	2:00:00 p, m,	20.7	87	0
7/8/2020	3:00:00 p, m,	20.3	90	0
7/8/2020	4:00:00 p, m,	22	83	0
7/8/2020	5:00:00 p, m,	21.4	85	0
7/8/2020	6:00:00 p, m,	19.8	89	0
7/8/2020	7:00:00 p, m,	18.6	89	0
7/8/2020	8:00:00 p, m,	18.4	84	0
7/8/2020	9:00:00 p, m,	17.9	84	0
7/8/2020	10:00:00 p, m	18.1	84	0
7/8/2020	11:00:00 p, m	18.6	82	0
7/9/2020	12:00:00 a, m	18.1	83	0
7/9/2020	1:00:00 a, m,	17	90	0.2
7/9/2020	2:00:00 a, m,	15.8	96	0.8
7/9/2020	3:00:00 a, m,	15.8	94	0
7/9/2020	4:00:00 a, m,	15.7	94	0
7/9/2020	5:00:00 a, m,	15.7	94	0
7/9/2020	6:00:00 a, m,	16	94	0
7/9/2020	7:00:00 a, m,	15.9	94	0
7/9/2020	8:00:00 a, m,	16.2	95	0
7/9/2020	9:00:00 a, m,	16.4	96	0
7/9/2020	10:00:00 a, m	16.5	97	0.6
7/9/2020	11:00:00 a, m	16.6	98	0.2
7/9/2020	12:00:00 p, m	17.3	98	0
7/9/2020	1:00:00 p, m,	18.3	98	0
7/9/2020	2:00:00 p, m,	19.6	89	0
7/9/2020	3:00:00 p, m,	19.7	89	0
7/9/2020	4:00:00 p, m,	21.1	87	0

7/9/2020	5:00:00 p, m,	20.9	88	0
7/9/2020	6:00:00 p, m,	20.4	87	0
7/9/2020	7:00:00 p, m,	18.3	87	0
7/9/2020	8:00:00 p, m,	17.7	86	0
7/9/2020	9:00:00 p, m,	17.8	85	0
7/9/2020	10:00:00 p, m	18.7	83	0
7/9/2020	11:00:00 p, m	18.1	82	0
7/10/2020	12:00:00 a, m	18.1	83	0
7/10/2020	1:00:00 a, m,	17.8	84	0
7/10/2020	2:00:00 a, m,	17.4	84	0
7/10/2020	3:00:00 a, m,	17.5	87	0
7/10/2020	4:00:00 a, m,	17.3	87	0
7/10/2020	5:00:00 a, m,	17.6	86	0
7/10/2020	6:00:00 a, m,	17.6	84	0
7/10/2020	7:00:00 a, m,	17.7	86	0
7/10/2020	8:00:00 a, m,	17.9	87	0
7/10/2020	9:00:00 a, m,	17.9	89	0
7/10/2020	10:00:00 a, m	18.4	88	0
7/10/2020	11:00:00 a, m	18.7	93	0
7/10/2020	12:00:00 p, m	19.1	88	0
7/10/2020	1:00:00 p, m,	19.5	90	0
7/10/2020	2:00:00 p, m,	20.8	83	0
7/10/2020	3:00:00 p, m,	21.1	83	0
7/10/2020	4:00:00 p, m,	21.2	84	0
7/10/2020	5:00:00 p, m,	20.3	87	0
7/10/2020	6:00:00 p, m,	19.4	87	0
7/10/2020	7:00:00 p, m,	18.2	87	0
7/10/2020	8:00:00 p, m,	17.8	83	0
7/10/2020	9:00:00 p, m,	17.7	82	0
7/10/2020	10:00:00 p, m	18.4	78	0
7/10/2020	11:00:00 p, m	18.2	79	0
7/11/2020	12:00:00 a, m	18	78	0
7/11/2020	1:00:00 a, m,	17.8	78	0
7/11/2020	2:00:00 a, m,	17.3	82	0
7/11/2020	3:00:00 a, m,	17.2	83	0
7/11/2020	4:00:00 a, m,	17.1	84	0
7/11/2020	5:00:00 a, m,	17	84	0
7/11/2020	6:00:00 a, m,	16.9	86	0
7/11/2020	7:00:00 a, m,	17.6	83	0
7/11/2020	8:00:00 a, m,	17.8	85	0
7/11/2020	9:00:00 a, m,	18.3	86	0
7/11/2020	10:00:00 a, m	18.3	88	0
7/11/2020	11:00:00 a, m	19.4	86	0
7/11/2020	12:00:00 p, m	20.2	85	0
7/11/2020	1:00:00 p, m,	20.1	86	0
7/11/2020	2:00:00 p, m,	19.1	95	2
7/11/2020	3:00:00 p, m,	19.2	92	0

7/11/2020	4:00:00 p, m,	17.6	97	4
7/11/2020	5:00:00 p, m,	17.3	95	0
7/11/2020	6:00:00 p, m,	17.6	95	0
7/11/2020	7:00:00 p, m,	16.9	93	0
7/11/2020	8:00:00 p, m,	16.6	93	0
7/11/2020	9:00:00 p, m,	16.7	94	0
7/11/2020	10:00:00 p, m	16.6	93	0
7/11/2020	11:00:00 p, m	16.6	93	0
7/12/2020	12:00:00 a, m	16.7	93	0
7/12/2020	1:00:00 a, m,	16.3	93	0
7/12/2020	2:00:00 a, m,	16	93	0
7/12/2020	3:00:00 a, m,	15.9	93	0
7/12/2020	4:00:00 a, m,	15.9	94	0
7/12/2020	5:00:00 a, m,	16.1	94	0
7/12/2020	6:00:00 a, m,	16.1	94	0
7/12/2020	7:00:00 a, m,	16.1	95	0
7/12/2020	8:00:00 a, m,	16.5	95	0
7/12/2020	9:00:00 a, m,	16.9	97	0
7/12/2020	10:00:00 a, m	17.6	97	0.2
7/12/2020	11:00:00 a, m	18.1	96	0
7/12/2020	12:00:00 p, m	18.1	97	0.6
7/12/2020	1:00:00 p, m,	19.1	96	0
7/12/2020	2:00:00 p, m,	19.3	96	0
7/12/2020	3:00:00 p, m,	20.1	93	0
7/12/2020	4:00:00 p, m,	19.7	93	0
7/12/2020	5:00:00 p, m,	19.9	95	0
7/12/2020	6:00:00 p, m,	19.1	90	0
7/12/2020	7:00:00 p, m,	17.7	92	0
7/12/2020	8:00:00 p, m,	17.8	93	0
7/12/2020	9:00:00 p, m,	17.3	92	0
7/12/2020	10:00:00 p, m	17.3	94	0
7/12/2020	11:00:00 p, m	17.1	94	0
7/13/2020	12:00:00 a, m	17.2	95	0
7/13/2020	1:00:00 a, m,	17.1	95	0
7/13/2020	2:00:00 a, m,	17.1	94	0
7/13/2020	3:00:00 a, m,	16.8	95	0.2
7/13/2020	4:00:00 a, m,	16.4	95	0
7/13/2020	5:00:00 a, m,	16.2	96	0
7/13/2020	6:00:00 a, m,	15.9	94	0
7/13/2020	7:00:00 a, m,	15.8	95	0
7/13/2020	8:00:00 a, m,	16.2	96	0
7/13/2020	9:00:00 a, m,	16.8	97	0
7/13/2020	10:00:00 a, m	17.7	96	0
7/13/2020	11:00:00 a, m	18.6	97	0
7/13/2020	12:00:00 p, m	19.4	95	0
7/13/2020	1:00:00 p, m,	20.1	96	0
7/13/2020	2:00:00 p, m,	20.2	92	0

7/13/2020	3:00:00 p, m,	20.7	90	0
7/13/2020	4:00:00 p, m,	21.1	90	0
7/13/2020	5:00:00 p, m,	21	89	0
7/13/2020	6:00:00 p, m,	19.1	84	0
7/13/2020	7:00:00 p, m,	18.9	82	0
7/13/2020	8:00:00 p, m,	18.6	82	0
7/13/2020	9:00:00 p, m,	18.9	80	0
7/13/2020	10:00:00 p, m	18.9	79	0
7/13/2020	11:00:00 p, m	18.4	83	0
7/14/2020	12:00:00 a, m	18.7	81	0
7/14/2020	1:00:00 a, m,	17.9	84	0
7/14/2020	2:00:00 a, m,	17.7	81	0
7/14/2020	3:00:00 a, m,	17.7	79	0
7/14/2020	4:00:00 a, m,	18.1	77	0
7/14/2020	5:00:00 a, m,	17.1	81	0
7/14/2020	6:00:00 a, m,	16.6	85	0
7/14/2020	7:00:00 a, m,	17.4	84	0
7/14/2020	8:00:00 a, m,	17.8	83	0
7/14/2020	9:00:00 a, m,	18.3	83	0
7/14/2020	10:00:00 a, m	18.9	85	0
7/14/2020	11:00:00 a, m	20.3	80	0
7/14/2020	12:00:00 p, m	22.2	69	0
7/14/2020	1:00:00 p, m,	21.6	78	0
7/14/2020	2:00:00 p, m,	20.8	77	0
7/14/2020	3:00:00 p, m,	22.6	74	0
7/14/2020	4:00:00 p, m,	21.9	76	0
7/14/2020	5:00:00 p, m,	22.4	74	0
7/14/2020	6:00:00 p, m,	21.3	69	0
7/14/2020	7:00:00 p, m,	19.6	72	0
7/14/2020	8:00:00 p, m,	18.8	76	0
7/14/2020	9:00:00 p, m,	18.8	74	0
7/14/2020	10:00:00 p, m	19.2	70	0
7/14/2020	11:00:00 p, m	18.3	78	0
7/15/2020	12:00:00 a, m	16.8	88	0.2
7/15/2020	1:00:00 a, m,	15.7	92	0
7/15/2020	2:00:00 a, m,	16.1	89	0
7/15/2020	3:00:00 a, m,	15.5	89	0
7/15/2020	4:00:00 a, m,	15.7	87	0
7/15/2020	5:00:00 a, m,	15.3	87	0
7/15/2020	6:00:00 a, m,	15.9	86	0
7/15/2020	7:00:00 a, m,	16.4	85	0
7/15/2020	8:00:00 a, m,	16.7	85	0
7/15/2020	9:00:00 a, m,	17.3	89	0
7/15/2020	10:00:00 a, m	18.2	89	0
7/15/2020	11:00:00 a, m	18.6	85	0
7/15/2020	12:00:00 p, m	19	89	0
7/15/2020	1:00:00 p, m,	19.6	84	0

7/15/2020	2:00:00 p, m,	20.1	80	0.2
7/15/2020	3:00:00 p, m,	18.9	81	0
7/15/2020	4:00:00 p, m,	20.1	78	0
7/15/2020	5:00:00 p, m,	20.7	78	0
7/15/2020	6:00:00 p, m,	19.8	77	0
7/15/2020	7:00:00 p, m,	18.1	77	0
7/15/2020	8:00:00 p, m,	18.2	76	0
7/15/2020	9:00:00 p, m,	17.7	77	0
7/15/2020	10:00:00 p, m	17.7	74	0
7/15/2020	11:00:00 p, m	18.5	72	0
7/16/2020	12:00:00 a, m	18.1	76	0
7/16/2020	1:00:00 a, m,	17.7	77	0
7/16/2020	2:00:00 a, m,	17.1	81	0
7/16/2020	3:00:00 a, m,	17.1	81	0
7/16/2020	4:00:00 a, m,	17.1	80	0
7/16/2020	5:00:00 a, m,	17.1	80	0
7/16/2020	6:00:00 a, m,	16.7	81	0
7/16/2020	7:00:00 a, m,	16.7	85	0
7/16/2020	8:00:00 a, m,	17.5	83	0
7/16/2020	9:00:00 a, m,	17.8	84	0
7/16/2020	10:00:00 a, m	17.9	84	0
7/16/2020	11:00:00 a, m	18.5	87	0
7/16/2020	12:00:00 p, m	19.6	83	0
7/16/2020	1:00:00 p, m,	20.1	82	0
7/16/2020	2:00:00 p, m,	20.4	82	0
7/16/2020	3:00:00 p, m,	20.2	82	0
7/16/2020	4:00:00 p, m,	19.9	83	0
7/16/2020	5:00:00 p, m,	22	75	0
7/16/2020	6:00:00 p, m,	21.3	77	0
7/16/2020	7:00:00 p, m,	18.5	80	0
7/16/2020	8:00:00 p, m,	18.4	79	0
7/16/2020	9:00:00 p, m,	18.7	77	0
7/16/2020	10:00:00 p, m	18.1	77	0
7/16/2020	11:00:00 p, m	17.8	78	0
7/17/2020	12:00:00 a, m	18.2	77	0
7/17/2020	1:00:00 a, m,	18.2	77	0
7/17/2020	2:00:00 a, m,	17.1	83	0
7/17/2020	3:00:00 a, m,	17.2	82	0
7/17/2020	4:00:00 a, m,	16.9	84	0
7/17/2020	5:00:00 a, m,	16.8	83	0
7/17/2020	6:00:00 a, m,	17.1	80	0
7/17/2020	7:00:00 a, m,	16.9	79	0
7/17/2020	8:00:00 a, m,	17.4	84	0
7/17/2020	9:00:00 a, m,	17.9	81	0
7/17/2020	10:00:00 a, m	18.6	80	0
7/17/2020	11:00:00 a, m	19.1	80	0
7/17/2020	12:00:00 p, m	19.6	81	0

7/17/2020	1:00:00 p, m,	19.5	81	0
7/17/2020	2:00:00 p, m,	18.9	85	0
7/17/2020	3:00:00 p, m,	18	91	0.2
7/17/2020	4:00:00 p, m,	18.7	89	0
7/17/2020	5:00:00 p, m,	19	88	0
7/17/2020	6:00:00 p, m,	18.3	84	0
7/17/2020	7:00:00 p, m,	17.6	81	0
7/17/2020	8:00:00 p, m,	17.6	81	0
7/17/2020	9:00:00 p, m,	18.1	74	0
7/17/2020	10:00:00 p, m	17.9	75	0
7/17/2020	11:00:00 p, m	18.1	75	0
7/18/2020	12:00:00 a, m	18.4	76	0
7/18/2020	1:00:00 a, m,	17.9	76	0
7/18/2020	2:00:00 a, m,	17.7	78	0
7/18/2020	3:00:00 a, m,	17.6	78	0
7/18/2020	4:00:00 a, m,	16.9	78	0
7/18/2020	5:00:00 a, m,	16.6	80	0
7/18/2020	6:00:00 a, m,	17.6	78	0
7/18/2020	7:00:00 a, m,	17.7	77	0
7/18/2020	8:00:00 a, m,	17.9	80	0
7/18/2020	9:00:00 a, m,	18.6	75	0
7/18/2020	10:00:00 a, m	18.4	81	0
7/18/2020	11:00:00 a, m	19.3	80	0
7/18/2020	12:00:00 p, m	20.7	73	0
7/18/2020	1:00:00 p, m,	19.3	85	0.4
7/18/2020	2:00:00 p, m,	19.5	86	0
7/18/2020	3:00:00 p, m,	19.8	82	0
7/18/2020	4:00:00 p, m,	21.3	77	0
7/18/2020	5:00:00 p, m,	20.2	81	0
7/18/2020	6:00:00 p, m,	20.7	78	0
7/18/2020	7:00:00 p, m,	18.7	79	0
7/18/2020	8:00:00 p, m,	17.7	82	0
7/18/2020	9:00:00 p, m,	17.8	82	0
7/18/2020	10:00:00 p, m	17.6	83	0
7/18/2020	11:00:00 p, m	17.8	80	0
7/19/2020	12:00:00 a, m	17.7	81	0
7/19/2020	1:00:00 a, m,	17.7	82	0
7/19/2020	2:00:00 a, m,	17.3	82	0
7/19/2020	3:00:00 a, m,	17.3	83	0
7/19/2020	4:00:00 a, m,	17.3	82	0
7/19/2020	5:00:00 a, m,	17.1	83	0
7/19/2020	6:00:00 a, m,	17.3	81	0
7/19/2020	7:00:00 a, m,	17.4	84	0
7/19/2020	8:00:00 a, m,	18.4	83	0
7/19/2020	9:00:00 a, m,	18.7	81	0
7/19/2020	10:00:00 a, m	19.5	78	0
7/19/2020	11:00:00 a, m	21.5	77	0

7/19/2020	12:00:00 p, m	20.6	77	0
7/19/2020	1:00:00 p, m,	20.4	80	0
7/19/2020	2:00:00 p, m,	20.1	82	0.2
7/19/2020	3:00:00 p, m,	18.9	93	0.2
7/19/2020	4:00:00 p, m,	18.9	87	0
7/19/2020	5:00:00 p, m,	20.5	81	0
7/19/2020	6:00:00 p, m,	19.6	80	0
7/19/2020	7:00:00 p, m,	17.7	89	0
7/19/2020	8:00:00 p, m,	17.1	90	0.2
7/19/2020	9:00:00 p, m,	17.4	87	0
7/19/2020	10:00:00 p, m	17.7	87	0
7/19/2020	11:00:00 p, m	17.7	85	0
7/20/2020	12:00:00 a, m	17.2	85	0
7/20/2020	1:00:00 a, m,	17.1	85	0
7/20/2020	2:00:00 a, m,	17.6	84	0
7/20/2020	3:00:00 a, m,	17.5	81	0
7/20/2020	4:00:00 a, m,	17.9	79	0
7/20/2020	5:00:00 a, m,	17.7	79	0
7/20/2020	6:00:00 a, m,	17.7	80	0
7/20/2020	7:00:00 a, m,	18.1	80	0
7/20/2020	8:00:00 a, m,	18.3	84	0
7/20/2020	9:00:00 a, m,	18.6	81	0
7/20/2020	10:00:00 a, m	18.1	89	1
7/20/2020	11:00:00 a, m	17.9	96	0.6
7/20/2020	12:00:00 p, m	17.6	96	0.2
7/20/2020	1:00:00 p, m,	18.5	98	0.2
7/20/2020	2:00:00 p, m,	19.9	87	0
7/20/2020	3:00:00 p, m,	19.5	88	0
7/20/2020	4:00:00 p, m,	19.4	90	0
7/20/2020	5:00:00 p, m,	19.7	90	0
7/20/2020	6:00:00 p, m,	18.7	92	0
7/20/2020	7:00:00 p, m,	18	82	0.2
7/20/2020	8:00:00 p, m,	17.9	83	0
7/20/2020	9:00:00 p, m,	17.9	82	0
7/20/2020	10:00:00 p, m	17.9	82	0
7/20/2020	11:00:00 p, m	18.2	79	0
7/21/2020	12:00:00 a, m	18.1	79	0
7/21/2020	1:00:00 a, m,	17.7	81	0
7/21/2020	2:00:00 a, m,	17.7	83	0
7/21/2020	3:00:00 a, m,	17.3	86	0
7/21/2020	4:00:00 a, m,	17.2	85	0
7/21/2020	5:00:00 a, m,	17	84	0
7/21/2020	6:00:00 a, m,	17.1	85	0
7/21/2020	7:00:00 a, m,	17.3	85	0
7/21/2020	8:00:00 a, m,	17.7	87	0
7/21/2020	9:00:00 a, m,	18.3	87	0
7/21/2020	10:00:00 a, m	19	86	0

7/21/2020	11:00:00 a, m	19.5	86	0
7/21/2020	12:00:00 p, m	20.2	85	0
7/21/2020	1:00:00 p, m,	21.8	73	0
7/21/2020	2:00:00 p, m,	21.6	78	0
7/21/2020	3:00:00 p, m,	21.6	82	0
7/21/2020	4:00:00 p, m,	21.6	81	0
7/21/2020	5:00:00 p, m,	19.9	85	0
7/21/2020	6:00:00 p, m,	19	84	0
7/21/2020	7:00:00 p, m,	18.8	82	0
7/21/2020	8:00:00 p, m,	18.8	81	0
7/21/2020	9:00:00 p, m,	19.2	78	0
7/21/2020	10:00:00 p, m	19.1	79	0
7/21/2020	11:00:00 p, m	18.7	82	0
7/22/2020	12:00:00 a, m	18.8	81	0
7/22/2020	1:00:00 a, m,	18.2	83	0
7/22/2020	2:00:00 a, m,	18	83	0
7/22/2020	3:00:00 a, m,	17.3	85	0
7/22/2020	4:00:00 a, m,	17.3	84	0
7/22/2020	5:00:00 a, m,	17.1	85	0
7/22/2020	6:00:00 a, m,	17.1	86	0
7/22/2020	7:00:00 a, m,	17.1	88	0
7/22/2020	8:00:00 a, m,	17.5	87	0
7/22/2020	9:00:00 a, m,	17.8	88	0
7/22/2020	10:00:00 a, m	18.6	89	0
7/22/2020	11:00:00 a, m	19.1	88	0
7/22/2020	12:00:00 p, m	20.8	85	0
7/22/2020	1:00:00 p, m,	21.1	83	0
7/22/2020	2:00:00 p, m,	21.8	81	0
7/22/2020	3:00:00 p, m,	22.3	79	0
7/22/2020	4:00:00 p, m,	22.1	78	0
7/22/2020	5:00:00 p, m,	22.3	78	0
7/22/2020	6:00:00 p, m,	20.8	82	0
7/22/2020	7:00:00 p, m,	19.7	84	0
7/22/2020	8:00:00 p, m,	19.3	84	0
7/22/2020	9:00:00 p, m,	19.2	86	0
7/22/2020	10:00:00 p, m	19	86	0
7/22/2020	11:00:00 p, m	18.8	87	0
7/23/2020	12:00:00 a, m	18.3	92	0
7/23/2020	1:00:00 a, m,	17.6	94	0.2
7/23/2020	2:00:00 a, m,	17.2	96	0.2
7/23/2020	3:00:00 a, m,	16.9	95	0
7/23/2020	4:00:00 a, m,	16.5	95	0
7/23/2020	5:00:00 a, m,	16.3	94	0
7/23/2020	6:00:00 a, m,	16.5	94	0
7/23/2020	7:00:00 a, m,	16.7	95	0
7/23/2020	8:00:00 a, m,	17.3	95	0
7/23/2020	9:00:00 a, m,	17.9	91	0

7/23/2020	10:00:00 a, m	18.4	93	0
7/23/2020	11:00:00 a, m	19	90	0
7/23/2020	12:00:00 p, m	20	85	0
7/23/2020	1:00:00 p, m,	20.4	89	0
7/23/2020	2:00:00 p, m,	20.4	86	0
7/23/2020	3:00:00 p, m,	21.1	83	0
7/23/2020	4:00:00 p, m,	20.7	82	0
7/23/2020	5:00:00 p, m,	18.8	90	0.6
7/23/2020	6:00:00 p, m,	17.8	94	0
7/23/2020	7:00:00 p, m,	17.5	95	0.2
7/23/2020	8:00:00 p, m,	17.3	97	0
7/23/2020	9:00:00 p, m,	17.2	95	0
7/23/2020	10:00:00 p, m	17.1	95	0
7/23/2020	11:00:00 p, m	17	95	0
7/24/2020	12:00:00 a, m	16.9	95	0
7/24/2020	1:00:00 a, m,	16.9	94	0
7/24/2020	2:00:00 a, m,	16.9	94	0
7/24/2020	3:00:00 a, m,	16.8	94	0
7/24/2020	4:00:00 a, m,	16.8	95	0
7/24/2020	5:00:00 a, m,	16.8	94	0
7/24/2020	6:00:00 a, m,	16.8	94	0
7/24/2020	7:00:00 a, m,	17.1	94	0
7/24/2020	8:00:00 a, m,	17.4	93	0
7/24/2020	9:00:00 a, m,	17.7	95	0
7/24/2020	10:00:00 a, m	18.3	96	0
7/24/2020	11:00:00 a, m	18.3	93	0.2
7/24/2020	12:00:00 p, m	18.7	97	0
7/24/2020	1:00:00 p, m,	18.8	96	0
7/24/2020	2:00:00 p, m,	19.4	89	0
7/24/2020	3:00:00 p, m,	17.9	96	3.6
7/24/2020	4:00:00 p, m,	18.1	95	0
7/24/2020	5:00:00 p, m,	18.6	97	0
7/24/2020	6:00:00 p, m,	17.9	96	0
7/24/2020	7:00:00 p, m,	17.1	97	0
7/24/2020	8:00:00 p, m,	16.9	96	0
7/24/2020	9:00:00 p, m,	16.8	96	0
7/24/2020	10:00:00 p, m	16.9	95	0
7/24/2020	11:00:00 p, m	16.8	93	0
7/25/2020	12:00:00 a, m	17	95	0
7/25/2020	1:00:00 a, m,	17	96	1
7/25/2020	2:00:00 a, m,	15.9	97	4.2
7/25/2020	3:00:00 a, m,	15.6	97	2.6
7/25/2020	4:00:00 a, m,	15.4	98	0.2
7/25/2020	5:00:00 a, m,	15.5	98	0
7/25/2020	6:00:00 a, m,	15.4	98	0
7/25/2020	7:00:00 a, m,	15.7	98	0
7/25/2020	8:00:00 a, m,	16	98	0

7/25/2020	9:00:00 a, m,	16.2	98	0.6
7/25/2020	10:00:00 a, m	16.1	98	1
7/25/2020	11:00:00 a, m	15.6	97	1
7/25/2020	12:00:00 p, m	15.4	98	0.6
7/25/2020	1:00:00 p, m,	15.4	98	0.2
7/25/2020	2:00:00 p, m,	16	99	0.4
7/25/2020	3:00:00 p, m,	16.5	99	0
7/25/2020	4:00:00 p, m,	16.9	99	0
7/25/2020	5:00:00 p, m,	17	97	0.2
7/25/2020	6:00:00 p, m,	16.4	97	0
7/25/2020	7:00:00 p, m,	15.4	97	0
7/25/2020	8:00:00 p, m,	15	96	0
7/25/2020	9:00:00 p, m,	15.2	96	0
7/25/2020	10:00:00 p, m	15.3	95	0
7/25/2020	11:00:00 p, m	15.1	94	0
7/26/2020	12:00:00 a, m	15.1	93	0
7/26/2020	1:00:00 a, m,	15.3	93	0
7/26/2020	2:00:00 a, m,	15.9	87	0
7/26/2020	3:00:00 a, m,	16.7	84	0
7/26/2020	4:00:00 a, m,	16.7	84	0
7/26/2020	5:00:00 a, m,	15.9	88	0
7/26/2020	6:00:00 a, m,	16.1	87	0
7/26/2020	7:00:00 a, m,	16.6	86	0
7/26/2020	8:00:00 a, m,	16.6	91	0
7/26/2020	9:00:00 a, m,	17	91	0
7/26/2020	10:00:00 a, m	17.3	91	0
7/26/2020	11:00:00 a, m	17.6	92	0
7/26/2020	12:00:00 p, m	17.8	92	0
7/26/2020	1:00:00 p, m,	17.9	92	0
7/26/2020	2:00:00 p, m,	18.4	91	0
7/26/2020	3:00:00 p, m,	18.8	92	0
7/26/2020	4:00:00 p, m,	19.6	90	0
7/26/2020	5:00:00 p, m,	17.7	91	0
7/26/2020	6:00:00 p, m,	17.6	90	0
7/26/2020	7:00:00 p, m,	17.6	89	0
7/26/2020	8:00:00 p, m,	17.2	88	0.2
7/26/2020	9:00:00 p, m,	17.3	86	0
7/26/2020	10:00:00 p, m	17	86	0
7/26/2020	11:00:00 p, m	17.1	86	0
7/27/2020	12:00:00 a, m	17.1	87	0
7/27/2020	1:00:00 a, m,	16.9	84	0
7/27/2020	2:00:00 a, m,	16.7	85	0
7/27/2020	3:00:00 a, m,	16.3	88	0
7/27/2020	4:00:00 a, m,	16.3	86	0
7/27/2020	5:00:00 a, m,	16.2	86	0
7/27/2020	6:00:00 a, m,	15.6	86	0
7/27/2020	7:00:00 a, m,	16.3	87	0

7/27/2020	8:00:00 a, m,	16.7	88	0
7/27/2020	9:00:00 a, m,	17.3	87	0
7/27/2020	10:00:00 a, m	18.1	87	0
7/27/2020	11:00:00 a, m	17.9	90	0
7/27/2020	12:00:00 p, m	18.3	88	0
7/27/2020	1:00:00 p, m,	19	86	0
7/27/2020	2:00:00 p, m,	20.8	84	0
7/27/2020	3:00:00 p, m,	20.2	85	0
7/27/2020	4:00:00 p, m,	20.2	87	0
7/27/2020	5:00:00 p, m,	20.1	89	0
7/27/2020	6:00:00 p, m,	20.3	86	0
7/27/2020	7:00:00 p, m,	18.5	81	0
7/27/2020	8:00:00 p, m,	18.3	81	0
7/27/2020	9:00:00 p, m,	17.8	82	0
7/27/2020	10:00:00 p, m	18.1	80	0
7/27/2020	11:00:00 p, m	18.1	81	0
7/28/2020	12:00:00 a, m	18	83	0
7/28/2020	1:00:00 a, m,	17.8	84	0
7/28/2020	2:00:00 a, m,	17.6	84	0
7/28/2020	3:00:00 a, m,	17.6	84	0
7/28/2020	4:00:00 a, m,	17.3	85	0
7/28/2020	5:00:00 a, m,	17.2	84	0
7/28/2020	6:00:00 a, m,	17.2	85	0
7/28/2020	7:00:00 a, m,	17.3	86	0
7/28/2020	8:00:00 a, m,	17.8	87	0
7/28/2020	9:00:00 a, m,	18.2	87	0
7/28/2020	10:00:00 a, m	18.7	87	0
7/28/2020	11:00:00 a, m	19	86	0
7/28/2020	12:00:00 p, m	19.2	86	0.2
7/28/2020	1:00:00 p, m,	20.1	86	0
7/28/2020	2:00:00 p, m,	20.7	87	0
7/28/2020	3:00:00 p, m,	20.5	90	0
7/28/2020	4:00:00 p, m,	21.5	83	0
7/28/2020	5:00:00 p, m,	21.3	82	0
7/28/2020	6:00:00 p, m,	20	81	0
7/28/2020	7:00:00 p, m,	18.8	84	0
7/28/2020	8:00:00 p, m,	18.9	80	0
7/28/2020	9:00:00 p, m,	18.8	80	0
7/28/2020	10:00:00 p, m	18.8	84	0
7/28/2020	11:00:00 p, m	17.9	86	0
7/29/2020	12:00:00 a, m	18.2	84	0
7/29/2020	1:00:00 a, m,	18.1	83	0
7/29/2020	2:00:00 a, m,	17.8	84	0
7/29/2020	3:00:00 a, m,	16.9	92	0.2
7/29/2020	4:00:00 a, m,	16.1	95	0.4
7/29/2020	5:00:00 a, m,	15.9	95	0
7/29/2020	6:00:00 a, m,	15.8	95	0

7/29/2020	7:00:00 a, m,	15.8	97	0.6
7/29/2020	8:00:00 a, m,	16.3	96	0
7/29/2020	9:00:00 a, m,	16.7	97	0.4
7/29/2020	10:00:00 a, m	17.4	98	0
7/29/2020	11:00:00 a, m	18.1	96	0.2
7/29/2020	12:00:00 p, m	18.4	97	0
7/29/2020	1:00:00 p, m,	19.2	97	0
7/29/2020	2:00:00 p, m,	19.8	92	0
7/29/2020	3:00:00 p, m,	19.9	91	0
7/29/2020	4:00:00 p, m,	20.1	89	0
7/29/2020	5:00:00 p, m,	19.9	91	0
7/29/2020	6:00:00 p, m,	20.3	90	0
7/29/2020	7:00:00 p, m,	18.8	92	0
7/29/2020	8:00:00 p, m,	18.4	92	0
7/29/2020	9:00:00 p, m,	18.7	86	0
7/29/2020	10:00:00 p, m	19	84	0
7/29/2020	11:00:00 p, m	18.6	85	0
7/30/2020	12:00:00 a, m	18.3	85	0
7/30/2020	1:00:00 a, m,	18.2	83	0
7/30/2020	2:00:00 a, m,	17.9	86	0
7/30/2020	3:00:00 a, m,	18.5	82	0
7/30/2020	4:00:00 a, m,	17.8	87	0
7/30/2020	5:00:00 a, m,	17.5	89	0
7/30/2020	6:00:00 a, m,	16.9	92	0
7/30/2020	7:00:00 a, m,	16.2	94	0
7/30/2020	8:00:00 a, m,	17.1	93	0
7/30/2020	9:00:00 a, m,	17.7	92	0
7/30/2020	10:00:00 a, m	17.9	89	0
7/30/2020	11:00:00 a, m	18.4	93	0.2
7/30/2020	12:00:00 p, m	19.8	89	0
7/30/2020	1:00:00 p, m,	20.3	87	0
7/30/2020	2:00:00 p, m,	20.2	89	0
7/30/2020	3:00:00 p, m,	20.4	87	0
7/30/2020	4:00:00 p, m,	20.6	85	0
7/30/2020	5:00:00 p, m,	21	84	0
7/30/2020	6:00:00 p, m,	20.2	85	0.2
7/30/2020	7:00:00 p, m,	18.3	90	0
7/30/2020	8:00:00 p, m,	17.4	90	0
7/30/2020	9:00:00 p, m,	17.6	89	0
7/30/2020	10:00:00 p, m	17.9	86	0
7/30/2020	11:00:00 p, m	17.9	84	0
7/31/2020	12:00:00 a, m	17.8	84	0
7/31/2020	1:00:00 a, m,	17.4	79	0
7/31/2020	2:00:00 a, m,	17.3	80	0
7/31/2020	3:00:00 a, m,	17.3	81	0
7/31/2020	4:00:00 a, m,	17.3	80	0
7/31/2020	5:00:00 a, m,	17	79	0

7/31/2020	6:00:00 a, m,	17.1	81	0
7/31/2020	7:00:00 a, m,	16.8	84	0
7/31/2020	8:00:00 a, m,	17.1	85	0
7/31/2020	9:00:00 a, m,	17.7	83	0
7/31/2020	10:00:00 a, m	18.3	87	0
7/31/2020	11:00:00 a, m	19.1	77	0
7/31/2020	12:00:00 p, m	20.6	74	0
7/31/2020	1:00:00 p, m,	20.9	71	0
7/31/2020	2:00:00 p, m,	21.2	69	0
7/31/2020	3:00:00 p, m,	22	65	0
7/31/2020	4:00:00 p, m,	22.3	61	0
7/31/2020	5:00:00 p, m,	20.7	69	0
7/31/2020	6:00:00 p, m,	20.1	69	0
7/31/2020	7:00:00 p, m,	18.9	69	0
7/31/2020	8:00:00 p, m,	18.5	68	0
7/31/2020	9:00:00 p, m,	17.5	68	0
7/31/2020	10:00:00 p, m	18.3	57	0
7/31/2020	11:00:00 p, m	17.7	59	0
Jul_20		18.0723118	87.9569892	58.4
8/1/2020	12:00:00 a, m	16.6	64	0
8/1/2020	1:00:00 a, m,	16.6	68	0
8/1/2020	2:00:00 a, m,	17.8	65	0
8/1/2020	3:00:00 a, m,	17.7	68	0
8/1/2020	4:00:00 a, m,	17.6	72	0
8/1/2020	5:00:00 a, m,	17.3	73	0
8/1/2020	6:00:00 a, m,	17.2	73	0
8/1/2020	7:00:00 a, m,	17.4	72	0
8/1/2020	8:00:00 a, m,	17.2	76	0
8/1/2020	9:00:00 a, m,	17.6	75	0
8/1/2020	10:00:00 a, m	19.2	71	0
8/1/2020	11:00:00 a, m	20.5	72	0
8/1/2020	12:00:00 p, m	21.8	70	0
8/1/2020	1:00:00 p, m,	22.1	66	0
8/1/2020	2:00:00 p, m,	21.7	69	0
8/1/2020	3:00:00 p, m,	21.4	74	0
8/1/2020	4:00:00 p, m,	20.4	77	0
8/1/2020	5:00:00 p, m,	21.1	66	0
8/1/2020	6:00:00 p, m,	21.2	65	0
8/1/2020	7:00:00 p, m,	19.5	68	0
8/1/2020	8:00:00 p, m,	18.9	67	0
8/1/2020	9:00:00 p, m,	18.9	65	0
8/1/2020	10:00:00 p, m	19.1	59	0
8/1/2020	11:00:00 p, m	18.4	59	0
8/2/2020	12:00:00 a, m	18.6	54	0
8/2/2020	1:00:00 a, m,	18.2	55	0
8/2/2020	2:00:00 a, m,	18.2	59	0
8/2/2020	3:00:00 a, m,	18.4	59	0

8/2/2020	4:00:00 a, m,	18.2	64	0
8/2/2020	5:00:00 a, m,	17.4	70	0
8/2/2020	6:00:00 a, m,	17.6	68	0
8/2/2020	7:00:00 a, m,	16.8	75	0
8/2/2020	8:00:00 a, m,	17	76	0
8/2/2020	9:00:00 a, m,	17.8	78	0
8/2/2020	10:00:00 a, m	19	74	0
8/2/2020	11:00:00 a, m	19.9	72	0
8/2/2020	12:00:00 p, m	20.4	72	0
8/2/2020	1:00:00 p, m,	20.4	73	0
8/2/2020	2:00:00 p, m,	21.1	73	0
8/2/2020	3:00:00 p, m,	21.4	71	0
8/2/2020	4:00:00 p, m,	21.6	61	0
8/2/2020	5:00:00 p, m,	20.7	64	0
8/2/2020	6:00:00 p, m,	20.9	64	0
8/2/2020	7:00:00 p, m,	19.4	65	0
8/2/2020	8:00:00 p, m,	18.9	62	0
8/2/2020	9:00:00 p, m,	18.6	64	0
8/2/2020	10:00:00 p, m	17.8	68	0
8/2/2020	11:00:00 p, m	18.3	67	0
8/3/2020	12:00:00 a, m	17.8	68	0
8/3/2020	1:00:00 a, m,	17.4	72	0
8/3/2020	2:00:00 a, m,	17.3	72	0
8/3/2020	3:00:00 a, m,	17.5	70	0
8/3/2020	4:00:00 a, m,	17.4	71	0
8/3/2020	5:00:00 a, m,	17.3	70	0
8/3/2020	6:00:00 a, m,	17.7	71	0
8/3/2020	7:00:00 a, m,	17.7	71	0
8/3/2020	8:00:00 a, m,	18.2	71	0
8/3/2020	9:00:00 a, m,	18.6	75	0
8/3/2020	10:00:00 a, m	18.9	75	0
8/3/2020	11:00:00 a, m	19.3	79	0
8/3/2020	12:00:00 p, m	19.4	77	0
8/3/2020	1:00:00 p, m,	18.9	81	0
8/3/2020	2:00:00 p, m,	19.4	80	0
8/3/2020	3:00:00 p, m,	19.4	80	0
8/3/2020	4:00:00 p, m,	18.6	82	0
8/3/2020	5:00:00 p, m,	18.3	84	0
8/3/2020	6:00:00 p, m,	18.1	82	0.2
8/3/2020	7:00:00 p, m,	17.7	75	0
8/3/2020	8:00:00 p, m,	18.2	71	0
8/3/2020	9:00:00 p, m,	18.2	69	0
8/3/2020	10:00:00 p, m	18.5	71	0
8/3/2020	11:00:00 p, m	18.1	74	0
8/4/2020	12:00:00 a, m	17.8	75	0
8/4/2020	1:00:00 a, m,	17.5	77	0
8/4/2020	2:00:00 a, m,	17.2	75	0

8/4/2020	3:00:00 a, m,	17.1	77	0
8/4/2020	4:00:00 a, m,	17.2	78	0
8/4/2020	5:00:00 a, m,	16.6	80	0
8/4/2020	6:00:00 a, m,	16.8	77	0
8/4/2020	7:00:00 a, m,	16.8	79	0
8/4/2020	8:00:00 a, m,	17.4	80	0
8/4/2020	9:00:00 a, m,	17.9	81	0
8/4/2020	10:00:00 a, m	17.9	81	0
8/4/2020	11:00:00 a, m	18.4	79	0
8/4/2020	12:00:00 p, m	19	72	0
8/4/2020	1:00:00 p, m,	18.7	76	0
8/4/2020	2:00:00 p, m,	19.7	74	0
8/4/2020	3:00:00 p, m,	18.9	79	0
8/4/2020	4:00:00 p, m,	18.8	80	0
8/4/2020	5:00:00 p, m,	20.4	73	0
8/4/2020	6:00:00 p, m,	19.1	78	0
8/4/2020	7:00:00 p, m,	16.9	78	0
8/4/2020	8:00:00 p, m,	16.4	73	0
8/4/2020	9:00:00 p, m,	16.8	74	0
8/4/2020	10:00:00 p, m	17	74	0
8/4/2020	11:00:00 p, m	17.3	69	0
8/5/2020	12:00:00 a, m	17.2	69	0
8/5/2020	1:00:00 a, m,	17.8	67	0
8/5/2020	2:00:00 a, m,	17.9	65	0
8/5/2020	3:00:00 a, m,	17.9	64	0
8/5/2020	4:00:00 a, m,	17.7	66	0
8/5/2020	5:00:00 a, m,	17.4	70	0
8/5/2020	6:00:00 a, m,	17.7	68	0
8/5/2020	7:00:00 a, m,	17.2	76	0
8/5/2020	8:00:00 a, m,	17.4	76	0
8/5/2020	9:00:00 a, m,	17.9	76	0
8/5/2020	10:00:00 a, m	18.4	75	0
8/5/2020	11:00:00 a, m	18.6	80	0
8/5/2020	12:00:00 p, m	19.6	76	0
8/5/2020	1:00:00 p, m,	18.8	79	0
8/5/2020	2:00:00 p, m,	19.6	79	0
8/5/2020	3:00:00 p, m,	19.4	73	0
8/5/2020	4:00:00 p, m,	19.3	72	0
8/5/2020	5:00:00 p, m,	19.6	70	0
8/5/2020	6:00:00 p, m,	19.6	76	0
8/5/2020	7:00:00 p, m,	18.1	76	0
8/5/2020	8:00:00 p, m,	17.7	77	0
8/5/2020	9:00:00 p, m,	18.2	74	0
8/5/2020	10:00:00 p, m	17.6	77	0
8/5/2020	11:00:00 p, m	16.3	82	0
8/6/2020	12:00:00 a, m	16.8	82	0
8/6/2020	1:00:00 a, m,	16.3	82	0

8/6/2020	2:00:00 a, m,	16.6	81	0
8/6/2020	3:00:00 a, m,	16.8	81	0
8/6/2020	4:00:00 a, m,	16.8	82	0
8/6/2020	5:00:00 a, m,	16.6	82	0
8/6/2020	6:00:00 a, m,	16.8	81	0
8/6/2020	7:00:00 a, m,	17.6	79	0
8/6/2020	8:00:00 a, m,	18.2	78	0
8/6/2020	9:00:00 a, m,	19.2	75	0
8/6/2020	10:00:00 a, m	20.1	75	0
8/6/2020	11:00:00 a, m	20.4	70	0
8/6/2020	12:00:00 p, m	20.6	70	0
8/6/2020	1:00:00 p, m,	21.2	69	0
8/6/2020	2:00:00 p, m,	20.7	71	0
8/6/2020	3:00:00 p, m,	21.1	71	0
8/6/2020	4:00:00 p, m,	20.8	70	0
8/6/2020	5:00:00 p, m,	19.9	73	0
8/6/2020	6:00:00 p, m,	19.4	75	0
8/6/2020	7:00:00 p, m,	18.6	74	0
8/6/2020	8:00:00 p, m,	18.3	77	0
8/6/2020	9:00:00 p, m,	18.6	74	0
8/6/2020	10:00:00 p, m	18.4	75	0
8/6/2020	11:00:00 p, m	18.7	73	0
8/7/2020	12:00:00 a, m	18.7	72	0
8/7/2020	1:00:00 a, m,	18.6	71	0
8/7/2020	2:00:00 a, m,	18.3	73	0
8/7/2020	3:00:00 a, m,	17.9	75	0
8/7/2020	4:00:00 a, m,	16.6	84	0
8/7/2020	5:00:00 a, m,	16.7	83	0
8/7/2020	6:00:00 a, m,	16.6	84	0
8/7/2020	7:00:00 a, m,	16.8	85	0
8/7/2020	8:00:00 a, m,	17.3	84	0
8/7/2020	9:00:00 a, m,	17.9	81	0
8/7/2020	10:00:00 a, m	18.2	84	0
8/7/2020	11:00:00 a, m	18.6	82	0
8/7/2020	12:00:00 p, m	19	83	0
8/7/2020	1:00:00 p, m,	18.3	87	0.2
8/7/2020	2:00:00 p, m,	17.8	91	0
8/7/2020	3:00:00 p, m,	18.3	89	0
8/7/2020	4:00:00 p, m,	17.9	93	0.2
8/7/2020	5:00:00 p, m,	17.6	91	0
8/7/2020	6:00:00 p, m,	17.2	91	0
8/7/2020	7:00:00 p, m,	17.1	87	0
8/7/2020	8:00:00 p, m,	16.9	83	0
8/7/2020	9:00:00 p, m,	17.2	83	0
8/7/2020	10:00:00 p, m	17.4	81	0
8/7/2020	11:00:00 p, m	17.2	82	0
8/8/2020	12:00:00 a, m	17	83	0

8/8/2020	1:00:00 a, m,	17	83	0
8/8/2020	2:00:00 a, m,	17.1	83	0
8/8/2020	3:00:00 a, m,	17	84	0
8/8/2020	4:00:00 a, m,	16.8	85	0
8/8/2020	5:00:00 a, m,	16.7	84	0
8/8/2020	6:00:00 a, m,	16.9	83	0
8/8/2020	7:00:00 a, m,	17.1	84	0
8/8/2020	8:00:00 a, m,	17.6	84	0
8/8/2020	9:00:00 a, m,	17.9	83	0
8/8/2020	10:00:00 a, m	18.3	85	0
8/8/2020	11:00:00 a, m	19.3	82	0
8/8/2020	12:00:00 p, m	19.8	82	0
8/8/2020	1:00:00 p, m,	20.8	75	0
8/8/2020	2:00:00 p, m,	23.3	63	0
8/8/2020	3:00:00 p, m,	21.6	67	0
8/8/2020	4:00:00 p, m,	20.9	71	0
8/8/2020	5:00:00 p, m,	21.9	66	0
8/8/2020	6:00:00 p, m,	20.8	69	0
8/8/2020	7:00:00 p, m,	19.3	72	0
8/8/2020	8:00:00 p, m,	18.9	72	0
8/8/2020	9:00:00 p, m,	18.4	71	0
8/8/2020	10:00:00 p, m	18.3	73	0
8/8/2020	11:00:00 p, m	18.6	72	0
8/9/2020	12:00:00 a, m	18.5	74	0
8/9/2020	1:00:00 a, m,	18	77	0
8/9/2020	2:00:00 a, m,	17.8	77	0
8/9/2020	3:00:00 a, m,	18.2	75	0
8/9/2020	4:00:00 a, m,	18.4	73	0
8/9/2020	5:00:00 a, m,	18.2	75	0
8/9/2020	6:00:00 a, m,	17.6	79	0
8/9/2020	7:00:00 a, m,	17.8	80	0
8/9/2020	8:00:00 a, m,	18.7	79	0
8/9/2020	9:00:00 a, m,	19.4	76	0
8/9/2020	10:00:00 a, m	21.6	62	0
8/9/2020	11:00:00 a, m	20	74	0
8/9/2020	12:00:00 p, m	19.6	74	0
8/9/2020	1:00:00 p, m,	20.3	70	0
8/9/2020	2:00:00 p, m,	20.8	67	0
8/9/2020	3:00:00 p, m,	20.2	65	0.2
8/9/2020	4:00:00 p, m,	20.6	68	0
8/9/2020	5:00:00 p, m,	20.2	67	0
8/9/2020	6:00:00 p, m,	19.9	67	0
8/9/2020	7:00:00 p, m,	19.2	68	0
8/9/2020	8:00:00 p, m,	18.9	69	0
8/9/2020	9:00:00 p, m,	19	68	0
8/9/2020	10:00:00 p, m	18.8	69	0
8/9/2020	11:00:00 p, m	17.8	72	0

8/10/2020	12:00:00 a, m	18.2	70	0
8/10/2020	1:00:00 a, m,	18.5	69	0
8/10/2020	2:00:00 a, m,	17.9	73	0
8/10/2020	3:00:00 a, m,	17.8	74	0
8/10/2020	4:00:00 a, m,	18.5	70	0
8/10/2020	5:00:00 a, m,	18.6	70	0
8/10/2020	6:00:00 a, m,	19.1	66	0
8/10/2020	7:00:00 a, m,	18.7	73	0
8/10/2020	8:00:00 a, m,	18.6	75	0
8/10/2020	9:00:00 a, m,	19.4	70	0
8/10/2020	10:00:00 a, m	20.3	73	0
8/10/2020	11:00:00 a, m	22.8	59	0
8/10/2020	12:00:00 p, m	24.8	53	0
8/10/2020	1:00:00 p, m,	23.6	54	0
8/10/2020	2:00:00 p, m,	23.8	59	0
8/10/2020	3:00:00 p, m,	24.1	56	0
8/10/2020	4:00:00 p, m,	23.9	58	0
8/10/2020	5:00:00 p, m,	24.6	55	0
8/10/2020	6:00:00 p, m,	23.7	59	0
8/10/2020	7:00:00 p, m,	21.4	61	0
8/10/2020	8:00:00 p, m,	20.6	63	0
8/10/2020	9:00:00 p, m,	20.7	61	0
8/10/2020	10:00:00 p, m	20.9	61	0
8/10/2020	11:00:00 p, m	20.9	61	0
8/11/2020	12:00:00 a, m	19.6	66	0
8/11/2020	1:00:00 a, m,	19.3	69	0
8/11/2020	2:00:00 a, m,	18.7	70	0
8/11/2020	3:00:00 a, m,	18.7	71	0
8/11/2020	4:00:00 a, m,	18.7	72	0
8/11/2020	5:00:00 a, m,	18.6	72	0
8/11/2020	6:00:00 a, m,	17.9	75	0
8/11/2020	7:00:00 a, m,	18.2	78	0
8/11/2020	8:00:00 a, m,	18.7	77	0
8/11/2020	9:00:00 a, m,	19.2	77	0
8/11/2020	10:00:00 a, m	20.3	73	0
8/11/2020	11:00:00 a, m	21	75	0
8/11/2020	12:00:00 p, m	21.6	74	0
8/11/2020	1:00:00 p, m,	22.9	66	0
8/11/2020	2:00:00 p, m,	23.1	71	0
8/11/2020	3:00:00 p, m,	23.9	65	0
8/11/2020	4:00:00 p, m,	25.6	59	0
8/11/2020	5:00:00 p, m,	24.7	58	0
8/11/2020	6:00:00 p, m,	23.3	62	0
8/11/2020	7:00:00 p, m,	21.9	64	0
8/11/2020	8:00:00 p, m,	21.1	66	0
8/11/2020	9:00:00 p, m,	20.9	65	0
8/11/2020	10:00:00 p, m	20.9	64	0

8/11/2020	11:00:00 p, m	20.3	66	0
8/12/2020	12:00:00 a, m	20	67	0
8/12/2020	1:00:00 a, m,	19.6	71	0
8/12/2020	2:00:00 a, m,	19.6	72	0
8/12/2020	3:00:00 a, m,	19.1	74	0
8/12/2020	4:00:00 a, m,	18.6	75	0
8/12/2020	5:00:00 a, m,	18.1	76	0
8/12/2020	6:00:00 a, m,	17.8	80	0
8/12/2020	7:00:00 a, m,	18	81	0
8/12/2020	8:00:00 a, m,	18.9	80	0
8/12/2020	9:00:00 a, m,	19.6	79	0
8/12/2020	10:00:00 a, m	20.1	82	0
8/12/2020	11:00:00 a, m	20.6	80	0
8/12/2020	12:00:00 p, m	21.1	78	0
8/12/2020	1:00:00 p, m,	20.2	92	1
8/12/2020	2:00:00 p, m,	21.9	88	0
8/12/2020	3:00:00 p, m,	22.4	80	0
8/12/2020	4:00:00 p, m,	23.9	71	0
8/12/2020	5:00:00 p, m,	23	75	0
8/12/2020	6:00:00 p, m,	22.3	75	0
8/12/2020	7:00:00 p, m,	21.3	74	0
8/12/2020	8:00:00 p, m,	20.4	77	0
8/12/2020	9:00:00 p, m,	20.4	75	0
8/12/2020	10:00:00 p, m	20.5	75	0
8/12/2020	11:00:00 p, m	20.3	77	0
8/13/2020	12:00:00 a, m	19.2	83	0
8/13/2020	1:00:00 a, m,	18.7	83	0
8/13/2020	2:00:00 a, m,	18.6	85	0
8/13/2020	3:00:00 a, m,	18.4	86	0
8/13/2020	4:00:00 a, m,	18.4	86	0
8/13/2020	5:00:00 a, m,	18.1	88	0
8/13/2020	6:00:00 a, m,	17.8	88	0
8/13/2020	7:00:00 a, m,	18	89	0
8/13/2020	8:00:00 a, m,	18.6	88	0
8/13/2020	9:00:00 a, m,	18.9	89	0
8/13/2020	10:00:00 a, m	18.7	92	0.4
8/13/2020	11:00:00 a, m	19.1	92	0
8/13/2020	12:00:00 p, m	19.8	92	0
8/13/2020	1:00:00 p, m,	20.3	92	0
8/13/2020	2:00:00 p, m,	20.8	86	0
8/13/2020	3:00:00 p, m,	20.9	85	0
8/13/2020	4:00:00 p, m,	20.8	84	0
8/13/2020	5:00:00 p, m,	19.8	84	0
8/13/2020	6:00:00 p, m,	19.7	84	0.2
8/13/2020	7:00:00 p, m,	19.3	80	0
8/13/2020	8:00:00 p, m,	19.3	80	0
8/13/2020	9:00:00 p, m,	19.7	75	0

8/13/2020	10:00:00 p, m	20.2	71	0
8/13/2020	11:00:00 p, m	19.8	70	0
8/14/2020	12:00:00 a, m	19.8	72	0
8/14/2020	1:00:00 a, m,	19.2	76	0
8/14/2020	2:00:00 a, m,	18.9	74	0
8/14/2020	3:00:00 a, m,	18.9	78	0
8/14/2020	4:00:00 a, m,	18.7	78	0
8/14/2020	5:00:00 a, m,	18.5	79	0
8/14/2020	6:00:00 a, m,	18.3	79	0
8/14/2020	7:00:00 a, m,	18.4	81	0
8/14/2020	8:00:00 a, m,	19.1	81	0
8/14/2020	9:00:00 a, m,	19.7	78	0
8/14/2020	10:00:00 a, m	20.3	79	0
8/14/2020	11:00:00 a, m	20.8	80	0
8/14/2020	12:00:00 p, m	21.3	79	0
8/14/2020	1:00:00 p, m,	21.8	75	0
8/14/2020	2:00:00 p, m,	23.1	69	0
8/14/2020	3:00:00 p, m,	22.9	73	0
8/14/2020	4:00:00 p, m,	23.3	72	0
8/14/2020	5:00:00 p, m,	23.8	68	0
8/14/2020	6:00:00 p, m,	23	69	0
8/14/2020	7:00:00 p, m,	21.2	71	0
8/14/2020	8:00:00 p, m,	20.7	72	0
8/14/2020	9:00:00 p, m,	20	76	0
8/14/2020	10:00:00 p, m	20.1	76	0
8/14/2020	11:00:00 p, m	20.1	75	0
8/15/2020	12:00:00 a, m	19.6	75	0
8/15/2020	1:00:00 a, m,	19.1	76	0
8/15/2020	2:00:00 a, m,	19.3	76	0
8/15/2020	3:00:00 a, m,	16.7	93	1.4
8/15/2020	4:00:00 a, m,	16.3	95	0.6
8/15/2020	5:00:00 a, m,	16.3	95	0
8/15/2020	6:00:00 a, m,	15.8	95	0.2
8/15/2020	7:00:00 a, m,	16.1	95	0
8/15/2020	8:00:00 a, m,	16.7	95	0
8/15/2020	9:00:00 a, m,	17.4	92	0
8/15/2020	10:00:00 a, m	17.9	92	0.2
8/15/2020	11:00:00 a, m	18.5	93	0.2
8/15/2020	12:00:00 p, m	18.7	92	0
8/15/2020	1:00:00 p, m,	19.4	90	0
8/15/2020	2:00:00 p, m,	20	91	0
8/15/2020	3:00:00 p, m,	21.2	87	0
8/15/2020	4:00:00 p, m,	20.8	89	0.2
8/15/2020	5:00:00 p, m,	20	83	0
8/15/2020	6:00:00 p, m,	19.8	83	0
8/15/2020	7:00:00 p, m,	18.9	79	0
8/15/2020	8:00:00 p, m,	18.1	83	0

8/15/2020	9:00:00 p, m,	17.9	83	0
8/15/2020	10:00:00 p, m	17.9	85	0
8/15/2020	11:00:00 p, m	17.8	85	0
8/16/2020	12:00:00 a, m	17.4	84	0
8/16/2020	1:00:00 a, m,	17.5	82	0
8/16/2020	2:00:00 a, m,	17.3	86	0
8/16/2020	3:00:00 a, m,	17.2	86	0
8/16/2020	4:00:00 a, m,	16.9	86	0
8/16/2020	5:00:00 a, m,	17.3	85	0
8/16/2020	6:00:00 a, m,	17.1	90	0
8/16/2020	7:00:00 a, m,	16.9	90	0
8/16/2020	8:00:00 a, m,	17.3	91	0
8/16/2020	9:00:00 a, m,	17.7	92	0
8/16/2020	10:00:00 a, m	18.7	90	0
8/16/2020	11:00:00 a, m	19.4	91	0
8/16/2020	12:00:00 p, m	20.2	87	0
8/16/2020	1:00:00 p, m,	21.1	82	0
8/16/2020	2:00:00 p, m,	20.9	84	0
8/16/2020	3:00:00 p, m,	20	90	0.2
8/16/2020	4:00:00 p, m,	20.1	92	0
8/16/2020	5:00:00 p, m,	19.8	90	0
8/16/2020	6:00:00 p, m,	18.8	89	0
8/16/2020	7:00:00 p, m,	17.3	87	0
8/16/2020	8:00:00 p, m,	17.3	89	0
8/16/2020	9:00:00 p, m,	17.3	88	0
8/16/2020	10:00:00 p, m	17.4	87	0
8/16/2020	11:00:00 p, m	17.2	87	0
8/17/2020	12:00:00 a, m	16.9	86	0
8/17/2020	1:00:00 a, m,	16.9	87	0
8/17/2020	2:00:00 a, m,	16.8	87	0
8/17/2020	3:00:00 a, m,	16.8	88	0
8/17/2020	4:00:00 a, m,	16.4	89	0
8/17/2020	5:00:00 a, m,	16.3	90	0
8/17/2020	6:00:00 a, m,	15.8	90	0
8/17/2020	7:00:00 a, m,	16.1	92	0
8/17/2020	8:00:00 a, m,	16.7	90	0
8/17/2020	9:00:00 a, m,	17.6	93	0
8/17/2020	10:00:00 a, m	17.8	94	0
8/17/2020	11:00:00 a, m	17.4	96	0.4
8/17/2020	12:00:00 p, m	17.7	97	0
8/17/2020	1:00:00 p, m,	18.2	97	0.2
8/17/2020	2:00:00 p, m,	18.4	97	0
8/17/2020	3:00:00 p, m,	18.1	97	0
8/17/2020	4:00:00 p, m,	17.8	96	0.2
8/17/2020	5:00:00 p, m,	17.7	97	0.2
8/17/2020	6:00:00 p, m,	17.3	97	0
8/17/2020	7:00:00 p, m,	16.7	96	0

8/17/2020	8:00:00 p, m,	16.3	93	0
8/17/2020	9:00:00 p, m,	16.1	93	0
8/17/2020	10:00:00 p, m	16.1	91	0
8/17/2020	11:00:00 p, m	16.2	92	0
8/18/2020	12:00:00 a, m	16.3	91	0
8/18/2020	1:00:00 a, m,	16.1	93	0
8/18/2020	2:00:00 a, m,	15.7	93	0
8/18/2020	3:00:00 a, m,	15.8	93	0
8/18/2020	4:00:00 a, m,	15.5	91	0
8/18/2020	5:00:00 a, m,	15	92	0
8/18/2020	6:00:00 a, m,	15.1	92	0
8/18/2020	7:00:00 a, m,	15.4	92	0
8/18/2020	8:00:00 a, m,	15.9	94	0
8/18/2020	9:00:00 a, m,	17	93	0
8/18/2020	10:00:00 a, m	18.4	93	0
8/18/2020	11:00:00 a, m	20.3	88	0
8/18/2020	12:00:00 p, m	19.1	91	0
8/18/2020	1:00:00 p, m,	19.2	94	1.8
8/18/2020	2:00:00 p, m,	18.6	96	2.6
8/18/2020	3:00:00 p, m,	17.7	96	0.8
8/18/2020	4:00:00 p, m,	17.8	95	0
8/18/2020	5:00:00 p, m,	17.6	95	0
8/18/2020	6:00:00 p, m,	17.7	92	0
8/18/2020	7:00:00 p, m,	17.4	92	0
8/18/2020	8:00:00 p, m,	17.2	92	0
8/18/2020	9:00:00 p, m,	17.3	93	0
8/18/2020	10:00:00 p, m	17.5	92	0
8/18/2020	11:00:00 p, m	17.4	92	0
8/19/2020	12:00:00 a, m	17.3	92	0
8/19/2020	1:00:00 a, m,	17.3	92	0
8/19/2020	2:00:00 a, m,	17.3	92	0
8/19/2020	3:00:00 a, m,	17.4	91	0
8/19/2020	4:00:00 a, m,	17.3	93	0
8/19/2020	5:00:00 a, m,	17.3	94	0
8/19/2020	6:00:00 a, m,	17.4	94	0
8/19/2020	7:00:00 a, m,	17.3	95	0
8/19/2020	8:00:00 a, m,	17.6	95	0
8/19/2020	9:00:00 a, m,	18.2	96	0
8/19/2020	10:00:00 a, m	18.4	97	0
8/19/2020	11:00:00 a, m	17.9	98	0
8/19/2020	12:00:00 p, m	17.7	98	0.2
8/19/2020	1:00:00 p, m,	17.8	97	1.2
8/19/2020	2:00:00 p, m,	18.7	97	1.2
8/19/2020	3:00:00 p, m,	19.3	97	0
8/19/2020	4:00:00 p, m,	19.2	96	0
8/19/2020	5:00:00 p, m,	19.4	97	0
8/19/2020	6:00:00 p, m,	19.3	95	0

8/19/2020	7:00:00 p, m,	18.7	96	0
8/19/2020	8:00:00 p, m,	18.4	95	0
8/19/2020	9:00:00 p, m,	17.9	96	0
8/19/2020	10:00:00 p, m	17.3	97	0
8/19/2020	11:00:00 p, m	16.8	95	0
8/20/2020	12:00:00 a, m	16.9	96	0
8/20/2020	1:00:00 a, m,	17.2	96	0
8/20/2020	2:00:00 a, m,	17.3	96	0
8/20/2020	3:00:00 a, m,	16.9	95	0
8/20/2020	4:00:00 a, m,	16.8	96	0
8/20/2020	5:00:00 a, m,	16.7	95	0
8/20/2020	6:00:00 a, m,	16.1	95	0
8/20/2020	7:00:00 a, m,	16.7	94	0
8/20/2020	8:00:00 a, m,	17.2	95	0
8/20/2020	9:00:00 a, m,	17.9	96	0
8/20/2020	10:00:00 a, m	18.7	97	0
8/20/2020	11:00:00 a, m	19	97	0
8/20/2020	12:00:00 p, m	19.5	94	0
8/20/2020	1:00:00 p, m,	19.8	96	0
8/20/2020	2:00:00 p, m,	20.2	97	0.4
8/20/2020	3:00:00 p, m,	20.3	95	0
8/20/2020	4:00:00 p, m,	21.4	88	0
8/20/2020	5:00:00 p, m,	21.1	91	0
8/20/2020	6:00:00 p, m,	19.9	93	0
8/20/2020	7:00:00 p, m,	19.1	91	0
8/20/2020	8:00:00 p, m,	18.6	91	0
8/20/2020	9:00:00 p, m,	18.2	86	0
8/20/2020	10:00:00 p, m	18.1	85	0
8/20/2020	11:00:00 p, m	17.9	85	0
8/21/2020	12:00:00 a, m	18	91	0
8/21/2020	1:00:00 a, m,	17.9	85	0
8/21/2020	2:00:00 a, m,	18	84	0
8/21/2020	3:00:00 a, m,	18.1	88	0
8/21/2020	4:00:00 a, m,	17.7	89	0
8/21/2020	5:00:00 a, m,	17.1	88	0
8/21/2020	6:00:00 a, m,	16.6	90	0
8/21/2020	7:00:00 a, m,	16.8	93	0
8/21/2020	8:00:00 a, m,	17.3	94	0
8/21/2020	9:00:00 a, m,	17.9	94	0.2
8/21/2020	10:00:00 a, m	18.6	95	0
8/21/2020	11:00:00 a, m	19.6	93	0
8/21/2020	12:00:00 p, m	20.2	90	0
8/21/2020	1:00:00 p, m,	21.6	87	0
8/21/2020	2:00:00 p, m,	20.9	89	0
8/21/2020	3:00:00 p, m,	22.2	80	0
8/21/2020	4:00:00 p, m,	23.2	78	0
8/21/2020	5:00:00 p, m,	19.4	87	0.6

8/21/2020	6:00:00 p, m,	17.5	90	0
8/21/2020	7:00:00 p, m,	16.6	91	0.6
8/21/2020	8:00:00 p, m,	16.4	88	0
8/21/2020	9:00:00 p, m,	16	92	0
8/21/2020	10:00:00 p, m	16.7	88	0
8/21/2020	11:00:00 p, m	16.9	85	0
8/22/2020	12:00:00 a, m	16.8	87	0
8/22/2020	1:00:00 a, m,	16.4	89	0
8/22/2020	2:00:00 a, m,	16.1	89	0
8/22/2020	3:00:00 a, m,	16.4	88	0
8/22/2020	4:00:00 a, m,	16.6	87	0
8/22/2020	5:00:00 a, m,	16.6	87	0
8/22/2020	6:00:00 a, m,	16.4	88	0
8/22/2020	7:00:00 a, m,	16.5	88	0
8/22/2020	8:00:00 a, m,	17.1	88	0
8/22/2020	9:00:00 a, m,	17.8	88	0
8/22/2020	10:00:00 a, m	18.3	91	0
8/22/2020	11:00:00 a, m	18.7	87	0
8/22/2020	12:00:00 p, m	20.1	86	0
8/22/2020	1:00:00 p, m,	21.2	86	0
8/22/2020	2:00:00 p, m,	20.6	88	0
8/22/2020	3:00:00 p, m,	21.2	84	0
8/22/2020	4:00:00 p, m,	21.9	78	0
8/22/2020	5:00:00 p, m,	21.7	79	0
8/22/2020	6:00:00 p, m,	21.2	78	0
8/22/2020	7:00:00 p, m,	19.7	78	0
8/22/2020	8:00:00 p, m,	19.4	76	0
8/22/2020	9:00:00 p, m,	19.4	76	0
8/22/2020	10:00:00 p, m	19.3	73	0
8/22/2020	11:00:00 p, m	18.7	76	0
8/23/2020	12:00:00 a, m	18.7	76	0
8/23/2020	1:00:00 a, m,	18.3	80	0
8/23/2020	2:00:00 a, m,	17.8	82	0
8/23/2020	3:00:00 a, m,	17.6	83	0
8/23/2020	4:00:00 a, m,	17.4	83	0
8/23/2020	5:00:00 a, m,	17.5	84	0
8/23/2020	6:00:00 a, m,	17.2	85	0
8/23/2020	7:00:00 a, m,	17.2	87	0
8/23/2020	8:00:00 a, m,	17.3	87	0
8/23/2020	9:00:00 a, m,	17.7	88	0
8/23/2020	10:00:00 a, m	18.8	87	0
8/23/2020	11:00:00 a, m	19.7	83	0
8/23/2020	12:00:00 p, m	20.3	84	0
8/23/2020	1:00:00 p, m,	20.8	84	0
8/23/2020	2:00:00 p, m,	21.7	80	0
8/23/2020	3:00:00 p, m,	22.9	74	0
8/23/2020	4:00:00 p, m,	24.3	67	0

8/23/2020	5:00:00 p, m,	22.9	69	0
8/23/2020	6:00:00 p, m,	20.9	71	0
8/23/2020	7:00:00 p, m,	19.8	73	0.2
8/23/2020	8:00:00 p, m,	18.9	78	0
8/23/2020	9:00:00 p, m,	17.9	83	0
8/23/2020	10:00:00 p, m	18.1	77	0
8/23/2020	11:00:00 p, m	17.5	78	0
8/24/2020	12:00:00 a, m	17.4	79	0
8/24/2020	1:00:00 a, m,	17.3	80	0
8/24/2020	2:00:00 a, m,	17.1	79	0
8/24/2020	3:00:00 a, m,	16.6	81	0
8/24/2020	4:00:00 a, m,	16.3	81	0
8/24/2020	5:00:00 a, m,	16.2	82	0
8/24/2020	6:00:00 a, m,	15.9	83	0
8/24/2020	7:00:00 a, m,	16.1	84	0
8/24/2020	8:00:00 a, m,	16.1	84	0
8/24/2020	9:00:00 a, m,	16.7	87	0
8/24/2020	10:00:00 a, m	18.1	87	0
8/24/2020	11:00:00 a, m	20.3	81	0
8/24/2020	12:00:00 p, m	22.4	73	0
8/24/2020	1:00:00 p, m,	23.4	69	0
8/24/2020	2:00:00 p, m,	24.4	66	0
8/24/2020	3:00:00 p, m,	24.7	63	0
8/24/2020	4:00:00 p, m,	25.4	54	0
8/24/2020	5:00:00 p, m,	24.6	57	0
8/24/2020	6:00:00 p, m,	22.7	57	0
8/24/2020	7:00:00 p, m,	20.9	60	0
8/24/2020	8:00:00 p, m,	20.4	61	0
8/24/2020	9:00:00 p, m,	19.9	60	0
8/24/2020	10:00:00 p, m	19.5	63	0
8/24/2020	11:00:00 p, m	18.6	66	0
8/25/2020	12:00:00 a, m	17.8	70	0
8/25/2020	1:00:00 a, m,	17.5	70	0
8/25/2020	2:00:00 a, m,	17.2	72	0
8/25/2020	3:00:00 a, m,	16.8	74	0
8/25/2020	4:00:00 a, m,	16.8	74	0
8/25/2020	5:00:00 a, m,	16.6	73	0
8/25/2020	6:00:00 a, m,	16.7	74	0
8/25/2020	7:00:00 a, m,	16.9	77	0
8/25/2020	8:00:00 a, m,	17.1	78	0
8/25/2020	9:00:00 a, m,	17.8	76	0
8/25/2020	10:00:00 a, m	19	78	0
8/25/2020	11:00:00 a, m	20.1	77	0
8/25/2020	12:00:00 p, m	21.6	67	0
8/25/2020	1:00:00 p, m,	23.4	63	0
8/25/2020	2:00:00 p, m,	24.1	58	0
8/25/2020	3:00:00 p, m,	23.8	61	0

8/25/2020	4:00:00 p, m,	21.9	65	0
8/25/2020	5:00:00 p, m,	22.6	66	0
8/25/2020	6:00:00 p, m,	21.6	65	0
8/25/2020	7:00:00 p, m,	20.1	69	0
8/25/2020	8:00:00 p, m,	19.7	69	0
8/25/2020	9:00:00 p, m,	19.6	69	0
8/25/2020	10:00:00 p, m	19	70	0
8/25/2020	11:00:00 p, m	18.6	72	0
8/26/2020	12:00:00 a, m	18.3	72	0
8/26/2020	1:00:00 a, m,	17.6	75	0
8/26/2020	2:00:00 a, m,	17.3	74	0
8/26/2020	3:00:00 a, m,	17.1	75	0
8/26/2020	4:00:00 a, m,	17.8	75	0
8/26/2020	5:00:00 a, m,	17.3	77	0
8/26/2020	6:00:00 a, m,	17.4	78	0
8/26/2020	7:00:00 a, m,	17.2	79	0
8/26/2020	8:00:00 a, m,	17.4	81	0
8/26/2020	9:00:00 a, m,	18	80	0
8/26/2020	10:00:00 a, m	18.8	81	0
8/26/2020	11:00:00 a, m	20.4	74	0
8/26/2020	12:00:00 p, m	21.5	71	0
8/26/2020	1:00:00 p, m,	23.3	63	0
8/26/2020	2:00:00 p, m,	22.4	69	0
8/26/2020	3:00:00 p, m,	21.7	78	0.2
8/26/2020	4:00:00 p, m,	22.2	73	0
8/26/2020	5:00:00 p, m,	22.5	67	0
8/26/2020	6:00:00 p, m,	21.6	68	0
8/26/2020	7:00:00 p, m,	21.1	68	0
8/26/2020	8:00:00 p, m,	20.4	67	0
8/26/2020	9:00:00 p, m,	20.2	71	0
8/26/2020	10:00:00 p, m	19.2	71	0
8/26/2020	11:00:00 p, m	18.8	82	0
8/27/2020	12:00:00 a, m	18.6	84	0
8/27/2020	1:00:00 a, m,	18.4	85	0
8/27/2020	2:00:00 a, m,	18	85	0
8/27/2020	3:00:00 a, m,	17.7	86	0
8/27/2020	4:00:00 a, m,	17.4	87	0
8/27/2020	5:00:00 a, m,	17.2	86	0
8/27/2020	6:00:00 a, m,	17.1	86	0
8/27/2020	7:00:00 a, m,	17.4	88	0
8/27/2020	8:00:00 a, m,	17.7	88	0
8/27/2020	9:00:00 a, m,	18.3	91	0
8/27/2020	10:00:00 a, m	18.5	91	0
8/27/2020	11:00:00 a, m	19.4	89	0
8/27/2020	12:00:00 p, m	19.7	87	0
8/27/2020	1:00:00 p, m,	19.7	88	0
8/27/2020	2:00:00 p, m,	19.2	89	0

8/27/2020	3:00:00 p, m,	19.3	90	0
8/27/2020	4:00:00 p, m,	19.3	91	0
8/27/2020	5:00:00 p, m,	18.7	92	0.2
8/27/2020	6:00:00 p, m,	18.6	90	0
8/27/2020	7:00:00 p, m,	17.8	85	0
8/27/2020	8:00:00 p, m,	18.3	80	0
8/27/2020	9:00:00 p, m,	18.3	80	0
8/27/2020	10:00:00 p, m	18.3	80	0
8/27/2020	11:00:00 p, m	18.3	80	0
8/28/2020	12:00:00 a, m	18.1	79	0
8/28/2020	1:00:00 a, m,	17.7	80	0
8/28/2020	2:00:00 a, m,	17.4	80	0
8/28/2020	3:00:00 a, m,	17.3	81	0
8/28/2020	4:00:00 a, m,	17.5	80	0
8/28/2020	5:00:00 a, m,	17.1	87	0.2
8/28/2020	6:00:00 a, m,	15.3	92	0.2
8/28/2020	7:00:00 a, m,	15.6	92	0
8/28/2020	8:00:00 a, m,	16.6	91	0.2
8/28/2020	9:00:00 a, m,	17.4	92	0
8/28/2020	10:00:00 a, m	18.3	90	0
8/28/2020	11:00:00 a, m	19.9	85	0
8/28/2020	12:00:00 p, m	21.4	76	0
8/28/2020	1:00:00 p, m,	23	66	0
8/28/2020	2:00:00 p, m,	23.8	67	0
8/28/2020	3:00:00 p, m,	23.8	64	0
8/28/2020	4:00:00 p, m,	23.5	66	0
8/28/2020	5:00:00 p, m,	22.4	70	0
8/28/2020	6:00:00 p, m,	21.4	67	0
8/28/2020	7:00:00 p, m,	20.7	68	0
8/28/2020	8:00:00 p, m,	20.1	71	0
8/28/2020	9:00:00 p, m,	20.1	70	0
8/28/2020	10:00:00 p, m	19.6	74	0
8/28/2020	11:00:00 p, m	19.3	75	0
8/29/2020	12:00:00 a, m	19.3	77	0
8/29/2020	1:00:00 a, m,	18.8	78	0
8/29/2020	2:00:00 a, m,	19	85	0.2
8/29/2020	3:00:00 a, m,	16.6	92	0.6
8/29/2020	4:00:00 a, m,	16.8	93	0
8/29/2020	5:00:00 a, m,	16.6	94	0.2
8/29/2020	6:00:00 a, m,	16.4	95	0
8/29/2020	7:00:00 a, m,	16.7	95	0
8/29/2020	8:00:00 a, m,	16.8	96	0
8/29/2020	9:00:00 a, m,	17.2	97	0
8/29/2020	10:00:00 a, m	18.1	96	0
8/29/2020	11:00:00 a, m	19.3	92	0
8/29/2020	12:00:00 p, m	19.7	90	0
8/29/2020	1:00:00 p, m,	20.6	85	0

8/29/2020	2:00:00 p, m,	19.4	93	1.6
8/29/2020	3:00:00 p, m,	20.2	91	0
8/29/2020	4:00:00 p, m,	19.7	87	0
8/29/2020	5:00:00 p, m,	19.6	88	0
8/29/2020	6:00:00 p, m,	19.3	85	0
8/29/2020	7:00:00 p, m,	19	83	0
8/29/2020	8:00:00 p, m,	18.9	80	0
8/29/2020	9:00:00 p, m,	18.8	74	0
8/29/2020	10:00:00 p, m	18.2	79	0
8/29/2020	11:00:00 p, m	17.8	81	0.2
8/30/2020	12:00:00 a, m	17.7	86	0
8/30/2020	1:00:00 a, m,	17.6	87	0
8/30/2020	2:00:00 a, m,	16.4	95	4.8
8/30/2020	3:00:00 a, m,	14.6	95	12.4
8/30/2020	4:00:00 a, m,	14.2	96	1
8/30/2020	5:00:00 a, m,	14.1	97	0
8/30/2020	6:00:00 a, m,	14	97	0.2
8/30/2020	7:00:00 a, m,	14.1	97	0
8/30/2020	8:00:00 a, m,	14.4	97	0
8/30/2020	9:00:00 a, m,	15.4	98	0
8/30/2020	10:00:00 a, m	15.8	98	0
8/30/2020	11:00:00 a, m	16.4	98	0
8/30/2020	12:00:00 p, m	16.9	98	0
8/30/2020	1:00:00 p, m,	17.2	97	0
8/30/2020	2:00:00 p, m,	17.4	98	0
8/30/2020	3:00:00 p, m,	18.2	97	0
8/30/2020	4:00:00 p, m,	18.1	96	0
8/30/2020	5:00:00 p, m,	18.2	97	0
8/30/2020	6:00:00 p, m,	18.3	97	0
8/30/2020	7:00:00 p, m,	17.3	96	0
8/30/2020	8:00:00 p, m,	17.2	97	0
8/30/2020	9:00:00 p, m,	17.2	96	0
8/30/2020	10:00:00 p, m	17	96	0
8/30/2020	11:00:00 p, m	16.7	96	0
8/31/2020	12:00:00 a, m	16.7	96	0
8/31/2020	1:00:00 a, m,	16.4	95	0
8/31/2020	2:00:00 a, m,	16.3	95	0
8/31/2020	3:00:00 a, m,	16.3	95	0
8/31/2020	4:00:00 a, m,	15.9	94	0
8/31/2020	5:00:00 a, m,	15.6	95	0
8/31/2020	6:00:00 a, m,	15.3	95	0
8/31/2020	7:00:00 a, m,	15.7	97	0
8/31/2020	8:00:00 a, m,	16.6	96	0
8/31/2020	9:00:00 a, m,	17.3	96	0
8/31/2020	10:00:00 a, m	18.4	93	0
8/31/2020	11:00:00 a, m	19.3	91	0
8/31/2020	12:00:00 p, m	19.8	92	0

8/31/2020	1:00:00 p, m,	21.2	86	0
8/31/2020	2:00:00 p, m,	20.3	85	0
8/31/2020	3:00:00 p, m,	19.2	87	0
8/31/2020	4:00:00 p, m,	18.6	87	0
8/31/2020	5:00:00 p, m,	19.7	80	0
8/31/2020	6:00:00 p, m,	19	81	0
8/31/2020	7:00:00 p, m,	17.9	81	0
8/31/2020	8:00:00 p, m,	18.3	76	0
8/31/2020	9:00:00 p, m,	17.7	80	0
8/31/2020	10:00:00 p, m	17.4	80	0
8/31/2020	11:00:00 p, m	18	74	0
Aug_20		18.7400538	80.4663978	38.4
9/1/2020	12:00:00 a, m	18.1	76	0
9/1/2020	1:00:00 a, m,	18.1	74	0
9/1/2020	2:00:00 a, m,	17.7	76	0
9/1/2020	3:00:00 a, m,	16.8	78	0
9/1/2020	4:00:00 a, m,	16.3	81	0
9/1/2020	5:00:00 a, m,	15.9	82	0
9/1/2020	6:00:00 a, m,	15.7	83	0
9/1/2020	7:00:00 a, m,	16.1	86	0
9/1/2020	8:00:00 a, m,	17.3	85	0
9/1/2020	9:00:00 a, m,	18.1	84	0
9/1/2020	10:00:00 a, m	19	84	0
9/1/2020	11:00:00 a, m	19.6	81	0
9/1/2020	12:00:00 p, m	20.4	80	0
9/1/2020	1:00:00 p, m,	21.3	79	0
9/1/2020	2:00:00 p, m,	20.9	79	0
9/1/2020	3:00:00 p, m,	21.3	76	0
9/1/2020	4:00:00 p, m,	22.2	76	0
9/1/2020	5:00:00 p, m,	21.1	82	0
9/1/2020	6:00:00 p, m,	20.1	80	0
9/1/2020	7:00:00 p, m,	18.5	80	0
9/1/2020	8:00:00 p, m,	17.9	79	0
9/1/2020	9:00:00 p, m,	17.6	77	0
9/1/2020	10:00:00 p, m	17.8	74	0
9/1/2020	11:00:00 p, m	18.1	71	0
9/2/2020	12:00:00 a, m	18.6	68	0
9/2/2020	1:00:00 a, m,	17.9	71	0
9/2/2020	2:00:00 a, m,	17.6	70	0
9/2/2020	3:00:00 a, m,	17.7	71	0
9/2/2020	4:00:00 a, m,	17.6	72	0
9/2/2020	5:00:00 a, m,	17.5	74	0
9/2/2020	6:00:00 a, m,	16.7	75	0
9/2/2020	7:00:00 a, m,	16.8	78	0
9/2/2020	8:00:00 a, m,	17.6	79	0.2
9/2/2020	9:00:00 a, m,	18.3	77	0
9/2/2020	10:00:00 a, m	18.9	82	0

9/2/2020	11:00:00 a, m	19.8	76	0
9/2/2020	12:00:00 p, m	19.8	81	0
9/2/2020	1:00:00 p, m,	20.4	79	0
9/2/2020	2:00:00 p, m,	20.4	79	0
9/2/2020	3:00:00 p, m,	21.2	71	0
9/2/2020	4:00:00 p, m,	20.6	76	0
9/2/2020	5:00:00 p, m,	20.7	76	0
9/2/2020	6:00:00 p, m,	20.8	74	0
9/2/2020	7:00:00 p, m,	19.3	74	0
9/2/2020	8:00:00 p, m,	18.2	76	0
9/2/2020	9:00:00 p, m,	18.6	74	0
9/2/2020	10:00:00 p, m	18.2	75	0
9/2/2020	11:00:00 p, m	17.6	76	0
9/3/2020	12:00:00 a, m	17.6	75	0
9/3/2020	1:00:00 a, m,	17.5	76	0
9/3/2020	2:00:00 a, m,	17.3	76	0
9/3/2020	3:00:00 a, m,	16.9	77	0
9/3/2020	4:00:00 a, m,	16.6	78	0
9/3/2020	5:00:00 a, m,	16.4	78	0
9/3/2020	6:00:00 a, m,	16.2	80	0
9/3/2020	7:00:00 a, m,	16.4	81	0
9/3/2020	8:00:00 a, m,	16.7	83	0
9/3/2020	9:00:00 a, m,	17.9	81	0
9/3/2020	10:00:00 a, m	19.4	80	0
9/3/2020	11:00:00 a, m	20.4	76	0
9/3/2020	12:00:00 p, m	21.1	77	0
9/3/2020	1:00:00 p, m,	22.8	68	0
9/3/2020	2:00:00 p, m,	21.3	77	0
9/3/2020	3:00:00 p, m,	22.1	70	0
9/3/2020	4:00:00 p, m,	23.4	68	0
9/3/2020	5:00:00 p, m,	22.8	70	0
9/3/2020	6:00:00 p, m,	22	69	0
9/3/2020	7:00:00 p, m,	20.7	71	0
9/3/2020	8:00:00 p, m,	20.8	66	0
9/3/2020	9:00:00 p, m,	20.3	69	0
9/3/2020	10:00:00 p, m	20.6	67	0
9/3/2020	11:00:00 p, m	20.2	68	0
9/4/2020	12:00:00 a, m	19.9	70	0
9/4/2020	1:00:00 a, m,	19.6	75	0
9/4/2020	2:00:00 a, m,	18.8	77	0
9/4/2020	3:00:00 a, m,	17.4	88	0.2
9/4/2020	4:00:00 a, m,	16.4	92	0
9/4/2020	5:00:00 a, m,	16.2	94	0.2
9/4/2020	6:00:00 a, m,	15.7	96	1.6
9/4/2020	7:00:00 a, m,	15.8	97	0.6
9/4/2020	8:00:00 a, m,	16.3	97	0.4
9/4/2020	9:00:00 a, m,	16.9	97	0

9/4/2020	10:00:00 a, m	17.8	98	0
9/4/2020	11:00:00 a, m	18.2	97	0
9/4/2020	12:00:00 p, m	18.8	95	0
9/4/2020	1:00:00 p, m,	18.7	95	1.2
9/4/2020	2:00:00 p, m,	18.8	96	0.4
9/4/2020	3:00:00 p, m,	19.9	95	0
9/4/2020	4:00:00 p, m,	20.6	91	0
9/4/2020	5:00:00 p, m,	20.6	90	0
9/4/2020	6:00:00 p, m,	20.2	91	0
9/4/2020	7:00:00 p, m,	18.3	86	0
9/4/2020	8:00:00 p, m,	17.9	86	0
9/4/2020	9:00:00 p, m,	18.1	83	0
9/4/2020	10:00:00 p, m	18.4	80	0
9/4/2020	11:00:00 p, m	17.9	82	0
9/5/2020	12:00:00 a, m	17.9	83	0
9/5/2020	1:00:00 a, m,	18.2	81	0
9/5/2020	2:00:00 a, m,	17.4	84	0
9/5/2020	3:00:00 a, m,	17.1	86	0
9/5/2020	4:00:00 a, m,	16.7	85	0
9/5/2020	5:00:00 a, m,	16.8	87	0
9/5/2020	6:00:00 a, m,	17.1	85	0
9/5/2020	7:00:00 a, m,	17.6	88	0
9/5/2020	8:00:00 a, m,	17.9	87	0
9/5/2020	9:00:00 a, m,	18.5	87	0
9/5/2020	10:00:00 a, m	18.9	88	0
9/5/2020	11:00:00 a, m	19.8	86	0
9/5/2020	12:00:00 p, m	21.8	79	0
9/5/2020	1:00:00 p, m,	23.4	74	0
9/5/2020	2:00:00 p, m,	23.2	74	0
9/5/2020	3:00:00 p, m,	24.1	61	0
9/5/2020	4:00:00 p, m,	23.4	66	0
9/5/2020	5:00:00 p, m,	23.7	63	0
9/5/2020	6:00:00 p, m,	22.4	66	0
9/5/2020	7:00:00 p, m,	20.5	67	0
9/5/2020	8:00:00 p, m,	20.9	60	0
9/5/2020	9:00:00 p, m,	20.1	69	0
9/5/2020	10:00:00 p, m	20.1	69	0
9/5/2020	11:00:00 p, m	19.7	69	0
9/6/2020	12:00:00 a, m	19.2	73	0
9/6/2020	1:00:00 a, m,	18.5	71	0
9/6/2020	2:00:00 a, m,	17.9	73	0
9/6/2020	3:00:00 a, m,	17.4	77	0
9/6/2020	4:00:00 a, m,	17.6	76	0
9/6/2020	5:00:00 a, m,	16.8	79	0
9/6/2020	6:00:00 a, m,	16.6	79	0
9/6/2020	7:00:00 a, m,	16.4	80	0
9/6/2020	8:00:00 a, m,	15.7	89	0.2

9/6/2020	9:00:00 a, m,	17.3	85	0
9/6/2020	10:00:00 a, m	17.4	84	0
9/6/2020	11:00:00 a, m	18.1	85	0
9/6/2020	12:00:00 p, m	19.7	79	0
9/6/2020	1:00:00 p, m,	20.2	80	0
9/6/2020	2:00:00 p, m,	22.3	74	0
9/6/2020	3:00:00 p, m,	22	69	0
9/6/2020	4:00:00 p, m,	21.9	72	0
9/6/2020	5:00:00 p, m,	21.4	69	0
9/6/2020	6:00:00 p, m,	21	70	0
9/6/2020	7:00:00 p, m,	19.3	73	0
9/6/2020	8:00:00 p, m,	19.2	71	0
9/6/2020	9:00:00 p, m,	19.2	68	0
9/6/2020	10:00:00 p, m	19.4	68	0
9/6/2020	11:00:00 p, m	18.4	72	0
9/7/2020	12:00:00 a, m	18.5	73	0
9/7/2020	1:00:00 a, m,	18.3	74	0
9/7/2020	2:00:00 a, m,	18.2	75	0
9/7/2020	3:00:00 a, m,	17.8	74	0
9/7/2020	4:00:00 a, m,	16.9	76	0
9/7/2020	5:00:00 a, m,	16.7	76	0
9/7/2020	6:00:00 a, m,	16.6	77	0
9/7/2020	7:00:00 a, m,	16.9	80	0
9/7/2020	8:00:00 a, m,	17.3	83	0
9/7/2020	9:00:00 a, m,	17.9	83	0
9/7/2020	10:00:00 a, m	18.9	85	0
9/7/2020	11:00:00 a, m	20.1	81	0
9/7/2020	12:00:00 p, m	20.3	83	0
9/7/2020	1:00:00 p, m,	21.7	75	0
9/7/2020	2:00:00 p, m,	24.1	65	0
9/7/2020	3:00:00 p, m,	23.8	64	0
9/7/2020	4:00:00 p, m,	22.8	70	0
9/7/2020	5:00:00 p, m,	21.2	77	0
9/7/2020	6:00:00 p, m,	20.9	71	0
9/7/2020	7:00:00 p, m,	20.7	71	0
9/7/2020	8:00:00 p, m,	20.2	71	0
9/7/2020	9:00:00 p, m,	20.3	71	0
9/7/2020	10:00:00 p, m	18.5	82	0
9/7/2020	11:00:00 p, m	16.4	93	0.2
9/8/2020	12:00:00 a, m	16.2	90	0.2
9/8/2020	1:00:00 a, m,	16.3	88	0
9/8/2020	2:00:00 a, m,	16.7	88	0
9/8/2020	3:00:00 a, m,	16.4	86	0
9/8/2020	4:00:00 a, m,	16.6	86	0
9/8/2020	5:00:00 a, m,	16.6	86	0
9/8/2020	6:00:00 a, m,	16.3	87	0
9/8/2020	7:00:00 a, m,	16.8	88	0

9/8/2020	8:00:00 a, m,	17.1	90	0
9/8/2020	9:00:00 a, m,	17.4	91	0
9/8/2020	10:00:00 a, m	17.9	93	0
9/8/2020	11:00:00 a, m	18.7	92	0
9/8/2020	12:00:00 p, m	18.7	93	0
9/8/2020	1:00:00 p, m,	20	83	0
9/8/2020	2:00:00 p, m,	21.7	86	0
9/8/2020	3:00:00 p, m,	19.7	88	0
9/8/2020	4:00:00 p, m,	20.5	83	0
9/8/2020	5:00:00 p, m,	21.1	79	0
9/8/2020	6:00:00 p, m,	20.7	81	0
9/8/2020	7:00:00 p, m,	19.2	86	0
9/8/2020	8:00:00 p, m,	18.7	84	0
9/8/2020	9:00:00 p, m,	17.7	85	0
9/8/2020	10:00:00 p, m	17.3	85	0
9/8/2020	11:00:00 p, m	17.4	83	0
9/9/2020	12:00:00 a, m	17	87	0
9/9/2020	1:00:00 a, m,	16.2	86	0
9/9/2020	2:00:00 a, m,	16.3	84	0
9/9/2020	3:00:00 a, m,	15.8	85	0
9/9/2020	4:00:00 a, m,	15.5	86	0
9/9/2020	5:00:00 a, m,	15.6	85	0
9/9/2020	6:00:00 a, m,	16.1	85	0
9/9/2020	7:00:00 a, m,	16.4	87	0
9/9/2020	8:00:00 a, m,	17	87	0
9/9/2020	9:00:00 a, m,	17.7	85	0
9/9/2020	10:00:00 a, m	18.6	86	0
9/9/2020	11:00:00 a, m	19.3	85	0
9/9/2020	12:00:00 p, m	20.5	81	0
9/9/2020	1:00:00 p, m,	21.1	77	0
9/9/2020	2:00:00 p, m,	21.4	76	0
9/9/2020	3:00:00 p, m,	21.5	75	0
9/9/2020	4:00:00 p, m,	20.8	80	0
9/9/2020	5:00:00 p, m,	20.8	79	0
9/9/2020	6:00:00 p, m,	20.1	73	0.2
9/9/2020	7:00:00 p, m,	19.3	75	0
9/9/2020	8:00:00 p, m,	19.2	71	0
9/9/2020	9:00:00 p, m,	19.2	71	0
9/9/2020	10:00:00 p, m	19.3	70	0
9/9/2020	11:00:00 p, m	19.1	71	0
9/10/2020	12:00:00 a, m	18.7	72	0
9/10/2020	1:00:00 a, m,	18.4	73	0
9/10/2020	2:00:00 a, m,	17.9	74	0
9/10/2020	3:00:00 a, m,	17.8	74	0
9/10/2020	4:00:00 a, m,	17.3	77	0
9/10/2020	5:00:00 a, m,	17.5	76	0
9/10/2020	6:00:00 a, m,	17.7	77	0

9/10/2020	7:00:00 a, m,	17.7	81	0
9/10/2020	8:00:00 a, m,	18.3	81	0
9/10/2020	9:00:00 a, m,	18.9	77	0
9/10/2020	10:00:00 a, m	19.6	79	0
9/10/2020	11:00:00 a, m	20.3	79	0
9/10/2020	12:00:00 p, m	18.2	88	0.4
9/10/2020	1:00:00 p, m,	18.9	84	0
9/10/2020	2:00:00 p, m,	23.2	66	0
9/10/2020	3:00:00 p, m,	22.9	63	0.2
9/10/2020	4:00:00 p, m,	22.9	64	0
9/10/2020	5:00:00 p, m,	21.2	68	0
9/10/2020	6:00:00 p, m,	20.4	71	0
9/10/2020	7:00:00 p, m,	18.8	74	0
9/10/2020	8:00:00 p, m,	18.2	74	0
9/10/2020	9:00:00 p, m,	18.4	75	0
9/10/2020	10:00:00 p, m	18.8	71	0
9/10/2020	11:00:00 p, m	19.3	68	0
9/11/2020	12:00:00 a, m	19.2	67	0
9/11/2020	1:00:00 a, m,	19.2	66	0
9/11/2020	2:00:00 a, m,	18.8	68	0
9/11/2020	3:00:00 a, m,	18.2	68	0
9/11/2020	4:00:00 a, m,	17.7	73	0
9/11/2020	5:00:00 a, m,	17.4	74	0
9/11/2020	6:00:00 a, m,	17.6	74	0
9/11/2020	7:00:00 a, m,	18.1	73	0
9/11/2020	8:00:00 a, m,	18.9	74	0
9/11/2020	9:00:00 a, m,	19.5	76	0
9/11/2020	10:00:00 a, m	19.7	72	0
9/11/2020	11:00:00 a, m	20.1	74	0
9/11/2020	12:00:00 p, m	20.9	74	0
9/11/2020	1:00:00 p, m,	23.3	65	0
9/11/2020	2:00:00 p, m,	22.9	66	0
9/11/2020	3:00:00 p, m,	23.5	66	0
9/11/2020	4:00:00 p, m,	23.2	65	0
9/11/2020	5:00:00 p, m,	23.1	68	0
9/11/2020	6:00:00 p, m,	22.1	66	0
9/11/2020	7:00:00 p, m,	20.8	67	0
9/11/2020	8:00:00 p, m,	20.8	68	0
9/11/2020	9:00:00 p, m,	20.4	68	0
9/11/2020	10:00:00 p, m	20.3	67	0
9/11/2020	11:00:00 p, m	20.3	66	0
9/12/2020	12:00:00 a, m	19.6	67	0
9/12/2020	1:00:00 a, m,	19.3	70	0
9/12/2020	2:00:00 a, m,	19.7	68	0
9/12/2020	3:00:00 a, m,	19	70	0
9/12/2020	4:00:00 a, m,	18.4	71	0
9/12/2020	5:00:00 a, m,	18.4	69	0

9/12/2020	6:00:00 a, m,	17.5	74	0
9/12/2020	7:00:00 a, m,	18.3	77	0
9/12/2020	8:00:00 a, m,	18.5	78	0
9/12/2020	9:00:00 a, m,	19.4	77	0
9/12/2020	10:00:00 a, m	20.2	76	0
9/12/2020	11:00:00 a, m	21.6	70	0
9/12/2020	12:00:00 p, m	22.1	67	0
9/12/2020	1:00:00 p, m,	22.7	67	0
9/12/2020	2:00:00 p, m,	23.5	66	0
9/12/2020	3:00:00 p, m,	23.1	65	0
9/12/2020	4:00:00 p, m,	21.6	66	0
9/12/2020	5:00:00 p, m,	21.8	65	0
9/12/2020	6:00:00 p, m,	21.2	64	0
9/12/2020	7:00:00 p, m,	20.7	67	0
9/12/2020	8:00:00 p, m,	20.6	67	0
9/12/2020	9:00:00 p, m,	20.3	67	0
9/12/2020	10:00:00 p, m	19.6	70	0
9/12/2020	11:00:00 p, m	19.9	68	0
9/13/2020	12:00:00 a, m	19.8	68	0
9/13/2020	1:00:00 a, m,	19.6	71	0
9/13/2020	2:00:00 a, m,	19	73	0
9/13/2020	3:00:00 a, m,	17.4	78	0
9/13/2020	4:00:00 a, m,	17.9	73	0
9/13/2020	5:00:00 a, m,	17.8	72	0
9/13/2020	6:00:00 a, m,	18.2	72	0
9/13/2020	7:00:00 a, m,	18.7	73	0
9/13/2020	8:00:00 a, m,	19.3	74	0
9/13/2020	9:00:00 a, m,	20.5	71	0
9/13/2020	10:00:00 a, m	21.1	69	0
9/13/2020	11:00:00 a, m	20.4	74	0
9/13/2020	12:00:00 p, m	20.5	79	0
9/13/2020	1:00:00 p, m,	16.8	93	1.4
9/13/2020	2:00:00 p, m,	16	91	0.2
9/13/2020	3:00:00 p, m,	17.6	88	0
9/13/2020	4:00:00 p, m,	18.6	83	0
9/13/2020	5:00:00 p, m,	18.9	78	0
9/13/2020	6:00:00 p, m,	18.2	80	0
9/13/2020	7:00:00 p, m,	17.1	80	0
9/13/2020	8:00:00 p, m,	16.8	82	0
9/13/2020	9:00:00 p, m,	17.2	81	0
9/13/2020	10:00:00 p, m	17.1	81	0
9/13/2020	11:00:00 p, m	17.1	79	0
9/14/2020	12:00:00 a, m	16.3	81	0
9/14/2020	1:00:00 a, m,	16.3	81	0
9/14/2020	2:00:00 a, m,	16.7	77	0
9/14/2020	3:00:00 a, m,	16.7	77	0
9/14/2020	4:00:00 a, m,	16.6	76	0

9/14/2020	5:00:00 a, m,	16.6	78	0
9/14/2020	6:00:00 a, m,	16.9	77	0
9/14/2020	7:00:00 a, m,	17.7	77	0
9/14/2020	8:00:00 a, m,	18.6	74	0
9/14/2020	9:00:00 a, m,	18.9	76	0
9/14/2020	10:00:00 a, m	19.8	76	0
9/14/2020	11:00:00 a, m	20.6	72	0
9/14/2020	12:00:00 p, m	21.2	71	0
9/14/2020	1:00:00 p, m,	20.7	78	0
9/14/2020	2:00:00 p, m,	23.8	64	0
9/14/2020	3:00:00 p, m,	24.2	63	0
9/14/2020	4:00:00 p, m,	23	62	0
9/14/2020	5:00:00 p, m,	22.7	61	0
9/14/2020	6:00:00 p, m,	20.7	67	0.2
9/14/2020	7:00:00 p, m,	19	72	0
9/14/2020	8:00:00 p, m,	19.7	68	0
9/14/2020	9:00:00 p, m,	20.2	67	0
9/14/2020	10:00:00 p, m	19.8	68	0
9/14/2020	11:00:00 p, m	19.7	67	0
9/15/2020	12:00:00 a, m	19.4	66	0
9/15/2020	1:00:00 a, m,	19.4	67	0
9/15/2020	2:00:00 a, m,	17.8	68	0
9/15/2020	3:00:00 a, m,	17.5	69	0
9/15/2020	4:00:00 a, m,	17.4	69	0
9/15/2020	5:00:00 a, m,	17.1	71	0
9/15/2020	6:00:00 a, m,	17.1	72	0
9/15/2020	7:00:00 a, m,	17.4	74	0
9/15/2020	8:00:00 a, m,	18.8	71	0
9/15/2020	9:00:00 a, m,	19.1	74	0
9/15/2020	10:00:00 a, m	19.6	73	0
9/15/2020	11:00:00 a, m	20.3	71	0
9/15/2020	12:00:00 p, m	21.1	72	0
9/15/2020	1:00:00 p, m,	21.4	73	0
9/15/2020	2:00:00 p, m,	22.3	66	0
9/15/2020	3:00:00 p, m,	24.5	60	0
9/15/2020	4:00:00 p, m,	23.9	60	0
9/15/2020	5:00:00 p, m,	23.4	61	0
9/15/2020	6:00:00 p, m,	21.9	66	0
9/15/2020	7:00:00 p, m,	20.8	69	0
9/15/2020	8:00:00 p, m,	20.2	69	0
9/15/2020	9:00:00 p, m,	19.8	72	0
9/15/2020	10:00:00 p, m	19.5	70	0
9/15/2020	11:00:00 p, m	19.3	72	0
9/16/2020	12:00:00 a, m	18.8	73	0
9/16/2020	1:00:00 a, m,	18.3	73	0
9/16/2020	2:00:00 a, m,	17.8	74	0
9/16/2020	3:00:00 a, m,	17.4	76	0

9/16/2020	4:00:00 a, m,	17.2	76	0
9/16/2020	5:00:00 a, m,	17.3	78	0
9/16/2020	6:00:00 a, m,	16.9	79	0
9/16/2020	7:00:00 a, m,	16.7	83	0
9/16/2020	8:00:00 a, m,	16.9	83	0
9/16/2020	9:00:00 a, m,	17.7	86	0
9/16/2020	10:00:00 a, m	19.1	85	0
9/16/2020	11:00:00 a, m	20.1	83	0
9/16/2020	12:00:00 p, m	20.8	81	0
9/16/2020	1:00:00 p, m,	19.1	83	2.6
9/16/2020	2:00:00 p, m,	16.7	95	4
9/16/2020	3:00:00 p, m,	17.5	96	0
9/16/2020	4:00:00 p, m,	18.2	93	0
9/16/2020	5:00:00 p, m,	17.9	91	0.2
9/16/2020	6:00:00 p, m,	17.6	92	0
9/16/2020	7:00:00 p, m,	17.3	92	0
9/16/2020	8:00:00 p, m,	16.9	93	0
9/16/2020	9:00:00 p, m,	16.7	91	0
9/16/2020	10:00:00 p, m	16.7	92	0
9/16/2020	11:00:00 p, m	16.7	91	0
9/17/2020	12:00:00 a, m	16.7	92	0
9/17/2020	1:00:00 a, m,	16.5	92	0
9/17/2020	2:00:00 a, m,	16.6	93	0
9/17/2020	3:00:00 a, m,	16.4	92	0
9/17/2020	4:00:00 a, m,	16.1	92	0
9/17/2020	5:00:00 a, m,	15.7	93	0
9/17/2020	6:00:00 a, m,	15.7	94	0
9/17/2020	7:00:00 a, m,	16.1	93	0
9/17/2020	8:00:00 a, m,	16.4	94	0
9/17/2020	9:00:00 a, m,	17.1	96	0
9/17/2020	10:00:00 a, m	17.9	96	0
9/17/2020	11:00:00 a, m	18.5	95	0
9/17/2020	12:00:00 p, m	19.3	93	0
9/17/2020	1:00:00 p, m,	19.4	93	0.4
9/17/2020	2:00:00 p, m,	18.8	97	3.6
9/17/2020	3:00:00 p, m,	19.3	95	0
9/17/2020	4:00:00 p, m,	19.6	95	0
9/17/2020	5:00:00 p, m,	20.1	95	0
9/17/2020	6:00:00 p, m,	19.2	92	0
9/17/2020	7:00:00 p, m,	18.8	94	0.4
9/17/2020	8:00:00 p, m,	17.8	94	0
9/17/2020	9:00:00 p, m,	17.6	95	0
9/17/2020	10:00:00 p, m	17.2	94	0
9/17/2020	11:00:00 p, m	17.2	94	0
9/18/2020	12:00:00 a, m	16.9	93	0
9/18/2020	1:00:00 a, m,	17.1	95	0
9/18/2020	2:00:00 a, m,	16.9	94	0

9/18/2020	3:00:00 a, m,	16.6	95	0
9/18/2020	4:00:00 a, m,	16.3	93	0
9/18/2020	5:00:00 a, m,	16.1	95	0
9/18/2020	6:00:00 a, m,	16.5	94	0
9/18/2020	7:00:00 a, m,	16.8	94	0
9/18/2020	8:00:00 a, m,	16.8	95	0
9/18/2020	9:00:00 a, m,	17.6	97	0
9/18/2020	10:00:00 a, m	18.2	96	0
9/18/2020	11:00:00 a, m	18.9	94	0
9/18/2020	12:00:00 p, m	19.5	92	0.2
9/18/2020	1:00:00 p, m,	19.9	95	0
9/18/2020	2:00:00 p, m,	20.6	91	0
9/18/2020	3:00:00 p, m,	21.8	88	0
9/18/2020	4:00:00 p, m,	22.7	80	0
9/18/2020	5:00:00 p, m,	21.6	83	0
9/18/2020	6:00:00 p, m,	20.6	88	0
9/18/2020	7:00:00 p, m,	19.9	90	0
9/18/2020	8:00:00 p, m,	19.5	85	0
9/18/2020	9:00:00 p, m,	19.2	88	0
9/18/2020	10:00:00 p, m	19	87	0
9/18/2020	11:00:00 p, m	18.7	88	0
9/19/2020	12:00:00 a, m	18.6	89	0
9/19/2020	1:00:00 a, m,	18.2	91	0
9/19/2020	2:00:00 a, m,	17.7	95	0
9/19/2020	3:00:00 a, m,	16.8	97	0
9/19/2020	4:00:00 a, m,	16.4	96	0
9/19/2020	5:00:00 a, m,	16.7	94	0
9/19/2020	6:00:00 a, m,	16.7	93	0
9/19/2020	7:00:00 a, m,	16.3	97	0
9/19/2020	8:00:00 a, m,	16.7	95	0
9/19/2020	9:00:00 a, m,	16.6	96	0
9/19/2020	10:00:00 a, m	16.4	96	0.6
9/19/2020	11:00:00 a, m	16.8	97	0.8
9/19/2020	12:00:00 p, m	17.3	98	0
9/19/2020	1:00:00 p, m,	17.3	97	0
9/19/2020	2:00:00 p, m,	18.4	97	0
9/19/2020	3:00:00 p, m,	18.6	97	0
9/19/2020	4:00:00 p, m,	19.6	94	0
9/19/2020	5:00:00 p, m,	20.1	94	0
9/19/2020	6:00:00 p, m,	19.8	92	0
9/19/2020	7:00:00 p, m,	18.4	92	0
9/19/2020	8:00:00 p, m,	17.9	92	0
9/19/2020	9:00:00 p, m,	17.7	90	0
9/19/2020	10:00:00 p, m	17.4	91	0
9/19/2020	11:00:00 p, m	17.5	90	0
9/20/2020	12:00:00 a, m	17.4	91	0
9/20/2020	1:00:00 a, m,	17.1	92	0

9/20/2020	2:00:00 a, m,	17.1	92	0
9/20/2020	3:00:00 a, m,	17	90	0
9/20/2020	4:00:00 a, m,	16.8	90	0
9/20/2020	5:00:00 a, m,	16.8	89	0
9/20/2020	6:00:00 a, m,	16.8	90	0
9/20/2020	7:00:00 a, m,	17.2	91	0
9/20/2020	8:00:00 a, m,	17.9	91	0
9/20/2020	9:00:00 a, m,	18.8	91	0
9/20/2020	10:00:00 a, m	19.5	88	0
9/20/2020	11:00:00 a, m	20.1	87	0
9/20/2020	12:00:00 p, m	20.3	85	0
9/20/2020	1:00:00 p, m,	21.5	82	0.2
9/20/2020	2:00:00 p, m,	19.7	91	1.2
9/20/2020	3:00:00 p, m,	20.1	91	0
9/20/2020	4:00:00 p, m,	20.3	91	0
9/20/2020	5:00:00 p, m,	19.9	91	0
9/20/2020	6:00:00 p, m,	19.1	87	0
9/20/2020	7:00:00 p, m,	18.7	86	0
9/20/2020	8:00:00 p, m,	18.6	84	0
9/20/2020	9:00:00 p, m,	18.4	85	0
9/20/2020	10:00:00 p, m	18.4	85	0
9/20/2020	11:00:00 p, m	18.2	86	0
9/21/2020	12:00:00 a, m	18.6	80	0
9/21/2020	1:00:00 a, m,	18.5	81	0
9/21/2020	2:00:00 a, m,	17.7	83	0
9/21/2020	3:00:00 a, m,	17	85	0
9/21/2020	4:00:00 a, m,	17.4	84	0
9/21/2020	5:00:00 a, m,	17.7	85	0
9/21/2020	6:00:00 a, m,	17.9	82	0
9/21/2020	7:00:00 a, m,	17.9	84	0
9/21/2020	8:00:00 a, m,	18.4	87	0
9/21/2020	9:00:00 a, m,	19.3	86	0
9/21/2020	10:00:00 a, m	20.1	83	0
9/21/2020	11:00:00 a, m	20.9	82	0
9/21/2020	12:00:00 p, m	22.2	77	0
9/21/2020	1:00:00 p, m,	22.1	79	0
9/21/2020	2:00:00 p, m,	19.1	87	0.2
9/21/2020	3:00:00 p, m,	20.4	85	0
9/21/2020	4:00:00 p, m,	21.3	77	0
9/21/2020	5:00:00 p, m,	22.2	73	0
9/21/2020	6:00:00 p, m,	20.4	73	0.2
9/21/2020	7:00:00 p, m,	19.3	73	0
9/21/2020	8:00:00 p, m,	18.7	70	0
9/21/2020	9:00:00 p, m,	19.3	62	0
9/21/2020	10:00:00 p, m	19.2	60	0
9/21/2020	11:00:00 p, m	18.4	62	0
9/22/2020	12:00:00 a, m	18.6	62	0

9/22/2020	1:00:00 a, m,	17.8	62	0
9/22/2020	2:00:00 a, m,	17.6	61	0
9/22/2020	3:00:00 a, m,	17.1	64	0
9/22/2020	4:00:00 a, m,	16.7	64	0
9/22/2020	5:00:00 a, m,	16.6	68	0
9/22/2020	6:00:00 a, m,	16.1	69	0
9/22/2020	7:00:00 a, m,	16.1	71	0
9/22/2020	8:00:00 a, m,	16.7	74	0
9/22/2020	9:00:00 a, m,	17.7	73	0
9/22/2020	10:00:00 a, m	19.4	75	0
9/22/2020	11:00:00 a, m	20.6	73	0
9/22/2020	12:00:00 p, m	22.9	60	0
9/22/2020	1:00:00 p, m,	23.8	59	0
9/22/2020	2:00:00 p, m,	25.1	49	0
9/22/2020	3:00:00 p, m,	24.5	55	0
9/22/2020	4:00:00 p, m,	24.1	58	0
9/22/2020	5:00:00 p, m,	23.3	64	0
9/22/2020	6:00:00 p, m,	22.1	63	0
9/22/2020	7:00:00 p, m,	20.9	63	0
9/22/2020	8:00:00 p, m,	20.5	53	0
9/22/2020	9:00:00 p, m,	20	51	0
9/22/2020	10:00:00 p, m	20.8	47	0
9/22/2020	11:00:00 p, m	20	49	0
9/23/2020	12:00:00 a, m	19.6	54	0
9/23/2020	1:00:00 a, m,	19	55	0
9/23/2020	2:00:00 a, m,	18.3	58	0
9/23/2020	3:00:00 a, m,	18.3	64	0
9/23/2020	4:00:00 a, m,	18.8	61	0
9/23/2020	5:00:00 a, m,	18.4	63	0
9/23/2020	6:00:00 a, m,	15.2	93	1.2
9/23/2020	7:00:00 a, m,	14.7	95	4.4
9/23/2020	8:00:00 a, m,	15.4	98	0.4
9/23/2020	9:00:00 a, m,	15.5	96	0
9/23/2020	10:00:00 a, m	16.4	95	0
9/23/2020	11:00:00 a, m	17.7	93	0
9/23/2020	12:00:00 p, m	17.4	94	0.2
9/23/2020	1:00:00 p, m,	19.1	88	0
9/23/2020	2:00:00 p, m,	19.6	87	0
9/23/2020	3:00:00 p, m,	20.2	87	0
9/23/2020	4:00:00 p, m,	19.3	89	0
9/23/2020	5:00:00 p, m,	19.8	88	0
9/23/2020	6:00:00 p, m,	18.4	92	0.4
9/23/2020	7:00:00 p, m,	16.4	95	0.4
9/23/2020	8:00:00 p, m,	15.9	97	0.4
9/23/2020	9:00:00 p, m,	15.9	96	0
9/23/2020	10:00:00 p, m	15.9	95	0
9/23/2020	11:00:00 p, m	16.4	94	0

9/24/2020	12:00:00 a, m	16.5	93	0
9/24/2020	1:00:00 a, m,	16.3	93	0
9/24/2020	2:00:00 a, m,	16.2	94	0
9/24/2020	3:00:00 a, m,	16.1	94	0
9/24/2020	4:00:00 a, m,	15.4	96	0.4
9/24/2020	5:00:00 a, m,	15.1	96	0.2
9/24/2020	6:00:00 a, m,	14.9	96	0.2
9/24/2020	7:00:00 a, m,	15.1	97	0
9/24/2020	8:00:00 a, m,	15.5	96	0
9/24/2020	9:00:00 a, m,	16	93	0
9/24/2020	10:00:00 a, m	16.8	96	0
9/24/2020	11:00:00 a, m	17.4	95	0
9/24/2020	12:00:00 p, m	17.9	92	0
9/24/2020	1:00:00 p, m,	18.7	93	0
9/24/2020	2:00:00 p, m,	18.4	86	0
9/24/2020	3:00:00 p, m,	18.1	88	0
9/24/2020	4:00:00 p, m,	18.1	89	0
9/24/2020	5:00:00 p, m,	17.8	85	0
9/24/2020	6:00:00 p, m,	17.6	83	0
9/24/2020	7:00:00 p, m,	17.3	81	0
9/24/2020	8:00:00 p, m,	17.4	81	0
9/24/2020	9:00:00 p, m,	17.6	81	0
9/24/2020	10:00:00 p, m	17.3	82	0
9/24/2020	11:00:00 p, m	18.2	75	0
9/25/2020	12:00:00 a, m	17.7	81	0
9/25/2020	1:00:00 a, m,	17.5	81	0
9/25/2020	2:00:00 a, m,	17.4	81	0
9/25/2020	3:00:00 a, m,	17.1	81	0
9/25/2020	4:00:00 a, m,	16.6	84	0
9/25/2020	5:00:00 a, m,	16.5	82	0
9/25/2020	6:00:00 a, m,	17.2	79	0
9/25/2020	7:00:00 a, m,	17.4	78	0
9/25/2020	8:00:00 a, m,	17.8	80	0
9/25/2020	9:00:00 a, m,	18.4	81	0
9/25/2020	10:00:00 a, m	18.8	84	0
9/25/2020	11:00:00 a, m	19.7	81	0
9/25/2020	12:00:00 p, m	20.9	79	0
9/25/2020	1:00:00 p, m,	21.7	77	0
9/25/2020	2:00:00 p, m,	21	78	0
9/25/2020	3:00:00 p, m,	20.2	74	0
9/25/2020	4:00:00 p, m,	19.7	69	0
9/25/2020	5:00:00 p, m,	19.9	72	0
9/25/2020	6:00:00 p, m,	19.3	69	0
9/25/2020	7:00:00 p, m,	18.6	72	0
9/25/2020	8:00:00 p, m,	18.6	70	0
9/25/2020	9:00:00 p, m,	17.7	73	0
9/25/2020	10:00:00 p, m	17.7	74	0

9/25/2020	11:00:00 p, m	17.7	75	0
9/26/2020	12:00:00 a, m	17.8	72	0
9/26/2020	1:00:00 a, m,	17.7	74	0
9/26/2020	2:00:00 a, m,	17.8	78	0
9/26/2020	3:00:00 a, m,	16.6	82	0
9/26/2020	4:00:00 a, m,	17.2	80	0
9/26/2020	5:00:00 a, m,	17.3	80	0
9/26/2020	6:00:00 a, m,	17.1	80	0
9/26/2020	7:00:00 a, m,	17.1	83	0
9/26/2020	8:00:00 a, m,	17.6	82	0
9/26/2020	9:00:00 a, m,	18.3	80	0
9/26/2020	10:00:00 a, m	18.8	82	0
9/26/2020	11:00:00 a, m	19.3	80	0
9/26/2020	12:00:00 p, m	19.4	81	0
9/26/2020	1:00:00 p, m,	19.2	82	0
9/26/2020	2:00:00 p, m,	18.4	93	0.6
9/26/2020	3:00:00 p, m,	20.2	81	0
9/26/2020	4:00:00 p, m,	20	84	0
9/26/2020	5:00:00 p, m,	20.1	84	0
9/26/2020	6:00:00 p, m,	19.7	81	0
9/26/2020	7:00:00 p, m,	17.2	90	0
9/26/2020	8:00:00 p, m,	16.9	88	0
9/26/2020	9:00:00 p, m,	16.9	87	0
9/26/2020	10:00:00 p, m	16.9	83	0
9/26/2020	11:00:00 p, m	16.8	83	0
9/27/2020	12:00:00 a, m	16.7	84	0
9/27/2020	1:00:00 a, m,	17.1	85	0
9/27/2020	2:00:00 a, m,	17	86	0
9/27/2020	3:00:00 a, m,	16.9	86	0
9/27/2020	4:00:00 a, m,	16.7	87	0
9/27/2020	5:00:00 a, m,	16.7	88	0
9/27/2020	6:00:00 a, m,	16.4	92	0
9/27/2020	7:00:00 a, m,	16.4	92	0
9/27/2020	8:00:00 a, m,	16.7	94	0
9/27/2020	9:00:00 a, m,	17.6	94	0
9/27/2020	10:00:00 a, m	18.2	91	0
9/27/2020	11:00:00 a, m	18.8	93	0
9/27/2020	12:00:00 p, m	18.2	96	3.2
9/27/2020	1:00:00 p, m,	17.7	97	3.6
9/27/2020	2:00:00 p, m,	19.3	97	0
9/27/2020	3:00:00 p, m,	18.1	96	6
9/27/2020	4:00:00 p, m,	17.7	96	0
9/27/2020	5:00:00 p, m,	17.4	96	0.2
9/27/2020	6:00:00 p, m,	17.2	94	0
9/27/2020	7:00:00 p, m,	16.9	94	0
9/27/2020	8:00:00 p, m,	16.6	94	0
9/27/2020	9:00:00 p, m,	16.6	94	0

9/27/2020 10:00:00 p, m	16.6	93	0
9/27/2020 11:00:00 p, m	16.6	93	0
9/28/2020 12:00:00 a, m	16.5	93	0
9/28/2020 1:00:00 a, m,	16.2	93	0
9/28/2020 2:00:00 a, m,	16.2	93	0
9/28/2020 3:00:00 a, m,	16.2	93	0
9/28/2020 4:00:00 a, m,	16.3	93	0
9/28/2020 5:00:00 a, m,	16.2	93	0
9/28/2020 6:00:00 a, m,	16.1	93	0
9/28/2020 7:00:00 a, m,	16.2	94	0
9/28/2020 8:00:00 a, m,	16.5	95	0
9/28/2020 9:00:00 a, m,	17.2	92	0
9/28/2020 10:00:00 a, m	18.3	95	0
9/28/2020 11:00:00 a, m	18.8	94	0
9/28/2020 12:00:00 p, m	19.3	90	0
9/28/2020 1:00:00 p, m,	20	91	0
9/28/2020 2:00:00 p, m,	21.1	84	0
9/28/2020 3:00:00 p, m,	20.9	80	0
9/28/2020 4:00:00 p, m,	21.7	79	0
9/28/2020 5:00:00 p, m,	20.8	77	0
9/28/2020 6:00:00 p, m,	19.5	81	0
9/28/2020 7:00:00 p, m,	18.3	83	0
9/28/2020 8:00:00 p, m,	18.3	83	0
9/28/2020 9:00:00 p, m,	18.1	85	0
9/28/2020 10:00:00 p, m	17.8	83	0
9/28/2020 11:00:00 p, m	17.9	76	0
9/29/2020 12:00:00 a, m	18.4	76	0
9/29/2020 1:00:00 a, m,	18.1	78	0
9/29/2020 2:00:00 a, m,	17.6	80	0
9/29/2020 3:00:00 a, m,	17.1	83	0
9/29/2020 4:00:00 a, m,	16.6	83	0
9/29/2020 5:00:00 a, m,	16.8	81	0
9/29/2020 6:00:00 a, m,	16.8	81	0
9/29/2020 7:00:00 a, m,	17.6	83	0
9/29/2020 8:00:00 a, m,	18.3	83	0
9/29/2020 9:00:00 a, m,	18.7	82	0
9/29/2020 10:00:00 a, m	19.4	83	0
9/29/2020 11:00:00 a, m	20.4	80	0
9/29/2020 12:00:00 p, m	21.3	78	0
9/29/2020 1:00:00 p, m,	20.1	83	0
9/29/2020 2:00:00 p, m,	21.2	77	0
9/29/2020 3:00:00 p, m,	21.2	71	0
9/29/2020 4:00:00 p, m,	20.6	73	0
9/29/2020 5:00:00 p, m,	20.2	69	0
9/29/2020 6:00:00 p, m,	19.7	72	0
9/29/2020 7:00:00 p, m,	19.3	71	0
9/29/2020 8:00:00 p, m,	19.1	75	0

9/29/2020	9:00:00 p, m,	18.9	75	0
9/29/2020	10:00:00 p, m	18.9	75	0
9/29/2020	11:00:00 p, m	18.9	76	0
9/30/2020	12:00:00 a, m	18.4	77	0
9/30/2020	1:00:00 a, m,	18.7	76	0
9/30/2020	2:00:00 a, m,	18.3	78	0
9/30/2020	3:00:00 a, m,	18.1	79	0
9/30/2020	4:00:00 a, m,	17.9	80	0
9/30/2020	5:00:00 a, m,	17.7	82	0
9/30/2020	6:00:00 a, m,	16.7	88	0
9/30/2020	7:00:00 a, m,	15.7	94	0.4
9/30/2020	8:00:00 a, m,	15.9	95	0.2
9/30/2020	9:00:00 a, m,	16.3	95	0
9/30/2020	10:00:00 a, m	16.9	94	0
9/30/2020	11:00:00 a, m	17.4	94	0
9/30/2020	12:00:00 p, m	17.8	93	0
9/30/2020	1:00:00 p, m,	18.2	93	0
9/30/2020	2:00:00 p, m,	18.1	96	0.8
9/30/2020	3:00:00 p, m,	18.3	95	0
9/30/2020	4:00:00 p, m,	18.4	94	0
9/30/2020	5:00:00 p, m,	17.7	96	0
9/30/2020	6:00:00 p, m,	17.4	95	0
9/30/2020	7:00:00 p, m,	16.7	94	0
9/30/2020	8:00:00 p, m,	16.2	92	0
9/30/2020	9:00:00 p, m,	16.3	89	0
9/30/2020	10:00:00 p, m	16	88	0
9/30/2020	11:00:00 p, m	16.2	86	0
Sep_20		18.6576389	80.6625	45.8
10/1/2020	12:00:00 a, m	16.1	85	0
10/1/2020	1:00:00 a, m,	16	84	0
10/1/2020	2:00:00 a, m,	16.3	81	0
10/1/2020	3:00:00 a, m,	16.4	82	0
10/1/2020	4:00:00 a, m,	16.5	83	0
10/1/2020	5:00:00 a, m,	16.5	81	0
10/1/2020	6:00:00 a, m,	16.3	82	0
10/1/2020	7:00:00 a, m,	16.5	84	0
10/1/2020	8:00:00 a, m,	17.5	83	0
10/1/2020	9:00:00 a, m,	17.9	85	0
10/1/2020	10:00:00 a, m	18.9	81	0
10/1/2020	11:00:00 a, m	19.8	84	0
10/1/2020	12:00:00 p, m	20.3	81	0
10/1/2020	1:00:00 p, m,	20.2	80	0
10/1/2020	2:00:00 p, m,	20.5	84	0.2
10/1/2020	3:00:00 p, m,	21.5	76	0
10/1/2020	4:00:00 p, m,	22.4	60	0
10/1/2020	5:00:00 p, m,	21.1	66	0.2
10/1/2020	6:00:00 p, m,	20.2	65	0

10/1/2020	7:00:00 p, m,	19.7	64	0
10/1/2020	8:00:00 p, m,	18.9	64	0
10/1/2020	9:00:00 p, m,	19.3	61	0
10/1/2020	10:00:00 p, m	19.1	60	0
10/1/2020	11:00:00 p, m	19.3	60	0
10/2/2020	12:00:00 a, m	19.1	62	0
10/2/2020	1:00:00 a, m,	18.4	63	0
10/2/2020	2:00:00 a, m,	18	67	0
10/2/2020	3:00:00 a, m,	17.7	65	0
10/2/2020	4:00:00 a, m,	17.5	68	0
10/2/2020	5:00:00 a, m,	16.3	71	0
10/2/2020	6:00:00 a, m,	16	72	0
10/2/2020	7:00:00 a, m,	16.3	76	0
10/2/2020	8:00:00 a, m,	16.8	76	0
10/2/2020	9:00:00 a, m,	17.6	76	0
10/2/2020	10:00:00 a, m	18.7	77	0
10/2/2020	11:00:00 a, m	19.9	76	0
10/2/2020	12:00:00 p, m	20.4	76	0
10/2/2020	1:00:00 p, m,	21.8	72	0
10/2/2020	2:00:00 p, m,	22.9	65	0
10/2/2020	3:00:00 p, m,	22.4	68	0
10/2/2020	4:00:00 p, m,	21.7	70	0
10/2/2020	5:00:00 p, m,	21.3	69	0
10/2/2020	6:00:00 p, m,	20.8	68	0
10/2/2020	7:00:00 p, m,	20.2	68	0
10/2/2020	8:00:00 p, m,	19.7	67	0
10/2/2020	9:00:00 p, m,	20.2	63	0
10/2/2020	10:00:00 p, m	19.3	67	0
10/2/2020	11:00:00 p, m	19.3	69	0
10/3/2020	12:00:00 a, m	19.4	69	0
10/3/2020	1:00:00 a, m,	19.3	73	0
10/3/2020	2:00:00 a, m,	17.6	84	0
10/3/2020	3:00:00 a, m,	17.1	85	0
10/3/2020	4:00:00 a, m,	17.3	82	0
10/3/2020	5:00:00 a, m,	17.3	83	0
10/3/2020	6:00:00 a, m,	17.5	83	0
10/3/2020	7:00:00 a, m,	17.6	84	0
10/3/2020	8:00:00 a, m,	17.8	85	0
10/3/2020	9:00:00 a, m,	18.1	85	0
10/3/2020	10:00:00 a, m	18.4	85	0
10/3/2020	11:00:00 a, m	18.4	92	0.6
10/3/2020	12:00:00 p, m	18.7	93	0
10/3/2020	1:00:00 p, m,	18.8	90	0.4
10/3/2020	2:00:00 p, m,	18.2	96	1
10/3/2020	3:00:00 p, m,	18.8	94	0
10/3/2020	4:00:00 p, m,	18.7	94	0
10/3/2020	5:00:00 p, m,	19.2	93	0

10/3/2020	6:00:00 p, m,	18.5	90	0
10/3/2020	7:00:00 p, m,	17.7	86	0
10/3/2020	8:00:00 p, m,	16.4	91	0.2
10/3/2020	9:00:00 p, m,	16.3	94	0.2
10/3/2020	10:00:00 p, m	16.4	91	0
10/3/2020	11:00:00 p, m	17	87	0
10/4/2020	12:00:00 a, m	17.5	84	0
10/4/2020	1:00:00 a, m,	17.2	88	0
10/4/2020	2:00:00 a, m,	17.1	87	0
10/4/2020	3:00:00 a, m,	16.4	90	0
10/4/2020	4:00:00 a, m,	16.8	89	0
10/4/2020	5:00:00 a, m,	16.6	89	0
10/4/2020	6:00:00 a, m,	16.3	90	0
10/4/2020	7:00:00 a, m,	16.1	92	0
10/4/2020	8:00:00 a, m,	15.6	95	0.6
10/4/2020	9:00:00 a, m,	15.8	96	0.6
10/4/2020	10:00:00 a, m	16.3	97	0
10/4/2020	11:00:00 a, m	16.4	95	0
10/4/2020	12:00:00 p, m	16.9	96	0
10/4/2020	1:00:00 p, m,	17.1	97	1
10/4/2020	2:00:00 p, m,	17.2	96	0.2
10/4/2020	3:00:00 p, m,	17.7	98	0
10/4/2020	4:00:00 p, m,	17.9	97	0
10/4/2020	5:00:00 p, m,	17.5	96	0.2
10/4/2020	6:00:00 p, m,	17	95	0
10/4/2020	7:00:00 p, m,	16	94	0
10/4/2020	8:00:00 p, m,	16.2	95	0
10/4/2020	9:00:00 p, m,	16.1	94	0
10/4/2020	10:00:00 p, m	16.2	93	0
10/4/2020	11:00:00 p, m	16.2	94	0
10/5/2020	12:00:00 a, m	15.6	95	0.4
10/5/2020	1:00:00 a, m,	15.3	96	0
10/5/2020	2:00:00 a, m,	15.4	97	0
10/5/2020	3:00:00 a, m,	15.4	96	0
10/5/2020	4:00:00 a, m,	15.4	95	0.2
10/5/2020	5:00:00 a, m,	14.9	96	0.2
10/5/2020	6:00:00 a, m,	15.2	95	0
10/5/2020	7:00:00 a, m,	15.7	95	0
10/5/2020	8:00:00 a, m,	15.9	95	0
10/5/2020	9:00:00 a, m,	16.7	95	0
10/5/2020	10:00:00 a, m	17.6	95	0
10/5/2020	11:00:00 a, m	18.3	92	0
10/5/2020	12:00:00 p, m	18.8	91	0
10/5/2020	1:00:00 p, m,	19.6	89	0
10/5/2020	2:00:00 p, m,	20.3	81	0
10/5/2020	3:00:00 p, m,	20.9	80	0
10/5/2020	4:00:00 p, m,	21.1	83	0

10/5/2020	5:00:00 p, m,	19.2	82	0
10/5/2020	6:00:00 p, m,	18.3	80	0
10/5/2020	7:00:00 p, m,	17.8	80	0
10/5/2020	8:00:00 p, m,	17.7	79	0
10/5/2020	9:00:00 p, m,	17.2	79	0
10/5/2020	10:00:00 p, m	17	81	0
10/5/2020	11:00:00 p, m	17.1	79	0
10/6/2020	12:00:00 a, m	17	79	0
10/6/2020	1:00:00 a, m,	16.6	81	0
10/6/2020	2:00:00 a, m,	16.6	81	0
10/6/2020	3:00:00 a, m,	16.3	81	0
10/6/2020	4:00:00 a, m,	16.5	80	0
10/6/2020	5:00:00 a, m,	16.8	79	0
10/6/2020	6:00:00 a, m,	16.9	79	0
10/6/2020	7:00:00 a, m,	17.5	79	0
10/6/2020	8:00:00 a, m,	17.8	81	0
10/6/2020	9:00:00 a, m,	18.2	82	0
10/6/2020	10:00:00 a, m	19.2	81	0
10/6/2020	11:00:00 a, m	20.1	81	0
10/6/2020	12:00:00 p, m	20.9	76	0
10/6/2020	1:00:00 p, m,	22	74	0
10/6/2020	2:00:00 p, m,	22.2	74	0
10/6/2020	3:00:00 p, m,	23.2	69	0
10/6/2020	4:00:00 p, m,	22.2	68	0
10/6/2020	5:00:00 p, m,	21.2	72	0
10/6/2020	6:00:00 p, m,	20.9	68	0
10/6/2020	7:00:00 p, m,	20.1	69	0
10/6/2020	8:00:00 p, m,	20	68	0
10/6/2020	9:00:00 p, m,	20	66	0
10/6/2020	10:00:00 p, m	19.6	66	0
10/6/2020	11:00:00 p, m	19	71	0
10/7/2020	12:00:00 a, m	18.4	71	0
10/7/2020	1:00:00 a, m,	17.6	76	0
10/7/2020	2:00:00 a, m,	17.4	74	0
10/7/2020	3:00:00 a, m,	18.1	72	0
10/7/2020	4:00:00 a, m,	18	71	0
10/7/2020	5:00:00 a, m,	18.1	71	0
10/7/2020	6:00:00 a, m,	17.7	75	0
10/7/2020	7:00:00 a, m,	18.1	76	0
10/7/2020	8:00:00 a, m,	18.6	77	0
10/7/2020	9:00:00 a, m,	18.8	77	0
10/7/2020	10:00:00 a, m	19.2	77	0
10/7/2020	11:00:00 a, m	19.2	82	0
10/7/2020	12:00:00 p, m	16.5	92	1.2
10/7/2020	1:00:00 p, m,	16.4	95	0.6
10/7/2020	2:00:00 p, m,	16.7	95	0.2
10/7/2020	3:00:00 p, m,	17.8	94	0.2

10/7/2020	4:00:00 p, m,	17.8	95	0
10/7/2020	5:00:00 p, m,	17.6	93	0
10/7/2020	6:00:00 p, m,	17.2	89	0
10/7/2020	7:00:00 p, m,	16.6	90	0
10/7/2020	8:00:00 p, m,	16.7	85	0
10/7/2020	9:00:00 p, m,	17.2	84	0
10/7/2020	10:00:00 p, m	17.3	83	0
10/7/2020	11:00:00 p, m	17.6	81	0
10/8/2020	12:00:00 a, m	17.3	82	0
10/8/2020	1:00:00 a, m,	16.9	82	0
10/8/2020	2:00:00 a, m,	17.2	80	0
10/8/2020	3:00:00 a, m,	17.7	78	0
10/8/2020	4:00:00 a, m,	17.4	81	0
10/8/2020	5:00:00 a, m,	17.6	80	0
10/8/2020	6:00:00 a, m,	17.5	80	0
10/8/2020	7:00:00 a, m,	17.7	82	0
10/8/2020	8:00:00 a, m,	17.9	82	0
10/8/2020	9:00:00 a, m,	18.3	83	0
10/8/2020	10:00:00 a, m	19.1	84	0
10/8/2020	11:00:00 a, m	19.2	88	0.2
10/8/2020	12:00:00 p, m	19.3	87	0
10/8/2020	1:00:00 p, m,	18.9	93	0.2
10/8/2020	2:00:00 p, m,	18.5	92	0
10/8/2020	3:00:00 p, m,	18.7	88	0
10/8/2020	4:00:00 p, m,	18.5	90	0
10/8/2020	5:00:00 p, m,	17.3	89	0.2
10/8/2020	6:00:00 p, m,	16.9	89	0
10/8/2020	7:00:00 p, m,	16.8	85	0
10/8/2020	8:00:00 p, m,	17.1	83	0
10/8/2020	9:00:00 p, m,	17.6	79	0
10/8/2020	10:00:00 p, m	17.7	79	0
10/8/2020	11:00:00 p, m	17.2	80	0
10/9/2020	12:00:00 a, m	17.1	81	0
10/9/2020	1:00:00 a, m,	17.1	80	0
10/9/2020	2:00:00 a, m,	17.1	80	0
10/9/2020	3:00:00 a, m,	17.1	79	0
10/9/2020	4:00:00 a, m,	17.3	78	0
10/9/2020	5:00:00 a, m,	17.2	77	0
10/9/2020	6:00:00 a, m,	17.4	76	0
10/9/2020	7:00:00 a, m,	17.4	80	0
10/9/2020	8:00:00 a, m,	17.3	80	0
10/9/2020	9:00:00 a, m,	18.2	80	0
10/9/2020	10:00:00 a, m	19.4	77	0
10/9/2020	11:00:00 a, m	20.3	76	0
10/9/2020	12:00:00 p, m	20.2	77	0
10/9/2020	1:00:00 p, m,	20.6	75	0
10/9/2020	2:00:00 p, m,	22.2	71	0

10/9/2020	3:00:00 p, m,	20.8	77	0.2
10/9/2020	4:00:00 p, m,	21.4	73	0
10/9/2020	5:00:00 p, m,	21.2	69	0
10/9/2020	6:00:00 p, m,	20.1	69	0
10/9/2020	7:00:00 p, m,	19.5	69	0
10/9/2020	8:00:00 p, m,	19.8	66	0
10/9/2020	9:00:00 p, m,	19.5	65	0
10/9/2020	10:00:00 p, m	18.4	69	0
10/9/2020	11:00:00 p, m	18.3	68	0
10/10/2020	12:00:00 a, m	18.1	69	0
10/10/2020	1:00:00 a, m,	17.8	69	0
10/10/2020	2:00:00 a, m,	18.2	66	0
10/10/2020	3:00:00 a, m,	18.8	64	0
10/10/2020	4:00:00 a, m,	18.6	66	0
10/10/2020	5:00:00 a, m,	18.4	69	0
10/10/2020	6:00:00 a, m,	18.3	70	0
10/10/2020	7:00:00 a, m,	18.4	70	0
10/10/2020	8:00:00 a, m,	18.5	74	0
10/10/2020	9:00:00 a, m,	18.7	75	0
10/10/2020	10:00:00 a, m	19.5	74	0
10/10/2020	11:00:00 a, m	20.2	74	0
10/10/2020	12:00:00 p, m	21.2	70	0
10/10/2020	1:00:00 p, m,	22.4	65	0
10/10/2020	2:00:00 p, m,	22.4	69	0
10/10/2020	3:00:00 p, m,	21.8	69	0
10/10/2020	4:00:00 p, m,	21.1	69	0
10/10/2020	5:00:00 p, m,	21.6	67	0
10/10/2020	6:00:00 p, m,	20.8	66	0
10/10/2020	7:00:00 p, m,	19.9	67	0
10/10/2020	8:00:00 p, m,	20	68	0
10/10/2020	9:00:00 p, m,	19.8	67	0
10/10/2020	10:00:00 p, m	20	66	0
10/10/2020	11:00:00 p, m	19.6	69	0
10/11/2020	12:00:00 a, m	19.4	66	0
10/11/2020	1:00:00 a, m,	19.4	65	0
10/11/2020	2:00:00 a, m,	18.6	70	0
10/11/2020	3:00:00 a, m,	17.8	72	0
10/11/2020	4:00:00 a, m,	18.3	69	0
10/11/2020	5:00:00 a, m,	17.7	73	0
10/11/2020	6:00:00 a, m,	18	73	0
10/11/2020	7:00:00 a, m,	18.6	74	0
10/11/2020	8:00:00 a, m,	19.3	74	0
10/11/2020	9:00:00 a, m,	20.2	73	0
10/11/2020	10:00:00 a, m	20.3	73	0
10/11/2020	11:00:00 a, m	20.9	72	0
10/11/2020	12:00:00 p, m	21.8	70	0
10/11/2020	1:00:00 p, m,	22.9	62	0

10/11/2020	2:00:00 p, m,	23.9	62	0
10/11/2020	3:00:00 p, m,	22.8	62	0
10/11/2020	4:00:00 p, m,	22.1	65	0
10/11/2020	5:00:00 p, m,	21.7	66	0
10/11/2020	6:00:00 p, m,	20.4	66	0
10/11/2020	7:00:00 p, m,	20.1	66	0
10/11/2020	8:00:00 p, m,	19.8	68	0
10/11/2020	9:00:00 p, m,	20.1	63	0
10/11/2020	10:00:00 p, m	19.8	66	0
10/11/2020	11:00:00 p, m	20.3	63	0
10/12/2020	12:00:00 a, m	19.3	67	0
10/12/2020	1:00:00 a, m,	18.3	70	0
10/12/2020	2:00:00 a, m,	18.2	69	0
10/12/2020	3:00:00 a, m,	18.4	70	0
10/12/2020	4:00:00 a, m,	18.6	69	0
10/12/2020	5:00:00 a, m,	18.4	69	0
10/12/2020	6:00:00 a, m,	18	71	0
10/12/2020	7:00:00 a, m,	18.6	72	0
10/12/2020	8:00:00 a, m,	19	74	0
10/12/2020	9:00:00 a, m,	19.2	74	0
10/12/2020	10:00:00 a, m	20.2	72	0
10/12/2020	11:00:00 a, m	21.1	72	0
10/12/2020	12:00:00 p, m	21.8	68	0
10/12/2020	1:00:00 p, m,	24.2	57	0
10/12/2020	2:00:00 p, m,	25.2	53	0
10/12/2020	3:00:00 p, m,	24	60	0
10/12/2020	4:00:00 p, m,	22.7	65	0
10/12/2020	5:00:00 p, m,	22.2	64	0
10/12/2020	6:00:00 p, m,	21.2	64	0
10/12/2020	7:00:00 p, m,	17.4	87	0.4
10/12/2020	8:00:00 p, m,	16.4	87	0
10/12/2020	9:00:00 p, m,	17.3	85	0
10/12/2020	10:00:00 p, m	17.7	74	0
10/12/2020	11:00:00 p, m	18.5	72	0
10/13/2020	12:00:00 a, m	18.7	71	0
10/13/2020	1:00:00 a, m,	18.2	73	0
10/13/2020	2:00:00 a, m,	18.2	75	0
10/13/2020	3:00:00 a, m,	18.6	74	0
10/13/2020	4:00:00 a, m,	18.2	75	0
10/13/2020	5:00:00 a, m,	17.9	73	0
10/13/2020	6:00:00 a, m,	18.6	71	0
10/13/2020	7:00:00 a, m,	18.1	74	0
10/13/2020	8:00:00 a, m,	18.3	74	0
10/13/2020	9:00:00 a, m,	18.4	78	0
10/13/2020	10:00:00 a, m	18.6	77	0
10/13/2020	11:00:00 a, m	18.9	78	0.2
10/13/2020	12:00:00 p, m	19.2	78	0

10/13/2020	1:00:00 p, m,	19.6	78	0
10/13/2020	2:00:00 p, m,	20.1	77	0
10/13/2020	3:00:00 p, m,	20.6	70	0
10/13/2020	4:00:00 p, m,	20.9	73	0
10/13/2020	5:00:00 p, m,	20.1	75	0
10/13/2020	6:00:00 p, m,	19.3	78	0
10/13/2020	7:00:00 p, m,	18.6	76	0
10/13/2020	8:00:00 p, m,	18.4	74	0
10/13/2020	9:00:00 p, m,	18.5	73	0
10/13/2020	10:00:00 p, m	18.4	73	0
10/13/2020	11:00:00 p, m	18.4	72	0
10/14/2020	12:00:00 a, m	18.2	74	0
10/14/2020	1:00:00 a, m,	17.8	74	0
10/14/2020	2:00:00 a, m,	17.9	73	0
10/14/2020	3:00:00 a, m,	17.4	73	0
10/14/2020	4:00:00 a, m,	17.3	74	0
10/14/2020	5:00:00 a, m,	17.9	71	0
10/14/2020	6:00:00 a, m,	17.7	72	0
10/14/2020	7:00:00 a, m,	18.1	73	0
10/14/2020	8:00:00 a, m,	18.4	74	0
10/14/2020	9:00:00 a, m,	19	77	0
10/14/2020	10:00:00 a, m	19.6	72	0
10/14/2020	11:00:00 a, m	20.4	74	0
10/14/2020	12:00:00 p, m	21.2	72	0
10/14/2020	1:00:00 p, m,	21.2	72	0
10/14/2020	2:00:00 p, m,	23.3	61	0
10/14/2020	3:00:00 p, m,	22.2	66	0
10/14/2020	4:00:00 p, m,	21.1	71	0
10/14/2020	5:00:00 p, m,	21.8	67	0
10/14/2020	6:00:00 p, m,	20.7	71	0
10/14/2020	7:00:00 p, m,	19.2	74	0
10/14/2020	8:00:00 p, m,	19.3	70	0
10/14/2020	9:00:00 p, m,	19.4	69	0
10/14/2020	10:00:00 p, m	20.2	67	0
10/14/2020	11:00:00 p, m	20.1	68	0
10/15/2020	12:00:00 a, m	19.9	69	0
10/15/2020	1:00:00 a, m,	19.7	70	0
10/15/2020	2:00:00 a, m,	19.1	73	0
10/15/2020	3:00:00 a, m,	18.8	75	0
10/15/2020	4:00:00 a, m,	18.6	76	0
10/15/2020	5:00:00 a, m,	17.4	78	0
10/15/2020	6:00:00 a, m,	16.8	80	0
10/15/2020	7:00:00 a, m,	16.9	81	0
10/15/2020	8:00:00 a, m,	17.6	80	0
10/15/2020	9:00:00 a, m,	18	84	0
10/15/2020	10:00:00 a, m	19.4	80	0
10/15/2020	11:00:00 a, m	20.8	77	0

10/15/2020	12:00:00 p, m	22.3	73	0
10/15/2020	1:00:00 p, m,	22.4	68	0
10/15/2020	2:00:00 p, m,	22.3	70	0
10/15/2020	3:00:00 p, m,	22.4	67	0
10/15/2020	4:00:00 p, m,	22.5	70	0
10/15/2020	5:00:00 p, m,	21.8	64	0
10/15/2020	6:00:00 p, m,	21.7	66	0
10/15/2020	7:00:00 p, m,	21.2	67	0
10/15/2020	8:00:00 p, m,	21.1	62	0
10/15/2020	9:00:00 p, m,	20.4	65	0
10/15/2020	10:00:00 p, m	20.1	66	0
10/15/2020	11:00:00 p, m	19.9	67	0
10/16/2020	12:00:00 a, m	19.9	67	0
10/16/2020	1:00:00 a, m,	20	68	0
10/16/2020	2:00:00 a, m,	20	69	0
10/16/2020	3:00:00 a, m,	19.6	71	0
10/16/2020	4:00:00 a, m,	19.2	73	0
10/16/2020	5:00:00 a, m,	19.1	72	0
10/16/2020	6:00:00 a, m,	19.2	72	0
10/16/2020	7:00:00 a, m,	19	77	0
10/16/2020	8:00:00 a, m,	19.2	77	0
10/16/2020	9:00:00 a, m,	18.9	82	0
10/16/2020	10:00:00 a, m	19.1	82	0
10/16/2020	11:00:00 a, m	18.4	91	0.2
10/16/2020	12:00:00 p, m	18.7	87	0
10/16/2020	1:00:00 p, m,	19.3	85	0
10/16/2020	2:00:00 p, m,	20.3	81	0
10/16/2020	3:00:00 p, m,	20.8	77	0
10/16/2020	4:00:00 p, m,	20.9	79	0
10/16/2020	5:00:00 p, m,	20.8	78	0
10/16/2020	6:00:00 p, m,	20.2	72	0
10/16/2020	7:00:00 p, m,	19.9	71	0
10/16/2020	8:00:00 p, m,	20.1	72	0
10/16/2020	9:00:00 p, m,	20	71	0
10/16/2020	10:00:00 p, m	19.3	72	0
10/16/2020	11:00:00 p, m	18.2	74	0
10/17/2020	12:00:00 a, m	19.2	68	0
10/17/2020	1:00:00 a, m,	19.4	68	0
10/17/2020	2:00:00 a, m,	18.9	69	0
10/17/2020	3:00:00 a, m,	18.8	69	0
10/17/2020	4:00:00 a, m,	19.2	61	0
10/17/2020	5:00:00 a, m,	18.9	65	0
10/17/2020	6:00:00 a, m,	19	67	0
10/17/2020	7:00:00 a, m,	18.6	73	0
10/17/2020	8:00:00 a, m,	18.6	75	0
10/17/2020	9:00:00 a, m,	19.1	74	0
10/17/2020	10:00:00 a, m	20.1	72	0

10/17/2020	11:00:00 a, m	20.7	71	0
10/17/2020	12:00:00 p, m	22.1	68	0
10/17/2020	1:00:00 p, m,	22.1	71	0
10/17/2020	2:00:00 p, m,	22.4	67	0
10/17/2020	3:00:00 p, m,	23.6	59	0
10/17/2020	4:00:00 p, m,	22.6	66	0
10/17/2020	5:00:00 p, m,	21.4	73	0.4
10/17/2020	6:00:00 p, m,	20.1	72	0
10/17/2020	7:00:00 p, m,	19.8	70	0
10/17/2020	8:00:00 p, m,	19.9	68	0
10/17/2020	9:00:00 p, m,	20.2	69	0
10/17/2020	10:00:00 p, m	19.8	69	0
10/17/2020	11:00:00 p, m	19.2	70	0
10/18/2020	12:00:00 a, m	18.8	71	0
10/18/2020	1:00:00 a, m,	19.1	71	0
10/18/2020	2:00:00 a, m,	18.8	73	0
10/18/2020	3:00:00 a, m,	18.7	73	0
10/18/2020	4:00:00 a, m,	18.3	73	0
10/18/2020	5:00:00 a, m,	17.7	75	0
10/18/2020	6:00:00 a, m,	17.9	76	0
10/18/2020	7:00:00 a, m,	18.3	73	0
10/18/2020	8:00:00 a, m,	19.2	74	0
10/18/2020	9:00:00 a, m,	19.5	76	0
10/18/2020	10:00:00 a, m	19.9	79	0
10/18/2020	11:00:00 a, m	21.3	70	0
10/18/2020	12:00:00 p, m	22.7	67	0
10/18/2020	1:00:00 p, m,	22.4	65	0
10/18/2020	2:00:00 p, m,	22.7	66	0
10/18/2020	3:00:00 p, m,	23.9	59	0
10/18/2020	4:00:00 p, m,	23.9	58	0
10/18/2020	5:00:00 p, m,	22.5	65	0
10/18/2020	6:00:00 p, m,	20.6	69	0
10/18/2020	7:00:00 p, m,	20.3	67	0
10/18/2020	8:00:00 p, m,	19.6	67	0
10/18/2020	9:00:00 p, m,	19.7	59	0
10/18/2020	10:00:00 p, m	19.5	56	0
10/18/2020	11:00:00 p, m	19.4	56	0
10/19/2020	12:00:00 a, m	19.6	52	0
10/19/2020	1:00:00 a, m,	18.9	56	0
10/19/2020	2:00:00 a, m,	18.1	62	0
10/19/2020	3:00:00 a, m,	18.4	63	0
10/19/2020	4:00:00 a, m,	18.1	62	0
10/19/2020	5:00:00 a, m,	17.9	65	0
10/19/2020	6:00:00 a, m,	17.5	67	0
10/19/2020	7:00:00 a, m,	17.6	69	0
10/19/2020	8:00:00 a, m,	18.9	68	0
10/19/2020	9:00:00 a, m,	19.7	68	0

10/19/2020	10:00:00 a, m	20.1	67	0
10/19/2020	11:00:00 a, m	21.4	68	0
10/19/2020	12:00:00 p, m	21.7	68	0
10/19/2020	1:00:00 p, m,	22.4	65	0
10/19/2020	2:00:00 p, m,	21.8	71	0
10/19/2020	3:00:00 p, m,	24.6	56	0
10/19/2020	4:00:00 p, m,	24.3	59	0
10/19/2020	5:00:00 p, m,	23.5	60	0
10/19/2020	6:00:00 p, m,	22.4	62	0
10/19/2020	7:00:00 p, m,	21.6	65	0
10/19/2020	8:00:00 p, m,	21.3	67	0
10/19/2020	9:00:00 p, m,	20.5	68	0
10/19/2020	10:00:00 p, m	20.3	70	0
10/19/2020	11:00:00 p, m	19.7	75	0
10/20/2020	12:00:00 a, m	19.6	76	0
10/20/2020	1:00:00 a, m,	19.6	80	0
10/20/2020	2:00:00 a, m,	19.3	80	0
10/20/2020	3:00:00 a, m,	18.7	82	0
10/20/2020	4:00:00 a, m,	18.4	85	0
10/20/2020	5:00:00 a, m,	17.7	85	0
10/20/2020	6:00:00 a, m,	17.1	84	0
10/20/2020	7:00:00 a, m,	17.8	84	0
10/20/2020	8:00:00 a, m,	17.8	87	0
10/20/2020	9:00:00 a, m,	18.3	90	0
10/20/2020	10:00:00 a, m	19.8	88	0
10/20/2020	11:00:00 a, m	20.6	84	0
10/20/2020	12:00:00 p, m	21.3	79	0
10/20/2020	1:00:00 p, m,	22.7	77	0
10/20/2020	2:00:00 p, m,	22.7	72	0
10/20/2020	3:00:00 p, m,	22.9	75	0
10/20/2020	4:00:00 p, m,	22.8	73	0
10/20/2020	5:00:00 p, m,	22.9	69	0
10/20/2020	6:00:00 p, m,	21.8	68	0
10/20/2020	7:00:00 p, m,	21.6	66	0
10/20/2020	8:00:00 p, m,	21	66	0
10/20/2020	9:00:00 p, m,	20.9	70	0
10/20/2020	10:00:00 p, m	20.6	72	0
10/20/2020	11:00:00 p, m	20	77	0
10/21/2020	12:00:00 a, m	19.3	80	0
10/21/2020	1:00:00 a, m,	18.4	85	0
10/21/2020	2:00:00 a, m,	17.3	91	0.4
10/21/2020	3:00:00 a, m,	16.7	92	0
10/21/2020	4:00:00 a, m,	16.9	92	0
10/21/2020	5:00:00 a, m,	17.2	91	0
10/21/2020	6:00:00 a, m,	17.3	90	0
10/21/2020	7:00:00 a, m,	17.6	89	0
10/21/2020	8:00:00 a, m,	17.8	92	0

10/21/2020	9:00:00 a, m,	18.6	91	0
10/21/2020	10:00:00 a, m	18.9	90	0
10/21/2020	11:00:00 a, m	19.8	91	0
10/21/2020	12:00:00 p, m	20.8	86	0
10/21/2020	1:00:00 p, m,	21.7	84	0
10/21/2020	2:00:00 p, m,	22.3	73	0
10/21/2020	3:00:00 p, m,	23.5	62	0
10/21/2020	4:00:00 p, m,	23.2	68	0
10/21/2020	5:00:00 p, m,	22.3	63	0
10/21/2020	6:00:00 p, m,	21.1	66	0
10/21/2020	7:00:00 p, m,	20.3	67	0
10/21/2020	8:00:00 p, m,	20.2	67	0
10/21/2020	9:00:00 p, m,	20.3	71	0
10/21/2020	10:00:00 p, m	18.8	77	0
10/21/2020	11:00:00 p, m	18.9	76	0
10/22/2020	12:00:00 a, m	19	76	0
10/22/2020	1:00:00 a, m,	18.8	77	0
10/22/2020	2:00:00 a, m,	18.8	76	0
10/22/2020	3:00:00 a, m,	18.7	74	0
10/22/2020	4:00:00 a, m,	18.9	74	0
10/22/2020	5:00:00 a, m,	19.2	72	0
10/22/2020	6:00:00 a, m,	19.1	75	0
10/22/2020	7:00:00 a, m,	19	77	0
10/22/2020	8:00:00 a, m,	19.5	75	0
10/22/2020	9:00:00 a, m,	19.6	77	0
10/22/2020	10:00:00 a, m	20.1	77	0
10/22/2020	11:00:00 a, m	20.4	75	0
10/22/2020	12:00:00 p, m	20.9	76	0
10/22/2020	1:00:00 p, m,	21.3	76	0
10/22/2020	2:00:00 p, m,	22.2	72	0
10/22/2020	3:00:00 p, m,	22.6	68	0
10/22/2020	4:00:00 p, m,	23.1	65	0
10/22/2020	5:00:00 p, m,	22.3	66	0
10/22/2020	6:00:00 p, m,	21.4	68	0
10/22/2020	7:00:00 p, m,	20.6	68	0
10/22/2020	8:00:00 p, m,	20.3	65	0
10/22/2020	9:00:00 p, m,	19.6	68	0
10/22/2020	10:00:00 p, m	19.8	69	0
10/22/2020	11:00:00 p, m	20.3	69	0
10/23/2020	12:00:00 a, m	19.2	75	0
10/23/2020	1:00:00 a, m,	19.1	71	0
10/23/2020	2:00:00 a, m,	18.6	75	0
10/23/2020	3:00:00 a, m,	18.3	74	0
10/23/2020	4:00:00 a, m,	18.2	73	0
10/23/2020	5:00:00 a, m,	18.3	75	0
10/23/2020	6:00:00 a, m,	18.4	74	0
10/23/2020	7:00:00 a, m,	18.6	74	0

10/23/2020	8:00:00 a, m,	18.8	76	0
10/23/2020	9:00:00 a, m,	19.3	76	0
10/23/2020	10:00:00 a, m	20.7	74	0
10/23/2020	11:00:00 a, m	21.8	71	0
10/23/2020	12:00:00 p, m	22.9	65	0
10/23/2020	1:00:00 p, m,	22.7	67	0
10/23/2020	2:00:00 p, m,	23.7	62	0
10/23/2020	3:00:00 p, m,	25.3	51	0
10/23/2020	4:00:00 p, m,	23.9	56	0
10/23/2020	5:00:00 p, m,	23.4	54	0
10/23/2020	6:00:00 p, m,	22.3	57	0
10/23/2020	7:00:00 p, m,	21.4	59	0
10/23/2020	8:00:00 p, m,	20.3	64	0
10/23/2020	9:00:00 p, m,	19.9	66	0
10/23/2020	10:00:00 p, m	19.8	63	0
10/23/2020	11:00:00 p, m	19.8	63	0
10/24/2020	12:00:00 a, m	19	67	0
10/24/2020	1:00:00 a, m,	18.5	68	0
10/24/2020	2:00:00 a, m,	18.5	69	0
10/24/2020	3:00:00 a, m,	18.1	68	0
10/24/2020	4:00:00 a, m,	17.9	69	0
10/24/2020	5:00:00 a, m,	17.8	70	0
10/24/2020	6:00:00 a, m,	17.4	72	0
10/24/2020	7:00:00 a, m,	18.1	74	0
10/24/2020	8:00:00 a, m,	18.6	72	0
10/24/2020	9:00:00 a, m,	19.3	73	0
10/24/2020	10:00:00 a, m	20.1	75	0
10/24/2020	11:00:00 a, m	21.4	66	0
10/24/2020	12:00:00 p, m	21.3	70	0
10/24/2020	1:00:00 p, m,	22.6	65	0
10/24/2020	2:00:00 p, m,	25.1	53	0
10/24/2020	3:00:00 p, m,	23.6	57	0
10/24/2020	4:00:00 p, m,	24.1	58	0
10/24/2020	5:00:00 p, m,	23.1	62	0
10/24/2020	6:00:00 p, m,	21.9	68	0
10/24/2020	7:00:00 p, m,	20.8	63	0
10/24/2020	8:00:00 p, m,	20.3	69	0
10/24/2020	9:00:00 p, m,	20.2	73	0
10/24/2020	10:00:00 p, m	19.8	74	0
10/24/2020	11:00:00 p, m	19.7	76	0
10/25/2020	12:00:00 a, m	18.8	78	0
10/25/2020	1:00:00 a, m,	18.7	79	0
10/25/2020	2:00:00 a, m,	17.4	83	0
10/25/2020	3:00:00 a, m,	17.1	86	0
10/25/2020	4:00:00 a, m,	16.8	86	0
10/25/2020	5:00:00 a, m,	16.7	87	0
10/25/2020	6:00:00 a, m,	16.8	87	0

10/25/2020	7:00:00 a, m,	16.9	88	0
10/25/2020	8:00:00 a, m,	17.2	91	0
10/25/2020	9:00:00 a, m,	18.3	92	0
10/25/2020	10:00:00 a, m	19.3	91	0
10/25/2020	11:00:00 a, m	19.3	91	0
10/25/2020	12:00:00 p, m	20.3	87	0
10/25/2020	1:00:00 p, m,	21.4	82	0
10/25/2020	2:00:00 p, m,	24	71	0
10/25/2020	3:00:00 p, m,	21.9	85	1
10/25/2020	4:00:00 p, m,	19	94	0
10/25/2020	5:00:00 p, m,	19.1	90	0.2
10/25/2020	6:00:00 p, m,	18.6	88	0
10/25/2020	7:00:00 p, m,	18.4	87	0
10/25/2020	8:00:00 p, m,	18.3	88	0
10/25/2020	9:00:00 p, m,	18.2	86	0
10/25/2020	10:00:00 p, m	18.1	87	0
10/25/2020	11:00:00 p, m	18	88	0
10/26/2020	12:00:00 a, m	18	90	0
10/26/2020	1:00:00 a, m,	17.3	90	0
10/26/2020	2:00:00 a, m,	16.9	90	0
10/26/2020	3:00:00 a, m,	16.5	89	0
10/26/2020	4:00:00 a, m,	16.1	88	0
10/26/2020	5:00:00 a, m,	16.1	89	0
10/26/2020	6:00:00 a, m,	16.4	88	0
10/26/2020	7:00:00 a, m,	16.9	88	0
10/26/2020	8:00:00 a, m,	16.9	91	0
10/26/2020	9:00:00 a, m,	17.9	93	0
10/26/2020	10:00:00 a, m	18.8	94	0
10/26/2020	11:00:00 a, m	19.2	91	0
10/26/2020	12:00:00 p, m	20.8	87	0
10/26/2020	1:00:00 p, m,	21.4	86	0
10/26/2020	2:00:00 p, m,	21.3	86	0
10/26/2020	3:00:00 p, m,	22.7	78	0
10/26/2020	4:00:00 p, m,	21.8	84	0
10/26/2020	5:00:00 p, m,	21.5	86	0.4
10/26/2020	6:00:00 p, m,	19.3	91	0.2
10/26/2020	7:00:00 p, m,	18.5	92	0
10/26/2020	8:00:00 p, m,	18.4	92	0
10/26/2020	9:00:00 p, m,	18.6	91	0
10/26/2020	10:00:00 p, m	18.5	90	0
10/26/2020	11:00:00 p, m	18.2	90	0
10/27/2020	12:00:00 a, m	17.9	89	0
10/27/2020	1:00:00 a, m,	17.8	88	0
10/27/2020	2:00:00 a, m,	17.7	91	0
10/27/2020	3:00:00 a, m,	17.2	90	0
10/27/2020	4:00:00 a, m,	17.1	90	0
10/27/2020	5:00:00 a, m,	17.2	91	0

10/27/2020	6:00:00 a, m,	16.9	92	0
10/27/2020	7:00:00 a, m,	17.6	92	0
10/27/2020	8:00:00 a, m,	18.2	93	0
10/27/2020	9:00:00 a, m,	18.6	96	0
10/27/2020	10:00:00 a, m	18.7	97	0
10/27/2020	11:00:00 a, m	19.3	97	0
10/27/2020	12:00:00 p, m	20.7	92	0
10/27/2020	1:00:00 p, m,	21.5	88	0
10/27/2020	2:00:00 p, m,	21.7	86	0
10/27/2020	3:00:00 p, m,	21.6	86	0
10/27/2020	4:00:00 p, m,	21.5	85	0
10/27/2020	5:00:00 p, m,	21.2	83	0
10/27/2020	6:00:00 p, m,	20.4	84	0
10/27/2020	7:00:00 p, m,	20.2	83	0
10/27/2020	8:00:00 p, m,	19.7	83	0
10/27/2020	9:00:00 p, m,	19.7	84	0
10/27/2020	10:00:00 p, m	19.2	89	0
10/27/2020	11:00:00 p, m	19.2	90	0
10/28/2020	12:00:00 a, m	18.5	91	0
10/28/2020	1:00:00 a, m,	18.1	92	0
10/28/2020	2:00:00 a, m,	18.2	91	0
10/28/2020	3:00:00 a, m,	17.9	93	0
10/28/2020	4:00:00 a, m,	17.8	93	0
10/28/2020	5:00:00 a, m,	17.8	93	0
10/28/2020	6:00:00 a, m,	17.7	94	0
10/28/2020	7:00:00 a, m,	17.9	94	0
10/28/2020	8:00:00 a, m,	18.2	95	0
10/28/2020	9:00:00 a, m,	18.2	95	0
10/28/2020	10:00:00 a, m	19.4	91	0
10/28/2020	11:00:00 a, m	19.9	91	0
10/28/2020	12:00:00 p, m	19.9	93	0
10/28/2020	1:00:00 p, m,	18.7	97	1.8
10/28/2020	2:00:00 p, m,	19.7	95	0
10/28/2020	3:00:00 p, m,	19.9	95	0
10/28/2020	4:00:00 p, m,	19.8	93	0
10/28/2020	5:00:00 p, m,	19.2	94	0
10/28/2020	6:00:00 p, m,	18.8	95	0
10/28/2020	7:00:00 p, m,	18.4	95	0
10/28/2020	8:00:00 p, m,	16.7	93	0.8
10/28/2020	9:00:00 p, m,	15.8	95	0.2
10/28/2020	10:00:00 p, m	16.4	96	0
10/28/2020	11:00:00 p, m	16.8	96	0
10/29/2020	12:00:00 a, m	16.9	96	0
10/29/2020	1:00:00 a, m,	16.9	96	0
10/29/2020	2:00:00 a, m,	17.1	97	0
10/29/2020	3:00:00 a, m,	17.1	97	0
10/29/2020	4:00:00 a, m,	17.4	98	0

10/29/2020	5:00:00 a, m,	17.3	98	0.2
10/29/2020	6:00:00 a, m,	16.6	97	0.2
10/29/2020	7:00:00 a, m,	16.3	97	0
10/29/2020	8:00:00 a, m,	17	98	0
10/29/2020	9:00:00 a, m,	17.3	98	0
10/29/2020	10:00:00 a, m	17.8	98	0
10/29/2020	11:00:00 a, m	18.2	97	0
10/29/2020	12:00:00 p, m	18.7	97	0
10/29/2020	1:00:00 p, m,	19.2	96	0
10/29/2020	2:00:00 p, m,	19.1	95	0
10/29/2020	3:00:00 p, m,	19.6	95	0
10/29/2020	4:00:00 p, m,	19.2	93	0
10/29/2020	5:00:00 p, m,	19	93	0
10/29/2020	6:00:00 p, m,	18.6	92	0
10/29/2020	7:00:00 p, m,	18.1	94	0
10/29/2020	8:00:00 p, m,	18	92	0
10/29/2020	9:00:00 p, m,	18.1	93	0
10/29/2020	10:00:00 p, m	18.2	92	0
10/29/2020	11:00:00 p, m	17.7	93	0.2
10/30/2020	12:00:00 a, m	17.4	93	0
10/30/2020	1:00:00 a, m,	17.4	94	0
10/30/2020	2:00:00 a, m,	17.5	95	0
10/30/2020	3:00:00 a, m,	17.2	96	0
10/30/2020	4:00:00 a, m,	17.3	97	0.2
10/30/2020	5:00:00 a, m,	17.1	97	0.2
10/30/2020	6:00:00 a, m,	16.9	97	0
10/30/2020	7:00:00 a, m,	17.1	97	0
10/30/2020	8:00:00 a, m,	17.1	97	2.8
10/30/2020	9:00:00 a, m,	16.9	98	0.2
10/30/2020	10:00:00 a, m	17.9	99	0
10/30/2020	11:00:00 a, m	18.5	98	0.2
10/30/2020	12:00:00 p, m	19.1	99	0
10/30/2020	1:00:00 p, m,	19.1	96	0
10/30/2020	2:00:00 p, m,	19.6	96	0
10/30/2020	3:00:00 p, m,	19.4	95	0
10/30/2020	4:00:00 p, m,	19.1	96	0
10/30/2020	5:00:00 p, m,	19	97	0
10/30/2020	6:00:00 p, m,	18.6	96	0
10/30/2020	7:00:00 p, m,	18.1	96	0
10/30/2020	8:00:00 p, m,	17.7	96	0
10/30/2020	9:00:00 p, m,	17.3	94	0
10/30/2020	10:00:00 p, m	17.3	95	0
10/30/2020	11:00:00 p, m	17.1	94	0
10/31/2020	12:00:00 a, m	17.2	95	0
10/31/2020	1:00:00 a, m,	17.2	94	0
10/31/2020	2:00:00 a, m,	17.3	96	0
10/31/2020	3:00:00 a, m,	17.3	95	0

10/31/2020	4:00:00 a, m,	17.2	96	0
10/31/2020	5:00:00 a, m,	17.1	96	0
10/31/2020	6:00:00 a, m,	16.8	97	5.2
10/31/2020	7:00:00 a, m,	16.4	98	7.8
10/31/2020	8:00:00 a, m,	16.7	98	1.4
10/31/2020	9:00:00 a, m,	16.9	98	4.6
10/31/2020	10:00:00 a, m	17.3	99	0.4
10/31/2020	11:00:00 a, m	18.1	99	0
10/31/2020	12:00:00 p, m	18.3	99	0.2
10/31/2020	1:00:00 p, m,	18.3	99	0
10/31/2020	2:00:00 p, m,	17.9	99	0
10/31/2020	3:00:00 p, m,	17.7	99	0.4
10/31/2020	4:00:00 p, m,	18.4	99	0
10/31/2020	5:00:00 p, m,	18.4	99	0
10/31/2020	6:00:00 p, m,	17.8	98	0
10/31/2020	7:00:00 p, m,	17.7	98	0
10/31/2020	8:00:00 p, m,	17.6	98	0
10/31/2020	9:00:00 p, m,	17.2	98	0
10/31/2020	10:00:00 p, m	17.2	99	0
10/31/2020	11:00:00 p, m	17.1	98	0
Oct_20		19.0715054	78.9139785	39.6
11/1/2020	12:00:00 a, m	17.1	98	0
11/1/2020	1:00:00 a, m,	17.1	98	0
11/1/2020	2:00:00 a, m,	16.9	98	0
11/1/2020	3:00:00 a, m,	16.8	98	0.2
11/1/2020	4:00:00 a, m,	16.8	98	3.6
11/1/2020	5:00:00 a, m,	16.6	98	1
11/1/2020	6:00:00 a, m,	16.6	99	0
11/1/2020	7:00:00 a, m,	16.7	99	0.2
11/1/2020	8:00:00 a, m,	16.8	99	0
11/1/2020	9:00:00 a, m,	17.5	99	0
11/1/2020	10:00:00 a, m	17.7	99	0
11/1/2020	11:00:00 a, m	17.4	99	0
11/1/2020	12:00:00 p, m	18.1	99	0
11/1/2020	1:00:00 p, m,	18.7	99	0
11/1/2020	2:00:00 p, m,	19.1	98	0
11/1/2020	3:00:00 p, m,	19.4	97	0
11/1/2020	4:00:00 p, m,	19.3	98	0
11/1/2020	5:00:00 p, m,	19.5	98	0
11/1/2020	6:00:00 p, m,	19.1	97	0
11/1/2020	7:00:00 p, m,	18.7	97	0
11/1/2020	8:00:00 p, m,	18.7	98	0
11/1/2020	9:00:00 p, m,	18.6	98	0
11/1/2020	10:00:00 p, m	18.4	98	0
11/1/2020	11:00:00 p, m	18.3	98	0
11/2/2020	12:00:00 a, m	18	98	0
11/2/2020	1:00:00 a, m,	17.9	98	0

11/2/2020	2:00:00 a, m,	17.6	98	0
11/2/2020	3:00:00 a, m,	17.4	98	0
11/2/2020	4:00:00 a, m,	17.4	98	0
11/2/2020	5:00:00 a, m,	17.2	98	0.2
11/2/2020	6:00:00 a, m,	16.4	98	6.4
11/2/2020	7:00:00 a, m,	16.9	99	0
11/2/2020	8:00:00 a, m,	17.3	99	0.2
11/2/2020	9:00:00 a, m,	17.8	99	0
11/2/2020	10:00:00 a, m	18.5	99	0
11/2/2020	11:00:00 a, m	18.6	99	0
11/2/2020	12:00:00 p, m	19.5	99	0.2
11/2/2020	1:00:00 p, m,	20.2	99	0
11/2/2020	2:00:00 p, m,	20.9	95	0
11/2/2020	3:00:00 p, m,	21.7	94	0
11/2/2020	4:00:00 p, m,	21.6	93	0
11/2/2020	5:00:00 p, m,	21	92	0
11/2/2020	6:00:00 p, m,	19.4	93	0
11/2/2020	7:00:00 p, m,	18.9	95	0
11/2/2020	8:00:00 p, m,	18.7	95	0
11/2/2020	9:00:00 p, m,	18.7	96	0
11/2/2020	10:00:00 p, m	18.6	95	0
11/2/2020	11:00:00 p, m	18.4	95	0
11/3/2020	12:00:00 a, m	18.4	96	0
11/3/2020	1:00:00 a, m,	18.3	96	0
11/3/2020	2:00:00 a, m,	17.9	96	0
11/3/2020	3:00:00 a, m,	17.8	97	0
11/3/2020	4:00:00 a, m,	17.3	97	0
11/3/2020	5:00:00 a, m,	17.3	97	0
11/3/2020	6:00:00 a, m,	17.2	96	0
11/3/2020	7:00:00 a, m,	17.3	96	0
11/3/2020	8:00:00 a, m,	17.9	98	0
11/3/2020	9:00:00 a, m,	18.4	98	0
11/3/2020	10:00:00 a, m	18.9	97	0
11/3/2020	11:00:00 a, m	19.8	94	0
11/3/2020	12:00:00 p, m	20.8	91	0
11/3/2020	1:00:00 p, m,	21.8	87	0
11/3/2020	2:00:00 p, m,	22.1	86	0
11/3/2020	3:00:00 p, m,	22.3	86	0
11/3/2020	4:00:00 p, m,	22.2	86	0
11/3/2020	5:00:00 p, m,	21.8	90	0
11/3/2020	6:00:00 p, m,	20.6	90	0
11/3/2020	7:00:00 p, m,	19.8	92	0
11/3/2020	8:00:00 p, m,	19.6	92	0
11/3/2020	9:00:00 p, m,	19.2	91	0
11/3/2020	10:00:00 p, m	18.6	91	0
11/3/2020	11:00:00 p, m	18.7	93	0
11/4/2020	12:00:00 a, m	17.4	97	1.6

11/4/2020	1:00:00 a, m,	17.2	97	0.2
11/4/2020	2:00:00 a, m,	16.9	97	0.2
11/4/2020	3:00:00 a, m,	16.7	97	0.2
11/4/2020	4:00:00 a, m,	16.5	97	0
11/4/2020	5:00:00 a, m,	16.5	97	0
11/4/2020	6:00:00 a, m,	16.6	97	0
11/4/2020	7:00:00 a, m,	17	97	0
11/4/2020	8:00:00 a, m,	17.6	97	0
11/4/2020	9:00:00 a, m,	17.9	97	0
11/4/2020	10:00:00 a, m	18.7	98	0
11/4/2020	11:00:00 a, m	19.1	96	0
11/4/2020	12:00:00 p, m	20.6	95	0
11/4/2020	1:00:00 p, m,	21.1	91	0
11/4/2020	2:00:00 p, m,	21.7	90	0
11/4/2020	3:00:00 p, m,	22.1	90	0
11/4/2020	4:00:00 p, m,	21.9	86	0
11/4/2020	5:00:00 p, m,	20.8	89	0
11/4/2020	6:00:00 p, m,	19.8	88	0
11/4/2020	7:00:00 p, m,	19.3	85	0
11/4/2020	8:00:00 p, m,	19.2	86	0
11/4/2020	9:00:00 p, m,	19.2	87	0
11/4/2020	10:00:00 p, m	18.8	87	0
11/4/2020	11:00:00 p, m	18.3	86	0
11/5/2020	12:00:00 a, m	17.7	86	0
11/5/2020	1:00:00 a, m,	17.6	85	0
11/5/2020	2:00:00 a, m,	17.4	88	0
11/5/2020	3:00:00 a, m,	17.1	90	0
11/5/2020	4:00:00 a, m,	17.2	90	0
11/5/2020	5:00:00 a, m,	17.1	90	0
11/5/2020	6:00:00 a, m,	16.9	92	0
11/5/2020	7:00:00 a, m,	17.5	89	0
11/5/2020	8:00:00 a, m,	17.8	91	0
11/5/2020	9:00:00 a, m,	17.9	95	0
11/5/2020	10:00:00 a, m	18.8	95	0
11/5/2020	11:00:00 a, m	20	93	0
11/5/2020	12:00:00 p, m	21	90	0
11/5/2020	1:00:00 p, m,	21.8	85	0
11/5/2020	2:00:00 p, m,	22.2	86	0
11/5/2020	3:00:00 p, m,	21.7	88	0
11/5/2020	4:00:00 p, m,	21.6	86	0
11/5/2020	5:00:00 p, m,	20.8	90	0
11/5/2020	6:00:00 p, m,	20.3	90	0
11/5/2020	7:00:00 p, m,	19.7	92	0
11/5/2020	8:00:00 p, m,	19.3	93	0
11/5/2020	9:00:00 p, m,	19.2	93	0
11/5/2020	10:00:00 p, m	18.9	94	0
11/5/2020	11:00:00 p, m	18.6	94	0

11/6/2020	12:00:00 a, m	18.4	93	0
11/6/2020	1:00:00 a, m,	18	95	0
11/6/2020	2:00:00 a, m,	17.9	96	0
11/6/2020	3:00:00 a, m,	17.8	97	4.6
11/6/2020	4:00:00 a, m,	17.4	97	1.8
11/6/2020	5:00:00 a, m,	17.2	98	0
11/6/2020	6:00:00 a, m,	17.3	98	0
11/6/2020	7:00:00 a, m,	17.4	98	0
11/6/2020	8:00:00 a, m,	17.6	98	0
11/6/2020	9:00:00 a, m,	18	99	0
11/6/2020	10:00:00 a, m	18.3	99	0
11/6/2020	11:00:00 a, m	18.5	99	0
11/6/2020	12:00:00 p, m	18.7	98	0
11/6/2020	1:00:00 p, m,	19.1	98	0.2
11/6/2020	2:00:00 p, m,	19.4	96	0
11/6/2020	3:00:00 p, m,	19.2	98	0
11/6/2020	4:00:00 p, m,	18.9	98	0
11/6/2020	5:00:00 p, m,	18.9	98	0
11/6/2020	6:00:00 p, m,	18.6	97	0
11/6/2020	7:00:00 p, m,	17.8	97	0
11/6/2020	8:00:00 p, m,	17.3	95	0
11/6/2020	9:00:00 p, m,	17.5	97	0
11/6/2020	10:00:00 p, m	17.6	96	0
11/6/2020	11:00:00 p, m	17.2	95	0
11/7/2020	12:00:00 a, m	17.4	97	0
11/7/2020	1:00:00 a, m,	17.1	95	0
11/7/2020	2:00:00 a, m,	17.1	96	0
11/7/2020	3:00:00 a, m,	17	96	0
11/7/2020	4:00:00 a, m,	17.1	97	0
11/7/2020	5:00:00 a, m,	16.5	97	2.4
11/7/2020	6:00:00 a, m,	15.8	98	5.6
11/7/2020	7:00:00 a, m,	15.7	98	0.4
11/7/2020	8:00:00 a, m,	16.1	98	0.2
11/7/2020	9:00:00 a, m,	16.9	99	0
11/7/2020	10:00:00 a, m	17.9	98	0
11/7/2020	11:00:00 a, m	18.8	98	0
11/7/2020	12:00:00 p, m	19.3	96	0
11/7/2020	1:00:00 p, m,	19.7	94	0
11/7/2020	2:00:00 p, m,	19.6	94	0
11/7/2020	3:00:00 p, m,	19.8	94	0
11/7/2020	4:00:00 p, m,	19.6	93	0
11/7/2020	5:00:00 p, m,	19.1	92	0
11/7/2020	6:00:00 p, m,	18.7	87	0
11/7/2020	7:00:00 p, m,	18.3	87	0
11/7/2020	8:00:00 p, m,	17.7	90	0
11/7/2020	9:00:00 p, m,	17.6	90	0
11/7/2020	10:00:00 p, m	17.5	89	0

11/7/2020	11:00:00 p, m	17	89	0
11/8/2020	12:00:00 a, m	16.5	90	0
11/8/2020	1:00:00 a, m,	16.3	90	0
11/8/2020	2:00:00 a, m,	16.2	90	0
11/8/2020	3:00:00 a, m,	16.2	91	0
11/8/2020	4:00:00 a, m,	16	91	0
11/8/2020	5:00:00 a, m,	16.1	93	0
11/8/2020	6:00:00 a, m,	15.7	92	0
11/8/2020	7:00:00 a, m,	16.3	91	0
11/8/2020	8:00:00 a, m,	16.9	90	0
11/8/2020	9:00:00 a, m,	17.5	93	0
11/8/2020	10:00:00 a, m	18.6	92	0
11/8/2020	11:00:00 a, m	19.5	90	0
11/8/2020	12:00:00 p, m	21.1	88	0
11/8/2020	1:00:00 p, m,	21.5	85	0
11/8/2020	2:00:00 p, m,	21.1	85	0
11/8/2020	3:00:00 p, m,	21.4	80	0
11/8/2020	4:00:00 p, m,	20.7	86	0
11/8/2020	5:00:00 p, m,	20.8	87	0
11/8/2020	6:00:00 p, m,	19.7	86	0
11/8/2020	7:00:00 p, m,	18.9	86	0
11/8/2020	8:00:00 p, m,	18.8	80	0
11/8/2020	9:00:00 p, m,	18.3	80	0
11/8/2020	10:00:00 p, m	18.3	80	0
11/8/2020	11:00:00 p, m	18.1	80	0
11/9/2020	12:00:00 a, m	17.3	83	0
11/9/2020	1:00:00 a, m,	17.1	82	0
11/9/2020	2:00:00 a, m,	17.1	83	0
11/9/2020	3:00:00 a, m,	17.5	81	0
11/9/2020	4:00:00 a, m,	17.1	82	0
11/9/2020	5:00:00 a, m,	16.2	85	0
11/9/2020	6:00:00 a, m,	16.4	84	0
11/9/2020	7:00:00 a, m,	16.4	85	0
11/9/2020	8:00:00 a, m,	16.7	86	0
11/9/2020	9:00:00 a, m,	17.3	88	0
11/9/2020	10:00:00 a, m	18.6	87	0
11/9/2020	11:00:00 a, m	19.4	88	0
11/9/2020	12:00:00 p, m	20.2	85	0
11/9/2020	1:00:00 p, m,	21.5	81	0
11/9/2020	2:00:00 p, m,	22.1	80	0
11/9/2020	3:00:00 p, m,	21.5	79	0
11/9/2020	4:00:00 p, m,	22	78	0
11/9/2020	5:00:00 p, m,	22.1	81	0
11/9/2020	6:00:00 p, m,	20.4	82	0
11/9/2020	7:00:00 p, m,	19.3	83	0
11/9/2020	8:00:00 p, m,	18.9	84	0
11/9/2020	9:00:00 p, m,	18.3	85	0

11/9/2020	10:00:00 p, m	18	86	0
11/9/2020	11:00:00 p, m	17.5	85	0
11/10/2020	12:00:00 a, m	17.2	87	0
11/10/2020	1:00:00 a, m,	16.8	89	0
11/10/2020	2:00:00 a, m,	16.6	89	0
11/10/2020	3:00:00 a, m,	16.6	90	0
11/10/2020	4:00:00 a, m,	16.6	90	0
11/10/2020	5:00:00 a, m,	16.8	89	0
11/10/2020	6:00:00 a, m,	16.7	93	0.2
11/10/2020	7:00:00 a, m,	16.5	96	0
11/10/2020	8:00:00 a, m,	17.1	95	0
11/10/2020	9:00:00 a, m,	17.9	96	0
11/10/2020	10:00:00 a, m	18.1	97	0
11/10/2020	11:00:00 a, m	18.4	97	0
11/10/2020	12:00:00 p, m	18.8	95	0
11/10/2020	1:00:00 p, m,	18.9	94	0
11/10/2020	2:00:00 p, m,	19.9	94	0
11/10/2020	3:00:00 p, m,	19.7	94	0
11/10/2020	4:00:00 p, m,	19.4	95	0
11/10/2020	5:00:00 p, m,	19.3	95	0
11/10/2020	6:00:00 p, m,	18.9	93	0
11/10/2020	7:00:00 p, m,	18.2	95	0
11/10/2020	8:00:00 p, m,	18.1	94	0
11/10/2020	9:00:00 p, m,	18	95	0
11/10/2020	10:00:00 p, m	17.9	93	0
11/10/2020	11:00:00 p, m	17.5	93	0
11/11/2020	12:00:00 a, m	17.6	94	0
11/11/2020	1:00:00 a, m,	17.6	93	0
11/11/2020	2:00:00 a, m,	17.4	93	0
11/11/2020	3:00:00 a, m,	17.2	94	0
11/11/2020	4:00:00 a, m,	17.2	94	0
11/11/2020	5:00:00 a, m,	17.1	94	0
11/11/2020	6:00:00 a, m,	17	96	1.6
11/11/2020	7:00:00 a, m,	16.8	98	0.2
11/11/2020	8:00:00 a, m,	17.2	98	0
11/11/2020	9:00:00 a, m,	17.7	98	0
11/11/2020	10:00:00 a, m	18	99	0
11/11/2020	11:00:00 a, m	17.7	98	0.2
11/11/2020	12:00:00 p, m	18.2	99	1
11/11/2020	1:00:00 p, m,	18	99	0
11/11/2020	2:00:00 p, m,	17.9	99	2.2
11/11/2020	3:00:00 p, m,	18.6	99	0
11/11/2020	4:00:00 p, m,	18.4	99	0
11/11/2020	5:00:00 p, m,	18.2	99	0
11/11/2020	6:00:00 p, m,	18.1	98	0
11/11/2020	7:00:00 p, m,	17.9	98	0
11/11/2020	8:00:00 p, m,	17.8	98	0

11/11/2020	9:00:00 p, m,	17.4	98	0
11/11/2020	10:00:00 p, m	17.4	98	0
11/11/2020	11:00:00 p, m	17.2	98	0
11/12/2020	12:00:00 a, m	16.9	97	0
11/12/2020	1:00:00 a, m,	16.7	97	0
11/12/2020	2:00:00 a, m,	16.5	97	0
11/12/2020	3:00:00 a, m,	16.4	97	0
11/12/2020	4:00:00 a, m,	16.4	97	0
11/12/2020	5:00:00 a, m,	16.2	97	0
11/12/2020	6:00:00 a, m,	16.2	97	0
11/12/2020	7:00:00 a, m,	16.4	98	0.2
11/12/2020	8:00:00 a, m,	16.3	98	2
11/12/2020	9:00:00 a, m,	16.8	99	1
11/12/2020	10:00:00 a, m	17.1	99	0
11/12/2020	11:00:00 a, m	18.1	99	0
11/12/2020	12:00:00 p, m	17.9	98	0.2
11/12/2020	1:00:00 p, m,	18.6	99	0.6
11/12/2020	2:00:00 p, m,	18.5	99	0
11/12/2020	3:00:00 p, m,	19.3	99	0
11/12/2020	4:00:00 p, m,	18.9	98	1.2
11/12/2020	5:00:00 p, m,	18.6	98	0
11/12/2020	6:00:00 p, m,	18.2	98	0
11/12/2020	7:00:00 p, m,	17.1	97	0
11/12/2020	8:00:00 p, m,	17.2	99	0
11/12/2020	9:00:00 p, m,	16.6	98	0
11/12/2020	10:00:00 p, m	16.6	97	0
11/12/2020	11:00:00 p, m	16.8	97	0
11/13/2020	12:00:00 a, m	16.9	96	0
11/13/2020	1:00:00 a, m,	16.8	97	0
11/13/2020	2:00:00 a, m,	16.7	96	0
11/13/2020	3:00:00 a, m,	16.1	96	0
11/13/2020	4:00:00 a, m,	16.2	97	0
11/13/2020	5:00:00 a, m,	16.6	97	0
11/13/2020	6:00:00 a, m,	16.6	97	0
11/13/2020	7:00:00 a, m,	16.8	97	0
11/13/2020	8:00:00 a, m,	17.3	98	0
11/13/2020	9:00:00 a, m,	17.4	98	0.4
11/13/2020	10:00:00 a, m	17.4	99	0.2
11/13/2020	11:00:00 a, m	17.3	99	0
11/13/2020	12:00:00 p, m	17.4	99	0
11/13/2020	1:00:00 p, m,	17.3	99	0
11/13/2020	2:00:00 p, m,	18.4	99	0
11/13/2020	3:00:00 p, m,	18.3	99	0
11/13/2020	4:00:00 p, m,	18.2	99	0
11/13/2020	5:00:00 p, m,	18.3	99	0
11/13/2020	6:00:00 p, m,	17.8	98	0
11/13/2020	7:00:00 p, m,	17.2	98	0

11/13/2020	8:00:00 p, m,	16.5	97	0
11/13/2020	9:00:00 p, m,	16.7	97	0
11/13/2020	10:00:00 p, m	16.5	96	0
11/13/2020	11:00:00 p, m	16.8	97	0
11/14/2020	12:00:00 a, m	16.7	96	0
11/14/2020	1:00:00 a, m,	16.5	97	0
11/14/2020	2:00:00 a, m,	16.7	98	0
11/14/2020	3:00:00 a, m,	16.7	97	0
11/14/2020	4:00:00 a, m,	16.3	97	0
11/14/2020	5:00:00 a, m,	16.3	98	0
11/14/2020	6:00:00 a, m,	16.3	97	0
11/14/2020	7:00:00 a, m,	16.4	97	0
11/14/2020	8:00:00 a, m,	17.2	98	0
11/14/2020	9:00:00 a, m,	17.6	99	0
11/14/2020	10:00:00 a, m	17.8	99	0
11/14/2020	11:00:00 a, m	18.4	98	0
11/14/2020	12:00:00 p, m	18.9	98	0
11/14/2020	1:00:00 p, m,	19.6	97	0
11/14/2020	2:00:00 p, m,	19.4	97	0
11/14/2020	3:00:00 p, m,	19.7	96	0
11/14/2020	4:00:00 p, m,	19.3	96	0
11/14/2020	5:00:00 p, m,	18.7	93	0
11/14/2020	6:00:00 p, m,	16.5	96	0.4
11/14/2020	7:00:00 p, m,	16.8	96	0
11/14/2020	8:00:00 p, m,	17.1	97	0
11/14/2020	9:00:00 p, m,	17.3	96	0
11/14/2020	10:00:00 p, m	17.3	96	0
11/14/2020	11:00:00 p, m	17.2	96	0
11/15/2020	12:00:00 a, m	17.1	95	0
11/15/2020	1:00:00 a, m,	16.7	96	0
11/15/2020	2:00:00 a, m,	16.6	97	0
11/15/2020	3:00:00 a, m,	16.3	97	0
11/15/2020	4:00:00 a, m,	16.3	97	0
11/15/2020	5:00:00 a, m,	16.3	97	0
11/15/2020	6:00:00 a, m,	16.2	96	0
11/15/2020	7:00:00 a, m,	16.3	96	0
11/15/2020	8:00:00 a, m,	16.8	97	0
11/15/2020	9:00:00 a, m,	17.2	97	0
11/15/2020	10:00:00 a, m	17.9	98	0
11/15/2020	11:00:00 a, m	18.4	99	0
11/15/2020	12:00:00 p, m	18.7	97	0.8
11/15/2020	1:00:00 p, m,	19.2	99	0
11/15/2020	2:00:00 p, m,	19.7	99	0
11/15/2020	3:00:00 p, m,	19.7	98	2
11/15/2020	4:00:00 p, m,	18.5	99	6.4
11/15/2020	5:00:00 p, m,	18.1	98	0.2
11/15/2020	6:00:00 p, m,	17.5	97	0

11/15/2020	7:00:00 p, m,	17.4	98	0
11/15/2020	8:00:00 p, m,	16.7	98	0.6
11/15/2020	9:00:00 p, m,	16.3	98	0.4
11/15/2020	10:00:00 p, m	16.2	98	0
11/15/2020	11:00:00 p, m	16.3	98	0
11/16/2020	12:00:00 a, m	16.5	98	0
11/16/2020	1:00:00 a, m,	16.4	97	0
11/16/2020	2:00:00 a, m,	16.4	97	0
11/16/2020	3:00:00 a, m,	16.4	98	0
11/16/2020	4:00:00 a, m,	16.2	98	0.6
11/16/2020	5:00:00 a, m,	16.2	98	0.8
11/16/2020	6:00:00 a, m,	16	98	0.4
11/16/2020	7:00:00 a, m,	15.9	98	0.2
11/16/2020	8:00:00 a, m,	16.3	99	0
11/16/2020	9:00:00 a, m,	17	99	0
11/16/2020	10:00:00 a, m	17.3	99	0
11/16/2020	11:00:00 a, m	17.9	99	0
11/16/2020	12:00:00 p, m	18.1	98	0
11/16/2020	1:00:00 p, m,	18.9	97	0
11/16/2020	2:00:00 p, m,	19.1	97	0
11/16/2020	3:00:00 p, m,	19.1	97	0
11/16/2020	4:00:00 p, m,	19.2	94	0
11/16/2020	5:00:00 p, m,	18.8	96	0
11/16/2020	6:00:00 p, m,	18.1	97	0
11/16/2020	7:00:00 p, m,	17.3	96	0
11/16/2020	8:00:00 p, m,	17.1	94	0
11/16/2020	9:00:00 p, m,	17	94	0
11/16/2020	10:00:00 p, m	16.7	93	0
11/16/2020	11:00:00 p, m	16.7	95	0
11/17/2020	12:00:00 a, m	16.4	94	0
11/17/2020	1:00:00 a, m,	16.3	93	0
11/17/2020	2:00:00 a, m,	16	93	0
11/17/2020	3:00:00 a, m,	15.7	94	0
11/17/2020	4:00:00 a, m,	15.7	95	0
11/17/2020	5:00:00 a, m,	15.8	95	0
11/17/2020	6:00:00 a, m,	16.2	96	0
11/17/2020	7:00:00 a, m,	16.6	97	0
11/17/2020	8:00:00 a, m,	16.9	95	0
11/17/2020	9:00:00 a, m,	17.6	96	0
11/17/2020	10:00:00 a, m	18	97	0
11/17/2020	11:00:00 a, m	18.5	97	0
11/17/2020	12:00:00 p, m	18.6	97	0.2
11/17/2020	1:00:00 p, m,	18.4	98	0
11/17/2020	2:00:00 p, m,	18.4	98	0.2
11/17/2020	3:00:00 p, m,	18.1	97	0
11/17/2020	4:00:00 p, m,	18.1	98	0
11/17/2020	5:00:00 p, m,	17.9	97	0

11/17/2020	6:00:00 p, m,	17.7	97	0
11/17/2020	7:00:00 p, m,	17.6	98	0
11/17/2020	8:00:00 p, m,	17.5	97	0
11/17/2020	9:00:00 p, m,	17.4	98	0.2
11/17/2020	10:00:00 p, m	17.3	98	0
11/17/2020	11:00:00 p, m	17.1	99	0.2
11/18/2020	12:00:00 a, m	17.1	98	0
11/18/2020	1:00:00 a, m,	16.9	99	0
11/18/2020	2:00:00 a, m,	16.6	98	0.2
11/18/2020	3:00:00 a, m,	16.4	98	0.2
11/18/2020	4:00:00 a, m,	16.4	98	0
11/18/2020	5:00:00 a, m,	16.7	99	0
11/18/2020	6:00:00 a, m,	16.6	99	0
11/18/2020	7:00:00 a, m,	16.1	99	0
11/18/2020	8:00:00 a, m,	16.3	99	0
11/18/2020	9:00:00 a, m,	17	99	0
11/18/2020	10:00:00 a, m	17.2	99	0
11/18/2020	11:00:00 a, m	17.4	99	0
11/18/2020	12:00:00 p, m	17.4	99	0
11/18/2020	1:00:00 p, m,	17.9	99	0
11/18/2020	2:00:00 p, m,	18.1	99	0
11/18/2020	3:00:00 p, m,	18.2	99	0
11/18/2020	4:00:00 p, m,	18.3	98	0
11/18/2020	5:00:00 p, m,	18.3	98	0.2
11/18/2020	6:00:00 p, m,	17.6	97	0
11/18/2020	7:00:00 p, m,	17	97	0
11/18/2020	8:00:00 p, m,	16.7	96	0
11/18/2020	9:00:00 p, m,	16.6	96	0
11/18/2020	10:00:00 p, m	16.9	95	0
11/18/2020	11:00:00 p, m	17.1	94	0
11/19/2020	12:00:00 a, m	16.9	95	0
11/19/2020	1:00:00 a, m,	16.9	95	0
11/19/2020	2:00:00 a, m,	16.8	95	0
11/19/2020	3:00:00 a, m,	16.6	97	0.2
11/19/2020	4:00:00 a, m,	16.6	98	0.6
11/19/2020	5:00:00 a, m,	16.4	98	0
11/19/2020	6:00:00 a, m,	16.5	99	0
11/19/2020	7:00:00 a, m,	16.6	99	0
11/19/2020	8:00:00 a, m,	16.6	99	0.8
11/19/2020	9:00:00 a, m,	16.6	99	0
11/19/2020	10:00:00 a, m	16.6	99	1.8
11/19/2020	11:00:00 a, m	16.7	99	2.8
11/19/2020	12:00:00 p, m	17.2	99	1.2
11/19/2020	1:00:00 p, m,	17.9	99	0.2
11/19/2020	2:00:00 p, m,	18.1	99	0
11/19/2020	3:00:00 p, m,	18.1	99	0
11/19/2020	4:00:00 p, m,	18	98	0

11/19/2020	5:00:00 p, m,	18.1	98	0
11/19/2020	6:00:00 p, m,	17.6	98	0
11/19/2020	7:00:00 p, m,	16.9	97	0
11/19/2020	8:00:00 p, m,	16.4	97	0
11/19/2020	9:00:00 p, m,	16.4	96	0
11/19/2020	10:00:00 p, m	16.3	97	0
11/19/2020	11:00:00 p, m	16.6	96	0
11/20/2020	12:00:00 a, m	16.5	96	0
11/20/2020	1:00:00 a, m,	16.2	96	0
11/20/2020	2:00:00 a, m,	16.4	96	0
11/20/2020	3:00:00 a, m,	16.6	97	0
11/20/2020	4:00:00 a, m,	16.5	96	0
11/20/2020	5:00:00 a, m,	16.6	97	0
11/20/2020	6:00:00 a, m,	16.5	96	0
11/20/2020	7:00:00 a, m,	16.5	98	0
11/20/2020	8:00:00 a, m,	16.9	98	0
11/20/2020	9:00:00 a, m,	17.3	98	0
11/20/2020	10:00:00 a, m	17.4	99	0
11/20/2020	11:00:00 a, m	17.8	98	0
11/20/2020	12:00:00 p, m	18.6	98	0
11/20/2020	1:00:00 p, m,	18	98	0.6
11/20/2020	2:00:00 p, m,	18.3	99	0.2
11/20/2020	3:00:00 p, m,	17.8	98	1.8
11/20/2020	4:00:00 p, m,	17.7	98	0.4
11/20/2020	5:00:00 p, m,	17.3	98	0
11/20/2020	6:00:00 p, m,	17.2	98	0
11/20/2020	7:00:00 p, m,	17.1	98	0
11/20/2020	8:00:00 p, m,	16.6	97	0
11/20/2020	9:00:00 p, m,	16.6	98	0
11/20/2020	10:00:00 p, m	16.8	98	0
11/20/2020	11:00:00 p, m	16.3	97	0
11/21/2020	12:00:00 a, m	16.2	96	0
11/21/2020	1:00:00 a, m,	16.1	95	0
11/21/2020	2:00:00 a, m,	16.1	93	0
11/21/2020	3:00:00 a, m,	15.9	94	0
11/21/2020	4:00:00 a, m,	15.8	93	0
11/21/2020	5:00:00 a, m,	15.9	93	0
11/21/2020	6:00:00 a, m,	15.7	94	0
11/21/2020	7:00:00 a, m,	16.2	94	0
11/21/2020	8:00:00 a, m,	16.9	95	0
11/21/2020	9:00:00 a, m,	17.6	95	0
11/21/2020	10:00:00 a, m	18.1	97	0
11/21/2020	11:00:00 a, m	19	96	0
11/21/2020	12:00:00 p, m	19.4	95	0
11/21/2020	1:00:00 p, m,	20.2	94	0
11/21/2020	2:00:00 p, m,	20.3	94	0
11/21/2020	3:00:00 p, m,	20.4	96	0

11/21/2020	4:00:00 p, m,	20.4	92	0
11/21/2020	5:00:00 p, m,	20.4	92	0
11/21/2020	6:00:00 p, m,	19.7	94	0
11/21/2020	7:00:00 p, m,	18.9	94	0
11/21/2020	8:00:00 p, m,	18.3	93	0
11/21/2020	9:00:00 p, m,	17.8	92	0
11/21/2020	10:00:00 p, m	17.6	92	0
11/21/2020	11:00:00 p, m	17.3	92	0
11/22/2020	12:00:00 a, m	17.1	93	0
11/22/2020	1:00:00 a, m,	17.1	93	0
11/22/2020	2:00:00 a, m,	17.1	93	0
11/22/2020	3:00:00 a, m,	16.7	94	0
11/22/2020	4:00:00 a, m,	16.5	97	0
11/22/2020	5:00:00 a, m,	16.2	95	0
11/22/2020	6:00:00 a, m,	16	95	0
11/22/2020	7:00:00 a, m,	16.1	95	0
11/22/2020	8:00:00 a, m,	16.5	95	0
11/22/2020	9:00:00 a, m,	17.1	98	0
11/22/2020	10:00:00 a, m	18.2	98	0
11/22/2020	11:00:00 a, m	18.4	97	0
11/22/2020	12:00:00 p, m	18.8	97	0
11/22/2020	1:00:00 p, m,	20.1	95	0
11/22/2020	2:00:00 p, m,	19.7	97	0.4
11/22/2020	3:00:00 p, m,	19.9	98	0.6
11/22/2020	4:00:00 p, m,	19.8	98	0.2
11/22/2020	5:00:00 p, m,	18.1	96	0.2
11/22/2020	6:00:00 p, m,	17.8	96	0
11/22/2020	7:00:00 p, m,	17.3	95	0
11/22/2020	8:00:00 p, m,	17.4	95	0
11/22/2020	9:00:00 p, m,	17.3	94	0
11/22/2020	10:00:00 p, m	17.4	96	0
11/22/2020	11:00:00 p, m	17	95	0
11/23/2020	12:00:00 a, m	16.6	92	0
11/23/2020	1:00:00 a, m,	17.2	97	0
11/23/2020	2:00:00 a, m,	16.8	96	0
11/23/2020	3:00:00 a, m,	16.7	96	0
11/23/2020	4:00:00 a, m,	16.3	94	0
11/23/2020	5:00:00 a, m,	16.1	95	0
11/23/2020	6:00:00 a, m,	16.2	94	0
11/23/2020	7:00:00 a, m,	16.3	95	0
11/23/2020	8:00:00 a, m,	16.8	96	0
11/23/2020	9:00:00 a, m,	17.1	96	0
11/23/2020	10:00:00 a, m	17.4	97	0.2
11/23/2020	11:00:00 a, m	17.8	98	0
11/23/2020	12:00:00 p, m	18.2	98	0
11/23/2020	1:00:00 p, m,	19.6	94	0
11/23/2020	2:00:00 p, m,	19	97	0.6

11/23/2020	3:00:00 p, m,	18.6	97	0.8
11/23/2020	4:00:00 p, m,	18.7	97	0
11/23/2020	5:00:00 p, m,	18.7	97	1.4
11/23/2020	6:00:00 p, m,	17.7	97	0
11/23/2020	7:00:00 p, m,	17.2	97	0
11/23/2020	8:00:00 p, m,	17.3	97	0
11/23/2020	9:00:00 p, m,	17.1	95	0
11/23/2020	10:00:00 p, m	17.4	90	0
11/23/2020	11:00:00 p, m	17.6	89	0
11/24/2020	12:00:00 a, m	16.9	90	0
11/24/2020	1:00:00 a, m,	16.9	90	0
11/24/2020	2:00:00 a, m,	16.8	90	0
11/24/2020	3:00:00 a, m,	16.6	90	0
11/24/2020	4:00:00 a, m,	16.3	89	0
11/24/2020	5:00:00 a, m,	16.4	91	0
11/24/2020	6:00:00 a, m,	16.2	90	0
11/24/2020	7:00:00 a, m,	16.5	93	0
11/24/2020	8:00:00 a, m,	17.3	92	0
11/24/2020	9:00:00 a, m,	18.2	92	0
11/24/2020	10:00:00 a, m	18.9	93	0
11/24/2020	11:00:00 a, m	19.3	93	0
11/24/2020	12:00:00 p, m	20.4	92	0
11/24/2020	1:00:00 p, m,	20.9	90	0
11/24/2020	2:00:00 p, m,	20.9	91	0
11/24/2020	3:00:00 p, m,	21.5	89	0
11/24/2020	4:00:00 p, m,	21.9	88	0
11/24/2020	5:00:00 p, m,	20.8	88	0
11/24/2020	6:00:00 p, m,	19.8	87	0
11/24/2020	7:00:00 p, m,	19.3	87	0
11/24/2020	8:00:00 p, m,	19.3	84	0
11/24/2020	9:00:00 p, m,	19	84	0
11/24/2020	10:00:00 p, m	18.7	83	0
11/24/2020	11:00:00 p, m	19.1	81	0
11/25/2020	12:00:00 a, m	18.4	83	0
11/25/2020	1:00:00 a, m,	17.6	84	0
11/25/2020	2:00:00 a, m,	17.8	83	0
11/25/2020	3:00:00 a, m,	17.8	82	0
11/25/2020	4:00:00 a, m,	17.6	85	0
11/25/2020	5:00:00 a, m,	17.3	86	0
11/25/2020	6:00:00 a, m,	17.8	84	0
11/25/2020	7:00:00 a, m,	17.3	89	0
11/25/2020	8:00:00 a, m,	17.9	89	0
11/25/2020	9:00:00 a, m,	18.4	90	0
11/25/2020	10:00:00 a, m	19.3	86	0
11/25/2020	11:00:00 a, m	20.1	89	0
11/25/2020	12:00:00 p, m	21	88	0
11/25/2020	1:00:00 p, m,	21.4	87	0

11/25/2020	2:00:00 p, m,	22	84	0
11/25/2020	3:00:00 p, m,	21.9	86	0
11/25/2020	4:00:00 p, m,	21.1	87	0
11/25/2020	5:00:00 p, m,	21.1	84	0
11/25/2020	6:00:00 p, m,	20.2	84	0
11/25/2020	7:00:00 p, m,	19.2	88	0
11/25/2020	8:00:00 p, m,	18.8	87	0
11/25/2020	9:00:00 p, m,	18.6	88	0
11/25/2020	10:00:00 p, m	18.5	88	0
11/25/2020	11:00:00 p, m	18.2	88	0
11/26/2020	12:00:00 a, m	17.9	89	0
11/26/2020	1:00:00 a, m,	18.1	89	0
11/26/2020	2:00:00 a, m,	17.4	91	0
11/26/2020	3:00:00 a, m,	16.9	91	0
11/26/2020	4:00:00 a, m,	16.9	92	0
11/26/2020	5:00:00 a, m,	16.7	92	0
11/26/2020	6:00:00 a, m,	16.8	95	0
11/26/2020	7:00:00 a, m,	17.3	94	0
11/26/2020	8:00:00 a, m,	17.8	94	0
11/26/2020	9:00:00 a, m,	18.6	96	0
11/26/2020	10:00:00 a, m	18.5	97	0
11/26/2020	11:00:00 a, m	18.8	98	0.2
11/26/2020	12:00:00 p, m	19.8	96	0.2
11/26/2020	1:00:00 p, m,	20.3	94	0
11/26/2020	2:00:00 p, m,	21.1	93	0
11/26/2020	3:00:00 p, m,	20.2	96	0
11/26/2020	4:00:00 p, m,	20.5	96	0
11/26/2020	5:00:00 p, m,	20.2	96	0
11/26/2020	6:00:00 p, m,	19.5	93	0
11/26/2020	7:00:00 p, m,	19	96	0
11/26/2020	8:00:00 p, m,	18.4	91	0
11/26/2020	9:00:00 p, m,	18.4	93	0
11/26/2020	10:00:00 p, m	18.2	94	0
11/26/2020	11:00:00 p, m	17.5	95	0
11/27/2020	12:00:00 a, m	17.4	95	0.2
11/27/2020	1:00:00 a, m,	17.3	95	0
11/27/2020	2:00:00 a, m,	17.1	96	0
11/27/2020	3:00:00 a, m,	17.1	93	0
11/27/2020	4:00:00 a, m,	16.7	95	0
11/27/2020	5:00:00 a, m,	16.8	95	0
11/27/2020	6:00:00 a, m,	16.4	95	0
11/27/2020	7:00:00 a, m,	16.8	96	0
11/27/2020	8:00:00 a, m,	17.4	95	0
11/27/2020	9:00:00 a, m,	18.1	97	0
11/27/2020	10:00:00 a, m	18.2	97	0.2
11/27/2020	11:00:00 a, m	18.4	98	0.2
11/27/2020	12:00:00 p, m	18	97	3

11/27/2020	1:00:00 p, m,	16.7	98	7.6
11/27/2020	2:00:00 p, m,	16.9	98	1.4
11/27/2020	3:00:00 p, m,	17.3	98	0
11/27/2020	4:00:00 p, m,	17.7	99	0
11/27/2020	5:00:00 p, m,	18.1	98	0
11/27/2020	6:00:00 p, m,	17.8	98	0
11/27/2020	7:00:00 p, m,	17.3	97	0
11/27/2020	8:00:00 p, m,	16.9	97	0
11/27/2020	9:00:00 p, m,	16.8	97	0
11/27/2020	10:00:00 p, m	16.8	96	0
11/27/2020	11:00:00 p, m	16.8	96	0
11/28/2020	12:00:00 a, m	16.7	96	0
11/28/2020	1:00:00 a, m,	16.6	96	0
11/28/2020	2:00:00 a, m,	16.5	97	0
11/28/2020	3:00:00 a, m,	16.4	97	0.2
11/28/2020	4:00:00 a, m,	16.2	98	0.4
11/28/2020	5:00:00 a, m,	16.1	98	0.2
11/28/2020	6:00:00 a, m,	15.8	98	1.4
11/28/2020	7:00:00 a, m,	15.8	98	1.6
11/28/2020	8:00:00 a, m,	16.4	99	0.4
11/28/2020	9:00:00 a, m,	17	99	0.8
11/28/2020	10:00:00 a, m	17.4	99	0
11/28/2020	11:00:00 a, m	17.7	99	0
11/28/2020	12:00:00 p, m	18	99	0
11/28/2020	1:00:00 p, m,	18.3	99	3.4
11/28/2020	2:00:00 p, m,	18.8	99	0.2
11/28/2020	3:00:00 p, m,	19.1	99	0
11/28/2020	4:00:00 p, m,	19	99	0.2
11/28/2020	5:00:00 p, m,	17.4	97	2
11/28/2020	6:00:00 p, m,	16.1	97	0.4
11/28/2020	7:00:00 p, m,	16.1	98	0
11/28/2020	8:00:00 p, m,	16.4	98	0
11/28/2020	9:00:00 p, m,	16.5	98	0
11/28/2020	10:00:00 p, m	17.2	99	0
11/28/2020	11:00:00 p, m	17.1	98	0
11/29/2020	12:00:00 a, m	16.8	98	0
11/29/2020	1:00:00 a, m,	16.7	98	0
11/29/2020	2:00:00 a, m,	16.7	98	0
11/29/2020	3:00:00 a, m,	16.7	98	0
11/29/2020	4:00:00 a, m,	16.6	99	0
11/29/2020	5:00:00 a, m,	16.6	99	0.2
11/29/2020	6:00:00 a, m,	16.6	99	0.2
11/29/2020	7:00:00 a, m,	16.1	98	11.2
11/29/2020	8:00:00 a, m,	16.6	99	2
11/29/2020	9:00:00 a, m,	17.2	99	0.4
11/29/2020	10:00:00 a, m	17.6	99	0
11/29/2020	11:00:00 a, m	17.9	99	0

11/29/2020	12:00:00 p, m	17.9	99	0
11/29/2020	1:00:00 p, m,	18.2	99	0
11/29/2020	2:00:00 p, m,	19.1	99	0
11/29/2020	3:00:00 p, m,	19.2	98	0
11/29/2020	4:00:00 p, m,	19.6	98	0
11/29/2020	5:00:00 p, m,	19.2	97	0
11/29/2020	6:00:00 p, m,	17.9	94	0
11/29/2020	7:00:00 p, m,	17	93	0
11/29/2020	8:00:00 p, m,	16.8	94	0
11/29/2020	9:00:00 p, m,	17.1	93	0
11/29/2020	10:00:00 p, m	17.4	93	0
11/29/2020	11:00:00 p, m	17.6	90	0
11/30/2020	12:00:00 a, m	17.5	90	0
11/30/2020	1:00:00 a, m,	16.8	90	0
11/30/2020	2:00:00 a, m,	16.6	92	0
11/30/2020	3:00:00 a, m,	16.4	92	0
11/30/2020	4:00:00 a, m,	16.6	93	0
11/30/2020	5:00:00 a, m,	16.6	92	0
11/30/2020	6:00:00 a, m,	16.3	95	0
11/30/2020	7:00:00 a, m,	16.4	96	0
11/30/2020	8:00:00 a, m,	17.1	96	0
11/30/2020	9:00:00 a, m,	17.7	96	0
11/30/2020	10:00:00 a, m	18.6	95	0
11/30/2020	11:00:00 a, m	19.3	96	0
11/30/2020	12:00:00 p, m	20	93	0
11/30/2020	1:00:00 p, m,	20.7	85	0
11/30/2020	2:00:00 p, m,	20.9	88	0
11/30/2020	3:00:00 p, m,	21.1	87	0
11/30/2020	4:00:00 p, m,	21.1	88	0
11/30/2020	5:00:00 p, m,	20.1	90	0
11/30/2020	6:00:00 p, m,	19.4	88	0
11/30/2020	7:00:00 p, m,	18.7	89	0
11/30/2020	8:00:00 p, m,	18.4	88	0
11/30/2020	9:00:00 p, m,	18.3	88	0
11/30/2020	10:00:00 p, m	18	90	0
11/30/2020	11:00:00 p, m	17.8	90	0
Nov_20		17.8913889	94.5541667	108
12/1/2020	12:00:00 a, m	17.7	91	0
12/1/2020	1:00:00 a, m,	17.7	90	0
12/1/2020	2:00:00 a, m,	17.9	91	0
12/1/2020	3:00:00 a, m,	17.8	91	0
12/1/2020	4:00:00 a, m,	17.8	92	0
12/1/2020	5:00:00 a, m,	17.8	93	0
12/1/2020	6:00:00 a, m,	17.7	92	0
12/1/2020	7:00:00 a, m,	17.6	93	0
12/1/2020	8:00:00 a, m,	17.5	94	0
12/1/2020	9:00:00 a, m,	18.1	95	0

12/1/2020	10:00:00 a, m	18.4	97	0
12/1/2020	11:00:00 a, m	18.8	95	0
12/1/2020	12:00:00 p, m	19.3	97	0.4
12/1/2020	1:00:00 p, m,	18.7	98	0.4
12/1/2020	2:00:00 p, m,	19.3	96	0
12/1/2020	3:00:00 p, m,	20.1	92	0
12/1/2020	4:00:00 p, m,	20	96	0
12/1/2020	5:00:00 p, m,	19.9	95	0
12/1/2020	6:00:00 p, m,	19.4	94	0
12/1/2020	7:00:00 p, m,	19.1	96	0
12/1/2020	8:00:00 p, m,	18.9	96	0.2
12/1/2020	9:00:00 p, m,	17.9	97	0.4
12/1/2020	10:00:00 p, m	16.7	98	0.2
12/1/2020	11:00:00 p, m	16.6	99	0.2
12/2/2020	12:00:00 a, m	16.8	98	0.2
12/2/2020	1:00:00 a, m,	16.4	98	0
12/2/2020	2:00:00 a, m,	16.4	99	0
12/2/2020	3:00:00 a, m,	16.3	98	0
12/2/2020	4:00:00 a, m,	16.1	98	0.2
12/2/2020	5:00:00 a, m,	15.8	98	0
12/2/2020	6:00:00 a, m,	15.8	98	0
12/2/2020	7:00:00 a, m,	16	99	0
12/2/2020	8:00:00 a, m,	16.4	99	0
12/2/2020	9:00:00 a, m,	16.8	99	0.2
12/2/2020	10:00:00 a, m	17.2	99	0
12/2/2020	11:00:00 a, m	17.7	99	0
12/2/2020	12:00:00 p, m	18.3	98	0
12/2/2020	1:00:00 p, m,	18.9	98	0
12/2/2020	2:00:00 p, m,	19.5	98	0
12/2/2020	3:00:00 p, m,	18.8	97	0
12/2/2020	4:00:00 p, m,	19.1	97	0
12/2/2020	5:00:00 p, m,	18.8	95	0.2
12/2/2020	6:00:00 p, m,	18	96	0
12/2/2020	7:00:00 p, m,	17.7	97	0
12/2/2020	8:00:00 p, m,	17.3	96	0
12/2/2020	9:00:00 p, m,	17.1	96	0
12/2/2020	10:00:00 p, m	17.1	95	0
12/2/2020	11:00:00 p, m	17.2	95	0
12/3/2020	12:00:00 a, m	17.1	94	0
12/3/2020	1:00:00 a, m,	17.1	96	0
12/3/2020	2:00:00 a, m,	17.3	96	0
12/3/2020	3:00:00 a, m,	17.1	96	0
12/3/2020	4:00:00 a, m,	17	95	0
12/3/2020	5:00:00 a, m,	16.9	96	0
12/3/2020	6:00:00 a, m,	16.9	97	0
12/3/2020	7:00:00 a, m,	17.2	97	0
12/3/2020	8:00:00 a, m,	17.4	97	0

12/3/2020	9:00:00 a, m,	17.9	98	0.2
12/3/2020	10:00:00 a, m	17.6	99	0
12/3/2020	11:00:00 a, m	18.1	99	0
12/3/2020	12:00:00 p, m	17.1	99	0
12/3/2020	1:00:00 p, m,	17.6	99	0
12/3/2020	2:00:00 p, m,	18.9	99	0
12/3/2020	3:00:00 p, m,	18.8	99	0
12/3/2020	4:00:00 p, m,	18.7	99	0
12/3/2020	5:00:00 p, m,	18.9	99	0
12/3/2020	6:00:00 p, m,	18.5	98	0
12/3/2020	7:00:00 p, m,	17.8	98	0
12/3/2020	8:00:00 p, m,	16.9	98	0
12/3/2020	9:00:00 p, m,	16.6	98	0
12/3/2020	10:00:00 p, m	16.7	98	0
12/3/2020	11:00:00 p, m	16.8	98	0
12/4/2020	12:00:00 a, m	16.7	98	0
12/4/2020	1:00:00 a, m,	16.4	97	0
12/4/2020	2:00:00 a, m,	16.2	97	0
12/4/2020	3:00:00 a, m,	15.9	97	0
12/4/2020	4:00:00 a, m,	15.8	97	0
12/4/2020	5:00:00 a, m,	15.9	97	0
12/4/2020	6:00:00 a, m,	15.6	97	0
12/4/2020	7:00:00 a, m,	16.1	98	0
12/4/2020	8:00:00 a, m,	16.3	97	0
12/4/2020	9:00:00 a, m,	17	99	0
12/4/2020	10:00:00 a, m	17.7	99	0
12/4/2020	11:00:00 a, m	17.8	99	0
12/4/2020	12:00:00 p, m	18.6	98	0
12/4/2020	1:00:00 p, m,	19.1	97	0
12/4/2020	2:00:00 p, m,	19.9	94	0
12/4/2020	3:00:00 p, m,	20.2	96	0
12/4/2020	4:00:00 p, m,	20.3	97	0
12/4/2020	5:00:00 p, m,	20.3	98	0
12/4/2020	6:00:00 p, m,	19.8	97	0
12/4/2020	7:00:00 p, m,	18.5	96	0
12/4/2020	8:00:00 p, m,	18.1	94	0
12/4/2020	9:00:00 p, m,	18	96	0
12/4/2020	10:00:00 p, m	17.9	96	0
12/4/2020	11:00:00 p, m	17.7	95	0
12/5/2020	12:00:00 a, m	17.8	95	0
12/5/2020	1:00:00 a, m,	17.5	95	0
12/5/2020	2:00:00 a, m,	17.7	96	0
12/5/2020	3:00:00 a, m,	17.6	95	0
12/5/2020	4:00:00 a, m,	17.4	94	0
12/5/2020	5:00:00 a, m,	17.2	95	0
12/5/2020	6:00:00 a, m,	17.2	96	0
12/5/2020	7:00:00 a, m,	17.1	97	0

12/5/2020	8:00:00 a, m,	17.3	97	0
12/5/2020	9:00:00 a, m,	17.9	98	0
12/5/2020	10:00:00 a, m	18.4	98	0
12/5/2020	11:00:00 a, m	19.1	96	0
12/5/2020	12:00:00 p, m	19.2	95	0
12/5/2020	1:00:00 p, m,	19.4	95	0
12/5/2020	2:00:00 p, m,	19.9	96	0
12/5/2020	3:00:00 p, m,	20.2	95	0
12/5/2020	4:00:00 p, m,	20.6	91	0
12/5/2020	5:00:00 p, m,	20.1	94	0
12/5/2020	6:00:00 p, m,	19.2	91	0
12/5/2020	7:00:00 p, m,	18.4	92	0
12/5/2020	8:00:00 p, m,	18.3	93	0
12/5/2020	9:00:00 p, m,	18.5	94	0
12/5/2020	10:00:00 p, m	18.2	88	0
12/5/2020	11:00:00 p, m	17.9	92	0
12/6/2020	12:00:00 a, m	17.4	88	0.2
12/6/2020	1:00:00 a, m,	17.3	90	0
12/6/2020	2:00:00 a, m,	17.6	91	0
12/6/2020	3:00:00 a, m,	17.6	91	0
12/6/2020	4:00:00 a, m,	17.6	90	0
12/6/2020	5:00:00 a, m,	17.1	92	0
12/6/2020	6:00:00 a, m,	16.9	91	0
12/6/2020	7:00:00 a, m,	17.1	93	0
12/6/2020	8:00:00 a, m,	17.6	94	0
12/6/2020	9:00:00 a, m,	18.3	94	0
12/6/2020	10:00:00 a, m	18.8	93	0
12/6/2020	11:00:00 a, m	19.4	92	0
12/6/2020	12:00:00 p, m	19.4	90	0
12/6/2020	1:00:00 p, m,	19.7	93	0
12/6/2020	2:00:00 p, m,	20.9	89	0
12/6/2020	3:00:00 p, m,	20.7	88	0
12/6/2020	4:00:00 p, m,	20.1	89	0
12/6/2020	5:00:00 p, m,	19.7	91	0
12/6/2020	6:00:00 p, m,	18.9	93	0
12/6/2020	7:00:00 p, m,	18.3	88	0
12/6/2020	8:00:00 p, m,	17.9	89	0
12/6/2020	9:00:00 p, m,	18.3	87	0
12/6/2020	10:00:00 p, m	18.7	83	0
12/6/2020	11:00:00 p, m	18.6	84	0
12/7/2020	12:00:00 a, m	17.8	86	0
12/7/2020	1:00:00 a, m,	17.7	85	0
12/7/2020	2:00:00 a, m,	17.5	88	0
12/7/2020	3:00:00 a, m,	17.3	87	0
12/7/2020	4:00:00 a, m,	16.9	87	0
12/7/2020	5:00:00 a, m,	16.8	88	0
12/7/2020	6:00:00 a, m,	16.8	89	0

12/7/2020	7:00:00 a, m,	17.3	90	0
12/7/2020	8:00:00 a, m,	17.5	90	0
12/7/2020	9:00:00 a, m,	18.1	90	0
12/7/2020	10:00:00 a, m	18.7	91	0
12/7/2020	11:00:00 a, m	19.1	90	0
12/7/2020	12:00:00 p, m	19.2	90	0
12/7/2020	1:00:00 p, m,	20	89	0
12/7/2020	2:00:00 p, m,	20.7	88	0
12/7/2020	3:00:00 p, m,	20.5	88	0
12/7/2020	4:00:00 p, m,	20.1	90	0
12/7/2020	5:00:00 p, m,	19.8	90	0
12/7/2020	6:00:00 p, m,	19.3	89	0
12/7/2020	7:00:00 p, m,	18.7	90	0
12/7/2020	8:00:00 p, m,	18.4	91	0
12/7/2020	9:00:00 p, m,	18.3	90	0
12/7/2020	10:00:00 p, m	18.2	89	0
12/7/2020	11:00:00 p, m	18.2	90	0
12/8/2020	12:00:00 a, m	18.2	90	0
12/8/2020	1:00:00 a, m,	17.8	91	0
12/8/2020	2:00:00 a, m,	17.6	93	0
12/8/2020	3:00:00 a, m,	17.6	93	0
12/8/2020	4:00:00 a, m,	17.3	94	0
12/8/2020	5:00:00 a, m,	17	94	0
12/8/2020	6:00:00 a, m,	16.9	95	0
12/8/2020	7:00:00 a, m,	16.9	95	0
12/8/2020	8:00:00 a, m,	17.1	95	0
12/8/2020	9:00:00 a, m,	17.6	97	0
12/8/2020	10:00:00 a, m	18.2	96	0
12/8/2020	11:00:00 a, m	18.7	96	0
12/8/2020	12:00:00 p, m	19.1	93	3.2
12/8/2020	1:00:00 p, m,	19.5	91	0
12/8/2020	2:00:00 p, m,	19.4	94	0
12/8/2020	3:00:00 p, m,	19.8	93	0
12/8/2020	4:00:00 p, m,	19.8	94	0
12/8/2020	5:00:00 p, m,	19.5	92	0
12/8/2020	6:00:00 p, m,	19.1	95	0
12/8/2020	7:00:00 p, m,	18.6	94	0
12/8/2020	8:00:00 p, m,	18.3	92	0
12/8/2020	9:00:00 p, m,	18.1	90	0
12/8/2020	10:00:00 p, m	17.8	91	0
12/8/2020	11:00:00 p, m	17.5	91	0
12/9/2020	12:00:00 a, m	17.1	91	0
12/9/2020	1:00:00 a, m,	17.2	93	0
12/9/2020	2:00:00 a, m,	16.9	93	0
12/9/2020	3:00:00 a, m,	16.7	93	0
12/9/2020	4:00:00 a, m,	16.4	93	0
12/9/2020	5:00:00 a, m,	16.4	95	0

12/9/2020	6:00:00 a, m,	16	93	0
12/9/2020	7:00:00 a, m,	16.2	93	0
12/9/2020	8:00:00 a, m,	16.7	94	0
12/9/2020	9:00:00 a, m,	17.6	96	0
12/9/2020	10:00:00 a, m	18.3	96	0
12/9/2020	11:00:00 a, m	19.1	95	0
12/9/2020	12:00:00 p, m	19.2	97	0.8
12/9/2020	1:00:00 p, m,	18.2	95	6
12/9/2020	2:00:00 p, m,	18.5	99	1
12/9/2020	3:00:00 p, m,	19.1	99	0.2
12/9/2020	4:00:00 p, m,	19.1	97	0
12/9/2020	5:00:00 p, m,	19.1	98	0.4
12/9/2020	6:00:00 p, m,	19	97	0
12/9/2020	7:00:00 p, m,	17.6	98	0
12/9/2020	8:00:00 p, m,	17.4	98	0
12/9/2020	9:00:00 p, m,	16.8	97	0
12/9/2020	10:00:00 p, m	16.5	97	0
12/9/2020	11:00:00 p, m	16.3	96	0
12/10/2020	12:00:00 a, m	16.7	98	0
12/10/2020	1:00:00 a, m,	16.4	96	0
12/10/2020	2:00:00 a, m,	16.3	97	0
12/10/2020	3:00:00 a, m,	16.4	96	0
12/10/2020	4:00:00 a, m,	16.2	96	0
12/10/2020	5:00:00 a, m,	15.9	97	0
12/10/2020	6:00:00 a, m,	15.9	96	0
12/10/2020	7:00:00 a, m,	16.3	97	0
12/10/2020	8:00:00 a, m,	16.2	95	0
12/10/2020	9:00:00 a, m,	16.4	96	0
12/10/2020	10:00:00 a, m	17.5	98	0
12/10/2020	11:00:00 a, m	18.4	98	0
12/10/2020	12:00:00 p, m	18.6	99	0.2
12/10/2020	1:00:00 p, m,	18.8	98	0.4
12/10/2020	2:00:00 p, m,	19.2	98	0
12/10/2020	3:00:00 p, m,	19.5	98	0
12/10/2020	4:00:00 p, m,	19.2	98	0.6
12/10/2020	5:00:00 p, m,	19.3	99	0
12/10/2020	6:00:00 p, m,	18.7	98	0.4
12/10/2020	7:00:00 p, m,	18	98	0
12/10/2020	8:00:00 p, m,	17.7	98	0
12/10/2020	9:00:00 p, m,	17.2	98	0
12/10/2020	10:00:00 p, m	17.4	97	0
12/10/2020	11:00:00 p, m	17.1	97	0
12/11/2020	12:00:00 a, m	16.8	96	0
12/11/2020	1:00:00 a, m,	17	97	0
12/11/2020	2:00:00 a, m,	16.8	97	0
12/11/2020	3:00:00 a, m,	16.6	97	0
12/11/2020	4:00:00 a, m,	16.6	95	0

12/11/2020	5:00:00 a, m,	16.6	97	0
12/11/2020	6:00:00 a, m,	16.3	97	0
12/11/2020	7:00:00 a, m,	16.5	97	0
12/11/2020	8:00:00 a, m,	16.8	98	0
12/11/2020	9:00:00 a, m,	17.7	98	0
12/11/2020	10:00:00 a, m	18.4	98	0
12/11/2020	11:00:00 a, m	18.4	98	0
12/11/2020	12:00:00 p, m	19	98	0
12/11/2020	1:00:00 p, m,	19.9	96	0
12/11/2020	2:00:00 p, m,	19.8	96	0.2
12/11/2020	3:00:00 p, m,	20.3	97	0.2
12/11/2020	4:00:00 p, m,	20.2	99	0
12/11/2020	5:00:00 p, m,	20.2	98	0
12/11/2020	6:00:00 p, m,	19.2	96	0
12/11/2020	7:00:00 p, m,	18.7	97	0
12/11/2020	8:00:00 p, m,	18.8	97	0
12/11/2020	9:00:00 p, m,	18.7	97	0
12/11/2020	10:00:00 p, m	18.2	96	0
12/11/2020	11:00:00 p, m	18.2	96	0
12/12/2020	12:00:00 a, m	17.7	96	0
12/12/2020	1:00:00 a, m,	17.8	96	0
12/12/2020	2:00:00 a, m,	17.6	96	0
12/12/2020	3:00:00 a, m,	17.3	96	0
12/12/2020	4:00:00 a, m,	17.2	95	0
12/12/2020	5:00:00 a, m,	16.7	96	0
12/12/2020	6:00:00 a, m,	16.8	96	0
12/12/2020	7:00:00 a, m,	17.2	97	0
12/12/2020	8:00:00 a, m,	17.6	97	0
12/12/2020	9:00:00 a, m,	18.2	98	0
12/12/2020	10:00:00 a, m	18.5	98	0
12/12/2020	11:00:00 a, m	18.7	97	0
12/12/2020	12:00:00 p, m	19.1	96	0
12/12/2020	1:00:00 p, m,	19.5	96	0
12/12/2020	2:00:00 p, m,	20.6	95	0
12/12/2020	3:00:00 p, m,	19.3	93	3.4
12/12/2020	4:00:00 p, m,	17.8	98	0.8
12/12/2020	5:00:00 p, m,	18.4	99	0
12/12/2020	6:00:00 p, m,	18.2	96	0.2
12/12/2020	7:00:00 p, m,	17.7	98	0
12/12/2020	8:00:00 p, m,	18.2	98	0
12/12/2020	9:00:00 p, m,	18.2	96	0
12/12/2020	10:00:00 p, m	17.5	97	0
12/12/2020	11:00:00 p, m	17.4	97	0
12/13/2020	12:00:00 a, m	17.4	97	0
12/13/2020	1:00:00 a, m,	16.9	96	0
12/13/2020	2:00:00 a, m,	16.7	96	0
12/13/2020	3:00:00 a, m,	16.7	95	0

12/13/2020	4:00:00 a, m,	16.7	96	0
12/13/2020	5:00:00 a, m,	16.4	96	0
12/13/2020	6:00:00 a, m,	16.2	95	0
12/13/2020	7:00:00 a, m,	16.2	95	0
12/13/2020	8:00:00 a, m,	16.8	97	0
12/13/2020	9:00:00 a, m,	17.4	98	0
12/13/2020	10:00:00 a, m	18.1	97	0
12/13/2020	11:00:00 a, m	18.8	96	0
12/13/2020	12:00:00 p, m	19.2	96	0
12/13/2020	1:00:00 p, m,	20	95	0
12/13/2020	2:00:00 p, m,	19.9	95	0
12/13/2020	3:00:00 p, m,	20.3	93	0
12/13/2020	4:00:00 p, m,	20.3	92	0
12/13/2020	5:00:00 p, m,	19.9	93	0
12/13/2020	6:00:00 p, m,	18.9	92	0
12/13/2020	7:00:00 p, m,	18.1	90	0
12/13/2020	8:00:00 p, m,	17.7	89	0
12/13/2020	9:00:00 p, m,	17.3	88	0
12/13/2020	10:00:00 p, m	17.2	88	0
12/13/2020	11:00:00 p, m	17	90	0
12/14/2020	12:00:00 a, m	16.9	92	0
12/14/2020	1:00:00 a, m,	16.8	92	0
12/14/2020	2:00:00 a, m,	16.7	92	0
12/14/2020	3:00:00 a, m,	16.7	94	0
12/14/2020	4:00:00 a, m,	16.4	92	0
12/14/2020	5:00:00 a, m,	16.2	91	0
12/14/2020	6:00:00 a, m,	16.2	93	0
12/14/2020	7:00:00 a, m,	16.4	95	0
12/14/2020	8:00:00 a, m,	16.9	94	0
12/14/2020	9:00:00 a, m,	17.3	95	0
12/14/2020	10:00:00 a, m	18.2	95	0
12/14/2020	11:00:00 a, m	18.8	93	0
12/14/2020	12:00:00 p, m	19.8	91	0
12/14/2020	1:00:00 p, m,	20.7	88	0
12/14/2020	2:00:00 p, m,	19.9	89	0
12/14/2020	3:00:00 p, m,	19.9	90	0
12/14/2020	4:00:00 p, m,	20.2	90	0
12/14/2020	5:00:00 p, m,	20.1	89	0
12/14/2020	6:00:00 p, m,	19.5	88	0
12/14/2020	7:00:00 p, m,	18.7	88	0
12/14/2020	8:00:00 p, m,	18.2	87	0
12/14/2020	9:00:00 p, m,	17.8	88	0
12/14/2020	10:00:00 p, m	17.6	88	0
12/14/2020	11:00:00 p, m	17.4	88	0
12/15/2020	12:00:00 a, m	17.1	88	0
12/15/2020	1:00:00 a, m,	16.8	89	0
12/15/2020	2:00:00 a, m,	17	92	0

12/15/2020	3:00:00 a, m,	16.8	90	0
12/15/2020	4:00:00 a, m,	16.5	91	0
12/15/2020	5:00:00 a, m,	16.5	94	0
12/15/2020	6:00:00 a, m,	16.6	94	0
12/15/2020	7:00:00 a, m,	16.8	93	0
12/15/2020	8:00:00 a, m,	17.2	95	0
12/15/2020	9:00:00 a, m,	17.7	95	0
12/15/2020	10:00:00 a, m	18	97	0
12/15/2020	11:00:00 a, m	18.1	96	0
12/15/2020	12:00:00 p, m	18.6	96	0
12/15/2020	1:00:00 p, m,	19.1	96	0
12/15/2020	2:00:00 p, m,	19.7	95	0
12/15/2020	3:00:00 p, m,	20.4	93	0
12/15/2020	4:00:00 p, m,	20.7	92	0
12/15/2020	5:00:00 p, m,	18.2	93	3.2
12/15/2020	6:00:00 p, m,	16.7	97	4.8
12/15/2020	7:00:00 p, m,	16.8	98	0
12/15/2020	8:00:00 p, m,	16.8	97	0
12/15/2020	9:00:00 p, m,	16.6	97	0
12/15/2020	10:00:00 p, m	16.2	95	0
12/15/2020	11:00:00 p, m	16.7	97	0
12/16/2020	12:00:00 a, m	16.6	96	0
12/16/2020	1:00:00 a, m,	16.6	96	0
12/16/2020	2:00:00 a, m,	16.2	96	0
12/16/2020	3:00:00 a, m,	16	95	0
12/16/2020	4:00:00 a, m,	16.3	95	0
12/16/2020	5:00:00 a, m,	15.8	94	0
12/16/2020	6:00:00 a, m,	15.6	95	0
12/16/2020	7:00:00 a, m,	15.7	96	0
12/16/2020	8:00:00 a, m,	16.4	97	0
12/16/2020	9:00:00 a, m,	17.1	98	0
12/16/2020	10:00:00 a, m	17.8	98	0
12/16/2020	11:00:00 a, m	18.3	97	0
12/16/2020	12:00:00 p, m	18.7	96	0
12/16/2020	1:00:00 p, m,	19.4	94	0
12/16/2020	2:00:00 p, m,	19.7	95	0
12/16/2020	3:00:00 p, m,	20.1	94	0
12/16/2020	4:00:00 p, m,	20.4	94	0
12/16/2020	5:00:00 p, m,	20.2	96	0
12/16/2020	6:00:00 p, m,	19.7	94	0.2
12/16/2020	7:00:00 p, m,	18.7	95	0
12/16/2020	8:00:00 p, m,	18.2	94	0
12/16/2020	9:00:00 p, m,	17.8	93	0
12/16/2020	10:00:00 p, m	17.6	93	0
12/16/2020	11:00:00 p, m	17.4	94	0
12/17/2020	12:00:00 a, m	17.3	93	0
12/17/2020	1:00:00 a, m,	16.9	93	0

12/17/2020	2:00:00 a, m,	16.9	95	0
12/17/2020	3:00:00 a, m,	16.7	94	0
12/17/2020	4:00:00 a, m,	16.7	96	0
12/17/2020	5:00:00 a, m,	16.7	96	0
12/17/2020	6:00:00 a, m,	16.5	96	0
12/17/2020	7:00:00 a, m,	16.3	95	0
12/17/2020	8:00:00 a, m,	17.1	96	0
12/17/2020	9:00:00 a, m,	17.4	97	0
12/17/2020	10:00:00 a, m	18.1	98	0
12/17/2020	11:00:00 a, m	18.7	96	0
12/17/2020	12:00:00 p, m	19.4	92	0
12/17/2020	1:00:00 p, m,	19.7	94	0
12/17/2020	2:00:00 p, m,	20.1	91	0
12/17/2020	3:00:00 p, m,	20.8	90	0
12/17/2020	4:00:00 p, m,	18.6	97	2
12/17/2020	5:00:00 p, m,	18.2	98	0
12/17/2020	6:00:00 p, m,	17.5	97	0
12/17/2020	7:00:00 p, m,	17.3	96	0
12/17/2020	8:00:00 p, m,	17.2	96	0
12/17/2020	9:00:00 p, m,	17	95	0
12/17/2020	10:00:00 p, m	16.9	96	0
12/17/2020	11:00:00 p, m	16.9	96	0
12/18/2020	12:00:00 a, m	16.9	96	0
12/18/2020	1:00:00 a, m,	17	96	0
12/18/2020	2:00:00 a, m,	16.8	95	0
12/18/2020	3:00:00 a, m,	16.8	95	0
12/18/2020	4:00:00 a, m,	16.8	95	0
12/18/2020	5:00:00 a, m,	16.8	96	0
12/18/2020	6:00:00 a, m,	16.8	95	0
12/18/2020	7:00:00 a, m,	17	95	0
12/18/2020	8:00:00 a, m,	17.4	95	0
12/18/2020	9:00:00 a, m,	18.1	96	0
12/18/2020	10:00:00 a, m	18.4	96	0
12/18/2020	11:00:00 a, m	18.8	94	0
12/18/2020	12:00:00 p, m	19.5	93	0
12/18/2020	1:00:00 p, m,	20	91	0
12/18/2020	2:00:00 p, m,	20.2	91	0
12/18/2020	3:00:00 p, m,	20.4	91	0
12/18/2020	4:00:00 p, m,	20.6	89	0
12/18/2020	5:00:00 p, m,	20.1	88	0
12/18/2020	6:00:00 p, m,	18.8	89	0
12/18/2020	7:00:00 p, m,	18.3	91	0
12/18/2020	8:00:00 p, m,	18.1	91	0
12/18/2020	9:00:00 p, m,	18.1	91	0
12/18/2020	10:00:00 p, m	17.8	89	0
12/18/2020	11:00:00 p, m	17.7	91	0
12/19/2020	12:00:00 a, m	17.6	92	0

12/19/2020	1:00:00 a, m,	17.5	92	0
12/19/2020	2:00:00 a, m,	17.5	93	0
12/19/2020	3:00:00 a, m,	17.6	94	0
12/19/2020	4:00:00 a, m,	17.4	95	0
12/19/2020	5:00:00 a, m,	17.1	96	0
12/19/2020	6:00:00 a, m,	17.1	95	0
12/19/2020	7:00:00 a, m,	17.2	96	0
12/19/2020	8:00:00 a, m,	17.7	96	0
12/19/2020	9:00:00 a, m,	18.1	95	0
12/19/2020	10:00:00 a, m	18.1	95	0
12/19/2020	11:00:00 a, m	19.1	94	0
12/19/2020	12:00:00 p, m	19.2	91	0
12/19/2020	1:00:00 p, m,	19.7	91	0
12/19/2020	2:00:00 p, m,	19.4	90	0.2
12/19/2020	3:00:00 p, m,	18.8	97	0
12/19/2020	4:00:00 p, m,	19.7	98	0
12/19/2020	5:00:00 p, m,	19.6	96	0
12/19/2020	6:00:00 p, m,	18.4	97	4.4
12/19/2020	7:00:00 p, m,	17.5	98	0
12/19/2020	8:00:00 p, m,	17.1	98	0
12/19/2020	9:00:00 p, m,	17.2	97	0
12/19/2020	10:00:00 p, m	17.1	97	0
12/19/2020	11:00:00 p, m	16.8	96	0
12/20/2020	12:00:00 a, m	16.9	97	0
12/20/2020	1:00:00 a, m,	17	96	0
12/20/2020	2:00:00 a, m,	16.8	97	0
12/20/2020	3:00:00 a, m,	16.9	97	0
12/20/2020	4:00:00 a, m,	16.4	96	0
12/20/2020	5:00:00 a, m,	16	97	0
12/20/2020	6:00:00 a, m,	16.1	96	0
12/20/2020	7:00:00 a, m,	16	96	0
12/20/2020	8:00:00 a, m,	16.6	98	0
12/20/2020	9:00:00 a, m,	17.1	97	0
12/20/2020	10:00:00 a, m	17.8	98	0
12/20/2020	11:00:00 a, m	18.3	98	0
12/20/2020	12:00:00 p, m	19.3	97	0
12/20/2020	1:00:00 p, m,	20.1	95	0
12/20/2020	2:00:00 p, m,	20	94	0
12/20/2020	3:00:00 p, m,	19.7	95	0
12/20/2020	4:00:00 p, m,	19.9	96	0.2
12/20/2020	5:00:00 p, m,	20.1	96	0
12/20/2020	6:00:00 p, m,	19.2	92	0
12/20/2020	7:00:00 p, m,	17.9	90	0
12/20/2020	8:00:00 p, m,	17.6	92	0
12/20/2020	9:00:00 p, m,	17.7	93	0
12/20/2020	10:00:00 p, m	17.4	92	0
12/20/2020	11:00:00 p, m	17	91	0

12/21/2020	12:00:00 a, m	17.1	94	0
12/21/2020	1:00:00 a, m,	16.9	93	0
12/21/2020	2:00:00 a, m,	16.7	93	0
12/21/2020	3:00:00 a, m,	16.4	94	0
12/21/2020	4:00:00 a, m,	16.3	94	0
12/21/2020	5:00:00 a, m,	15.9	94	0
12/21/2020	6:00:00 a, m,	15.7	93	0
12/21/2020	7:00:00 a, m,	15.7	94	0
12/21/2020	8:00:00 a, m,	16.3	93	0
12/21/2020	9:00:00 a, m,	16.7	95	0
12/21/2020	10:00:00 a, m	17.6	97	0
12/21/2020	11:00:00 a, m	18.7	96	0
12/21/2020	12:00:00 p, m	19.7	94	0
12/21/2020	1:00:00 p, m,	19.8	94	0
12/21/2020	2:00:00 p, m,	20	92	0
12/21/2020	3:00:00 p, m,	20.5	92	0
12/21/2020	4:00:00 p, m,	20.8	92	0
12/21/2020	5:00:00 p, m,	20.6	93	0
12/21/2020	6:00:00 p, m,	20	94	0
12/21/2020	7:00:00 p, m,	19.1	94	0
12/21/2020	8:00:00 p, m,	18.6	94	0
12/21/2020	9:00:00 p, m,	18.3	92	0
12/21/2020	10:00:00 p, m	18.2	95	0
12/21/2020	11:00:00 p, m	18.2	92	0
12/22/2020	12:00:00 a, m	18.4	94	0
12/22/2020	1:00:00 a, m,	18.1	94	0
12/22/2020	2:00:00 a, m,	17.8	96	0
12/22/2020	3:00:00 a, m,	17.4	95	0
12/22/2020	4:00:00 a, m,	16.8	96	0
12/22/2020	5:00:00 a, m,	16.7	95	0
12/22/2020	6:00:00 a, m,	16.5	95	0
12/22/2020	7:00:00 a, m,	16.8	97	0
12/22/2020	8:00:00 a, m,	17.3	95	0
12/22/2020	9:00:00 a, m,	17.7	97	0
12/22/2020	10:00:00 a, m	18.2	97	0
12/22/2020	11:00:00 a, m	18.6	97	0
12/22/2020	12:00:00 p, m	18.8	96	0
12/22/2020	1:00:00 p, m,	19.9	95	0
12/22/2020	2:00:00 p, m,	19.6	95	0
12/22/2020	3:00:00 p, m,	19.7	96	0
12/22/2020	4:00:00 p, m,	19.7	95	0.8
12/22/2020	5:00:00 p, m,	18.6	98	0
12/22/2020	6:00:00 p, m,	17.8	97	0
12/22/2020	7:00:00 p, m,	17.6	96	0
12/22/2020	8:00:00 p, m,	17.4	96	0
12/22/2020	9:00:00 p, m,	17.5	95	0
12/22/2020	10:00:00 p, m	17.2	95	0

12/22/2020	11:00:00 p, m	17.2	94	0
12/23/2020	12:00:00 a, m	17.3	94	0
12/23/2020	1:00:00 a, m,	17.1	95	0
12/23/2020	2:00:00 a, m,	17.1	95	0
12/23/2020	3:00:00 a, m,	16.9	94	0
12/23/2020	4:00:00 a, m,	16.8	95	0
12/23/2020	5:00:00 a, m,	16.4	95	0
12/23/2020	6:00:00 a, m,	16.2	94	0
12/23/2020	7:00:00 a, m,	16.3	95	0
12/23/2020	8:00:00 a, m,	16.6	95	0
12/23/2020	9:00:00 a, m,	17.2	97	0
12/23/2020	10:00:00 a, m	17.7	98	0
12/23/2020	11:00:00 a, m	18.3	98	0
12/23/2020	12:00:00 p, m	18.9	97	0
12/23/2020	1:00:00 p, m,	20	94	0
12/23/2020	2:00:00 p, m,	20.5	93	0
12/23/2020	3:00:00 p, m,	20.3	94	0.6
12/23/2020	4:00:00 p, m,	19.4	99	1
12/23/2020	5:00:00 p, m,	18.8	92	0.2
12/23/2020	6:00:00 p, m,	18.2	95	0
12/23/2020	7:00:00 p, m,	18.2	96	0
12/23/2020	8:00:00 p, m,	17.9	95	0
12/23/2020	9:00:00 p, m,	17.7	94	0
12/23/2020	10:00:00 p, m	17.8	95	0
12/23/2020	11:00:00 p, m	17.8	94	0
12/24/2020	12:00:00 a, m	17.6	94	0
12/24/2020	1:00:00 a, m,	17.5	95	0
12/24/2020	2:00:00 a, m,	17.3	93	0
12/24/2020	3:00:00 a, m,	17.3	98	0.4
12/24/2020	4:00:00 a, m,	16.7	97	0
12/24/2020	5:00:00 a, m,	16.4	97	0
12/24/2020	6:00:00 a, m,	16.7	97	0
12/24/2020	7:00:00 a, m,	17.1	96	0
12/24/2020	8:00:00 a, m,	17.2	97	0
12/24/2020	9:00:00 a, m,	17.6	98	0
12/24/2020	10:00:00 a, m	18.3	98	0
12/24/2020	11:00:00 a, m	18.4	98	0
12/24/2020	12:00:00 p, m	18.8	97	0
12/24/2020	1:00:00 p, m,	18.7	98	3.4
12/24/2020	2:00:00 p, m,	18.7	99	0.2
12/24/2020	3:00:00 p, m,	19.2	99	0
12/24/2020	4:00:00 p, m,	19.1	98	0
12/24/2020	5:00:00 p, m,	19.7	99	0.4
12/24/2020	6:00:00 p, m,	18.8	97	0.2
12/24/2020	7:00:00 p, m,	17.9	98	0.6
12/24/2020	8:00:00 p, m,	17.3	97	0
12/24/2020	9:00:00 p, m,	17.2	98	0

12/24/2020	10:00:00 p, m	17.3	97	0
12/24/2020	11:00:00 p, m	17.3	98	0
12/25/2020	12:00:00 a, m	16.9	97	0
12/25/2020	1:00:00 a, m,	16.8	97	0
12/25/2020	2:00:00 a, m,	17.1	97	0
12/25/2020	3:00:00 a, m,	16.7	97	0
12/25/2020	4:00:00 a, m,	16.6	97	0
12/25/2020	5:00:00 a, m,	16.6	97	0
12/25/2020	6:00:00 a, m,	16.4	97	0
12/25/2020	7:00:00 a, m,	16.8	98	0
12/25/2020	8:00:00 a, m,	17.3	98	0
12/25/2020	9:00:00 a, m,	17.7	98	0
12/25/2020	10:00:00 a, m	17.6	99	0
12/25/2020	11:00:00 a, m	18.1	99	0
12/25/2020	12:00:00 p, m	17.7	98	0
12/25/2020	1:00:00 p, m,	18.6	98	0
12/25/2020	2:00:00 p, m,	19	97	0
12/25/2020	3:00:00 p, m,	18.6	98	0
12/25/2020	4:00:00 p, m,	18.6	99	0
12/25/2020	5:00:00 p, m,	18.4	99	0
12/25/2020	6:00:00 p, m,	18.1	98	0
12/25/2020	7:00:00 p, m,	17.5	97	0
12/25/2020	8:00:00 p, m,	17.2	97	0
12/25/2020	9:00:00 p, m,	17	96	0
12/25/2020	10:00:00 p, m	16.9	96	0
12/25/2020	11:00:00 p, m	17.2	96	0
12/26/2020	12:00:00 a, m	15.8	98	11
12/26/2020	1:00:00 a, m,	15.7	99	1
12/26/2020	2:00:00 a, m,	15.9	99	2
12/26/2020	3:00:00 a, m,	16.1	99	0.2
12/26/2020	4:00:00 a, m,	16.1	99	3.2
12/26/2020	5:00:00 a, m,	15.7	99	0.2
12/26/2020	6:00:00 a, m,	15.6	99	1.4
12/26/2020	7:00:00 a, m,	15.6	99	1.2
12/26/2020	8:00:00 a, m,	15.9	99	0.2
12/26/2020	9:00:00 a, m,	16.5	99	0
12/26/2020	10:00:00 a, m	16.9	100	0
12/26/2020	11:00:00 a, m	17.2	99	0
12/26/2020	12:00:00 p, m	17.3	100	0
12/26/2020	1:00:00 p, m,	17.7	100	0
12/26/2020	2:00:00 p, m,	17.9	100	0
12/26/2020	3:00:00 p, m,	17.8	99	2.2
12/26/2020	4:00:00 p, m,	17.9	99	2.2
12/26/2020	5:00:00 p, m,	17.7	99	3.8
12/26/2020	6:00:00 p, m,	17.3	99	0.4
12/26/2020	7:00:00 p, m,	16.8	99	0
12/26/2020	8:00:00 p, m,	16.7	99	0

12/26/2020	9:00:00 p, m,	16.9	99	0
12/26/2020	10:00:00 p, m	17	99	0.2
12/26/2020	11:00:00 p, m	17	99	0
12/27/2020	12:00:00 a, m	16.8	99	0
12/27/2020	1:00:00 a, m,	16.8	99	0
12/27/2020	2:00:00 a, m,	16.2	99	0
12/27/2020	3:00:00 a, m,	16.3	99	0
12/27/2020	4:00:00 a, m,	16.4	98	0
12/27/2020	5:00:00 a, m,	16.5	98	0
12/27/2020	6:00:00 a, m,	16.4	99	0
12/27/2020	7:00:00 a, m,	16.6	99	0.2
12/27/2020	8:00:00 a, m,	16.3	99	0.2
12/27/2020	9:00:00 a, m,	16.2	99	0
12/27/2020	10:00:00 a, m	16.6	99	0
12/27/2020	11:00:00 a, m	17.2	99	0
12/27/2020	12:00:00 p, m	17.6	99	0
12/27/2020	1:00:00 p, m,	17.7	99	0
12/27/2020	2:00:00 p, m,	17.7	99	0
12/27/2020	3:00:00 p, m,	17.7	99	0
12/27/2020	4:00:00 p, m,	17.6	99	0
12/27/2020	5:00:00 p, m,	17.5	99	3.4
12/27/2020	6:00:00 p, m,	17.5	99	1
12/27/2020	7:00:00 p, m,	16.9	99	0.2
12/27/2020	8:00:00 p, m,	16.4	99	0
12/27/2020	9:00:00 p, m,	16.4	99	0
12/27/2020	10:00:00 p, m	16.3	99	0
12/27/2020	11:00:00 p, m	16.6	99	0
12/28/2020	12:00:00 a, m	16.6	99	0.2
12/28/2020	1:00:00 a, m,	16.8	99	0
12/28/2020	2:00:00 a, m,	16.5	99	0
12/28/2020	3:00:00 a, m,	16.1	99	0
12/28/2020	4:00:00 a, m,	15.9	99	0
12/28/2020	5:00:00 a, m,	16.1	99	0
12/28/2020	6:00:00 a, m,	16.2	99	0.2
12/28/2020	7:00:00 a, m,	16.1	99	0
12/28/2020	8:00:00 a, m,	16.3	99	0.2
12/28/2020	9:00:00 a, m,	16.2	99	0.2
12/28/2020	10:00:00 a, m	17.1	100	0
12/28/2020	11:00:00 a, m	17.2	99	0
12/28/2020	12:00:00 p, m	17.3	100	0.2
12/28/2020	1:00:00 p, m,	17.9	100	0
12/28/2020	2:00:00 p, m,	17.8	99	0
12/28/2020	3:00:00 p, m,	18.2	99	0
12/28/2020	4:00:00 p, m,	17.8	99	0
12/28/2020	5:00:00 p, m,	17.7	99	0
12/28/2020	6:00:00 p, m,	17.4	98	0
12/28/2020	7:00:00 p, m,	16.8	98	0

12/28/2020	8:00:00 p, m,	16.4	98	0
12/28/2020	9:00:00 p, m,	16.3	98	0
12/28/2020	10:00:00 p, m	16.3	98	0
12/28/2020	11:00:00 p, m	16.1	98	0
12/29/2020	12:00:00 a, m	16.2	98	0
12/29/2020	1:00:00 a, m,	16.3	99	0
12/29/2020	2:00:00 a, m,	15.8	98	0
12/29/2020	3:00:00 a, m,	15.6	98	0
12/29/2020	4:00:00 a, m,	15.5	97	0
12/29/2020	5:00:00 a, m,	15.7	98	0
12/29/2020	6:00:00 a, m,	15.8	98	0
12/29/2020	7:00:00 a, m,	15.8	98	0
12/29/2020	8:00:00 a, m,	15.8	98	0
12/29/2020	9:00:00 a, m,	16	99	1.8
12/29/2020	10:00:00 a, m	16.6	100	0
12/29/2020	11:00:00 a, m	16.6	100	1.4
12/29/2020	12:00:00 p, m	17.2	99	0.6
12/29/2020	1:00:00 p, m,	17.7	100	0
12/29/2020	2:00:00 p, m,	18.2	99	0
12/29/2020	3:00:00 p, m,	19.2	99	0
12/29/2020	4:00:00 p, m,	18.7	98	0
12/29/2020	5:00:00 p, m,	18.8	99	0
12/29/2020	6:00:00 p, m,	18.1	98	5.2
12/29/2020	7:00:00 p, m,	17.4	99	0
12/29/2020	8:00:00 p, m,	17.3	99	0
12/29/2020	9:00:00 p, m,	17.2	99	0
12/29/2020	10:00:00 p, m	16.9	99	0
12/29/2020	11:00:00 p, m	16.8	99	0
12/30/2020	12:00:00 a, m	16.9	99	0.2
12/30/2020	1:00:00 a, m,	16.7	99	0
12/30/2020	2:00:00 a, m,	16.7	99	0
12/30/2020	3:00:00 a, m,	16.5	99	0
12/30/2020	4:00:00 a, m,	16.1	99	0
12/30/2020	5:00:00 a, m,	16.2	99	0
12/30/2020	6:00:00 a, m,	16.1	99	0
12/30/2020	7:00:00 a, m,	16.2	99	0
12/30/2020	8:00:00 a, m,	16.2	99	0
12/30/2020	9:00:00 a, m,	16.5	99	0
12/30/2020	10:00:00 a, m	17.5	100	0
12/30/2020	11:00:00 a, m	17.6	100	0
12/30/2020	12:00:00 p, m	17.4	99	0
12/30/2020	1:00:00 p, m,	17.8	100	0
12/30/2020	2:00:00 p, m,	18.1	99	0
12/30/2020	3:00:00 p, m,	18.7	99	0
12/30/2020	4:00:00 p, m,	17.6	98	1.2
12/30/2020	5:00:00 p, m,	17.2	99	0
12/30/2020	6:00:00 p, m,	17.2	99	0

12/30/2020	7:00:00 p, m,	17	99	0
12/30/2020	8:00:00 p, m,	17	98	0
12/30/2020	9:00:00 p, m,	16.8	98	0
12/30/2020	10:00:00 p, m	16.9	98	0
12/30/2020	11:00:00 p, m	16.9	99	0
12/31/2020	12:00:00 a, m	16.9	98	0
12/31/2020	1:00:00 a, m,	17	98	0
12/31/2020	2:00:00 a, m,	16.7	98	0
12/31/2020	3:00:00 a, m,	16.8	99	0
12/31/2020	4:00:00 a, m,	16.6	98	0
12/31/2020	5:00:00 a, m,	16.3	98	0
12/31/2020	6:00:00 a, m,	16.3	98	0
12/31/2020	7:00:00 a, m,	16.2	98	0
12/31/2020	8:00:00 a, m,	16.7	99	0
12/31/2020	9:00:00 a, m,	17	99	0
12/31/2020	10:00:00 a, m	17.2	99	0
12/31/2020	11:00:00 a, m	17.3	99	0
12/31/2020	12:00:00 p, m	17.6	99	0.2
12/31/2020	1:00:00 p, m,	18	99	0
12/31/2020	2:00:00 p, m,	18.6	99	0
12/31/2020	3:00:00 p, m,	19.2	98	0
12/31/2020	4:00:00 p, m,	19.4	97	0
12/31/2020	5:00:00 p, m,	19.3	98	0
12/31/2020	6:00:00 p, m,	19.1	97	0
12/31/2020	7:00:00 p, m,	18.6	98	0
12/31/2020	8:00:00 p, m,	18.5	99	0.2
12/31/2020	9:00:00 p, m,	18.2	99	0
12/31/2020	10:00:00 p, m	17.9	98	0
12/31/2020	11:00:00 p, m	17.7	98	0
Dec_20		17.740457	95.6223118	89.8
1/1/2021	12:00:00 a, m	17.3	98	0
1/1/2021	1:00:00 a, m,	17.1	98	0
1/1/2021	2:00:00 a, m,	16.8	98	0
1/1/2021	3:00:00 a, m,	16.4	98	0
1/1/2021	4:00:00 a, m,	16.4	98	0
1/1/2021	5:00:00 a, m,	16.4	98	0
1/1/2021	6:00:00 a, m,	16.5	98	0
1/1/2021	7:00:00 a, m,	16.5	98	0
1/1/2021	8:00:00 a, m,	17.1	99	0
1/1/2021	9:00:00 a, m,	17.3	99	0
1/1/2021	10:00:00 a, m	17.4	98	0
1/1/2021	11:00:00 a, m	17.4	99	2.4
1/1/2021	12:00:00 p, m	17.8	99	0.2
1/1/2021	1:00:00 p, m,	18.2	99	0
1/1/2021	2:00:00 p, m,	18.8	99	0
1/1/2021	3:00:00 p, m,	18.9	98	0
1/1/2021	4:00:00 p, m,	19.3	97	0

1/1/2021	5:00:00 p, m,	19.4	99	0
1/1/2021	6:00:00 p, m,	18.9	97	0
1/1/2021	7:00:00 p, m,	18	98	0
1/1/2021	8:00:00 p, m,	17.6	98	0
1/1/2021	9:00:00 p, m,	17.2	98	0
1/1/2021	10:00:00 p, m	16.9	98	0
1/1/2021	11:00:00 p, m	16.8	97	0
1/2/2021	12:00:00 a, m	16.7	97	0
1/2/2021	1:00:00 a, m,	16.5	97	0
1/2/2021	2:00:00 a, m,	16.5	97	0
1/2/2021	3:00:00 a, m,	16.3	96	0
1/2/2021	4:00:00 a, m,	16.5	98	0
1/2/2021	5:00:00 a, m,	16.4	98	0
1/2/2021	6:00:00 a, m,	16.3	97	0
1/2/2021	7:00:00 a, m,	16.5	97	0
1/2/2021	8:00:00 a, m,	17.1	96	0
1/2/2021	9:00:00 a, m,	17.5	98	0
1/2/2021	10:00:00 a, m	18.1	98	0
1/2/2021	11:00:00 a, m	18.6	98	0
1/2/2021	12:00:00 p, m	19	96	0
1/2/2021	1:00:00 p, m,	19.2	96	0
1/2/2021	2:00:00 p, m,	19.7	94	0
1/2/2021	3:00:00 p, m,	20.3	93	0
1/2/2021	4:00:00 p, m,	20.4	93	0
1/2/2021	5:00:00 p, m,	20.1	93	0
1/2/2021	6:00:00 p, m,	19	94	0
1/2/2021	7:00:00 p, m,	18.5	95	0
1/2/2021	8:00:00 p, m,	18.4	94	0
1/2/2021	9:00:00 p, m,	17.9	93	0
1/2/2021	10:00:00 p, m	18.1	95	0
1/2/2021	11:00:00 p, m	17.8	94	0
1/3/2021	12:00:00 a, m	17.9	95	0
1/3/2021	1:00:00 a, m,	17.7	94	0
1/3/2021	2:00:00 a, m,	17.1	94	0
1/3/2021	3:00:00 a, m,	17.2	95	0
1/3/2021	4:00:00 a, m,	17.3	95	0
1/3/2021	5:00:00 a, m,	17.2	95	0
1/3/2021	6:00:00 a, m,	16.8	95	0
1/3/2021	7:00:00 a, m,	17.1	97	0
1/3/2021	8:00:00 a, m,	17.3	97	0
1/3/2021	9:00:00 a, m,	17.7	97	0
1/3/2021	10:00:00 a, m	18.2	98	0
1/3/2021	11:00:00 a, m	18.4	98	0
1/3/2021	12:00:00 p, m	18	98	1.2
1/3/2021	1:00:00 p, m,	18.1	99	1.6
1/3/2021	2:00:00 p, m,	18.4	99	0.2
1/3/2021	3:00:00 p, m,	18.4	99	0.2

1/3/2021	4:00:00 p, m,	18.1	99	0
1/3/2021	5:00:00 p, m,	18.1	99	3.4
1/3/2021	6:00:00 p, m,	17.7	99	0.2
1/3/2021	7:00:00 p, m,	17.6	99	0
1/3/2021	8:00:00 p, m,	16.8	99	0
1/3/2021	9:00:00 p, m,	16.5	99	0
1/3/2021	10:00:00 p, m	16.3	99	0
1/3/2021	11:00:00 p, m	16.1	99	0
1/4/2021	12:00:00 a, m	15.9	98	0
1/4/2021	1:00:00 a, m,	15.8	99	0
1/4/2021	2:00:00 a, m,	15.9	99	0
1/4/2021	3:00:00 a, m,	15.9	99	0
1/4/2021	4:00:00 a, m,	15.8	99	0
1/4/2021	5:00:00 a, m,	15.9	99	0
1/4/2021	6:00:00 a, m,	16	99	0
1/4/2021	7:00:00 a, m,	16.1	99	0
1/4/2021	8:00:00 a, m,	16.7	99	0.2
1/4/2021	9:00:00 a, m,	17.1	99	0.8
1/4/2021	10:00:00 a, m	17.5	99	0
1/4/2021	11:00:00 a, m	17.4	99	0.2
1/4/2021	12:00:00 p, m	17.2	99	0.2
1/4/2021	1:00:00 p, m,	18.2	100	0
1/4/2021	2:00:00 p, m,	18.7	100	0
1/4/2021	3:00:00 p, m,	18.7	100	0
1/4/2021	4:00:00 p, m,	19.2	100	0
1/4/2021	5:00:00 p, m,	19.2	99	0
1/4/2021	6:00:00 p, m,	18.5	98	0
1/4/2021	7:00:00 p, m,	17.7	98	0
1/4/2021	8:00:00 p, m,	17.2	98	0
1/4/2021	9:00:00 p, m,	16.7	98	0
1/4/2021	10:00:00 p, m	16.5	98	0
1/4/2021	11:00:00 p, m	16.4	98	0.2
1/5/2021	12:00:00 a, m	16.3	98	0
1/5/2021	1:00:00 a, m,	16.8	99	0
1/5/2021	2:00:00 a, m,	16.4	98	3.4
1/5/2021	3:00:00 a, m,	15.8	99	9.6
1/5/2021	4:00:00 a, m,	15.6	99	15.2
1/5/2021	5:00:00 a, m,	14.2	99	17.8
1/5/2021	6:00:00 a, m,	14.2	99	3
1/5/2021	7:00:00 a, m,	14.6	99	0.4
1/5/2021	8:00:00 a, m,	14.8	99	2
1/5/2021	9:00:00 a, m,	14.8	99	5.4
1/5/2021	10:00:00 a, m	15.2	99	1
1/5/2021	11:00:00 a, m	16.2	100	0
1/5/2021	12:00:00 p, m	17.4	100	0
1/5/2021	1:00:00 p, m,	18.2	100	0
1/5/2021	2:00:00 p, m,	18.6	100	0

1/5/2021	3:00:00 p, m,	18.4	100	0
1/5/2021	4:00:00 p, m,	18.5	99	0
1/5/2021	5:00:00 p, m,	18.4	99	0
1/5/2021	6:00:00 p, m,	17.6	99	0
1/5/2021	7:00:00 p, m,	17.3	99	0
1/5/2021	8:00:00 p, m,	16.9	99	0
1/5/2021	9:00:00 p, m,	16.8	99	0
1/5/2021	10:00:00 p, m	16.8	99	0
1/5/2021	11:00:00 p, m	16.7	99	0
1/6/2021	12:00:00 a, m	16.5	99	0
1/6/2021	1:00:00 a, m,	16.2	98	0.4
1/6/2021	2:00:00 a, m,	16	99	2
1/6/2021	3:00:00 a, m,	15.8	99	1
1/6/2021	4:00:00 a, m,	15.7	99	0.8
1/6/2021	5:00:00 a, m,	15.4	99	0.4
1/6/2021	6:00:00 a, m,	15.4	99	0.2
1/6/2021	7:00:00 a, m,	15.8	99	0.6
1/6/2021	8:00:00 a, m,	15.9	100	0
1/6/2021	9:00:00 a, m,	16.3	100	0.8
1/6/2021	10:00:00 a, m	16.2	99	10.2
1/6/2021	11:00:00 a, m	16.5	100	1.4
1/6/2021	12:00:00 p, m	17	100	0
1/6/2021	1:00:00 p, m,	17.7	100	0
1/6/2021	2:00:00 p, m,	18.1	100	0
1/6/2021	3:00:00 p, m,	17.7	99	0
1/6/2021	4:00:00 p, m,	17.7	99	0
1/6/2021	5:00:00 p, m,	17.8	99	0
1/6/2021	6:00:00 p, m,	17.1	99	0
1/6/2021	7:00:00 p, m,	16.7	99	0
1/6/2021	8:00:00 p, m,	16.7	99	0
1/6/2021	9:00:00 p, m,	16.5	98	0
1/6/2021	10:00:00 p, m	16.4	98	0
1/6/2021	11:00:00 p, m	16.2	98	0
1/7/2021	12:00:00 a, m	16	98	0
1/7/2021	1:00:00 a, m,	15.8	97	0
1/7/2021	2:00:00 a, m,	15.4	97	0
1/7/2021	3:00:00 a, m,	15.8	98	0
1/7/2021	4:00:00 a, m,	15.6	97	0
1/7/2021	5:00:00 a, m,	15.6	97	0
1/7/2021	6:00:00 a, m,	15.4	98	0
1/7/2021	7:00:00 a, m,	15.4	98	0
1/7/2021	8:00:00 a, m,	16.1	99	0
1/7/2021	9:00:00 a, m,	16.8	99	0
1/7/2021	10:00:00 a, m	17.2	99	0
1/7/2021	11:00:00 a, m	17.7	99	0
1/7/2021	12:00:00 p, m	18.4	98	0
1/7/2021	1:00:00 p, m,	18.8	97	0

1/7/2021	2:00:00 p, m,	19.3	98	0
1/7/2021	3:00:00 p, m,	18.9	96	0
1/7/2021	4:00:00 p, m,	18.7	93	0
1/7/2021	5:00:00 p, m,	17.9	92	0
1/7/2021	6:00:00 p, m,	17.7	93	0
1/7/2021	7:00:00 p, m,	17.3	93	0
1/7/2021	8:00:00 p, m,	17.3	89	0
1/7/2021	9:00:00 p, m,	16.7	93	0
1/7/2021	10:00:00 p, m	16.7	93	0
1/7/2021	11:00:00 p, m	16.7	94	0
1/8/2021	12:00:00 a, m	16.6	94	0
1/8/2021	1:00:00 a, m,	16.3	93	0
1/8/2021	2:00:00 a, m,	16.3	94	0
1/8/2021	3:00:00 a, m,	16.3	92	0
1/8/2021	4:00:00 a, m,	16.4	93	0
1/8/2021	5:00:00 a, m,	16.3	94	0
1/8/2021	6:00:00 a, m,	16.2	94	0
1/8/2021	7:00:00 a, m,	16.1	95	0
1/8/2021	8:00:00 a, m,	16.5	96	0
1/8/2021	9:00:00 a, m,	17	95	0
1/8/2021	10:00:00 a, m	17.4	98	0
1/8/2021	11:00:00 a, m	18.1	97	0
1/8/2021	12:00:00 p, m	17.2	98	4.4
1/8/2021	1:00:00 p, m,	16.8	98	0
1/8/2021	2:00:00 p, m,	17.4	99	0
1/8/2021	3:00:00 p, m,	17.9	99	0.2
1/8/2021	4:00:00 p, m,	17.4	97	0.4
1/8/2021	5:00:00 p, m,	15.7	96	0.2
1/8/2021	6:00:00 p, m,	15.3	97	0
1/8/2021	7:00:00 p, m,	15.4	98	0
1/8/2021	8:00:00 p, m,	15.7	97	0
1/8/2021	9:00:00 p, m,	15.6	96	0
1/8/2021	10:00:00 p, m	15.6	96	0
1/8/2021	11:00:00 p, m	15.5	96	0
1/9/2021	12:00:00 a, m	15.6	95	0
1/9/2021	1:00:00 a, m,	15.6	95	0
1/9/2021	2:00:00 a, m,	15.6	95	0
1/9/2021	3:00:00 a, m,	15.6	95	0
1/9/2021	4:00:00 a, m,	15.4	96	0
1/9/2021	5:00:00 a, m,	15.3	95	0
1/9/2021	6:00:00 a, m,	15.2	96	0
1/9/2021	7:00:00 a, m,	15.1	98	0
1/9/2021	8:00:00 a, m,	15.8	98	0
1/9/2021	9:00:00 a, m,	16.4	97	0
1/9/2021	10:00:00 a, m	16.9	97	0
1/9/2021	11:00:00 a, m	17.7	95	0
1/9/2021	12:00:00 p, m	18.3	94	0

1/9/2021	1:00:00 p, m,	18.6	95	0
1/9/2021	2:00:00 p, m,	19.1	97	0
1/9/2021	3:00:00 p, m,	19.7	92	0
1/9/2021	4:00:00 p, m,	19.8	94	0
1/9/2021	5:00:00 p, m,	19.3	93	0
1/9/2021	6:00:00 p, m,	18.7	93	0
1/9/2021	7:00:00 p, m,	18	93	0
1/9/2021	8:00:00 p, m,	17.4	93	0
1/9/2021	9:00:00 p, m,	17.3	93	0
1/9/2021	10:00:00 p, m	17.1	95	0
1/9/2021	11:00:00 p, m	17.3	95	0
1/10/2021	12:00:00 a, m	17.3	94	0
1/10/2021	1:00:00 a, m,	17.3	95	0
1/10/2021	2:00:00 a, m,	17	95	0
1/10/2021	3:00:00 a, m,	16.6	95	0
1/10/2021	4:00:00 a, m,	16.4	95	0
1/10/2021	5:00:00 a, m,	16.5	96	0
1/10/2021	6:00:00 a, m,	16.6	96	0
1/10/2021	7:00:00 a, m,	16.5	95	0
1/10/2021	8:00:00 a, m,	16.9	97	0
1/10/2021	9:00:00 a, m,	17.3	97	0
1/10/2021	10:00:00 a, m	17.4	97	0
1/10/2021	11:00:00 a, m	17.9	97	0
1/10/2021	12:00:00 p, m	18.4	96	0
1/10/2021	1:00:00 p, m,	18.9	96	0
1/10/2021	2:00:00 p, m,	19.2	93	0
1/10/2021	3:00:00 p, m,	19.9	88	0
1/10/2021	4:00:00 p, m,	19.8	88	0
1/10/2021	5:00:00 p, m,	19	91	0
1/10/2021	6:00:00 p, m,	18.1	91	0
1/10/2021	7:00:00 p, m,	17.6	92	0
1/10/2021	8:00:00 p, m,	17.7	91	0
1/10/2021	9:00:00 p, m,	17.6	91	0
1/10/2021	10:00:00 p, m	16.9	94	0
1/10/2021	11:00:00 p, m	16.3	96	0.2
1/11/2021	12:00:00 a, m	16.4	96	0
1/11/2021	1:00:00 a, m,	16.6	96	0
1/11/2021	2:00:00 a, m,	16.5	95	0
1/11/2021	3:00:00 a, m,	16.2	97	0.4
1/11/2021	4:00:00 a, m,	15.7	98	1.4
1/11/2021	5:00:00 a, m,	15.6	98	1
1/11/2021	6:00:00 a, m,	15.5	99	0.2
1/11/2021	7:00:00 a, m,	15.7	99	0
1/11/2021	8:00:00 a, m,	16.3	99	0
1/11/2021	9:00:00 a, m,	16.9	99	0
1/11/2021	10:00:00 a, m	17.3	99	0
1/11/2021	11:00:00 a, m	17.7	99	0

1/11/2021	12:00:00 p, m	18.4	98	0
1/11/2021	1:00:00 p, m,	19.3	95	0
1/11/2021	2:00:00 p, m,	19.3	94	0
1/11/2021	3:00:00 p, m,	19.5	96	0
1/11/2021	4:00:00 p, m,	19.6	96	0
1/11/2021	5:00:00 p, m,	19.6	96	0
1/11/2021	6:00:00 p, m,	18.9	94	0
1/11/2021	7:00:00 p, m,	18.3	94	0
1/11/2021	8:00:00 p, m,	17.9	95	0
1/11/2021	9:00:00 p, m,	17.8	96	0
1/11/2021	10:00:00 p, m	17.6	95	0
1/11/2021	11:00:00 p, m	17.8	97	0
1/12/2021	12:00:00 a, m	17.6	97	0
1/12/2021	1:00:00 a, m,	17.3	97	0
1/12/2021	2:00:00 a, m,	17.1	95	0
1/12/2021	3:00:00 a, m,	16.9	96	0
1/12/2021	4:00:00 a, m,	16.8	96	0
1/12/2021	5:00:00 a, m,	16.7	96	0
1/12/2021	6:00:00 a, m,	16.6	96	0
1/12/2021	7:00:00 a, m,	16.6	97	0
1/12/2021	8:00:00 a, m,	16.9	96	0
1/12/2021	9:00:00 a, m,	17.2	97	0
1/12/2021	10:00:00 a, m	17.6	97	0
1/12/2021	11:00:00 a, m	18.5	97	0
1/12/2021	12:00:00 p, m	19.3	94	0
1/12/2021	1:00:00 p, m,	20.2	91	0
1/12/2021	2:00:00 p, m,	20.9	92	0
1/12/2021	3:00:00 p, m,	21	88	0
1/12/2021	4:00:00 p, m,	20.8	91	0
1/12/2021	5:00:00 p, m,	20.7	91	0
1/12/2021	6:00:00 p, m,	19.4	79	0
1/12/2021	7:00:00 p, m,	18	80	0
1/12/2021	8:00:00 p, m,	17.8	86	0
1/12/2021	9:00:00 p, m,	17.2	87	0
1/12/2021	10:00:00 p, m	17.1	88	0
1/12/2021	11:00:00 p, m	17	89	0
1/13/2021	12:00:00 a, m	16.9	91	0
1/13/2021	1:00:00 a, m,	17.1	91	0
1/13/2021	2:00:00 a, m,	17.2	93	0
1/13/2021	3:00:00 a, m,	17.1	92	0
1/13/2021	4:00:00 a, m,	16.9	92	0
1/13/2021	5:00:00 a, m,	16.8	93	0
1/13/2021	6:00:00 a, m,	16.8	93	0
1/13/2021	7:00:00 a, m,	16.8	93	0
1/13/2021	8:00:00 a, m,	17.3	95	0
1/13/2021	9:00:00 a, m,	17.8	94	0
1/13/2021	10:00:00 a, m	18.2	93	0

1/13/2021	11:00:00 a, m	18.8	92	0
1/13/2021	12:00:00 p, m	19.8	90	0
1/13/2021	1:00:00 p, m,	20.1	92	0
1/13/2021	2:00:00 p, m,	20.2	90	0
1/13/2021	3:00:00 p, m,	20.8	89	0
1/13/2021	4:00:00 p, m,	20.8	91	0
1/13/2021	5:00:00 p, m,	20.4	92	0
1/13/2021	6:00:00 p, m,	19.9	91	0
1/13/2021	7:00:00 p, m,	18.6	88	0
1/13/2021	8:00:00 p, m,	18.2	90	0
1/13/2021	9:00:00 p, m,	17.9	90	0
1/13/2021	10:00:00 p, m	17.7	89	0
1/13/2021	11:00:00 p, m	17.4	90	0
1/14/2021	12:00:00 a, m	16.9	89	0
1/14/2021	1:00:00 a, m,	16.8	90	0
1/14/2021	2:00:00 a, m,	16.6	92	0
1/14/2021	3:00:00 a, m,	16.3	90	0
1/14/2021	4:00:00 a, m,	16.3	90	0
1/14/2021	5:00:00 a, m,	16.2	91	0
1/14/2021	6:00:00 a, m,	15.9	92	0
1/14/2021	7:00:00 a, m,	16.2	93	0
1/14/2021	8:00:00 a, m,	16.6	94	0
1/14/2021	9:00:00 a, m,	16.9	95	0
1/14/2021	10:00:00 a, m	17.6	96	0
1/14/2021	11:00:00 a, m	18.5	95	0
1/14/2021	12:00:00 p, m	19.2	93	0
1/14/2021	1:00:00 p, m,	19.7	94	0
1/14/2021	2:00:00 p, m,	19.6	95	0
1/14/2021	3:00:00 p, m,	18.3	94	0
1/14/2021	4:00:00 p, m,	19.5	94	0
1/14/2021	5:00:00 p, m,	19.4	91	0.2
1/14/2021	6:00:00 p, m,	19.2	92	0
1/14/2021	7:00:00 p, m,	18.2	91	0
1/14/2021	8:00:00 p, m,	17.8	90	0
1/14/2021	9:00:00 p, m,	17.8	89	0
1/14/2021	10:00:00 p, m	17.9	89	0
1/14/2021	11:00:00 p, m	17.7	89	0
1/15/2021	12:00:00 a, m	17.4	91	0
1/15/2021	1:00:00 a, m,	17.3	92	0
1/15/2021	2:00:00 a, m,	17.4	91	0
1/15/2021	3:00:00 a, m,	17.4	94	0
1/15/2021	4:00:00 a, m,	16.8	94	0
1/15/2021	5:00:00 a, m,	16.4	94	0
1/15/2021	6:00:00 a, m,	16.2	94	0
1/15/2021	7:00:00 a, m,	16.6	95	0
1/15/2021	8:00:00 a, m,	17	94	0
1/15/2021	9:00:00 a, m,	17.4	96	0

1/15/2021	10:00:00 a, m	17.9	96	0
1/15/2021	11:00:00 a, m	18.1	97	0
1/15/2021	12:00:00 p, m	18.3	97	0
1/15/2021	1:00:00 p, m,	19.7	93	0
1/15/2021	2:00:00 p, m,	20.6	90	0
1/15/2021	3:00:00 p, m,	20.4	93	0
1/15/2021	4:00:00 p, m,	20.3	94	0
1/15/2021	5:00:00 p, m,	20	96	0
1/15/2021	6:00:00 p, m,	19.8	95	0
1/15/2021	7:00:00 p, m,	18.9	94	0
1/15/2021	8:00:00 p, m,	18.4	94	0
1/15/2021	9:00:00 p, m,	18.2	94	0
1/15/2021	10:00:00 p, m	18.1	94	0
1/15/2021	11:00:00 p, m	18.2	95	0
1/16/2021	12:00:00 a, m	18.1	95	0
1/16/2021	1:00:00 a, m,	17.9	96	0
1/16/2021	2:00:00 a, m,	17.7	95	0
1/16/2021	3:00:00 a, m,	17.6	96	0
1/16/2021	4:00:00 a, m,	17.6	97	0
1/16/2021	5:00:00 a, m,	17.6	98	0
1/16/2021	6:00:00 a, m,	17.6	98	0
1/16/2021	7:00:00 a, m,	17.3	98	0
1/16/2021	8:00:00 a, m,	17.6	99	0
1/16/2021	9:00:00 a, m,	17.9	99	0
1/16/2021	10:00:00 a, m	18.7	99	0
1/16/2021	11:00:00 a, m	18.6	98	0
1/16/2021	12:00:00 p, m	19.4	96	0
1/16/2021	1:00:00 p, m,	20.3	95	0
1/16/2021	2:00:00 p, m,	20.2	95	0
1/16/2021	3:00:00 p, m,	21.1	93	0
1/16/2021	4:00:00 p, m,	21.1	93	0
1/16/2021	5:00:00 p, m,	20.7	94	0
1/16/2021	6:00:00 p, m,	20.2	94	0
1/16/2021	7:00:00 p, m,	19.2	94	0
1/16/2021	8:00:00 p, m,	18.2	95	0
1/16/2021	9:00:00 p, m,	17.9	95	0
1/16/2021	10:00:00 p, m	17.9	94	0
1/16/2021	11:00:00 p, m	17.8	95	0
1/17/2021	12:00:00 a, m	17.5	95	0
1/17/2021	1:00:00 a, m,	17.7	96	0
1/17/2021	2:00:00 a, m,	17.7	96	0
1/17/2021	3:00:00 a, m,	17.3	98	0.4
1/17/2021	4:00:00 a, m,	16.8	98	0.8
1/17/2021	5:00:00 a, m,	16.8	99	4.6
1/17/2021	6:00:00 a, m,	16.9	99	0
1/17/2021	7:00:00 a, m,	16.9	99	0
1/17/2021	8:00:00 a, m,	17.1	99	0

1/17/2021	9:00:00 a, m,	17.2	99	0
1/17/2021	10:00:00 a, m	17.3	99	0
1/17/2021	11:00:00 a, m	18.1	100	0
1/17/2021	12:00:00 p, m	18.1	99	0
1/17/2021	1:00:00 p, m,	18.3	100	0
1/17/2021	2:00:00 p, m,	18.2	99	1.4
1/17/2021	3:00:00 p, m,	18.2	99	0
1/17/2021	4:00:00 p, m,	18.4	99	0
1/17/2021	5:00:00 p, m,	18.7	98	0
1/17/2021	6:00:00 p, m,	18.3	97	0
1/17/2021	7:00:00 p, m,	17.6	98	0
1/17/2021	8:00:00 p, m,	17.2	98	0
1/17/2021	9:00:00 p, m,	17	98	0
1/17/2021	10:00:00 p, m	16.8	98	0
1/17/2021	11:00:00 p, m	16.7	98	0
1/18/2021	12:00:00 a, m	16.9	98	0
1/18/2021	1:00:00 a, m,	17.1	98	0
1/18/2021	2:00:00 a, m,	16.7	97	0
1/18/2021	3:00:00 a, m,	16.7	97	0
1/18/2021	4:00:00 a, m,	16.4	98	0
1/18/2021	5:00:00 a, m,	16.6	98	0
1/18/2021	6:00:00 a, m,	16.4	98	0
1/18/2021	7:00:00 a, m,	16.3	98	0
1/18/2021	8:00:00 a, m,	16.7	98	0
1/18/2021	9:00:00 a, m,	17.2	99	0
1/18/2021	10:00:00 a, m	17.7	99	0
1/18/2021	11:00:00 a, m	17.3	99	0
1/18/2021	12:00:00 p, m	17.7	99	0
1/18/2021	1:00:00 p, m,	18.1	100	0
1/18/2021	2:00:00 p, m,	18.3	98	0.2
1/18/2021	3:00:00 p, m,	18.1	99	0
1/18/2021	4:00:00 p, m,	18.7	100	0
1/18/2021	5:00:00 p, m,	19.1	98	0
1/18/2021	6:00:00 p, m,	18.8	98	0
1/18/2021	7:00:00 p, m,	18.1	97	0
1/18/2021	8:00:00 p, m,	17.4	97	0
1/18/2021	9:00:00 p, m,	17	95	0
1/18/2021	10:00:00 p, m	16.7	95	0
1/18/2021	11:00:00 p, m	16.5	95	0
1/19/2021	12:00:00 a, m	16.9	96	0
1/19/2021	1:00:00 a, m,	17	95	0
1/19/2021	2:00:00 a, m,	16.7	95	0
1/19/2021	3:00:00 a, m,	16.3	96	0
1/19/2021	4:00:00 a, m,	16.3	97	0
1/19/2021	5:00:00 a, m,	16.2	96	0
1/19/2021	6:00:00 a, m,	16	96	0
1/19/2021	7:00:00 a, m,	16.1	96	0

1/19/2021	8:00:00 a, m,	16.8	97	0
1/19/2021	9:00:00 a, m,	17.3	98	0
1/19/2021	10:00:00 a, m	17.9	98	0
1/19/2021	11:00:00 a, m	18.2	97	0
1/19/2021	12:00:00 p, m	18.9	97	0
1/19/2021	1:00:00 p, m,	19.6	95	0
1/19/2021	2:00:00 p, m,	20.4	91	0
1/19/2021	3:00:00 p, m,	20.6	90	0
1/19/2021	4:00:00 p, m,	20.1	95	0
1/19/2021	5:00:00 p, m,	19.9	96	0
1/19/2021	6:00:00 p, m,	19.2	86	0
1/19/2021	7:00:00 p, m,	18.3	88	0
1/19/2021	8:00:00 p, m,	17.5	88	0
1/19/2021	9:00:00 p, m,	17.8	94	0
1/19/2021	10:00:00 p, m	17.6	93	0
1/19/2021	11:00:00 p, m	17.5	94	0
1/20/2021	12:00:00 a, m	17.3	93	0
1/20/2021	1:00:00 a, m,	17.1	94	0
1/20/2021	2:00:00 a, m,	16.9	94	0
1/20/2021	3:00:00 a, m,	17.1	94	0
1/20/2021	4:00:00 a, m,	17.2	95	0
1/20/2021	5:00:00 a, m,	17.1	95	0
1/20/2021	6:00:00 a, m,	17.1	95	0
1/20/2021	7:00:00 a, m,	17.1	95	0
1/20/2021	8:00:00 a, m,	17.5	95	0
1/20/2021	9:00:00 a, m,	17.6	96	0
1/20/2021	10:00:00 a, m	17.9	97	0
1/20/2021	11:00:00 a, m	18.7	96	0
1/20/2021	12:00:00 p, m	19.3	94	0
1/20/2021	1:00:00 p, m,	20.3	91	0
1/20/2021	2:00:00 p, m,	20.7	92	0
1/20/2021	3:00:00 p, m,	20.4	92	0
1/20/2021	4:00:00 p, m,	20.6	90	0
1/20/2021	5:00:00 p, m,	20.8	90	0.2
1/20/2021	6:00:00 p, m,	19.3	82	0
1/20/2021	7:00:00 p, m,	18.2	81	0
1/20/2021	8:00:00 p, m,	17.8	82	0
1/20/2021	9:00:00 p, m,	17.8	82	0
1/20/2021	10:00:00 p, m	17.6	84	0
1/20/2021	11:00:00 p, m	17.1	87	0
1/21/2021	12:00:00 a, m	16.9	86	0
1/21/2021	1:00:00 a, m,	16.9	88	0
1/21/2021	2:00:00 a, m,	16.7	88	0
1/21/2021	3:00:00 a, m,	16.4	91	0
1/21/2021	4:00:00 a, m,	16.3	89	0
1/21/2021	5:00:00 a, m,	16.3	89	0
1/21/2021	6:00:00 a, m,	15.9	88	0

1/21/2021	7:00:00 a, m,	16.2	91	0
1/21/2021	8:00:00 a, m,	16.4	92	0
1/21/2021	9:00:00 a, m,	16.7	92	0
1/21/2021	10:00:00 a, m	17.3	92	0
1/21/2021	11:00:00 a, m	18.4	93	0
1/21/2021	12:00:00 p, m	19.2	92	0
1/21/2021	1:00:00 p, m,	19.8	91	0
1/21/2021	2:00:00 p, m,	19.9	92	0
1/21/2021	3:00:00 p, m,	19.7	91	0
1/21/2021	4:00:00 p, m,	20.3	90	0
1/21/2021	5:00:00 p, m,	20.2	90	0
1/21/2021	6:00:00 p, m,	19.6	91	0
1/21/2021	7:00:00 p, m,	18.6	85	0
1/21/2021	8:00:00 p, m,	18	86	0
1/21/2021	9:00:00 p, m,	17.7	86	0
1/21/2021	10:00:00 p, m	17.8	84	0
1/21/2021	11:00:00 p, m	17.4	84	0
1/22/2021	12:00:00 a, m	17.2	86	0
1/22/2021	1:00:00 a, m,	16.8	87	0
1/22/2021	2:00:00 a, m,	16.6	88	0
1/22/2021	3:00:00 a, m,	16.4	89	0
1/22/2021	4:00:00 a, m,	16.5	91	0
1/22/2021	5:00:00 a, m,	16.9	91	0
1/22/2021	6:00:00 a, m,	16.5	91	0
1/22/2021	7:00:00 a, m,	16.4	93	0
1/22/2021	8:00:00 a, m,	17.1	94	0
1/22/2021	9:00:00 a, m,	17.6	94	0
1/22/2021	10:00:00 a, m	18.2	95	0
1/22/2021	11:00:00 a, m	18.6	95	0
1/22/2021	12:00:00 p, m	19.2	94	0
1/22/2021	1:00:00 p, m,	19.6	93	0
1/22/2021	2:00:00 p, m,	19.4	94	0
1/22/2021	3:00:00 p, m,	20	88	0
1/22/2021	4:00:00 p, m,	19.2	92	0
1/22/2021	5:00:00 p, m,	18.7	93	0
1/22/2021	6:00:00 p, m,	18	97	0.8
1/22/2021	7:00:00 p, m,	16.8	96	0.8
1/22/2021	8:00:00 p, m,	16.4	98	0
1/22/2021	9:00:00 p, m,	16.4	98	0
1/22/2021	10:00:00 p, m	16.3	97	0
1/22/2021	11:00:00 p, m	16.4	97	0
1/23/2021	12:00:00 a, m	16.6	96	0
1/23/2021	1:00:00 a, m,	16.7	94	0
1/23/2021	2:00:00 a, m,	16.7	95	0
1/23/2021	3:00:00 a, m,	16.5	95	0
1/23/2021	4:00:00 a, m,	16.2	97	0.2
1/23/2021	5:00:00 a, m,	15.9	97	0

1/23/2021	6:00:00 a, m,	16.1	97	0
1/23/2021	7:00:00 a, m,	16.3	97	0
1/23/2021	8:00:00 a, m,	16.8	97	0
1/23/2021	9:00:00 a, m,	17.1	97	0
1/23/2021	10:00:00 a, m	17.7	97	0
1/23/2021	11:00:00 a, m	17.9	97	0
1/23/2021	12:00:00 p, m	18.2	96	0
1/23/2021	1:00:00 p, m,	18.7	95	0
1/23/2021	2:00:00 p, m,	19.2	96	0
1/23/2021	3:00:00 p, m,	19.2	97	1
1/23/2021	4:00:00 p, m,	19.1	98	0
1/23/2021	5:00:00 p, m,	18.1	96	0
1/23/2021	6:00:00 p, m,	18.2	97	0
1/23/2021	7:00:00 p, m,	17.4	96	0
1/23/2021	8:00:00 p, m,	17	95	0
1/23/2021	9:00:00 p, m,	16.8	93	0
1/23/2021	10:00:00 p, m	16.8	93	0
1/23/2021	11:00:00 p, m	16.7	93	0
1/24/2021	12:00:00 a, m	16.6	92	0
1/24/2021	1:00:00 a, m,	16.6	93	0
1/24/2021	2:00:00 a, m,	16.3	93	0
1/24/2021	3:00:00 a, m,	16.4	93	0
1/24/2021	4:00:00 a, m,	16.5	95	0
1/24/2021	5:00:00 a, m,	16.9	94	0
1/24/2021	6:00:00 a, m,	16.4	96	0
1/24/2021	7:00:00 a, m,	16.5	95	0
1/24/2021	8:00:00 a, m,	17.1	96	0
1/24/2021	9:00:00 a, m,	17.7	95	0
1/24/2021	10:00:00 a, m	17.9	95	0
1/24/2021	11:00:00 a, m	18.4	95	0
1/24/2021	12:00:00 p, m	19.2	92	0
1/24/2021	1:00:00 p, m,	19.9	91	0
1/24/2021	2:00:00 p, m,	20.6	86	0
1/24/2021	3:00:00 p, m,	19.9	88	0
1/24/2021	4:00:00 p, m,	20.1	89	0
1/24/2021	5:00:00 p, m,	20.3	89	0
1/24/2021	6:00:00 p, m,	20	90	0
1/24/2021	7:00:00 p, m,	19	88	0
1/24/2021	8:00:00 p, m,	18.9	90	0
1/24/2021	9:00:00 p, m,	18.8	88	0
1/24/2021	10:00:00 p, m	18.7	88	0
1/24/2021	11:00:00 p, m	18.3	88	0
1/25/2021	12:00:00 a, m	18.1	88	0
1/25/2021	1:00:00 a, m,	17.8	91	0
1/25/2021	2:00:00 a, m,	17.6	93	0
1/25/2021	3:00:00 a, m,	17.2	94	0
1/25/2021	4:00:00 a, m,	17.1	95	0.2

1/25/2021	5:00:00 a, m,	16.9	96	0
1/25/2021	6:00:00 a, m,	17.1	96	0
1/25/2021	7:00:00 a, m,	17.2	96	0
1/25/2021	8:00:00 a, m,	17.4	97	0
1/25/2021	9:00:00 a, m,	17.9	96	0
1/25/2021	10:00:00 a, m	18.2	96	0
1/25/2021	11:00:00 a, m	18.1	97	0.6
1/25/2021	12:00:00 p, m	18.4	96	0
1/25/2021	1:00:00 p, m,	18.8	97	0.2
1/25/2021	2:00:00 p, m,	18.9	95	0
1/25/2021	3:00:00 p, m,	20.1	95	0
1/25/2021	4:00:00 p, m,	20.2	94	0
1/25/2021	5:00:00 p, m,	19.7	94	0
1/25/2021	6:00:00 p, m,	19.1	94	0
1/25/2021	7:00:00 p, m,	18.1	92	0
1/25/2021	8:00:00 p, m,	17.8	87	0
1/25/2021	9:00:00 p, m,	18.2	89	0
1/25/2021	10:00:00 p, m	18.1	88	0
1/25/2021	11:00:00 p, m	18.1	90	0
1/26/2021	12:00:00 a, m	17.4	89	0
1/26/2021	1:00:00 a, m,	17.3	90	0
1/26/2021	2:00:00 a, m,	17.3	90	0
1/26/2021	3:00:00 a, m,	17.4	91	0
1/26/2021	4:00:00 a, m,	17.5	91	0
1/26/2021	5:00:00 a, m,	17.5	92	0
1/26/2021	6:00:00 a, m,	17.2	92	0
1/26/2021	7:00:00 a, m,	17.2	93	0
1/26/2021	8:00:00 a, m,	17.3	94	0
1/26/2021	9:00:00 a, m,	17.6	96	0
1/26/2021	10:00:00 a, m	17.9	94	0
1/26/2021	11:00:00 a, m	18.4	94	0
1/26/2021	12:00:00 p, m	19.1	94	0
1/26/2021	1:00:00 p, m,	20.1	92	0
1/26/2021	2:00:00 p, m,	20.9	89	0
1/26/2021	3:00:00 p, m,	20.2	91	0
1/26/2021	4:00:00 p, m,	20.2	92	0
1/26/2021	5:00:00 p, m,	20.5	92	0.2
1/26/2021	6:00:00 p, m,	19.8	91	0
1/26/2021	7:00:00 p, m,	18.9	91	0
1/26/2021	8:00:00 p, m,	18.6	90	0
1/26/2021	9:00:00 p, m,	18.1	89	0
1/26/2021	10:00:00 p, m	17.9	90	0
1/26/2021	11:00:00 p, m	17.8	86	0
1/27/2021	12:00:00 a, m	17.6	85	0
1/27/2021	1:00:00 a, m,	17.5	89	0
1/27/2021	2:00:00 a, m,	17.6	90	0
1/27/2021	3:00:00 a, m,	17.3	89	0

1/27/2021	4:00:00 a, m,	17.2	89	0
1/27/2021	5:00:00 a, m,	16.9	89	0
1/27/2021	6:00:00 a, m,	16.8	89	0
1/27/2021	7:00:00 a, m,	16.7	92	0
1/27/2021	8:00:00 a, m,	17	91	0
1/27/2021	9:00:00 a, m,	17.2	93	0
1/27/2021	10:00:00 a, m	18.1	93	0
1/27/2021	11:00:00 a, m	18.9	93	0
1/27/2021	12:00:00 p, m	19.4	91	0
1/27/2021	1:00:00 p, m,	19.8	91	0
1/27/2021	2:00:00 p, m,	20.3	90	0
1/27/2021	3:00:00 p, m,	20.4	90	0
1/27/2021	4:00:00 p, m,	20.4	87	0
1/27/2021	5:00:00 p, m,	20.5	86	0
1/27/2021	6:00:00 p, m,	20	85	0
1/27/2021	7:00:00 p, m,	18.7	78	0
1/27/2021	8:00:00 p, m,	18.2	82	0
1/27/2021	9:00:00 p, m,	18.2	82	0
1/27/2021	10:00:00 p, m	17.9	83	0
1/27/2021	11:00:00 p, m	17.9	84	0
1/28/2021	12:00:00 a, m	17.6	86	0
1/28/2021	1:00:00 a, m,	17.4	86	0
1/28/2021	2:00:00 a, m,	17.3	87	0
1/28/2021	3:00:00 a, m,	17.3	89	0
1/28/2021	4:00:00 a, m,	17.3	88	0
1/28/2021	5:00:00 a, m,	16.8	88	0
1/28/2021	6:00:00 a, m,	16.7	89	0
1/28/2021	7:00:00 a, m,	17.1	91	0
1/28/2021	8:00:00 a, m,	17.2	92	0
1/28/2021	9:00:00 a, m,	17.1	92	0
1/28/2021	10:00:00 a, m	17.7	93	0
1/28/2021	11:00:00 a, m	18.7	92	0
1/28/2021	12:00:00 p, m	20.2	88	0
1/28/2021	1:00:00 p, m,	20.8	89	0
1/28/2021	2:00:00 p, m,	20.3	90	0
1/28/2021	3:00:00 p, m,	19.1	97	1.4
1/28/2021	4:00:00 p, m,	19.5	97	0
1/28/2021	5:00:00 p, m,	19.5	97	0
1/28/2021	6:00:00 p, m,	19.2	96	0
1/28/2021	7:00:00 p, m,	18.2	95	0
1/28/2021	8:00:00 p, m,	17.7	94	0
1/28/2021	9:00:00 p, m,	17.2	94	0
1/28/2021	10:00:00 p, m	17.2	95	0
1/28/2021	11:00:00 p, m	17.4	95	0
1/29/2021	12:00:00 a, m	17.1	95	0
1/29/2021	1:00:00 a, m,	16.9	94	0
1/29/2021	2:00:00 a, m,	16.9	95	0

1/29/2021	3:00:00 a, m,	17	95	0
1/29/2021	4:00:00 a, m,	16.6	94	0
1/29/2021	5:00:00 a, m,	16.7	95	0
1/29/2021	6:00:00 a, m,	16.6	95	0
1/29/2021	7:00:00 a, m,	16.5	96	0
1/29/2021	8:00:00 a, m,	16.8	96	0
1/29/2021	9:00:00 a, m,	17.2	97	0
1/29/2021	10:00:00 a, m	18	98	0
1/29/2021	11:00:00 a, m	18.5	97	0
1/29/2021	12:00:00 p, m	19.3	94	0
1/29/2021	1:00:00 p, m,	20.6	90	0
1/29/2021	2:00:00 p, m,	21.6	88	0
1/29/2021	3:00:00 p, m,	21.2	91	0
1/29/2021	4:00:00 p, m,	21.2	87	0
1/29/2021	5:00:00 p, m,	20.6	88	0
1/29/2021	6:00:00 p, m,	19.9	84	0
1/29/2021	7:00:00 p, m,	19.3	82	0
1/29/2021	8:00:00 p, m,	18.9	82	0
1/29/2021	9:00:00 p, m,	18.7	82	0
1/29/2021	10:00:00 p, m	18.8	85	0
1/29/2021	11:00:00 p, m	18.6	85	0
1/30/2021	12:00:00 a, m	18.1	87	0
1/30/2021	1:00:00 a, m,	17.8	90	0
1/30/2021	2:00:00 a, m,	17.3	90	0
1/30/2021	3:00:00 a, m,	17.2	90	0
1/30/2021	4:00:00 a, m,	16.8	91	0
1/30/2021	5:00:00 a, m,	16.7	90	0
1/30/2021	6:00:00 a, m,	16.2	93	0
1/30/2021	7:00:00 a, m,	16.5	93	0
1/30/2021	8:00:00 a, m,	16.8	93	0
1/30/2021	9:00:00 a, m,	17.2	94	0
1/30/2021	10:00:00 a, m	17.9	95	0
1/30/2021	11:00:00 a, m	18.8	94	0
1/30/2021	12:00:00 p, m	19.7	91	0
1/30/2021	1:00:00 p, m,	20	92	0
1/30/2021	2:00:00 p, m,	21.1	90	0
1/30/2021	3:00:00 p, m,	21.1	88	0
1/30/2021	4:00:00 p, m,	21.1	88	0
1/30/2021	5:00:00 p, m,	20.7	72	0
1/30/2021	6:00:00 p, m,	20.1	74	0
1/30/2021	7:00:00 p, m,	18.1	75	0
1/30/2021	8:00:00 p, m,	18.4	63	0
1/30/2021	9:00:00 p, m,	18.3	69	0
1/30/2021	10:00:00 p, m	17.5	74	0
1/30/2021	11:00:00 p, m	17.3	71	0
1/31/2021	12:00:00 a, m	17.2	72	0
1/31/2021	1:00:00 a, m,	16.9	74	0

1/31/2021	2:00:00 a, m,	16.4	77	0
1/31/2021	3:00:00 a, m,	16.7	69	0
1/31/2021	4:00:00 a, m,	16.4	68	0
1/31/2021	5:00:00 a, m,	16.1	72	0
1/31/2021	6:00:00 a, m,	16.4	71	0
1/31/2021	7:00:00 a, m,	16.5	72	0
1/31/2021	8:00:00 a, m,	16.4	77	0
1/31/2021	9:00:00 a, m,	16.5	79	0
1/31/2021	10:00:00 a, m	17.5	81	0
1/31/2021	11:00:00 a, m	18.7	78	0
1/31/2021	12:00:00 p, m	19.8	80	0
1/31/2021	1:00:00 p, m,	20	80	0
1/31/2021	2:00:00 p, m,	19.9	79	0
1/31/2021	3:00:00 p, m,	20.4	80	0
1/31/2021	4:00:00 p, m,	21.1	74	0
1/31/2021	5:00:00 p, m,	20.4	74	0
1/31/2021	6:00:00 p, m,	19.9	73	0
1/31/2021	7:00:00 p, m,	19.9	72	0
1/31/2021	8:00:00 p, m,	20.2	69	0
1/31/2021	9:00:00 p, m,	19.5	71	0
1/31/2021	10:00:00 p, m	19.8	70	0
1/31/2021	11:00:00 p, m	19.8	69	0
Jan_21		17.7479839	93.4758065	108.2
2/1/2021	12:00:00 a, m	19.7	69	0
2/1/2021	1:00:00 a, m,	19.6	69	0
2/1/2021	2:00:00 a, m,	19.6	72	0
2/1/2021	3:00:00 a, m,	18.7	76	0
2/1/2021	4:00:00 a, m,	18.6	76	0
2/1/2021	5:00:00 a, m,	18.4	79	0
2/1/2021	6:00:00 a, m,	18.1	80	0
2/1/2021	7:00:00 a, m,	18	81	0
2/1/2021	8:00:00 a, m,	18.5	81	0
2/1/2021	9:00:00 a, m,	19.2	84	0
2/1/2021	10:00:00 a, m	19.6	82	0
2/1/2021	11:00:00 a, m	19.8	84	0
2/1/2021	12:00:00 p, m	19.8	85	0
2/1/2021	1:00:00 p, m,	20	84	0
2/1/2021	2:00:00 p, m,	21.1	82	0
2/1/2021	3:00:00 p, m,	20.8	83	0
2/1/2021	4:00:00 p, m,	20.6	87	0
2/1/2021	5:00:00 p, m,	20.7	87	0
2/1/2021	6:00:00 p, m,	20.1	86	0
2/1/2021	7:00:00 p, m,	19.7	82	0
2/1/2021	8:00:00 p, m,	19.4	85	0
2/1/2021	9:00:00 p, m,	19.2	84	0
2/1/2021	10:00:00 p, m	18.9	84	0
2/1/2021	11:00:00 p, m	18.7	84	0

2/2/2021	12:00:00 a, m	18.6	85	0
2/2/2021	1:00:00 a, m,	18.3	83	0
2/2/2021	2:00:00 a, m,	17.9	85	0
2/2/2021	3:00:00 a, m,	17.8	87	0
2/2/2021	4:00:00 a, m,	17.9	86	0
2/2/2021	5:00:00 a, m,	17.8	86	0
2/2/2021	6:00:00 a, m,	17.9	86	0
2/2/2021	7:00:00 a, m,	18.1	86	0
2/2/2021	8:00:00 a, m,	18.4	88	0
2/2/2021	9:00:00 a, m,	18.7	88	0
2/2/2021	10:00:00 a, m	19.4	88	0
2/2/2021	11:00:00 a, m	19.7	87	0
2/2/2021	12:00:00 p, m	19.4	90	0
2/2/2021	1:00:00 p, m,	19.4	90	0.2
2/2/2021	2:00:00 p, m,	18.8	93	0
2/2/2021	3:00:00 p, m,	19.9	91	0
2/2/2021	4:00:00 p, m,	20.3	92	0
2/2/2021	5:00:00 p, m,	20.4	93	0
2/2/2021	6:00:00 p, m,	19.5	82	0
2/2/2021	7:00:00 p, m,	18.6	83	0
2/2/2021	8:00:00 p, m,	18.6	83	0
2/2/2021	9:00:00 p, m,	18.5	84	0
2/2/2021	10:00:00 p, m	18.6	84	0
2/2/2021	11:00:00 p, m	18.7	76	0
2/3/2021	12:00:00 a, m	18.7	81	0
2/3/2021	1:00:00 a, m,	18.3	77	0
2/3/2021	2:00:00 a, m,	18.1	77	0
2/3/2021	3:00:00 a, m,	17.5	79	0
2/3/2021	4:00:00 a, m,	17.4	80	0
2/3/2021	5:00:00 a, m,	17.1	81	0
2/3/2021	6:00:00 a, m,	16.8	82	0
2/3/2021	7:00:00 a, m,	16.9	83	0
2/3/2021	8:00:00 a, m,	17.2	85	0
2/3/2021	9:00:00 a, m,	18.2	85	0
2/3/2021	10:00:00 a, m	19	87	0
2/3/2021	11:00:00 a, m	19.4	87	0
2/3/2021	12:00:00 p, m	20.3	85	0
2/3/2021	1:00:00 p, m,	21.9	81	0
2/3/2021	2:00:00 p, m,	21.2	85	0
2/3/2021	3:00:00 p, m,	21.9	76	0
2/3/2021	4:00:00 p, m,	22.1	78	0
2/3/2021	5:00:00 p, m,	22.3	74	0
2/3/2021	6:00:00 p, m,	21.6	74	0
2/3/2021	7:00:00 p, m,	20.1	60	0
2/3/2021	8:00:00 p, m,	19.6	69	0
2/3/2021	9:00:00 p, m,	19.2	70	0
2/3/2021	10:00:00 p, m	19.4	73	0

2/3/2021	11:00:00 p, m	19.6	72	0
2/4/2021	12:00:00 a, m	19.7	77	0
2/4/2021	1:00:00 a, m,	18.9	80	0
2/4/2021	2:00:00 a, m,	18.4	79	0
2/4/2021	3:00:00 a, m,	18.4	80	0
2/4/2021	4:00:00 a, m,	18.4	80	0
2/4/2021	5:00:00 a, m,	17.9	81	0
2/4/2021	6:00:00 a, m,	17.3	84	0
2/4/2021	7:00:00 a, m,	17.1	84	0
2/4/2021	8:00:00 a, m,	17.3	87	0
2/4/2021	9:00:00 a, m,	18.6	84	0
2/4/2021	10:00:00 a, m	18.9	82	0
2/4/2021	11:00:00 a, m	19.7	83	0
2/4/2021	12:00:00 p, m	20.1	80	0
2/4/2021	1:00:00 p, m,	20.4	82	0
2/4/2021	2:00:00 p, m,	21	83	0
2/4/2021	3:00:00 p, m,	20.9	84	0
2/4/2021	4:00:00 p, m,	20.7	86	0
2/4/2021	5:00:00 p, m,	20.6	81	0
2/4/2021	6:00:00 p, m,	20.2	79	0
2/4/2021	7:00:00 p, m,	20.2	78	0
2/4/2021	8:00:00 p, m,	20.4	76	0
2/4/2021	9:00:00 p, m,	20.1	78	0
2/4/2021	10:00:00 p, m	19.8	79	0
2/4/2021	11:00:00 p, m	18.6	89	0
2/5/2021	12:00:00 a, m	17.7	92	0
2/5/2021	1:00:00 a, m,	17.6	92	0
2/5/2021	2:00:00 a, m,	17.4	91	0
2/5/2021	3:00:00 a, m,	17.7	89	0
2/5/2021	4:00:00 a, m,	17.4	91	0.2
2/5/2021	5:00:00 a, m,	17.3	90	0
2/5/2021	6:00:00 a, m,	17.6	88	0
2/5/2021	7:00:00 a, m,	17.9	88	0
2/5/2021	8:00:00 a, m,	18.6	88	0
2/5/2021	9:00:00 a, m,	18.9	88	0
2/5/2021	10:00:00 a, m	19.1	87	0
2/5/2021	11:00:00 a, m	19.3	87	0
2/5/2021	12:00:00 p, m	19.9	84	0
2/5/2021	1:00:00 p, m,	21.3	84	0
2/5/2021	2:00:00 p, m,	22.2	78	0
2/5/2021	3:00:00 p, m,	22.3	82	0
2/5/2021	4:00:00 p, m,	22.5	81	0
2/5/2021	5:00:00 p, m,	21.4	82	0
2/5/2021	6:00:00 p, m,	21.1	83	0
2/5/2021	7:00:00 p, m,	20.4	80	0
2/5/2021	8:00:00 p, m,	20.3	75	0
2/5/2021	9:00:00 p, m,	19.6	78	0

2/5/2021	10:00:00 p, m	19.2	77	0
2/5/2021	11:00:00 p, m	18.9	78	0
2/6/2021	12:00:00 a, m	19.1	79	0
2/6/2021	1:00:00 a, m,	18.7	80	0
2/6/2021	2:00:00 a, m,	18.6	82	0
2/6/2021	3:00:00 a, m,	18.4	83	0
2/6/2021	4:00:00 a, m,	18.2	84	0
2/6/2021	5:00:00 a, m,	18.3	85	0
2/6/2021	6:00:00 a, m,	18.3	86	0
2/6/2021	7:00:00 a, m,	18	89	0
2/6/2021	8:00:00 a, m,	17.9	90	0
2/6/2021	9:00:00 a, m,	18.6	93	0
2/6/2021	10:00:00 a, m	18.8	93	0
2/6/2021	11:00:00 a, m	18.7	96	0
2/6/2021	12:00:00 p, m	19.7	94	0
2/6/2021	1:00:00 p, m,	20.2	93	0
2/6/2021	2:00:00 p, m,	20.4	88	0
2/6/2021	3:00:00 p, m,	21.5	86	0
2/6/2021	4:00:00 p, m,	21.3	83	0
2/6/2021	5:00:00 p, m,	21.2	86	0
2/6/2021	6:00:00 p, m,	20.6	86	0
2/6/2021	7:00:00 p, m,	19.4	87	0
2/6/2021	8:00:00 p, m,	19.1	87	0
2/6/2021	9:00:00 p, m,	18.9	86	0
2/6/2021	10:00:00 p, m	18.6	86	0
2/6/2021	11:00:00 p, m	18.2	85	0
2/7/2021	12:00:00 a, m	18.2	83	0
2/7/2021	1:00:00 a, m,	18.1	86	0
2/7/2021	2:00:00 a, m,	17.9	88	0
2/7/2021	3:00:00 a, m,	17.8	87	0
2/7/2021	4:00:00 a, m,	17.5	87	0
2/7/2021	5:00:00 a, m,	17.4	87	0
2/7/2021	6:00:00 a, m,	17.7	87	0
2/7/2021	7:00:00 a, m,	18	86	0
2/7/2021	8:00:00 a, m,	18.2	87	0
2/7/2021	9:00:00 a, m,	19.2	87	0
2/7/2021	10:00:00 a, m	19.6	87	0
2/7/2021	11:00:00 a, m	20.1	85	0
2/7/2021	12:00:00 p, m	20.8	87	0
2/7/2021	1:00:00 p, m,	21.5	83	0
2/7/2021	2:00:00 p, m,	21.9	82	0
2/7/2021	3:00:00 p, m,	22.1	83	0
2/7/2021	4:00:00 p, m,	21.8	81	0
2/7/2021	5:00:00 p, m,	21.9	77	0
2/7/2021	6:00:00 p, m,	21.3	81	0
2/7/2021	7:00:00 p, m,	20	80	0
2/7/2021	8:00:00 p, m,	19.6	81	0

2/7/2021	9:00:00 p, m,	20.2	72	0
2/7/2021	10:00:00 p, m	19.6	79	0
2/7/2021	11:00:00 p, m	19.2	81	0
2/8/2021	12:00:00 a, m	18.9	81	0
2/8/2021	1:00:00 a, m,	18.8	79	0
2/8/2021	2:00:00 a, m,	18.4	81	0
2/8/2021	3:00:00 a, m,	18.3	81	0
2/8/2021	4:00:00 a, m,	18.1	81	0
2/8/2021	5:00:00 a, m,	18.1	82	0
2/8/2021	6:00:00 a, m,	18.2	84	0
2/8/2021	7:00:00 a, m,	18.4	85	0
2/8/2021	8:00:00 a, m,	18.7	85	0
2/8/2021	9:00:00 a, m,	19.3	84	0
2/8/2021	10:00:00 a, m	19.7	87	0
2/8/2021	11:00:00 a, m	20.2	88	0
2/8/2021	12:00:00 p, m	20.6	86	0
2/8/2021	1:00:00 p, m,	21.6	82	0
2/8/2021	2:00:00 p, m,	22.1	82	0
2/8/2021	3:00:00 p, m,	21.7	86	0
2/8/2021	4:00:00 p, m,	21.7	86	0
2/8/2021	5:00:00 p, m,	21.2	86	0
2/8/2021	6:00:00 p, m,	20.4	86	0.2
2/8/2021	7:00:00 p, m,	19.7	84	0
2/8/2021	8:00:00 p, m,	19.7	85	0
2/8/2021	9:00:00 p, m,	19.6	86	0
2/8/2021	10:00:00 p, m	19.1	86	0
2/8/2021	11:00:00 p, m	19	84	0
2/9/2021	12:00:00 a, m	18.8	87	0
2/9/2021	1:00:00 a, m,	18.3	87	0
2/9/2021	2:00:00 a, m,	18.6	88	0
2/9/2021	3:00:00 a, m,	18.2	88	0
2/9/2021	4:00:00 a, m,	18	92	0
2/9/2021	5:00:00 a, m,	17.8	91	0
2/9/2021	6:00:00 a, m,	17.6	91	0
2/9/2021	7:00:00 a, m,	17.5	91	0
2/9/2021	8:00:00 a, m,	17.6	94	0
2/9/2021	9:00:00 a, m,	18.1	96	0
2/9/2021	10:00:00 a, m	18.1	97	0
2/9/2021	11:00:00 a, m	19.2	94	0
2/9/2021	12:00:00 p, m	19.8	93	0
2/9/2021	1:00:00 p, m,	20.5	94	0
2/9/2021	2:00:00 p, m,	20.2	94	0
2/9/2021	3:00:00 p, m,	19.6	94	0
2/9/2021	4:00:00 p, m,	19.7	93	0
2/9/2021	5:00:00 p, m,	19.7	95	0
2/9/2021	6:00:00 p, m,	19.7	92	0
2/9/2021	7:00:00 p, m,	18.8	93	0

2/9/2021	8:00:00 p, m,	18.6	92	0
2/9/2021	9:00:00 p, m,	18.8	92	0
2/9/2021	10:00:00 p, m	18.8	92	0
2/9/2021	11:00:00 p, m	17.9	93	20.2
2/10/2021	12:00:00 a, m	16.3	97	7.6
2/10/2021	1:00:00 a, m,	16.6	98	0.2
2/10/2021	2:00:00 a, m,	16.7	98	0
2/10/2021	3:00:00 a, m,	16.8	98	0
2/10/2021	4:00:00 a, m,	16.7	98	0.2
2/10/2021	5:00:00 a, m,	16.4	98	0
2/10/2021	6:00:00 a, m,	16.7	98	0
2/10/2021	7:00:00 a, m,	16.8	98	0
2/10/2021	8:00:00 a, m,	16.8	99	0
2/10/2021	9:00:00 a, m,	17.1	99	0
2/10/2021	10:00:00 a, m	17.4	99	0
2/10/2021	11:00:00 a, m	17.7	99	0
2/10/2021	12:00:00 p, m	18.4	99	0
2/10/2021	1:00:00 p, m,	18.9	99	0
2/10/2021	2:00:00 p, m,	19.2	99	0
2/10/2021	3:00:00 p, m,	18.9	99	0
2/10/2021	4:00:00 p, m,	19.4	99	0
2/10/2021	5:00:00 p, m,	18.9	98	0
2/10/2021	6:00:00 p, m,	18.6	98	0.2
2/10/2021	7:00:00 p, m,	18.3	98	0
2/10/2021	8:00:00 p, m,	18.1	98	0
2/10/2021	9:00:00 p, m,	18.1	98	0
2/10/2021	10:00:00 p, m	17.9	98	0
2/10/2021	11:00:00 p, m	17.2	98	2.4
2/11/2021	12:00:00 a, m	17.2	98	1
2/11/2021	1:00:00 a, m,	16.8	98	0
2/11/2021	2:00:00 a, m,	16.8	98	1.8
2/11/2021	3:00:00 a, m,	16.8	99	0.8
2/11/2021	4:00:00 a, m,	16.4	98	0
2/11/2021	5:00:00 a, m,	16.1	98	0
2/11/2021	6:00:00 a, m,	16.1	98	0
2/11/2021	7:00:00 a, m,	16.1	99	0
2/11/2021	8:00:00 a, m,	16.4	99	0
2/11/2021	9:00:00 a, m,	16.9	99	0
2/11/2021	10:00:00 a, m	17.2	99	0
2/11/2021	11:00:00 a, m	17.7	99	0
2/11/2021	12:00:00 p, m	18.3	99	0
2/11/2021	1:00:00 p, m,	18.9	99	0
2/11/2021	2:00:00 p, m,	19.5	98	0
2/11/2021	3:00:00 p, m,	19.8	97	0
2/11/2021	4:00:00 p, m,	19.9	97	0
2/11/2021	5:00:00 p, m,	19.8	97	0
2/11/2021	6:00:00 p, m,	19.2	97	0

2/11/2021	7:00:00 p, m,	18.6	97	0
2/11/2021	8:00:00 p, m,	18.6	97	0
2/11/2021	9:00:00 p, m,	18.4	97	0
2/11/2021	10:00:00 p, m	18.2	97	0
2/11/2021	11:00:00 p, m	18.2	98	0
2/12/2021	12:00:00 a, m	18.2	96	0
2/12/2021	1:00:00 a, m,	17.9	96	0
2/12/2021	2:00:00 a, m,	17.8	97	0
2/12/2021	3:00:00 a, m,	17.7	98	0.4
2/12/2021	4:00:00 a, m,	17.4	98	0
2/12/2021	5:00:00 a, m,	17.2	98	0
2/12/2021	6:00:00 a, m,	16.8	98	0
2/12/2021	7:00:00 a, m,	16.9	99	0
2/12/2021	8:00:00 a, m,	16.9	99	0.4
2/12/2021	9:00:00 a, m,	17.4	99	0
2/12/2021	10:00:00 a, m	17.6	98	0
2/12/2021	11:00:00 a, m	18.4	99	0
2/12/2021	12:00:00 p, m	19	98	0
2/12/2021	1:00:00 p, m,	19.4	97	0
2/12/2021	2:00:00 p, m,	19.9	95	0
2/12/2021	3:00:00 p, m,	20.2	94	0
2/12/2021	4:00:00 p, m,	20	96	0
2/12/2021	5:00:00 p, m,	19.8	96	0
2/12/2021	6:00:00 p, m,	19.1	96	0.2
2/12/2021	7:00:00 p, m,	18.1	95	0.2
2/12/2021	8:00:00 p, m,	17.4	98	0
2/12/2021	9:00:00 p, m,	17.4	98	0
2/12/2021	10:00:00 p, m	17.6	97	0
2/12/2021	11:00:00 p, m	17.4	97	0
2/13/2021	12:00:00 a, m	17.4	97	0
2/13/2021	1:00:00 a, m,	17.2	98	0
2/13/2021	2:00:00 a, m,	16.8	97	0
2/13/2021	3:00:00 a, m,	16.4	96	0.2
2/13/2021	4:00:00 a, m,	16.1	97	0
2/13/2021	5:00:00 a, m,	15.5	95	0
2/13/2021	6:00:00 a, m,	15.4	95	0
2/13/2021	7:00:00 a, m,	15.5	96	0
2/13/2021	8:00:00 a, m,	15.8	97	0
2/13/2021	9:00:00 a, m,	16.6	95	0
2/13/2021	10:00:00 a, m	17.4	96	0
2/13/2021	11:00:00 a, m	18.4	97	0
2/13/2021	12:00:00 p, m	19.6	92	0
2/13/2021	1:00:00 p, m,	20.4	93	0
2/13/2021	2:00:00 p, m,	20.6	93	0
2/13/2021	3:00:00 p, m,	20.4	93	0
2/13/2021	4:00:00 p, m,	20.3	91	0
2/13/2021	5:00:00 p, m,	19.8	94	0

2/13/2021	6:00:00 p, m,	19.5	93	0
2/13/2021	7:00:00 p, m,	18.6	92	0
2/13/2021	8:00:00 p, m,	18.1	89	0
2/13/2021	9:00:00 p, m,	17.7	90	0
2/13/2021	10:00:00 p, m	17.3	90	0
2/13/2021	11:00:00 p, m	17.1	91	0
2/14/2021	12:00:00 a, m	16.9	91	0
2/14/2021	1:00:00 a, m,	16.8	91	0
2/14/2021	2:00:00 a, m,	16.5	92	0
2/14/2021	3:00:00 a, m,	16.6	93	0
2/14/2021	4:00:00 a, m,	16.6	94	0
2/14/2021	5:00:00 a, m,	16.7	96	0
2/14/2021	6:00:00 a, m,	16.9	95	0
2/14/2021	7:00:00 a, m,	16.8	96	0
2/14/2021	8:00:00 a, m,	17.3	97	0
2/14/2021	9:00:00 a, m,	17.8	97	0
2/14/2021	10:00:00 a, m	18.3	98	0
2/14/2021	11:00:00 a, m	18.4	97	0
2/14/2021	12:00:00 p, m	19.1	95	0
2/14/2021	1:00:00 p, m,	19.4	95	0
2/14/2021	2:00:00 p, m,	19.7	92	0
2/14/2021	3:00:00 p, m,	20.8	93	0
2/14/2021	4:00:00 p, m,	20	93	5
2/14/2021	5:00:00 p, m,	18.8	98	1.4
2/14/2021	6:00:00 p, m,	18.7	99	0
2/14/2021	7:00:00 p, m,	18.2	98	0
2/14/2021	8:00:00 p, m,	18	98	0
2/14/2021	9:00:00 p, m,	17.8	99	0
2/14/2021	10:00:00 p, m	17.5	98	0
2/14/2021	11:00:00 p, m	17.4	98	0
2/15/2021	12:00:00 a, m	17.3	98	0
2/15/2021	1:00:00 a, m,	17.3	98	0.2
2/15/2021	2:00:00 a, m,	17	98	0
2/15/2021	3:00:00 a, m,	16.9	98	0
2/15/2021	4:00:00 a, m,	17	99	0
2/15/2021	5:00:00 a, m,	17	98	0.2
2/15/2021	6:00:00 a, m,	16.7	98	0.8
2/15/2021	7:00:00 a, m,	16.7	99	1
2/15/2021	8:00:00 a, m,	16.4	99	0.4
2/15/2021	9:00:00 a, m,	16.9	99	0.4
2/15/2021	10:00:00 a, m	17.1	99	0.2
2/15/2021	11:00:00 a, m	17.5	99	0.2
2/15/2021	12:00:00 p, m	17.9	99	0.2
2/15/2021	1:00:00 p, m,	17.9	99	0
2/15/2021	2:00:00 p, m,	18.8	99	0
2/15/2021	3:00:00 p, m,	19.1	100	0.2
2/15/2021	4:00:00 p, m,	19.5	99	0

2/15/2021	5:00:00 p, m,	19.9	99	0.2
2/15/2021	6:00:00 p, m,	19.4	98	0
2/15/2021	7:00:00 p, m,	18.4	98	0
2/15/2021	8:00:00 p, m,	17.1	97	0
2/15/2021	9:00:00 p, m,	16.6	96	0.2
2/15/2021	10:00:00 p, m	16.9	99	0
2/15/2021	11:00:00 p, m	17.6	98	0
2/16/2021	12:00:00 a, m	17.3	97	0
2/16/2021	1:00:00 a, m,	17.3	98	0
2/16/2021	2:00:00 a, m,	17.3	97	0
2/16/2021	3:00:00 a, m,	17.1	98	0
2/16/2021	4:00:00 a, m,	17.1	97	0.2
2/16/2021	5:00:00 a, m,	17.1	98	0
2/16/2021	6:00:00 a, m,	16.3	98	0
2/16/2021	7:00:00 a, m,	16.1	98	0
2/16/2021	8:00:00 a, m,	16.1	99	0
2/16/2021	9:00:00 a, m,	16.6	99	0
2/16/2021	10:00:00 a, m	17.2	99	0
2/16/2021	11:00:00 a, m	17.7	99	0
2/16/2021	12:00:00 p, m	18.8	99	0
2/16/2021	1:00:00 p, m,	19.2	98	0.2
2/16/2021	2:00:00 p, m,	19.5	98	0
2/16/2021	3:00:00 p, m,	19.2	98	0
2/16/2021	4:00:00 p, m,	19.3	98	0
2/16/2021	5:00:00 p, m,	18.9	98	0
2/16/2021	6:00:00 p, m,	18.8	98	0
2/16/2021	7:00:00 p, m,	18.1	98	0
2/16/2021	8:00:00 p, m,	17.9	98	0
2/16/2021	9:00:00 p, m,	17.7	98	0
2/16/2021	10:00:00 p, m	17.8	98	0.2
2/16/2021	11:00:00 p, m	17.7	98	0
2/17/2021	12:00:00 a, m	17.4	97	0
2/17/2021	1:00:00 a, m,	17.6	98	0
2/17/2021	2:00:00 a, m,	16.7	95	0
2/17/2021	3:00:00 a, m,	16.3	99	0
2/17/2021	4:00:00 a, m,	16.6	99	0
2/17/2021	5:00:00 a, m,	16.7	99	0
2/17/2021	6:00:00 a, m,	16.8	99	0
2/17/2021	7:00:00 a, m,	16.8	99	0
2/17/2021	8:00:00 a, m,	17.1	99	0
2/17/2021	9:00:00 a, m,	17.3	100	0
2/17/2021	10:00:00 a, m	17.3	100	0.2
2/17/2021	11:00:00 a, m	17.5	99	0
2/17/2021	12:00:00 p, m	17.7	100	0
2/17/2021	1:00:00 p, m,	18.1	100	0
2/17/2021	2:00:00 p, m,	18.6	99	0
2/17/2021	3:00:00 p, m,	18.9	99	0

2/17/2021	4:00:00 p, m,	18.9	99	0
2/17/2021	5:00:00 p, m,	19.1	99	0
2/17/2021	6:00:00 p, m,	18.5	99	0
2/17/2021	7:00:00 p, m,	17.9	99	0.2
2/17/2021	8:00:00 p, m,	17.8	99	0
2/17/2021	9:00:00 p, m,	17.6	99	0
2/17/2021	10:00:00 p, m	17.3	98	0
2/17/2021	11:00:00 p, m	17.3	98	0
2/18/2021	12:00:00 a, m	17.7	98	0
2/18/2021	1:00:00 a, m,	17.3	98	0
2/18/2021	2:00:00 a, m,	16.8	99	0
2/18/2021	3:00:00 a, m,	16.7	99	0
2/18/2021	4:00:00 a, m,	16.7	99	0
2/18/2021	5:00:00 a, m,	16.4	99	0
2/18/2021	6:00:00 a, m,	16.3	99	0.2
2/18/2021	7:00:00 a, m,	16.2	98	0
2/18/2021	8:00:00 a, m,	16.4	99	0
2/18/2021	9:00:00 a, m,	17.1	99	0
2/18/2021	10:00:00 a, m	17.3	99	0
2/18/2021	11:00:00 a, m	17.7	100	0
2/18/2021	12:00:00 p, m	17.9	99	0
2/18/2021	1:00:00 p, m,	17.9	100	0
2/18/2021	2:00:00 p, m,	18.2	98	0
2/18/2021	3:00:00 p, m,	19.1	97	0.2
2/18/2021	4:00:00 p, m,	18.9	97	0
2/18/2021	5:00:00 p, m,	19.1	97	0
2/18/2021	6:00:00 p, m,	18.7	98	0
2/18/2021	7:00:00 p, m,	18.1	98	0
2/18/2021	8:00:00 p, m,	17.7	98	0
2/18/2021	9:00:00 p, m,	17.7	98	0
2/18/2021	10:00:00 p, m	17.6	98	0
2/18/2021	11:00:00 p, m	17.5	98	0
2/19/2021	12:00:00 a, m	17.4	98	0
2/19/2021	1:00:00 a, m,	17	98	0
2/19/2021	2:00:00 a, m,	16.5	99	0.2
2/19/2021	3:00:00 a, m,	16.3	99	0
2/19/2021	4:00:00 a, m,	16.3	99	0
2/19/2021	5:00:00 a, m,	16.2	99	0
2/19/2021	6:00:00 a, m,	16.2	99	0
2/19/2021	7:00:00 a, m,	16.2	99	0
2/19/2021	8:00:00 a, m,	16.2	99	0.2
2/19/2021	9:00:00 a, m,	16.5	99	0
2/19/2021	10:00:00 a, m	16.7	99	6.8
2/19/2021	11:00:00 a, m	16.7	100	1.8
2/19/2021	12:00:00 p, m	16.9	100	0
2/19/2021	1:00:00 p, m,	17.7	100	0.2
2/19/2021	2:00:00 p, m,	17.9	100	0

2/19/2021	3:00:00 p, m,	17.9	100	0
2/19/2021	4:00:00 p, m,	17.9	100	0
2/19/2021	5:00:00 p, m,	17.8	100	0
2/19/2021	6:00:00 p, m,	17.7	100	0
2/19/2021	7:00:00 p, m,	17.2	99	0
2/19/2021	8:00:00 p, m,	17.1	99	0
2/19/2021	9:00:00 p, m,	17	100	0.6
2/19/2021	10:00:00 p, m	16.8	99	1.4
2/19/2021	11:00:00 p, m	16.3	99	10.4
2/20/2021	12:00:00 a, m	16.1	99	0.8
2/20/2021	1:00:00 a, m,	15.8	99	0.2
2/20/2021	2:00:00 a, m,	15.9	99	0
2/20/2021	3:00:00 a, m,	16	100	0
2/20/2021	4:00:00 a, m,	15.9	99	0
2/20/2021	5:00:00 a, m,	15.8	99	0
2/20/2021	6:00:00 a, m,	15.8	99	0
2/20/2021	7:00:00 a, m,	15.8	99	0
2/20/2021	8:00:00 a, m,	16.2	100	0
2/20/2021	9:00:00 a, m,	16.7	100	0
2/20/2021	10:00:00 a, m	17.2	100	0.2
2/20/2021	11:00:00 a, m	17.2	100	0
2/20/2021	12:00:00 p, m	17.7	100	0
2/20/2021	1:00:00 p, m,	17.5	100	1.2
2/20/2021	2:00:00 p, m,	18.3	100	0
2/20/2021	3:00:00 p, m,	18.5	100	0
2/20/2021	4:00:00 p, m,	18.5	100	0
2/20/2021	5:00:00 p, m,	18.6	100	0
2/20/2021	6:00:00 p, m,	18.2	99	0
2/20/2021	7:00:00 p, m,	17.6	99	0
2/20/2021	8:00:00 p, m,	17.5	99	0
2/20/2021	9:00:00 p, m,	17.4	99	0
2/20/2021	10:00:00 p, m	17.4	99	0
2/20/2021	11:00:00 p, m	17	99	0
2/21/2021	12:00:00 a, m	16.6	99	0
2/21/2021	1:00:00 a, m,	16.4	98	0
2/21/2021	2:00:00 a, m,	16.1	98	0
2/21/2021	3:00:00 a, m,	16.2	99	0
2/21/2021	4:00:00 a, m,	16.3	99	1
2/21/2021	5:00:00 a, m,	15.9	99	2
2/21/2021	6:00:00 a, m,	15.8	99	0.4
2/21/2021	7:00:00 a, m,	15.8	99	0
2/21/2021	8:00:00 a, m,	16.3	100	0
2/21/2021	9:00:00 a, m,	17.1	100	0
2/21/2021	10:00:00 a, m	17.1	100	2.4
2/21/2021	11:00:00 a, m	16.7	100	5
2/21/2021	12:00:00 p, m	17.1	100	0.8
2/21/2021	1:00:00 p, m,	18.2	100	0

2/21/2021	2:00:00 p, m,	18.6	100	0
2/21/2021	3:00:00 p, m,	18.7	99	0
2/21/2021	4:00:00 p, m,	18.7	99	0
2/21/2021	5:00:00 p, m,	18.7	99	0
2/21/2021	6:00:00 p, m,	18.4	99	0
2/21/2021	7:00:00 p, m,	18	99	0
2/21/2021	8:00:00 p, m,	17.8	99	0
2/21/2021	9:00:00 p, m,	17.4	99	0
2/21/2021	10:00:00 p, m	17.3	99	0
2/21/2021	11:00:00 p, m	17.2	99	0
2/22/2021	12:00:00 a, m	17.1	99	0.4
2/22/2021	1:00:00 a, m,	16.9	99	1
2/22/2021	2:00:00 a, m,	16.8	99	0
2/22/2021	3:00:00 a, m,	16.8	99	0
2/22/2021	4:00:00 a, m,	16.7	100	0.4
2/22/2021	5:00:00 a, m,	16.3	100	2.8
2/22/2021	6:00:00 a, m,	16.2	100	0.2
2/22/2021	7:00:00 a, m,	16.2	100	0.4
2/22/2021	8:00:00 a, m,	16.3	100	0.4
2/22/2021	9:00:00 a, m,	16.2	100	0.2
2/22/2021	10:00:00 a, m	16.7	100	0.2
2/22/2021	11:00:00 a, m	17	100	0
2/22/2021	12:00:00 p, m	17.2	100	0
2/22/2021	1:00:00 p, m,	17.1	100	0
2/22/2021	2:00:00 p, m,	17.3	100	0
2/22/2021	3:00:00 p, m,	17.4	100	0
2/22/2021	4:00:00 p, m,	17.4	100	0
2/22/2021	5:00:00 p, m,	17.3	99	0
2/22/2021	6:00:00 p, m,	16.9	99	0
2/22/2021	7:00:00 p, m,	16.4	99	0
2/22/2021	8:00:00 p, m,	16.1	99	0
2/22/2021	9:00:00 p, m,	16.2	99	0
2/22/2021	10:00:00 p, m	16.2	98	0
2/22/2021	11:00:00 p, m	16.2	98	0
2/23/2021	12:00:00 a, m	15.9	97	0
2/23/2021	1:00:00 a, m,	15.7	97	0
2/23/2021	2:00:00 a, m,	15.4	96	0
2/23/2021	3:00:00 a, m,	15.3	97	0
2/23/2021	4:00:00 a, m,	15.2	96	0
2/23/2021	5:00:00 a, m,	15.4	96	0
2/23/2021	6:00:00 a, m,	15.6	96	0
2/23/2021	7:00:00 a, m,	15.7	98	0
2/23/2021	8:00:00 a, m,	16.1	96	0
2/23/2021	9:00:00 a, m,	16.4	97	0
2/23/2021	10:00:00 a, m	16.7	97	0
2/23/2021	11:00:00 a, m	17.4	97	0
2/23/2021	12:00:00 p, m	18.2	97	0

2/23/2021	1:00:00 p, m,	18.6	94	0
2/23/2021	2:00:00 p, m,	18.6	95	0
2/23/2021	3:00:00 p, m,	19.1	96	0
2/23/2021	4:00:00 p, m,	19.4	96	0
2/23/2021	5:00:00 p, m,	18.8	93	0
2/23/2021	6:00:00 p, m,	18.2	93	0
2/23/2021	7:00:00 p, m,	17.7	93	0
2/23/2021	8:00:00 p, m,	17.4	93	0
2/23/2021	9:00:00 p, m,	17.3	93	0
2/23/2021	10:00:00 p, m	17.4	92	0
2/23/2021	11:00:00 p, m	17.5	93	0
2/24/2021	12:00:00 a, m	17.3	93	0
2/24/2021	1:00:00 a, m,	17.1	94	0
2/24/2021	2:00:00 a, m,	16.9	93	0
2/24/2021	3:00:00 a, m,	17.1	93	0
2/24/2021	4:00:00 a, m,	17.1	95	0
2/24/2021	5:00:00 a, m,	17.1	94	0
2/24/2021	6:00:00 a, m,	17.2	95	0
2/24/2021	7:00:00 a, m,	17.3	94	0
2/24/2021	8:00:00 a, m,	17.4	95	0
2/24/2021	9:00:00 a, m,	17.7	96	0
2/24/2021	10:00:00 a, m	17.3	98	0.6
2/24/2021	11:00:00 a, m	17.5	99	0.2
2/24/2021	12:00:00 p, m	17.8	99	0
2/24/2021	1:00:00 p, m,	19.1	98	0
2/24/2021	2:00:00 p, m,	18.7	98	0.2
2/24/2021	3:00:00 p, m,	18.9	97	0
2/24/2021	4:00:00 p, m,	19.3	93	0
2/24/2021	5:00:00 p, m,	18.9	96	0
2/24/2021	6:00:00 p, m,	17.7	98	0.2
2/24/2021	7:00:00 p, m,	17.2	99	0
2/24/2021	8:00:00 p, m,	17	97	0
2/24/2021	9:00:00 p, m,	16.9	96	0
2/24/2021	10:00:00 p, m	16.9	96	0
2/24/2021	11:00:00 p, m	17.1	97	0
2/25/2021	12:00:00 a, m	17.3	96	0
2/25/2021	1:00:00 a, m,	17.4	95	0
2/25/2021	2:00:00 a, m,	17.4	95	0
2/25/2021	3:00:00 a, m,	17.3	95	0
2/25/2021	4:00:00 a, m,	17.2	94	0
2/25/2021	5:00:00 a, m,	17.2	95	0
2/25/2021	6:00:00 a, m,	17.1	93	0
2/25/2021	7:00:00 a, m,	17.3	93	0
2/25/2021	8:00:00 a, m,	17.8	95	0
2/25/2021	9:00:00 a, m,	18.4	95	0
2/25/2021	10:00:00 a, m	18.8	94	0
2/25/2021	11:00:00 a, m	19.2	92	0

2/25/2021	12:00:00 p, m	19	96	0
2/25/2021	1:00:00 p, m,	20.2	91	0
2/25/2021	2:00:00 p, m,	20.5	91	0
2/25/2021	3:00:00 p, m,	19.5	94	0
2/25/2021	4:00:00 p, m,	19.6	92	0
2/25/2021	5:00:00 p, m,	19.9	86	0
2/25/2021	6:00:00 p, m,	18.9	90	0
2/25/2021	7:00:00 p, m,	18.2	92	0
2/25/2021	8:00:00 p, m,	18.2	91	0
2/25/2021	9:00:00 p, m,	18.2	92	0
2/25/2021	10:00:00 p, m	18.2	90	0
2/25/2021	11:00:00 p, m	17.9	91	0
2/26/2021	12:00:00 a, m	17.9	91	0
2/26/2021	1:00:00 a, m,	17.3	91	0
2/26/2021	2:00:00 a, m,	17.3	89	0
2/26/2021	3:00:00 a, m,	17.2	92	0
2/26/2021	4:00:00 a, m,	17.3	91	0
2/26/2021	5:00:00 a, m,	17.3	92	0
2/26/2021	6:00:00 a, m,	17.2	93	0
2/26/2021	7:00:00 a, m,	17.3	92	0
2/26/2021	8:00:00 a, m,	16.9	97	0
2/26/2021	9:00:00 a, m,	17.5	96	0
2/26/2021	10:00:00 a, m	18.2	95	0
2/26/2021	11:00:00 a, m	18.8	95	0
2/26/2021	12:00:00 p, m	19.4	93	0
2/26/2021	1:00:00 p, m,	19.3	94	0
2/26/2021	2:00:00 p, m,	19.2	96	0.2
2/26/2021	3:00:00 p, m,	19.3	95	0
2/26/2021	4:00:00 p, m,	20	96	0
2/26/2021	5:00:00 p, m,	20	95	0
2/26/2021	6:00:00 p, m,	20	97	0.2
2/26/2021	7:00:00 p, m,	19	97	0
2/26/2021	8:00:00 p, m,	18.1	97	0
2/26/2021	9:00:00 p, m,	18	95	0
2/26/2021	10:00:00 p, m	18.1	95	0
2/26/2021	11:00:00 p, m	18	94	0
2/27/2021	12:00:00 a, m	17.9	93	0
2/27/2021	1:00:00 a, m,	18.1	93	0
2/27/2021	2:00:00 a, m,	17.9	93	0
2/27/2021	3:00:00 a, m,	17.9	93	0
2/27/2021	4:00:00 a, m,	17.8	95	0
2/27/2021	5:00:00 a, m,	17.6	96	0.8
2/27/2021	6:00:00 a, m,	16.7	98	0
2/27/2021	7:00:00 a, m,	16.7	98	0
2/27/2021	8:00:00 a, m,	17.1	99	0
2/27/2021	9:00:00 a, m,	17.6	99	0
2/27/2021	10:00:00 a, m	17.8	99	0

2/27/2021	11:00:00 a, m	17.7	99	0
2/27/2021	12:00:00 p, m	17.7	99	0.2
2/27/2021	1:00:00 p, m,	18.3	99	0.2
2/27/2021	2:00:00 p, m,	18.6	99	0
2/27/2021	3:00:00 p, m,	19.8	98	0
2/27/2021	4:00:00 p, m,	19.5	98	0
2/27/2021	5:00:00 p, m,	19.2	98	0
2/27/2021	6:00:00 p, m,	19.2	98	0
2/27/2021	7:00:00 p, m,	18.2	97	0
2/27/2021	8:00:00 p, m,	17.7	98	0
2/27/2021	9:00:00 p, m,	17.4	98	0
2/27/2021	10:00:00 p, m	17.3	98	0
2/27/2021	11:00:00 p, m	17.1	97	0
2/28/2021	12:00:00 a, m	17.2	97	0
2/28/2021	1:00:00 a, m,	17.1	98	0
2/28/2021	2:00:00 a, m,	17.2	97	0
2/28/2021	3:00:00 a, m,	16.8	96	0
2/28/2021	4:00:00 a, m,	16.6	96	0
2/28/2021	5:00:00 a, m,	16.7	97	0
2/28/2021	6:00:00 a, m,	16.6	96	0
2/28/2021	7:00:00 a, m,	17	98	0
2/28/2021	8:00:00 a, m,	17.5	98	0
2/28/2021	9:00:00 a, m,	18	98	0
2/28/2021	10:00:00 a, m	18.4	97	0
2/28/2021	11:00:00 a, m	19.1	97	0
2/28/2021	12:00:00 p, m	19.6	94	0
2/28/2021	1:00:00 p, m,	19.8	95	0
2/28/2021	2:00:00 p, m,	19.8	94	0
2/28/2021	3:00:00 p, m,	20.4	94	0
2/28/2021	4:00:00 p, m,	20.3	94	0
2/28/2021	5:00:00 p, m,	20.3	95	0
2/28/2021	6:00:00 p, m,	19.6	94	0
2/28/2021	7:00:00 p, m,	18.8	95	0
2/28/2021	8:00:00 p, m,	18.3	95	0
2/28/2021	9:00:00 p, m,	17.9	95	0
2/28/2021	10:00:00 p, m	17.9	96	0
2/28/2021	11:00:00 p, m	17.9	95	0
3/1/2021	12:00:00 a, m	17.8	96	0
3/1/2021	1:00:00 a, m,	17.7	97	0
3/1/2021	2:00:00 a, m,	17.3	99	0
3/1/2021	3:00:00 a, m,	17.1	99	0
3/1/2021	4:00:00 a, m,	17	99	0
3/1/2021	5:00:00 a, m,	16.9	99	0.2
3/1/2021	6:00:00 a, m,	16.8	98	0
3/1/2021	7:00:00 a, m,	16.9	99	0
3/1/2021	8:00:00 a, m,	17.2	99	0
3/1/2021	9:00:00 a, m,	17.2	99	0

Feb_21	18.1543988	93.0923754	93

	Temperature	Humidity	Precipitation
Feb_20	19.340	87.483	142.4
Mar_20	18.782	90.860	164.6
Apr_20	19.276	85.867	47
May_20	18.792	89.187	81.2
Jun_20	18.269	89.885	65.8
Jul_20	18.072	87.957	58.4
Aug_20	18.740	80.466	38.4
Sep_20	18.658	80.663	45.8
Oct_20	19.072	78.914	39.6
Nov_20	17.891	94.554	108
Dec_20	17.740	95.622	89.8
Jan_21	17.748	93.476	108.2
Feb_21	18.154	93.092	93
			1082.2
